# The Geometry and Nature of C─I···O─N Interactions in Perfluoroiodobenzene‐Pyridine *N*‐oxide Halogen‐Bonded Complexes

**DOI:** 10.1002/advs.202403945

**Published:** 2024-06-13

**Authors:** Juha Mikko Rautiainen, Arto Valkonen, Jan Lundell, Kari Rissanen, Rakesh Puttreddy

**Affiliations:** ^1^ Department of chemistry University of Jyvaskyla P.O. BOX 35 Jyvaskyla FI‐40014 Finland

**Keywords:** halogen bond, N‐oxide, perfluoroiodobenzene, sigma hole, X‐ray structure

## Abstract

The N─Oxide oxygen in the 111 C─I···⁻O─N^+^ halogen bond (XB) complexes, formed by five perfluoroiodobenzene XB donors and 32 pyridine N‐oxides (PyNO) XB acceptors, exhibits three XB modes: bidentate, tridentate, and monodentate. Their C─I···O XB angles range from 148° to 180°, reflecting the iodine σ‐hole's structure‐guiding influence. The I···⁻O─N^+^ angles range from 87° to 152°. On the contrary, the I···⁻O─N^+^ angles have a narrower range from 107° to 125° in stronger monodentate N─I···⁻O─N^+^ XBs of N‐iodoimides and PyNOs. The C─I···⁻O─N^+^ systems exhibit a larger variation in I···⁻O─N^+^ angles due to weaker XB donor perfluoroiodoaromatics forming weak I···O XBs, which allows wider access to electron‐rich N‐O group regions. Density Functional Theory analysis shows that I···O interactions are attractive even when the I···⁻O─N^+^ angle is ≈80°. Correlation analysis of structural parameters showed that weak I···O XBs in perfluoroiodobenzene‐PyNO complexes affect the C─I bond via n(O)→σ*(C─I) donation less than the N─I bond via n(O)→σ*(N─I) donation in very strong I···O XBs of N‐iodoimide‐PyNO complexes. This implies that PyNOs' oxygen self‐tunes its XB acceptor property, dependent on the XB donor σ‐hole strength affecting the bonding denticity, geometry, and interaction energies.

## Graphical abstract

The analysis of 111 C–I···⁻O─N^+^ halogen‐bonded complexes formed by perfluoroiodobenzenes and pyridine N‐oxides has provided conclusive evidence that I···O halogen bonds are narrowly directional as per the halogen σ‐hole theory. The directionality observed from the N‐oxides viewpoint is significantly larger due to their N‐oxide oxygen atom's polydentate feature.

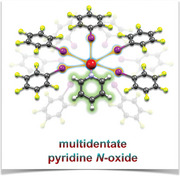

## 1 Introduction

Non‐covalent interactions (NCIs) are ubiquitous in a wide variety of soft and crystalline materials, both natural and artificial, but the manipulation of NCIs to produce desirable materials is a relatively new area of research. However, most NCIs are weak and lack the directionality and strengths of covalent bonds. Therefore, the approach of modifying the functional properties of materials by changing single NCIs is seldom appropriate since a palette of interactions works together to determine these features.^[^ ^1^ ^]^ In such cases, directional NCIs tend to be given special consideration, and the hydrogen bond (HB)^[^ ^2^ ^]^ is the most exploited directional NCI in the supramolecular toolbox.^[^ ^3^ ^]^ The importance of HBs in chemical and life processes has prompted substantial research, and HBs have been used to engineer myriad systems, e.g., porous organic frameworks,^[^ ^4^ ^]^ conductor properties in organic electronics,^[^ ^5^ ^]^ and the functions of soft robots.^[^ ^6^ ^]^ Over the last few decades, halogen bonding (XB)^[^ ^7^ ^]^ has emerged as a promising substitute for HB in organic catalysis,^[^ ^8^ ^]^ and biological systems.^[^ ^9^ ^]^ Although HB and XB share similar properties, the properties of materials significantly change when a donor molecule's hydrogen atom is replaced by a halogen atom. This change in behavior is caused primarily by the large σ‐hole of the polarized halogen atoms complemented with heavy atom effects.^[^ ^10^ ^]^

Proficiency in XB engineering of materials requires knowledge of the underlying features of XB, viz. the geometries of the complexes and the nature of the bonding, as well as whether the XB complex can be controllably fine‐tuned to optimise its attributes. In this regard, unarguably the I···N_Py_ (Py = Pyridine) halogen‐bonded complexes have emerged as iconic examples for various applications such as bending crystals,^[^ ^11^ ^]^ and functional soft materials.^[^ ^12^ ^]^ To give one example, 1,4‐diiodotetrafluorobenzene (pDIB) and a polymerizable acrylate group containing pyridine were used as XB building blocks to create stimuli‐responsive shape memory polymers.^[^ ^13^ ^]^ This was made possible by the extensive experimental and theoretical knowledge of small molecule C─I···N_Py_ halogen‐bonded complexes of pDIB and pyridines.^[^ ^14^ ^]^ These small molecule pyridine‐based D–X···N_Py_ (D = C, N; X = Cl, Br, I) halogen‐bonded systems can be broadly divided into three classes. 1) D–X···N_Py_, where the halogen is attached to a non‐fluorinated organic structure; 2) D_F_–X···N_Py_, in which the halogen is attached to a fluorinated organic structure, and 3) _Py_N···X⁺···N_Py_ complexes with positively charged halogen trapped between two pyridines. These three classes are well‐recognised for their divergent uses. For example, class *1* C─I···N halogen‐bonded systems have been employed in the synthesis of phosphorescent materials,^[^ ^15^ ^]^ the dynamic nature and fluorine content of the class *2* C_F_–I···N systems make them suitable for liquid crystals,^[^ ^16^ ^]^ and class *3* [N···I···N]⁺ systems are used in the preparation of supramolecular capsules,^[^ ^17^ ^]^ porous structures,^[^ ^18^ ^]^ and as halogenating reagents in organic synthesis.^[^ ^19^ ^]^

Pyridine *N*‐oxides (PyNOs) have an aromatic ring similar to pyridine, but because they contain an N─O group, their electronic ring structure is very different. There are at least three main differences between pyridines and PyNOs. i) The oxygen of the N−O group can push and pull electrons into and out of the aromatic ring.^[^ ^20^ ^]^ ii) The zwitterionic nature of the *N*‐oxide group generally results in large dipole moments.^[^ ^21^ ^]^ iii) As ambivalent species, PyNOs are useful for both electrophilic and nucleophilic substitutions at 2‐, 4‐, and 6‐positions.^[^ ^20^ ^]^ Despite these unique features, PyNOs have been appreciated only as valuable synthetic intermediates in the preparation of pyridines.^[^ ^22^ ^]^ As a result, much of the innovative research and breakthroughs in PyNO chemistry have been in the field of organic synthesis, thus leaving their potential in supramolecular materials underexplored. The paucity of information on, e.g., *N*‐oxide oxygen's bonding geometries and the types of supramolecular assemblies they can form with different molecules limits their applicability. The few reports where I···O(PyNO) halogen bonds have been discussed from a crystal engineering perspective include the works by Resnati and co‐workers,^[^ ^23^ ^]^ Rosokha and co‐workers,^[^ ^24^ ^]^ Jin and co‐workers,^[^ ^25^ ^]^ and Aakeröy and co‐workers.^[^ ^26^ ^]^

**Figure 1:**
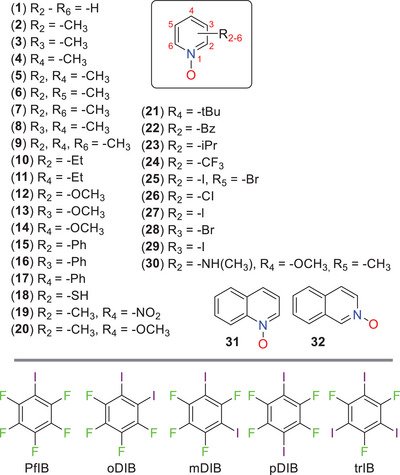
The chemical structures of pyridine *N*‐oxides (**1**‐**32**) as XB acceptors and perfluoroiodobenzene (**PfIB**), 1,2‐diiodotetrafluorobenzene (**oDIB**), 1,3‐diiodotetrafluorobenzene(**mDIB**), 1,4‐diiodotetrafluorobenzene(**pDIB**), and 2,4,6‐triiodotrifluorobenzene(**trIB**) as XB donors.

In this study, we explore the nature of I···O interactions formed by perfluoroiodoaromatics and PyNO as crystalline materials, motivated by the observation these I···O XBs are dynamic and comparable to the commonly utilised I···N XBs in functional materials. An in‐depth experimental and theoretical study outlining their bonding properties would aid their use in the rational design of, e.g., drug molecules and the preparation of functional materials with desirable characteristics. The task is complicated by the fact that *N*‐oxide oxygen has three electronpairs and a propensity to form polydentate interactions. This could make it difficult to properly describe its bonding nature if the study relies on a limited number of XB complexes since different donor‐acceptor partners may show distinct bonding geometries and offer unique structural information. We thus comprehensively investigated the I···O interactions of 111 halogen‐bonded complexes formed by five perfluoroiodoaromatic donors and thirty‐two PyNO acceptors (**Figure** **1**). A complication can arise as perfluoroiodoaromatics usually exhibit complex (and often non‐linear) interactions with one another driven by F···F and *π*–*π* interactions.^[^ ^27^ ^]^ In this regard, the large dataset helps to understand the similarities of XB bond building blocks that these model systems may generate.

## 2 Results and Discussion

**Figure 2:**
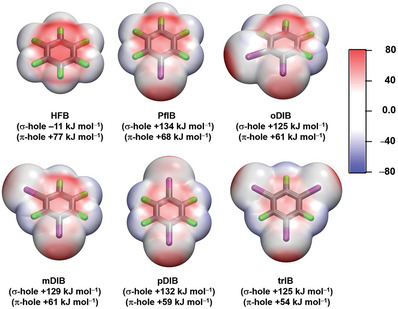
Computed electrostatic potential (ESP) surfaces at the PBE0‐D3/def2‐TZVP level of theory projected on the 0.001 au electron density surfaces of perfluoroiodobenzenes with *V*_S,max_ values for **HFB**, **PfIB**, **oDIB**, **mDIB**, **pDIB**, and **trIB**.

Perfluoroiodoaromatics can manifest two main types of intermolecular interactions, the σ‐hole interactions of the iodine donor complemented with possible π‐π interactions. When an electron‐withdrawing fluorine group is added onto the aromatic ring of an iodine‐based XB donor, the electrostatic potential at the 0.001 au electron density surface (ESP, V_s,max_) is typically altered by π‐resonance and inductive/field effects. In order to investigate the relationship between the σ‐/π‐hole^[^ ^28^ ^]^ strengths and the halogen substitution patterns in the used perfluoroiodoaromatics, electrostatic potential (ESP) maps were computed at the PBE0‐D3/def2‐TZVP^[^ ^29^ ^]^ level of theory. Hexafluorobenzene (**HFB**) ESP map of V_s,max_ was calculated to provide a reference for the π‐hole strengths (**Figure** **2**). **PfIB** and **pDIB** have the largest σ‐hole strengths while **mDIB** has an intermediate and **oDIB** and **trIB** have the smallest V_s,max_ values. The π‐hole values range from +54 to +68 kJ mol^−1^, with **PfIB** being the largest and **trIB** the smallest. The overall σ‐/π‐hole strengths are largely determined by the electronegativity of the halogen; however, it should be noted that the fluorine substitution pattern that alters dipole moments will also affect the σ‐/π‐hole strengths.^[^ ^30^ ^]^ The ESP analysis offers the following helpful insights: i) The iodine σ‐hole is much more positive than the π‐hole. ii) The σ‐ and π‐holes are the two key NCIs that potentially control the donor‐acceptor molecular recognition in the crystal structures in addition to π‐π stacking and the propensity for fluorine atoms to pack together in crystal structures.

**Figure 3:**
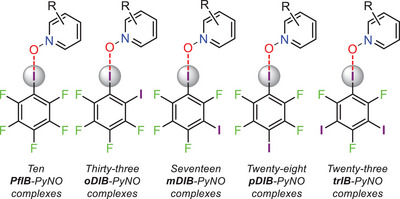
The number of isolated perfluoroiodobenzene‐PyNO halogen‐bonded complexes.

Out of a total of 128 X‐ray structures (see Figures S2–S129, Supporting Information), 111 were halogen‐bonded complexes (Figures S2–S113, Supporting Information). These XB complexes were crystallized either from dichloromethane, chloroform or acetone using a 1:1 donor:acceptor equivalent ratio and slow evaporation of the solvent (for details, see Supporting Information). Except for **pDIB**‐**28**, **trIB**‐**16**, and **trIB**‐**17**, no solvent molecules were found in their structures. The physical properties of XB donors appeared to have the biggest impact on the success in crystallizing the XB complexes, solvent and other experimental conditions having a lesser impact. At room temperature, **PfIB** is a liquid and **mDIB** is a semisolid, whereas **oDIB**, **pDIB**, and **trIB** are solids. The XB complexes of non‐solid XB donors are challenging to crystallize compared to the solid ones. For instance, out of 111 determined crystal structures, only 10 were from liquid **PfIB** but 33 crystal structures could be determined for complexes from the solid **oDIB** (**Figure** **3**). The **PfIB**‐PyNOs form only discrete 1:1 and 2:1 donor:acceptor complexes, while **oDIB**‐PyNOs form two types of complexes viz. 2:2 donor:acceptor macrocycles and polymers, the **mDIB**‐, **pDIB**‐ and **trIB**‐PyNOs form both discrete and polymeric complexes (e.g., See Figures S130–S134, Supporting Information). In addition to dichloromethane, chloroform or acetone solvent conditions, the selected **oDIB**‐**1**/**2**/**4**/**7**/**31**/**32** and **pDIB**‐**1**/**2**/**4**/**7**/**31**/**32** complexes with 1:1 donor:acceptor equivalent ratios were crystallized from various solvents, including methanol, ethanol, tetrahydrofuran, ethyl acetate, and dimethylformamide, to investigate the impact of solvents on the robustness of the halogen‐bonded complexes. Despite the varying polarities of the above solvents, all complexes crystallize similarly to those from dichloromethane, chloroform and acetone, with no solvent molecules.

**Figure 4:**
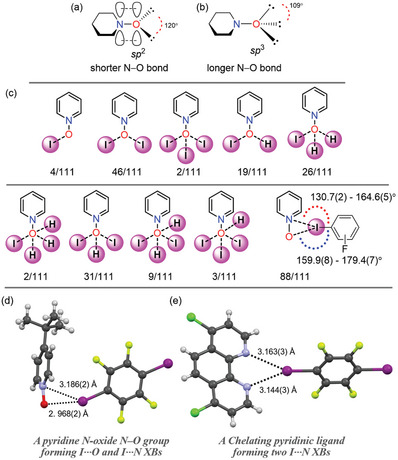
a,b) A representation of N‐oxide oxygens sp^2^ and sp^3^ hybridizations c) A summary of PyNOs bonding modes observed in the 111 halogen‐bonded complexes. Denotation:, e.g., 4/111 means, the N−O group participating in C−I···⁻O−N+ interactions was observed 4 times in 111 XB complexes. This notation is employed since some asymmetric units have more than one PyNO. d,e) Comparison of XB chelating modes of the PyNO and phenanthroline with pDIB (CCDC code: TAWFOP).^[^ ^34^ ^]^

The oxygen atom in PyNO can exhibit both *sp* ^2^ and *sp* ^3^ characters due to the zwitterionic nature of the N−O group, as shown in **Figure** **4**a,b.^[^ ^31^ ^]^ Thus, in contrast to nitrogen in pyridines, the oxygen in PyNO can act as a two or three electron pair donor, allowing them to manifest polydentate interactions., i.e., interacting with one, two, or three electron acceptors.^[^ ^32^ ^]^ Furthermore, the N‐ and O‐atoms of the N−O group are electron rich due to the N←O back donation property.^[^ ^31a^ ^]^ Figure 4c shows the possible interaction modes and their frequency of occurrence in the studied X‐ray structures. In addition to XB and XB+HB hybrid modes, the N−O groups of ligands **19** and **28** in **oDIB**‐**19** and **oDIB**‐**28**, do not form I···O XBs, and they exhibit only monodentate and bidentate C−H···**⁻**O−N^+^ interactions, respectively (Figure S135, Supporting Information). It should be noted that C−H···**⁻**O−N^+^ short contacts bring significant additional stabilization to the crystalline materials. The **oDIB**‐PyNO complexes form 2:2 donor:acceptor macrocycles, which firmly stack on top of one another, as a result, the PyNOs in these complexes only form µ_2_‐*O*,*O* XB mode and (**oDIB**)C−F···H−C(PyNO) HBs instead of typical (PyNO)C−H···**⁻**O−N^+^ HBs (See Figure S136, Supporting Information). The nitrogen of N−O groups and iodine donors exhibits I···N short contacts at distances ranging from 3.186(3) to 3.534(5) Å that are less than the sum of the van der Waals radii of N‐ and I‐atoms (3.53 Å) as shown in Figure 4c,d. Such short distances could be attributed to attractive forces, e.g., due to dispersion interactions or packing arrangement in the crystal structures. A quantum theory of atoms in molecules^[^ ^33^ ^]^ test calculation on the **pDIB‐21** complex (Figure 4d; Figure S150, Supporting Information) did not show any bond path between N‐ and I‐atoms which suggests that packing forces and/or long range electrostatic attractive interactions are more likely the reason for the N···I short contacts. Nevertheless, similar XB distances range was observed in perfluoroiodobenzene complexes of chelating‐type ligands, e.g., phenanthroline (Figure 4e).^[^ ^34^ ^]^

**Figure 5:**
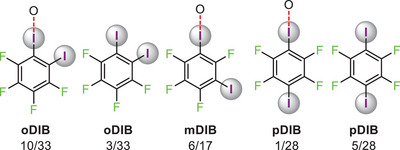
A summary of C−I group denticity of **oDIB**, **mDIB**, and **pDIB** donors. Denotation:, e.g., 10/33 means, one C−I group participating in C−I···⁻O−N^+^ interactions was observed 10 times in 33 **oDIB**‐PyNO complexes. This notation is used because some asymmetric units have multiple **oDIB** donors. Note: The thione form of 18 XBs are not included in these statistics since they generate I···S XBs (See Figure 10). The data correspond to 78 crystal structures.

The iodine atom of the **PfIB** participates in the C−I···**⁻**O−N^+^ interactions in all **PfIB**‐PyNO complexes. Out of 33 **oDIB**‐PyNO complexes, in ten of them, one of the **oDIB**’s iodine atoms does not form C−I···⁻O−N^+^ interaction and in three cases both iodine atoms do not form XBs at all (**Figure** 5). Out of 17 **mDIB**‐PyNO complexes, in six of them, one of the **mDIB**’s iodine atoms does not form a C−I···⁻O−N^+^ interaction. Within the 28 **pDIB**‐PyNO complexes, in one case one C−I···⁻O−N^+^ interaction is formed while in five cases C−I···⁻O−N^+^ interactions are not observed at all. In all **trIB**‐PyNO complexes, only two C−I···⁻O−N^+^ interactions are observed. The iodine atoms that do not form I···O interactions stabilize the lattice by weak C−I···I’ (I’ = XB acceptor) and C−I···H interactions.

The **trIB**‐**1**, **trIB**‐**2**, **trIB**‐**4**, and **trIB**‐**19** were crystallized in chloroform with different equivalent donor:acceptor ratios (1:3, 1:6, and 1:9) to determine whether all three iodines of trIB can form the I···O XBs. Regrettably, none of the tested complexes differed from those synthesized by the 1:1 ratio, showing only two I···O XBs. Yet in acetone solvate complexes **trIB**‐**16** and **trIB**‐**17**, the two iodines participate in C−I···⁻O−N^+^ interactions with PyNOs, while the third iodine shows the C−I···O═C interaction with acetone (**trIB**‐**16**; 2.904(3) and **trIB**‐**17**; 3.033(4) Å, Figure S137, Supporting Information).

A detailed geometrical analysis was carried out for C−I···⁻O−N^+^ XBs using the C−I and N−O covalent bond lengths, the I···O XB distances, the C···O distances, the C−I···O XB angles, and the I···**⁻**O−N^+^ angles as parameters (See Tables S1–S5, Supporting Information). The I···O XB distances have quite a narrow range, from 2.648(2) to 2.999(2) Å, with only seven I···O distances exceeding 3.0 Å (up to 3.252(4) Å). The shortest I···O XB distance is found in **PfIB**‐**20** (2.648(4) Å) corresponding to a monodentate halogen bond. In systems with bidentate XBs, one I···O distance is usually <0.25 Å shorter than the other. For example, in **oDIB**‐**15**, the I···O XBs are 2.735(2) and 2.852(2) Å, with a difference of 0.117 Å.

To investigate the differences between the weakest and the strongest C−I···**⁻**O−N^+^ halogen‐bonded systems, the interaction energies, Δ*E*_int_, of the XB complexes with the shortest and the longest experimental I···O XB distances were calculated at the PBE0‐D3/def2‐TZVP^[^ ^29^ ^]^ level of theory and given in **Table** **1**. The I···O XB distances from DFT and crystal structures are generally similar, with a maximum difference of 0.156 Å observed in **oDIB**‐**19**. In comparison to the N−I···**⁻**O−N^+^ XBs formed by N‐haloimides and PyNOs, which exhibit a large variation in the *E*_int_(from –120 to –56 kJ mol^−1^),^[^ ^35^ ^]^ the **Δ** *E*_int_ values of strong and weak XBs for each perfluoroiodobenzene donor have a narrow range and are comparable (**Table** **2**).

**Table 1:** Calculated interaction energies (**Δ** *E*_int_, kJ mol^−1^) for shortest and longest XBs in perfluoroiodoarene‐PyNO XB complexes at PBE0‐D3/def2‐TZVP level of theory.

code	I⋅⋅⋅O distances [Å]		Δ*E*_int_
	XRD	DFT	
**PfIB‐20**	2.648(2)	2.698	−40.2
**PfIB‐15**	2.841(2)	2.790	−38.5
**oDIB‐8**	2.706(4)	2.748	−35.4
**oDIB‐19**	2.850(4)	3.006	−30.4
**mDIB‐8**	2.652(6)	2.734	−35.3
**mDIB‐15**	2.818(3)	2.790	−27.8
**pDIB‐1**	2.692(3)	2.768	−32.2
**pDIB‐24**	2.948(3)	2.979	−20.2
**trIB‐13**	2.696(2)	2.767	−31.9
**trIB‐29**	2.889(4)	2.815	−29.0

**Table 2:** Experimental Raman frequencies for **PfIB**, **oDIB**, **mDIB**, **pDIB**, **trIB**, and their XB complexes with the shortest I···O distances.

code	*v* [cm^−1^]	code	*v* [cm^−1^]
**PfIB**	206	**PfIB‐20**	199
**oDIB**	235	**oDIB‐20**	230
**mDIB**	201	**mDIB‐8**	192
**pDIB**	158	**pDIB‐9**	148
**trIB**	175	**trIB‐13**	171

The **Δ** *E*_int_ values of **oDIB**‐**8**, which contains a PyNO with electron‐donating methyl groups, and **oDIB**‐**19**, which has a PyNO with electron‐withdrawing nitro and methyl groups, are similar. This can be attributed to the weak electron accepting capabilities of perfluoroiodoarenes. The *N*‐oxide oxygen electron density does, however, appear to play a minor role in determining the strengths of I···O interactions. For instance, the **Δ** *E*_int_ value of **pDIB**‐**1**, which contains a parent PyNO is 12 kJ mol^−1^ larger than **pDIB**‐**24**, which has an electron‐withdrawing *ortho*‐CF_3_ group. In general, the weakest and strongest C−I···**⁻**O−N^+^ XBs have energies between −20 and −40 kJ mol^−1^.

**Figure 6:**
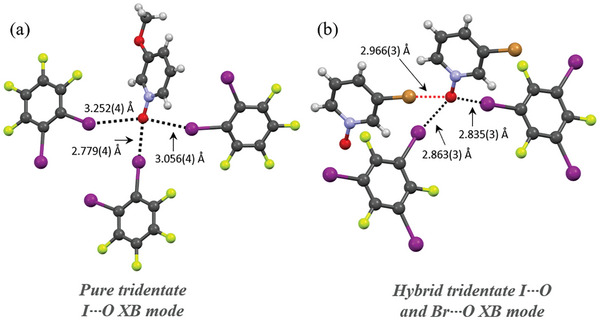
Tridentate XB modes of a) **oDIB**‐**13** and b) **trIB**‐**28**. The black broken line represents the I···O XBs and the red broken line the Br···O XBs.

Five complexes contain tridentate X···O XBs, which can be divided into three classes. i) “’Pure”’ tridentate I···O XBs formed in **oDIB**‐**13** between the C−I groups of three **oDIB** donors and the *N*‐oxide oxygen, as shown in **Figure** **6**a and Figure S138 (Supporting Information). ii) Pure tridentate I···O XBs in **pDIB**‐**29**, **mDIB**‐**29**, and **trIB**‐**29**, where one of the I···O XBs are formed between the *N*‐oxide oxygen and the C−I group of another 3‐iodopyridine *N*‐oxide (**29**) and two are formed to the C−I groups of two corresponding perfluoroiodobenzene donors. (iii) Heteroleptic tridentate bonds found in **trIB‐28** between *N*‐oxide oxygen and the C−I groups of two **trIB** and the C−Br group of a second 3‐bromopyridine *N*‐oxide as shown in Figure 6b. Thus, **trIB**‐**28** is a hybrid tridentate I···O and Br···O halogen‐bonded system. Surprisingly, halogen substituents of 2‐iodo‐5‐bromopyridine *N*‐oxide (**25**) and 2‐iodopyridine *N*‐oxide (**27**) complexes do not exhibit such tridentate X···O interactions in their XB complexes. This suggests that 3‐halopyridine *N*‐oxides are XB acceptors that have the propensity to form these uncommon tridentate XBs (See Figure S147, Supporting Information for complexes that form I···O interactions in addition to the N−O group). In tridentate complexes, the subsequent I···O XB distances show an increasing trend. For instance, in **oDIB**‐**3**, the shortest I···O distance is 2.779(4) Å, while the other two are 3.056(4) and 3.252(4) Å. The increase in I···O distances with an increasing number of bonds can be caused by one of two factors: i) The electron density of the O‐atom diminishes following the development of a XB mode, making the next XB mode comparatively weaker, and/or ii) the iodine atoms are unable to fit closer to the *N*‐oxide oxygen due to steric restraints.

The XB complexation causes a small but noticeable C−I bond elongation. The C−I bond elongation can be described as Δ(C−I) = (C−I)_complex_ – (C−I)_ligand_, and is at maximum 0.04 Å for **PfIB**, **oDIB**, and **trIB**, while for **mDIB** and **pDIB** it is 0.03 Å (Tables S1–S5, Supporting Information). The Δ(C−I) values are small due to their weak XB complexation capabilities and are comparable to Δ(C−I) values observed in, e.g., C−I···N,^[^ ^36^ ^]^ C−I···S,^[^ ^37^ ^]^ and C−I···Y**⁻** (Y = Cl, Br)^[^ ^38^ ^]^ XBs. The nature of N−O bond distances of PyNOs has been extensively investigated in the literature.^[^ ^31^ ^]^ Electron‐withdrawing groups, such as ‐NO_2_, pull electron density away from the N−O group, imparting it a double bond character, whereas electron‐donating groups, such as ‐OCH_3_, have the opposite effect. In C−I···**⁻**O−N^+^ XB complexes, the N−O bond distances vary between 1.266(10) and 1.381(3) Å (Tables S1–S5, Supporting Information), the shortest is observed for 2‐methyl‐4‐nitropyridine *N*‐oxide (**19**) in **oDIB**‐**19**, and the longest for thione form of 2‐mercaptopyridine *N*‐oxide (**18**) in **pDIB**‐**18**. In both cases, their N−O groups are not participating in C–I···**⁻**O−N^+^ XBs. The short and double‐bond character of the N−O group in **oDIB**‐**19** is attributable to the electron‐withdrawing −NO_2_ group and the longer and single bond feature in **pDIB**‐**18** to the thione/SH form. The N−O distances of the N–O groups engaged in the I···O interaction are found to be the shortest in **mDIB‐19** (1.297(5) Å) and the longest in **pDIB**‐**14** (1.349(17) Å). It should be noted that the O→N *π*‐back‐donation can also be affected by bonding at the *N*‐oxide oxygen, observed in metal complexes, and that this phenomenon is independent of substituents.^[^ ^31b^ ^]^ This feature is also observed in perfluoroiodobenzene‐PyNO XB complexes. For instance, the 3‐methylpyridine *N*‐oxide (**3**) exhibits different N−O bond lengths in **oDIB‐3** (1.348(10) Å) and **mDIB**‐**3** (1.315(4) Å), suggesting that the changes are caused by XB complexation rather than the *meta*‐methyl group.

**Figure 7:**
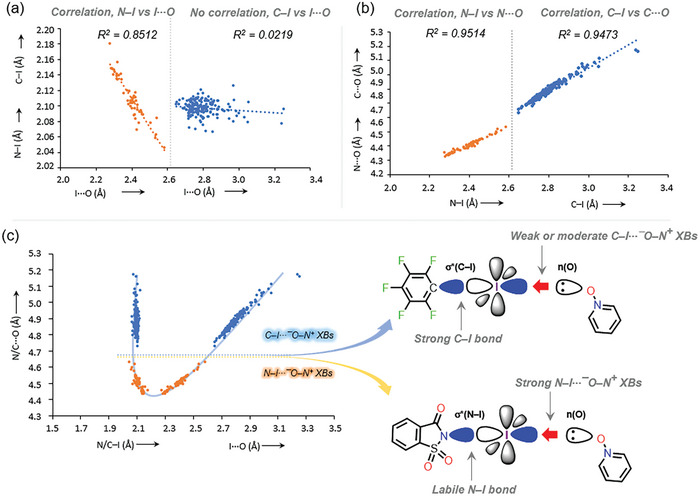
Comparison of correlations analysis crystal structure bond parameters of C−I⋯⁻O−N⁺ and N−I⋯⁻O−N⁺ halogen‐bonded systems: a) N−I versus I⋯O distances and C−I versus I⋯O distances, and b) N−I versus N⋯O distances and C−I versus C⋯O distances. c) Correlation between N/C–I and I···O versus N/C···O distances of C−I⋯⁻O−N⁺ and N−I⋯⁻O−N⁺ halogen‐bonded complexes. Note: i) The data on N−I⋯⁻O−N⁺ halogen‐bonded complexes include N‐iodosuccinimide‐PyNOs, N‐iodophthalimide‐PyNOs and N‐iodosaccharin‐PyNOs, and N‐Iodosaccharin‐PyNOs is shown is used as a model for illustration of the data. ii) As the protonated N─O groups of **oDIB/pDIB/trIB‐18** are not involved in XB, their data are included in the analysis and correspond to 108 halogen bonded complexes. iii) The linear regression equations for N−I versus I⋯O and C−I versus I⋯O correlation analysis of Figure 7a are y = −0.360x + 2.9736 and y = −0.0143x + 2.137, respectively, and for N−I versus N⋯O distances and C−I versus C⋯O of Figure 7b are y = −6323x + 2.9791 and y = −0.8095x + 2.6183, respectively.

In addition to electrostatic interactions, some halogen‐bonded systems have been shown to exhibit significant covalent character due to charge transfer from a Lewis base to σ* of C−I species of XB donor.^[^ ^39^ ^]^ If there is a significant covalent component to the I···O XB in pentafluorobenzene‐PyNO systems, the n(O)→σ*(C−I) electron donation should directly affect the C−I bond length as electron population of the σ* weakens the C−I bond. In such case, there should be a correlation between C−I and I···O bond parameters. The NBO analysis of **PfIB‐1** revealed that the N‐oxide oxygen lone‐pair with p‐character transfers an amount of 0.06e charge into the σ* orbital of the C−I bond, with a stabilization energy of −26 kJ mol^−1^ (Figure S151, Supporting Information). However, there is no correlation between C−I and I···O bond parameters (R^2^ = 0.022, **Figure** **7**a), as there is little change in C−I bond distances (2.06 – 2.12 Å) compared to changes in I···O distances (2.60–3.22 Å) and XB systems with similar C−I bond distances can have significantly different I···O distances (e.g., **PfIB‐15**: 2.091(2) Å, 2.841(2) Å and **PfIB‐16**: 2.090(2) Å, 2.720(7) Å). In contrast, strong N−I⋯⁻O−N⁺ halogen‐bonded systems formed by N‐iodoimides and PyNOs shown a much higher correlation for N–I versus I···O distances (R^2^ = 0.851) indicating larger n(O)→σ*(N−I) electron donation by stronger I⋯O XBs.^[^ ^35^ ^]^ Note that upon assessing several correlations (Figures S139–S143, Supporting Information), only the C···O versus I···O distances showed a correlation (R^2^ = 0.956). A similar correlation was observed for N···O versus I···O distances of strong N−I⋯⁻O−N⁺ halogen‐bonded systems (R^2^ = 0.951, Figure 7b).

In our previous report, a hypothetical symmetrical point for N−I⋯⁻O−N⁺ halogen‐bonded systems were estimated by plotting N–I and I···O distances against N···O distances. The N−I⋯⁻O−N⁺ data showed a parabolic curve, with the minimum occurring when N–I = I···N, at ≈4.4 Å, meaning, symmetrization occurs when both N–I and I–O bond distances are 2.22 Å with an N···O separation of ≈4.4 Å.^[^ ^35^ ^]^ Upon plotting the C–I and I···O distances against C···O distances, the C−I⋯⁻O−N⁺ data (blue dots) positioned over the N−I⋯⁻O−N⁺ data (orange dots) as shown in Figure 7c. The C−I⋯⁻O−N⁺ systems are much farther away from a symmetric C···I···O bonding situation due to the weaker σ‐hole strength of iodine in perfluoroiodoaromatics. This comparison of perfluoroiodobenzene‐PyNOs and N‐haloimide‐PyNOs halogen‐bonded complexes demonstrates that XB donors greatly influence the XB strength properties, and *N*‐oxide oxygen cannot form strong XBs unless there is sufficient σ‐hole strength available on the XB donor such as those in N‐haloimides. The most significant finding is that their data demonstrate a correlation even if their C/N–I elongations and I···O distances vary.

**Figure 8:**
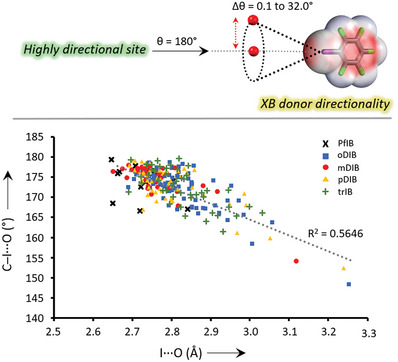
(above) The XB directionality depicted using **PfIB** donor (below) Correlation between C−I⋯O XB angles versus I⋯O distances. The data correspond to 108 crystal structures.

**Figure 9:**
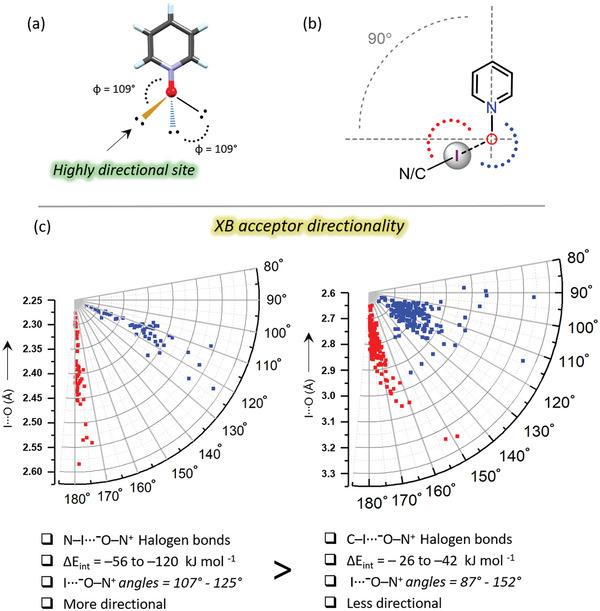
a) Tetrahedral arrangement of *N*‐oxide oxygen lone pair lobes, b) depiction of I⋯⁻O−N⁺ XB angles and C−I⋯O XB angles c) Comparison of XB acceptor directionality N−I⋯⁻O−N⁺ and C−I⋯⁻O−N⁺ complexes via N−O⋯I (blue points) and C/N−I⋯O XB angles (red points) parameters. The data of Figure 9c (left side) correspond to 75 crystal structures, and Figure 9c (right side) to 108 crystal structures.

The main structural property of the XB is its linearity. The deviation from linearity, defined as the absolute Δθ = 180 – θ_complex_, is used to analyze the deviation from the ideal XB angle. The Δθ of **PfIB** complexes ranges from 0.6 to 13°, **oDIB** from 1.2 to 32°, **mDIB** from 2 to 26°, **pDIB** from 0.8 to 20°, and **trIB** from 0.1 to 18.5°. The ∠C–I···O angles versus I···O distances are plotted to obtain a broader insight into the distribution of ∠C–I···O XB angles, as shown in **Figures** **8** and **9**. At ≈2.70–2.85 Å distances, the scatter plot shows a densely populated region that is more linear (≈175°–179°). At longer distances ≈2.90–3.10 Å, there is a region of random scatter, which corresponds to bidentate XBs with angles ranging from ≈150 to 171°. At ≈3.20‐3.30 Å, the two well‐isolated points (orange triangle and blue square) correspond to tridentate XBs with high Δθ values of 28° and 32°. **oDIB** and **pDIB** complexes appear to deviate notably more from linearity when compared to **PfIB**, **mDIB**, and **trIB** complexes. The reason for this cannot be attributed to the proximity effect of the XB donor iodine substituents, as the ∠C–I···O angles of complexes of **oDIB** with a 60° angle and **pDIB** with a 180° angle between the two iodine donor sites, are both widely distributed. More likely the difference comes from packing forces.

The I···**⁻**O−N^+^ angles are used to investigate the XB directionality from the XB acceptor perspective. The lone pair lobes of *sp* ^3^ hybridized *N*‐oxide oxygen have a tetrahedral arrangement, forming angles of ≈109° (Figure 9a). The I···O XBs are considered directional when they form I···**⁻**O−N^+^ XB angles close to 109°. In the case of strong N−I···**⁻**O−N^+^ XBs (Δ*E*_int_ = –56 to –120 kJ mol^−1^) formed by N‐haloimides and PyNOs the I···**⁻**O−N^+^ XB angles, that range from 107° to 125°, are close to tetrahedral angles and can be classified as highly directional, as shown in Figure 9c. The I···**⁻**O−N^+^ (blue points) and N−I⋯O XB angles (red points) form two well‐separated groups. Notably, the I···**⁻**O−N^+^ XB angles in strong N−I···**⁻**O−N^+^ halogen‐bonded systems are comparable to those of M···**⁻**O−N^+^ valence angles in *N*‐oxide metal complexes, which typically vary between 103° and 141°.^[^ ^31b^ ^]^ In contrast for weaker C−I···**⁻**O−N^+^ halogen‐bonded systems (Δ*E*_int_ = –20 to –40 kJ mol^−1^) the I···**⁻**O−N^+^ XB angles vary in much larger range between 87° and 152°; the minimum of the range is perpendicular to the PyNO backbone, with the maximum approaching the C−I⋯O XB angle (Figure 9c; Tables S1–S5, Supporting Information). Both the I···**⁻**O−N^+^ XB angles (blue points) and C−I⋯O XB angles (red points) are dispersed, and the groups diffuse into each other. These more substantial deviations (of directionality from the viewpoint of XB donor and acceptor) in weaker halogen‐bonded systems suggest that: i) the stronger electrostatic attraction exhibited by iodine of N‐iodoimides and the *N*‐oxide oxygen favor tetrahedral XB angles; and ii) the weaker interaction exhibited by iodine of perfluoroiodobenzenes and *N*‐oxide oxygen allows the XB donor σ‐holes a much wider range of access to the electron density‐rich regions of the *N*‐oxide oxygen.

**Figure 10:**
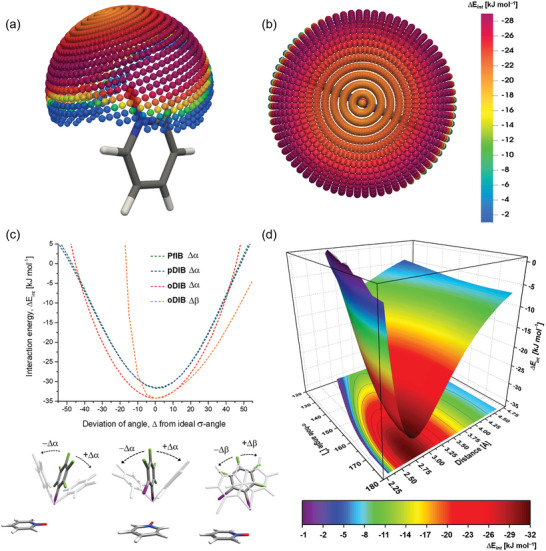
Interaction energy, Δ*E*_int_ scans calculated at PBE0‐D3/def2‐TZVP level of theory. (a) side and (b) top views of Δ*E*_int_ of **PfIB**‐PyNO complexes as a function of **PfIB** positions around PyNO at an I···O distance constrained to that of the optimized complex (2.904 Å). Colored spheres indicate the positions of **PfIB** iodine atoms and colors map the Δ*E*_int_ values. (c) Δ*E*_int_ dependence on angle deviations from the ideal ∠C−I···O in **PfIB**‐PyNO, **pDIB**‐PyNO, and **oDIB**‐PyNO complexes. (d) Interdependence of Δ*E*_int_ on I···O distance and σ‐hole ∠C−I···O in **PfIB**‐PyNO model system. There are 1446 energies in Figure 10a,b (gas‐phase models) and 1260 energies in Figure 10d.

Inspired by these findings, three aspects of bonding were examined by DFT calculations: i) the favorable and unfavorable XB locations surrounding the *N*‐oxide oxygen. ii) The relationship between interaction energy, Δ*E*_int_ and deviations of XB from the ideal direction of the σ‐hole. iii) The relationship between deviations from the optimal I···O distance and Δ*E*_int_. The 1^st^ aspect is illustrated in **Figure** **10**a,b using a complex of **PfIB** and parent PyNO as a model, constraining the I···O distance to that of the optimized complex, and scanning the Δ*E*_int_ as a function of **PfIB** positions on a sphere around the oxygen atom of PyNO. The latter two aspects were studied using complexes of **PfIB**, **pDIB**, and **oDIB** with parent PyNO as model systems.

The results of the spherical analysis demonstrate that the most favorable XB interactions occur between 100° and 145°, with energy ranging from −25.0 to −29.5 kJ mol^−1^, as shown by the red and purple spheres in Figure 10a,b. The ring of the most favorable interactions is reminiscent of the observations reported earlier for C−I···O−C XBs in halobenzene N‐methylacetamide systems.^[^ ^40^ ^]^ The XB interactions parallel to the extension of the *N*‐oxide N−O bond are notably weaker but still attractive with Δ*E*_int_ values close to −21 kJ mol^−1^. This indicates that PyNOs can form weaker XB with I⋯⁻O−N⁺ XB angles approaching 180° if forced by other interactions in the packing structure. For XB orientations perpendicular to the N−O bond, Δ*E*_int_ become repulsive at angles below 80° for **PfIB** orientations out of the plane of PyNO and much sooner for orientations in the plane of PyNO as the iodine of **PfIB** starts to collide with the ortho atoms of PyNO.

The attractive overlap of the σ‐hole with the *N*‐oxide oxygen electron density decreases with increasing distance between iodine and oxygen (Figure S149, Supporting Information). The potential curves as a function of I···O distance for **PfIB**‐PyNO and **pDIB**‐PyNO complexes with similar σ‐hole strengths (**PfIB**, 132 kJ mol^−1^; **pDIB**, 134 kJ mol^−1^), overlap and reach the minimum ≈2.65 Å. For the **oDIB**‐PyNO complex with a weaker σ‐hole strength (125 kJ mol^−1^), the distance versus energy profile reaches the minimum ≈2.80 Å. At I···O distances shorter than 2.28 Å, the interaction in these complexes becomes repulsive. At distances between 3.53 and 4.50 Å, all three complex types still show modest attractive interaction energies.

The XBs are highly directional because the σ‐hole electron density accepting power is located parallel to the covalent bond of the XB donor. Therefore, examination of the relationship between the deviation of the XB angle from the line of C−I bond in perfluoroiodobenzenes and interaction energy is of interest. The out‐of‐plane (Δα) and the in‐plane (Δβ) deviations of the orientations of XB donors with respect to the line of I⋯O XB to the parent pyridine *N*‐oxide are used to analyse this relationship. As shown in Figure 10c, all out‐of‐plane +Δα and –Δα deviations result in symmetric parabolic curves, i.e., energy loss or gain is the same when the deviation is +Δα or –Δα. For example, the out‐of‐plane deviation in the **PfIB**‐PyNO complex by 30° roughly halves the Δ*E*_int_ compared to the energy in the minimum structure. The **PfIB**‐PyNO and **pDIB**‐PyNO complexes’ curves overlap due to similar σ‐hole strengths. The steeper curve of the **oDIB**‐PyNO Δ*E*_int_ compared to the others despite the lower σ‐hole strength of **oDIB** compared to other donors is due to the interaction of the second iodine atom with the π‐cloud of the PyNO that cannot be separated from the XB interaction. For reference, Δ*E*_int_ calculated for **oDIB**‐PyNO stationary point structure where the second iodine points away from the PyNO ring (−31.2 kJ mol^−1^) is much more in line with the relative XB donor strength of the **oDIB** expected from the σ‐hole strength. The rapid decrease in Δ*E*_int_ upon in‐plane ‐Δβ deviation of **oDIB** in the **oDIB**‐PyNO complex is related to the clash of the second iodine with the PyNO ring. In +Δβ deviation, the change in energy numbers is much slower.

By co‐plotting Δ*E*_int_ as a function of both I⋯O XB distances and σ‐hole angles a more general view of the limits of favorable structural parameters can be achieved. An example plot using the data of the **PfIB**‐PyNO model system is presented in Figure 10d. The most attractive interactions fall into the red‐hot zone with I⋯O interactions at ≈2.50–3.30 Å and ∠C−I⋯O angles at 160–180°. The energies at this hot zone are stronger than −25 kJ mol^−1^. By limiting the I⋯O distance closer to the optimal distance the ∠C−I⋯O angle can be bent further to 150° while retaining the strong attractive Δ*E*_int_. For I⋯O interactions close to or just above the sum of van der Waals radii (3.65–3.75 Å) the interaction energies are modest ≈−15 kJ mol^−1^, but ∠C−I⋯O angle can be bent similarly to ≈150° without significant loss of energy. Any longer or more bent XB interactions lead to poorer Δ*E*_int_ (green and cyan regions) and these interactions are not expected to effectively stabilize structures.

**Figure 11:**
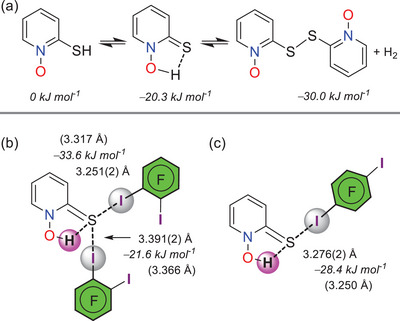
2‐Mercaptopyridine *N*‐oxide's (**18**) a) tautomeric forms and dimerization structures with relative free energies of formation in CHCl_3_. Non‐*N*‐oxide based I⋯S halogen bonding interactions in b) **oDIB**‐**18** and c) **pDIB**‐**18** All energies have been calculated at PBE0‐D3/def2‐TZVP level of theory. ΔE_int_ values are given in italics and optimized structures’ XB distances are in parentheses.

Two aspects influence or alter the *N*‐oxide's C−I···**⁻**O−N^+^ XB modes: the ‐SH substituent and the hydrogen bonding caused by water molecules. With regard to the first aspect, 2‐mercaptopyridine *N*‐oxide (**18**) containing a *ortho*‐SH group exists as a thione form rather than a thiol and it spontaneously dimerizes to generate the *N*,*N*‐dioxide disulfide derivative. The observed stability of the thione form is supported by the relative free energies of formation calculated in CHCl_3_ and shown in **Figure** **11**a. The *N*,*N*‐dioxide disulfide dimer formation is not energetically favorable but S−S bond formation affinity of sulfur compounds and the escape of formed hydrogen appears to provide enough driving force for the reaction. Five XB complexes of **18** have been determined, and the *N*‐oxide ligand is in thione form in three complexes (**oDIB**‐**18, pDIB**‐**18**, **trIB**‐**18**) and *N,N*‐dioxide disulfide form in the other two complexes (**oDIB**‐**18a**, **pDIB**‐**18a**). The *N*‐oxide oxygens in thione complexes are I⋯O passive and exhibit an intramolecular N−O−H⋯S hydrogen bond between the protonated *N*‐oxide oxygen and sulfur (Figure 11a). The asymmetric unit of **oDIB**‐**18** consists of two crystallographically independent donors and two thiones, one of which accepts one XB and the other that forms a bidentate XB. In **pDIB**‐**18**, thione sulfur is a single XB acceptor, whereas in **trIB**‐**18**, it accepts a bidentate XB. The overall I⋯S distances range from 3.163(2) to 3.391(2) Å, and the simple I⋯S XBs (3.163(2) – 3.208(2) Å) are shorter than bidentate I⋯S XBs (3.229(2) – 3.391(2)Å). The energy of the simple I⋯S in **pDIB**‐**18** is –28.4 kJ mol^−1^, while the shorter and longer bidentate I⋯S XBs in **oDIB**‐**18** are –33.6 and –21.6 kJ mol^−1^, respectively (Figure 11b‐c). The *N,N*‐dioxide disulfide forms of **oDIB**‐**18a** and **pDIB**‐**18a** exhibit I⋯O contacts, and their sulfur atoms do not show any I⋯S short contacts (Figure S148, Supporting Information).

**Figure 12:**
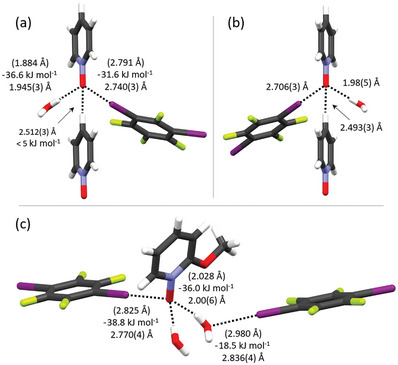
Water co‐crystallized halogen‐bonded complexes of a) **mDIB**‐**1**, b) **pDIB**‐**1**, and c) **pDIB**‐**12**. Notes: i) The XB and HB interaction energies of **pDIB**‐**1** are similar to those of **mDIB**‐**1**. ii)Two water‐based hydrogen bonds in **pDIB**‐**12** are symmetrically equivalent.

In terms of the second aspect, a water molecule co‐crystallizes with XB donor and acceptor in five complexes (**mDIB**‐**1**, **mDIB**‐**2**, **mDIB**‐**7**, **pDIB**‐**1**, and **pDIB**‐**12**), rendering *N*‐oxide oxygens a hybrid HB‐XB mode (**Figure** **12**; Figure S144, Supporting Information; for HB‐XB polymer structures, See Figures S145 and S146, Supporting Information). The *N*‐oxide oxygen in **mDIB**‐**1**, **mDIB**‐**2**, **mDIB**‐**7**, and **pDIB**‐**1** exhibits a µ_3_‐*O*,*O*,*O* mode with three different interaction types; one C−I···**⁻**O−N^+^ XB, one (water)O−H···**⁻**O−N^+^ HB, and one C−H···**⁻**O−N^+^ HB mode. In contrast, the *N*‐oxide oxygen in **pDIB**‐**12** displays a µ_3_‐*O*,*O*,*O* mode with one C−I···**⁻**O−N^+^ XB and two (water)O−H···**⁻**O−N^+^ HBs. Remarkably, **pDIB**‐**12** exhibits I···O XBs between the oxygen of water and iodine of **pDIB** at a distance of 2.836(4) Å. To the best of our knowledge, a water halogen‐bonded complex has never been crystallised before. In general, water molecules do not compete with XB donors, but rather share an *N*‐oxide oxygen acceptor. DFT energies are calculated and compared in **mDIB**‐**1** and **pDIB**‐**7** to access whether XBs are energetically different from their shared HBs. As shown in Figure 12a,c, the C−I···**⁻**O−N^+^ XB energies are comparable to (water)O−H···**⁻**O−N^+^ HBs. The tetrahedral arrangement of lone pairs on oxygen in part is responsible for providing access to equal amounts of electron density for HB and XB interactions with comparable energies. The energies of C−H···**⁻**O−N^+^ HB could not be estimated due to their weak nature. The energy of C−I···O(water) XB is weaker than C−I···**⁻**O−N^+^ and O−H···**⁻**O−N^+^ interactions, which explains why the weaker perfluoroiodoarene‐water XB complexes do not crystallize in all complexes.

Motivated by these XB‐HB structures, a 1:1 ratio acetone:H_2_O (1 mL) mixture was used to crystallize the 1:1 eq. donor:acceptor of **oDIB**‐**2**, **mDIB**‐**2**, and **pDIB**‐**2** in order to incorporate water into halogen‐bonded structures. Unfortunately, their crystal structures do not contain water molecules and are identical to those crystallized from pure acetone. Surprisingly, when just PyNOs crystallized from a 1:1 ratio acetone:H_2_O (1 mL) mixture, some PyNOs crystallized without water and some with water molecules (Figures S114–S129, Supporting Information). This implies that water's presence in these halogen‐bonded structures is a result of serendipitous coincidence. Note that XB complexes of **4**, **14,** and **31** do not contain water molecules, despite having been purchased commercially as hydrates.

The ^15^N NMR coordination shift has been used to measure the coordination strength in solution for halogen‐bonded systems.^[^ ^41^ ^]^ For C−I···⁻O−N^+^ XBs in this study, we were unable to determine non‐zero coordination shifts or association constants in XB non‐competitive CDCl_3_ (For spectra, See Figures S152–S154, Supporting Information). Raman spectroscopy has been previously employed to investigate the C−I vibrational modes of the XB donor of halogen‐bonded complexes relative to their uncomplexed XB donors.^[^ ^42^ ^]^ The typical uncomplexed C−I bond signal is located between 100 and 400 cm^−1^, and it red shifts upon XB complexation. Raman spectrum was acquired for the shortest C−I···⁻O−N^+^ XBs in each series (Table 2, Figures S155–S164, Supporting Information). **PfIB**‐**20**, **oDIB**‐**20**, **mDIB**‐**8**, **pDIB**‐**9**, and **trIB**‐**13** were found to have redshifts of 7, 5, 9, 10, and 4 cm^−1^, in that order. A comparable magnitude of redshift has been observed for C−I···N_Py_ complexes in the literature.^[^ ^42^ ^]^ Nevertheless, as compared to uncomplexed donors, the XB complexes clearly show the redshifts, suggesting a weaker C−I bond force constant and n→σ* characteristic of the I···O interaction.

## 3 Conclusion

The nature of C−I···⁻O−N^+^ halogen bonds (XB) formed by five different perfluoroiodoarenes and thirty‐two pyridine *N*‐oxides (PyNOs) is examined via experimental and theoretical studies. A detailed analysis of these XBs revealed consistent/uniform structural and geometrical behavior from the XB donor and acceptor viewpoints, observation of which would not have been possible from a small data set. The *N*‐oxide oxygen rarely displays a single C−I···⁻O−N^+^ halogen bond due to its propensity for polydentate interactions; instead, they prefer C−H···⁻O−N^+^ and C−I···⁻O−N^+^ mixed modes. Note that these perfluoroiodoarene‐PyNO complexes form either discrete or consistent polymeric structures, despite the *N*‐oxide's preference for polydentate interactions and the potential of perfluoroiodoarenes to generate complex structures via, e.g., F···F and *π*–*π* interactions. The energy of C−H···⁻O−N^+^ HBs is small (<5 kJ mol^−1^), yet these HBs are shown to be essential for the stabilization of the crystal lattices. Their role should not be undermined when studying XBs between perfluoroiodoarenes and PyNOs.

The O‐atom of *N*‐oxides are excellent electron donors for XB complex formation; nevertheless, they cannot form strong XBs unless the XB donor, such as those in N‐haloimides, have large enough σ‐holes. Thus, in these C−I···⁻O−N^+^ halogen‐bonded complexes, the σ‐hole of perfluoroiodobenzenes becomes the most essential component in defining the XB strength/distance and directionality. Due to the weak electron‐accepting properties of their iodine atoms, they exhibit a broad range of I···O XB contacts ranging from 2.648(2) to 3.252(4) Å. The C−I···O XB angles also vary quite a lot, being between 148° and 180°. The I···⁻O−N^+^ XB angles range from 87° to 152°. The angle analysis shows that, from the XB donor perspective, the I···O XBs are more directional and are in full accordance with the σ‐hole concept; but from PyNOs point of view, their directionality is substantially more versatile due to the *N*‐oxide's aptitude for polydentate interactions. The broad range of XB directionality for PyNOs was not observed with N‐haloimide N−I···⁻O−N^+^ halogen‐bonded complexes due to the large σ‐holes of N‐haloimides. This led to a conclusion that weaker C−I···⁻O─N^+^ interactions give perfluoroiodoarenes more access to the electron‐rich regions of *N*‐oxide oxygen. Density Functional Theory (DFT) spherical analysis indicates that I···O interactions are only repulsive for I···⁻O─N^+^ angles below ≈80°. This suggests that all XBs observed in crystal structures with I···⁻O─N^+^ XB angles varying between 87° and 152° are attractive. The range's minimum is almost perpendicular to the PyNO backbone, while its maximum is close to the C─I···O XB angle.

The similar relative strengths of (water)O─H···⁻O─N^+^ hydrogen bonds (HBs) and C─I···⁻O─N^+^ XBs shown by DFT analysis can lead to a situation where HBs and XBs share a common *N*‐oxide oxygen acceptor rather than displace each other. The tetrahedral configuration of a lone‐pair of electrons on the *N*‐oxide oxygen enables this partnership. By comparison in pyridine XB acceptor systems, where pyridinic nitrogen has one lone pair of electrons, such HB‐XB co‐existence is not feasible. However, building these highly desirable HB‐XB hybrid structures is not an easy task as even in the case of PyNO systems any attempts to deliberately introduce water to structures failed.

## 4 Experimental Section

### Crystallography Data

Deposition Numbers 2336839–2336848 (for **PfIB**‐PyNO series), 2337233–2337264, 2337807 (for **oDIB**‐PyNO series), 2337501–2337518 (for **mDIB**‐PyNO series), 2337658–2337685 (for **pDIB**‐PyNO series), 2337690–2337712 (for **trIB**‐PyNO series), and 2337775–2337790 (for PyNOs crystallized from water), contain the supplementary crystallography data for this paper. These data were provided free of charge by the joint Cambridge Crystallographic Data Centre and Fachinformationszentrum Karlsruhe Access Structures service.

### Statistical Analysis

X‐ray crystallography data was processed using CrysAlis^Pro^.^[^ ^43^ ^]^ ChemDraw^[^ ^44^ ^]^ and Mercury^[^ ^45^ ^]^ softwares were used to prepare the crystal structure graphics in manuscript and Supporting Information, Topspin^[^ ^46^ ^]^ for ^15^N NMR spectra, MestReNova^[^ ^47^ ^]^ for ^1^H NMR spectra and OMNIC^[^ ^48^ ^]^ for Raman spectra in the Supporting Information. Olex2^[^ ^49^ ^]^ was used to extract bond parameters of 128 X‐ray crystal structures. Microsoft Excel was used for the correlation analysis of Figures 7 and 8, whereas OriginPro 2017^[^ ^50^ ^]^ program for Figure 9. The mean values of N−O bond distances were 1.324 ± 0.001, C−I 2.097 ± 0.013, I⋯O 2.798 ± 0.001 Å, C⋯O 4.884 ± 0.006, Å, N−O⋯I angles 116.91 ± 0.057°, and C−I⋯O angles 173.44±0.015°. There were 1446 energy values in Figure 10a,b (gas‐phase models) and 1260 energy values in Figure 10d. Figure 10a,b were prepared using the ParaView 5.11.2^[^ ^51^ ^]^ software and Figure 10c,d using the OriginPro 2017.

The authors have cited additional references within the Supporting Information.^[^ ^49^, ^52^, ^53^, ^54^, ^55^, ^56^, ^57^, ^58^, ^59^ ^]^

## Conflict of Interest

The authors declare no conflict of interest.

## Supporting information

Supporting Information

## Data Availability

The data that support the findings of this study are available from the corresponding author upon reasonable request.
