# Supplementary material for: The Geometry and Nature of C─I···O─N Interactions in Perfluoroiodobenzene‐Pyridine N‐oxide Halogen‐Bonded Complexes
Source: Adv Sci (Weinh). 2024 Jun 13;11(30):2403945. doi: 10.1002/advs.202403945 (PMC11321645; doi:10.1002/advs.202403945)
Supplement: Supplementary file 1 — Supporting Information [file ADVS-11-2403945-s001.pdf]

ADVANCED SCIENCE
Open Access

Supporting Information

for *Adv. Sci.*, DOI 10.1002/advs.202403945

The Geometry and Nature of C—I···O—N Interactions in Perfluoroiodobenzene-Pyridine *N*-oxide Halogen-Bonded Complexes

Juha Mikko Rautiainen, Arto Valkonen, Jan Lundell, Kari Rissanen and Rakesh Puttreddy**

SUPPORTING INFORMATION

J. Mikko Rautiainen,[a] Arto Valkonen,[a] Jan Lundell,[a] Kari Rissanen*[a] and Rakesh Puttreddy*[a]

[a]University of Jyvaskyla, Department of Chemistry, P.O. Box. 35, FI-40014 University of Jyvaskyla, Finland

Table of Contents

1. General information ***Page 2***

2. X-ray Crystallography ***Page 3***

3. Density Functional Theory Studies ***Page 100***

4. NMR and Raman Studies ***Page 102***

5. References ***Page 109***

1. General information

1.1 Starting materials and solvents

Solvents used for synthesis and crystallization experiments were HPLC grade and used as received. Pyridine N-oxide (95%), 2-methylpyridine N-oxide (>96%), 3-methylpyridine N-oxide (98%), 4-methylpyridine N-oxide (98%), 4-methoxypyridine N-oxide (97%), 4-phenylpyridine N-oxide (98%), 2-mercaptopyridine N-oxide (99%), quinoline N-oxide (97%), isoquinoline N-oxide (98%) were purchased from Sigma Aldrich whereas the other N-oxides were synthesized as reported.[1] Pentafluoroiodobenzene (99%, **PfIB**) was purchased from Aldrich, 1,2-diiodotetrafluorobenzene (97%, **oDIB**), 1,3-diiodotetrafluorobenzene (97%, **mDIB**) and 1,4-diiodotetrafluorobenzene (97%, **pDIB**) were purchased from Apollo Scientifics, and 2,4,6-triiodotrifluorobenzene (95%, **trIB**) from Fluorochem.

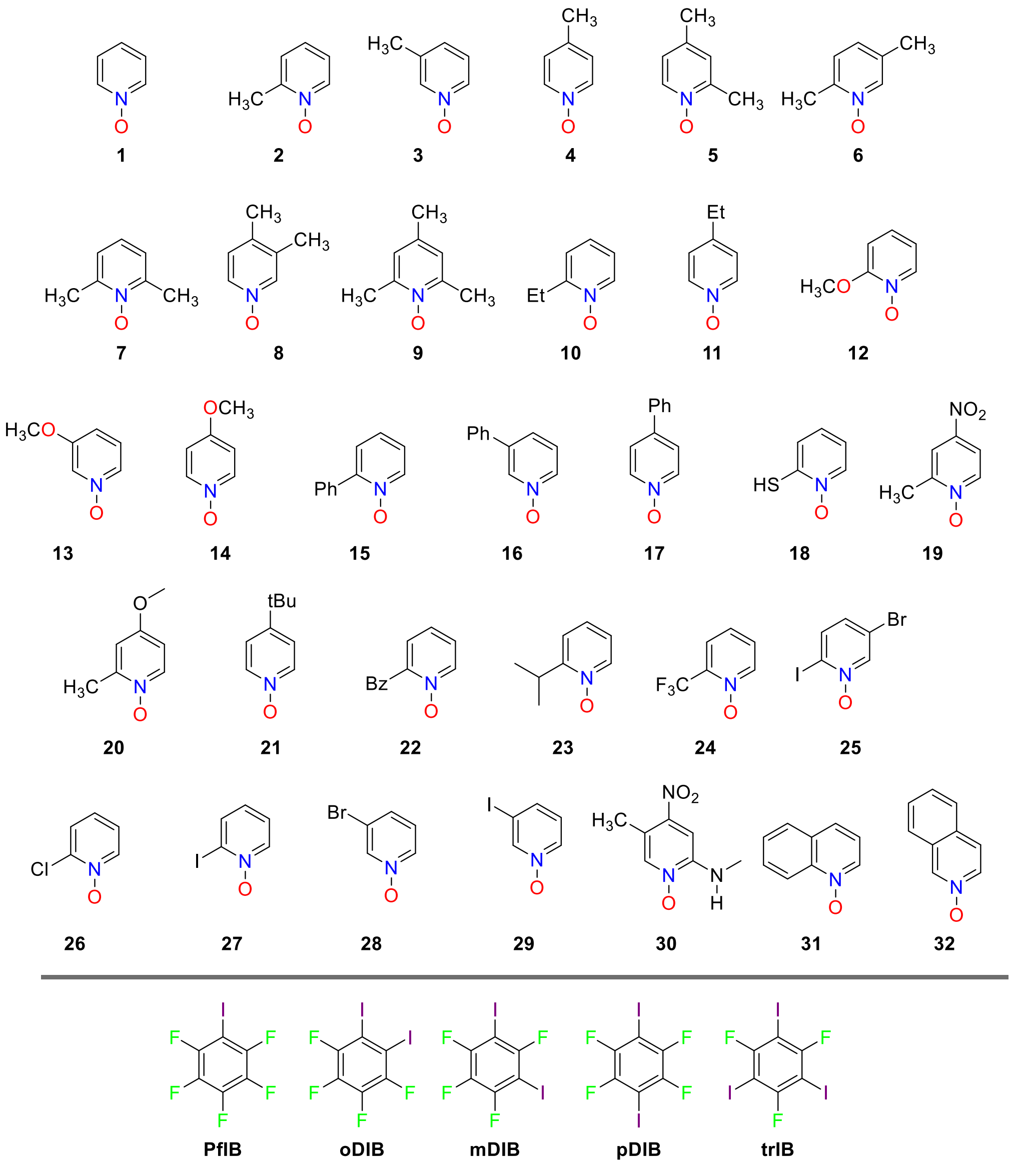

Figure S1. List of pyridine N-oxides (**1** - **32**) as XB acceptors and pentafluoroiodobenzene (**PfIB**), 1,2-diiodotetrafluorobenzene (**oDIB**), 1,3-diiodotetrafluorobenzene (**mDIB**), 1,4-diiodotetrafluorobenzene (**pDIB**), and 2,4,6-triiodotrifluorobenzene (**trIB**) as XB donors.

2. X-ray Crystallography

2.1 X-Ray Experimental details

Data collected on Bruker-Nonius Kappa CCD diffractometer with an APEX-II detector and graphite monochromatized Mo-Kα (λ = 0.71073 Å) radiation: **oDIB-4, oDIB-4a, oDIB-12, oDIB-12a, oDIB-13, oDIB-14, oDIB-17, oDIB-23, oDIB-28, oDIB-31, pDIB-2, pDIB-3, pDIB-4, pDIB-6, pDIB-12, pDIB-13, pDIB-14, pDIB-15, pDIB-27, 4, 9-H_2O, 9a-H_2O, 12-H_2O, 14-H_2O, 16, 18, 18a, 19,** and **32.**

Data collected on Rigaku SuperNova single-source diffractometer with an Atlas EoS-CCD detector using mirror-monochromated Mo-*K*α (λ = 0.71073 Å) radiation: **oDIB-1, oDIB-6, oDIB-7, oDIB-9, oDIB-10, oDIB-11, oDIB-12b, oDIB-15a, oDIB-18a, oDIB-19, oDIB-24, oDIB-25, oDIB-27, pDIB-1, pDIB-7, pDIB-9, pDIB-10, pDIB-11, pDIB-16, pDIB-18a, pDIB-19, pDIB-21, pDIB-23, pDIB-24, pDIB-29, pDIB-31, trIB-1, trIB-2,** and **30.**

Data collected using an Rigaku SuperNova four-circle diffractometer equipped with a Hybrid Pixel Array Detector (detector type: HyPix-Arc 100) and Cu-K_α (λ = 1.54184 Å) radiation: **PfIB-13, oDIB-2, oDIB-3, oDIB-20, oDIB-21, oDIB-31, mDIB-1, mDIB-6, mDIB-11, pDIB-8, pDIB-17, pDIB-18, pDIB-26, trIB-3, trIB-6, trIB-11, trIB-13, trIB-15, trIB-18, trIB-22, 16-H_2O, 15, 17-H_2O, 20-H_2O,** and **22.**

The data collected using an XtaLAB Synergy R four-circle diffractometer equipped with a Hybrid Pixel Array Detector (detector type: HyPix-Arc 100; Diffraction source type is PhotonJet R (Cu, λ = 1.54184 Å) X-ray source): **mDIB, PfIB-3, PfIB-8, PfIB-14, PfIB-15, PfIB-16, PfIB-17, PfIB-20, PfIB-31, PfIB-32, oDIB-5, oDIB-8, oDIB-15, oDIB-18, oDIB-26, mDIB-2, mDIB-3, mDIB-4, mDIB-5, mDIB-7, mDIB-8, mDIB-9, mDIB-15, mDIB-19, mDIB-21, mDIB-22, mDIB-29, mDIB-31, mDIB-32, pDIB-5, pDIB-28, trIB-4, trIB-5, trIB-7, trIB-8, trIB-9, trIB-12, trIB-16, trIB-17, trIB-20, trIB-21, trIB-28, trIB-29, trIB-30,** and **trIB-32.**

For Rigaku SuperNova diffractometer and XtaLAB Synergy R four-circle diffractometer datasets, CrysAlisPro (version 1.171.43.100a) was used for the data collection and reduction, and the intensities were absorption corrected using a gaussian/analytical face index absorption correction method. For the data obtained from Bruker Nonius Kappa diffractometer were performed using the program COLLECT and HKL DENZO AND SCALEPACK,[2] and the intensities were corrected for absorption using 'sadabs' with multi-scan absorption correction type method. All structures were solved by intrinsic phasing (SHELXT)[3] and refined by full-matrix least squares on F^2 using the OLEX2,[4] utilizing the SHELXL-2015 module.[5,6] Anisotropic displacement parameters were assigned to non-H atoms and isotropic displacement parameters for all H atoms were constrained to multiples of the equivalent displacement parameters of their parent atoms with $U_{iso}(H) = 1.2\ U_{eq}$(parent atom). The X-ray single crystal data and CCDC numbers of all new structures are included below. These data can be obtained free of charge via http://www.ccdc.cam.ac.uk/conts/retrieving.html (or from the CCDC, 12 Union Road, Cambridge CB2 1EZ, UK; Fax: +44 1223 336033; E-mail: deposit@ccdc.cam.ac.uk).

2.2 General procedure for crystallizations

The pyridine N-oxide (0.1 mmol, 1.0 eq) was added to the perfluoroiodoarene (0.1 mmol, 1.0 eq) diluted in 1.0 - 1.2 mL chloroform. The mixture was briefly stirred with a vortex at room temperature to dissolve the components, and the solutions were allowed to slowly evaporate to produce single crystals suitable for X-ray crystallography. The following complexes were obtained by this method: **PfIB-8, oDIB-1, oDIB-2, oDIB-3, oDIB-4, oDIB-4a, oDIB-5, oDIB-6, oDIB-7, oDIB-8, oDIB-9, oDIB-10, oDIB-11, oDIB-12, oDIB-12a, oDIB-12b, oDIB-13, oDIB-14, oDIB-15, oDIB-15a, oDIB-17, oDIB-18, oDIB-18a, oDIB-19, oDIB-20, oDIB-21, oDIB-23, oDIB-24, oDIB-25, oDIB-27, oDIB-28, oDIB-31, oDIB-32, mDIB-5, pDIB-1, pDIB-2, pDIB-3, pDIB-4, pDIB-5, pDIB-6, pDIB-7, pDIB-8, pDIB-9, pDIB-10, pDIB-11, pDIB-12, pDIB-13, pDIB-14, pDIB-15, pDIB-16, pDIB-17, pDIB-18, pDIB-18a, pDIB-19, pDIB-21, pDIB-23, pDIB-24, pDIB-27, pDIB-29, pDIB-31, trIB-1, trIB-3, trIB-4, trIB-5, trIB-6, trIB-7, trIB-8, trIB-9, trIB-11, trIB-13, trIB-15, trIB-18, trIB-29, trIB-30,** and **trIB-32.**

The combinations that did not produce single crystals were redissolved in acetone (1.0 – 1.2 mL) and stirred until the mixture is completely dissolved at room temperature. The solutions were kept at room temperature to produce crystals for X-ray diffraction analysis. With this method, the following complexes were prepared: **PfIB-16** and **pDIB-26**.

The combinations that failed to yield single crystals were remade by mixing pyridine N-oxide (0.1 mmol, 1.0 eq) and perfluoroiodoarene (0.1 mmol, 1.0 eq) in 1.0 - 1.2 mL of acetone. The components were dissolved with the help vortex stir at room temperature. The solutions were then allowed to slowly evaporate, yielding single crystals that were appropriate for X-ray crystallography. Using this technique, the following complexes were produced:

PfIB-16, mDIB-1, mDIB-2, mDIB-3, mDIB-4, mDIB-6, mDIB-7, mDIB-9, mDIB-32, trIB-12, trIB-16, and **trIB-17.**

The donor:acceptor mixtures that did not yield single crystals from acetone were redissolved in dichloromethane (1.0 - 1.2 mL) and stirred until completely dissolved at room temperature. The solutions were left at room temperature to form crystals for X-ray diffraction analysis. The following complexes were made using this technique: **PfIB-3, PfIB-13, PfIB-14, PfIB-15, PfIB-17, PfIB-32, oDIB-26,** and **trIB-2.**

For the halogen bond complexes that did not form single crystals by a simple mixing procedure, a grinding method was undertaken. Using a pestle and mortar, pyridine N-oxide (0.1 mmol, 1.0 eq) and perfluoroiodoarene (0.1 mmol, 1.0 eq) were grinded for approximately a minute before being dissolved in chloroform (1.0 – 1.2 mL). The solutions were kept at room temperature to form crystals suitable for X-ray diffraction. Using this procedure, the following complexes were formed: **PfIB-20, PfIB-31, mDIB-8, mDIB-11, mDIB-15, mDIB-19, mDIB-21, mDIB-22, mDIB-29, mDIB-31, pDIB-28, trIB-21, trIB-22, trIB-20,** and **trIB-28.**

To obtain single crystals of N-oxides from water, approximately 5-6mg of N-oxide was dissolved in 1:1 acetone:water (1 mL) and briefly stirred with a vortex. The solutions were left at room temperature to slowly evaporate, resulting in crystals for X-ray diffraction analysis.

2.3. PfIB-PyNO halogen-bonded complexes

1. Complex PfIB-3

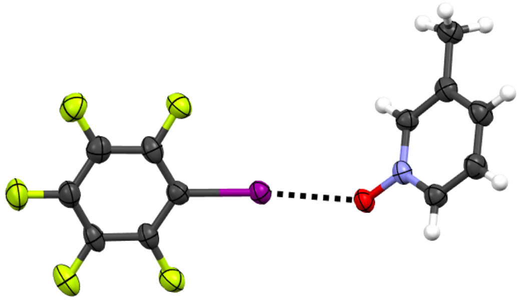

Figure S2. The X-ray crystal structure of **PfIB-3** with the thermal displacement parameter at 50% probability level. The black broken line represents the halogen bond. Colour Key: green = fluorine, purple = iodine, blue = nitrogen, dark grey = carbon, white = hydrogen. **Notes**: The asymmetric unit contains one **PfIB** and one PyNO molecule. Disordered **PfIB** part is omitted for viewing clarity.

Crystal data for PfIB-3 (crystallization solvent: CH_2Cl_2): CCDC-2336839, $C_{12}H_7F_5INO$, M = 403.09 gmol^{-1}, colourless block, 0.116 × 0.076 × 0.054 mm, triclinic, space group *P-1*, a = 6.5317(2) Å, b = 7.9457(3) Å, c = 13.0055(4) Å, α = 97.731(3)°, β = 90.147(3)°, γ = 106.781(3)°, V = 639.74(4) Å^3, Z = 2, D_{calc} = 2.093 gcm^{-3}, F(000) = 384, μ = 20.243 mm^{-1}, T = 120(1) K, θ_{max} = 66.735°, 7241 total reflections, 2156 with $I_o > 2\sigma(I_o)$, R_{int} = 0.0350, 2260 data, 254 parameters, 0 restraints, GooF = 1.055, R = 0.0237 and wR = 0.0621 [$I_o > 2\sigma(I_o)$], R = 0.0247 and wR = 0.0626 (all reflections), 0.613 <dΔρ < -0.877 eÅ^{-3}.

2. Complex PfIB-8

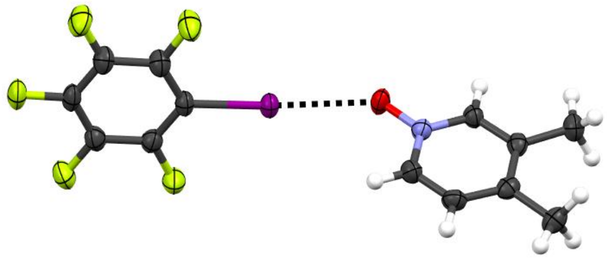

Figure S3. The X-ray crystal structure of **PfIB-8** with the thermal displacement parameter at 50% probability level. The black broken line represents the halogen bond. Colour Key: green = fluorine, purple = iodine, blue = nitrogen, dark grey = carbon, white = hydrogen. **Notes**: The asymmetric unit consists of five **PfIB** molecules and three PyNO molecules, one of which is disordered over two positions in a 50:50 ratio. For viewing clarity, just the donor:acceptor ratio of 1:1 is shown.

Crystal data for PfIB-8 (crystallization solvent: $CHCl_3$): CCDC-2336840, $C_{102}H_{54}F_{50}I_{10}N_6O_6$, M = 3678.51 gmol^{-1}, colourless block, 0.19 × 0.11 × 0.1 mm, triclinic, space group *P-1*, a = 14.2949(2) Å, b = 14.4978(3) Å, c = 15.5296(2) Å, α = 110.457(2)°, β = 92.0220(10)°, γ = 107.107(2)°, V = 2847.57(9) Å^3, Z = 2, D_{calc} = 2.145 gcm^{-3}, F(000) = 1736, μ = 22.615 mm^{-1}, T = 120(1) K, θ_{max} = 66.745°, 41143 total reflections, 9266 with $I_o > 2\sigma(I_o)$, R_{int} = 0.0347, 10084 data, 867 parameters, 0 restraints, GooF = 1.029, R = 0.0262 and wR = 0.0662 [$I_o > 2\sigma(I_o)$], R = 0.0288 and wR = 0.0676 (all reflections), 0.704 <dΔρ < -0.792 eÅ^{-3}.

3. Complex PfIB-13

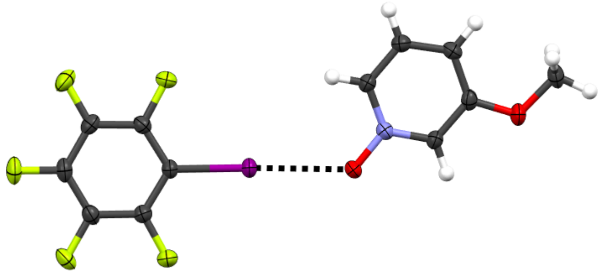

Figure S4. The X-ray crystal structure of **PfIB-13** with the thermal displacement parameter at 50% probability level. The black broken line represents the halogen bond. Colour Key: green = fluorine, purple = iodine, blue = nitrogen, dark grey = carbon, white = hydrogen. **Notes**: The asymmetric unit contains one **PfIB** and one PyNO molecule.

Crystal data for PfIB-13 (crystallization solvent: CH_2Cl_2): CCDC-2336841, $C_{12}H_7F_5INO_2$, M = 419.09 gmol^{-1}, colourless block, 0.08 × 0.05 × 0.04 mm, triclinic, space group *P-1*, a = 6.06891(18) Å, b = 9.9313(3) Å, c = 11.3839(3) Å, α = 86.005(2)°, β = 77.224(2)°, γ = 81.982(3)°, V = 662.07(3) Å^3, Z = 2, D_{calc} = 2.102 gcm^{-3}, F(000) = 400, μ = 19.652 mm^{-1}, T = 120(1) K, θ_{max} = 66.694°, 7452 total reflections, 2231 with $I_o > 2\sigma(I_o)$, R_{int} = 0.0354, 2335 data, 191 parameters, 0 restraints, GooF = 1.111, R = 0.0238 and wR = 0.0600 [$I_o > 2\sigma(I_o)$], R = 0.0256 and wR = 0.0611 (all reflections), 0.754 <dΔρ < -0.861 eÅ^{-3}.

4. Complex PfIB-14

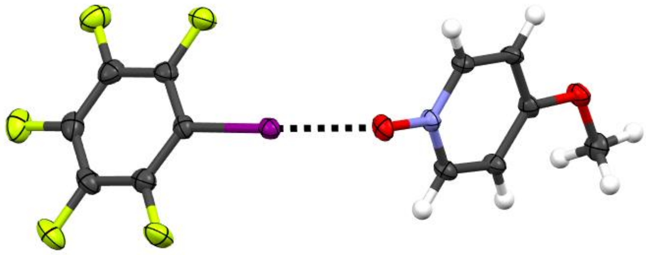

Figure S5. The X-ray crystal structure of **PfIB-14** with the thermal displacement parameter at 50% probability level. The black broken line represents the halogen bond. Colour Key: green = fluorine, purple = iodine, blue = nitrogen, dark grey = carbon, white = hydrogen. **Notes**: The asymmetric unit contains one **PfIB** and one PyNO molecule.

Crystal data for PfIB-14 (crystallization solvent: CH_2Cl_2): CCDC-2336842, $C_{12}H_7F_5INO_2$, M = 419.09 gmol^{-1}, colourless block, 0.157 × 0.104 × 0.042 mm, monoclinic, space group *P2₁/c*, a = 14.4702(3) Å, b = 6.47890(10) Å, c = 15.7426(3) Å, α = 90°, β = 114.544(2)°, γ = 90°, V = 1342.53(5) Å^3, Z = 4, D_{calc} = 2.073 gcm^{-3}, F(000) = 800, μ = 19.383 mm^{-1}, T = 120(1) K, θ_{max} = 66.729°, 9262 total reflections, 2206 with $I_o > 2\sigma(I_o)$, R_{int} = 0.0413, 2377 data, 191 parameters, 0 restraints, GooF = 1.023, R = 0.0237 and wR = 0.0642 [$I_o > 2\sigma(I_o)$], R = 0.0272 and wR = 0.0656 (all reflections), 0.532 <dΔρ < -0.572 eÅ^{-3}.

5. Complex PfIB-15

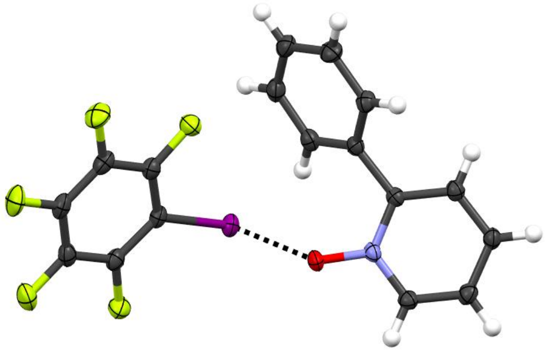

Figure S6. The X-ray crystal structure of **PfIB-15** with the thermal displacement parameter at 50% probability level. The black broken line represents the halogen bond. Colour Key: green = fluorine, purple = iodine, blue = nitrogen, dark grey = carbon, white = hydrogen. **Notes**: The asymmetric unit contains one **PfIB** and one PyNO molecule.

Crystal data for PfIB-15 (crystallization solvent: CH_2Cl_2): CCDC-2336843, $C_{17}H_9F_5INO$, M = 465.15 gmol^{-1}, colourless block, 0.07 × 0.03 × 0.03 mm, triclinic, space group *P-1*, a = 6.00730(10) Å, b = 8.44180(10) Å, c = 16.1606(2) Å, α = 97.3750(10)°, β = 90.8160(10)°, γ = 102.815(2)°, V = 791.74(2) Å^3, Z = 2, D_{calc} = 1.951 gcm^{-3}, F(000) = 448, μ = 16.470 mm^{-1}, T = 120(1) K, θ_{max} = 79.188°, 12443 total reflections, 3263 with $I_o > 2\sigma(I_o)$, R_{int} = 0.0358, 3411 data, 226 parameters, 0 restraints, GooF = 1.072, R = 0.0226 and wR = 0.0543 [$I_o > 2\sigma(I_o)$], R = 0.0238 and wR = 0.0549 (all reflections), 0.500 <dΔρ < -0.609 eÅ^{-3}.

6. Complex PfIB-16

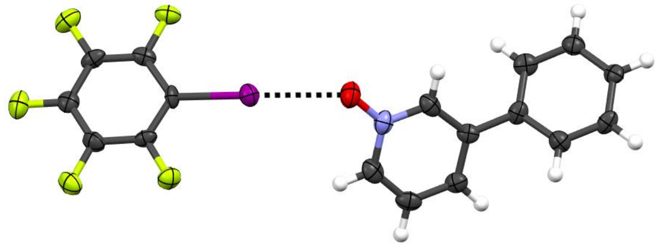

Figure S7. The X-ray crystal structure of **PfIB-16** with the thermal displacement parameter at 50% probability level. The black broken line represents the halogen bond. Colour Key: green = fluorine, purple = iodine, blue = nitrogen, dark grey = carbon, white = hydrogen. **Notes**: The asymmetric unit consists of two **PfIB** molecules and two PyNO molecules. For viewing clarity, just the 1:1 donor:acceptor ratio complex is shown.

Crystal data for PfIB-16 (crystallization solvent: acetone): CCDC-2336844, $C_{17}H_9F_5INO$, M = 465.15 gmol^{-1}, colourless block, 0.121 × 0.021 × 0.012 mm, monoclinic, space group *I2/a*, a = 28.909(3) Å, b = 6.1933(4) Å, c = 35.648(10) Å, α = 90°, β = 90.486(19)°, γ = 90°, V = 6382(2) Å^3, Z = 16, D_{calc} = 1.936 gcm^{-3}, F(000) = 3584, μ = 16.346 mm^{-1}, T = 120(1) K, θ_{max} = 66.743°, 34258 total reflections, 4272 with $I_o > 2\sigma(I_o)$, R_{int} = 0.1517, 5629 data, 451 parameters, 0 restraints, GooF = 1.041, R = 0.0719 and wR = 0.1620 [$I_o > 2\sigma(I_o)$], R = 0.0957 and wR = 0.1751 (all reflections), 3.088 <dΔρ < -2.379 eÅ^{-3}.

7. Complex PfIB-17

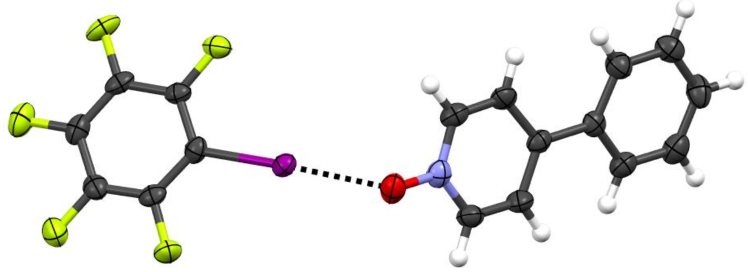

Figure S8. The X-ray crystal structure of **PfIB-17** with the thermal displacement parameter at 50% probability level. The black broken line represents the halogen bond. Colour Key: green = fluorine, purple = iodine, blue = nitrogen, dark grey = carbon, white = hydrogen. **Notes**: The asymmetric unit contains one **PfIB** and one PyNO molecule.

Crystal data for PfIB-17 (crystallization solvent: CH_2Cl_2): CCDC-2336845, $C_{17}H_9F_5INO$, M = 465.15 gmol^{-1}, colourless block, 0.161 × 0.082 × 0.024 mm, orthorhombic, space group *P2₁2₁2₁*, a = 6.22080(10) Å, b = 8.12470(10) Å, c = 31.5338(5) Å, α = 90°, β = 90°, γ = 90°, V = 1593.79(4) Å^3, Z = 4, D_{calc} = 1.939 gcm^{-3}, F(000) = 896, μ = 16.364 mm^{-1}, T = 120(1) K, θ_{max} = 66.749°, 8695 total reflections, 2670 with $I_o > 2\sigma(I_o)$, R_{int} = 0.0345, 2740 data, 226 parameters, 0 restraints, GooF = 1.145, R = 0.0321 and wR = 0.0862 [$I_o > 2\sigma(I_o)$], R = 0.0328 and wR = 0.0865 (all reflections), 1.654 <dΔρ < -0.605 eÅ^{-3}.

8. Complex PfIB-20

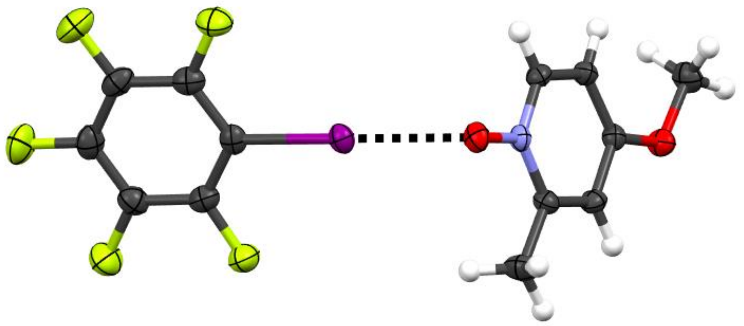

Figure S9. The X-ray crystal structure of **PfIB-20** with the thermal displacement parameter at 50% probability level. The black broken line represents the halogen bond. Colour Key: green = fluorine, purple = iodine, blue = nitrogen, dark grey = carbon, white = hydrogen. **Notes**: The asymmetric unit contains one **PfIB** and one PyNO molecule.

Crystal data for PfIB-20 (crystallization solvent: $CHCl_3$): CCDC-2336846, $C_{13}H_9F_5INO_2$, M = 433.11 gmol^{-1}, colourless block, 0.12 × 0.07 × 0.06 mm, triclinic, space group *P-1*, a = 6.4299(2) Å, b = 9.1625(3) Å, c = 12.7758(4) Å, α = 78.410(3)°, β = 82.829(3)°, γ = 84.812(3)°, V = 729.90(4) Å^3, Z = 2, D_{calc} = 1.971 gcm^{-3}, F(000) = 416, μ = 17.850 mm^{-1}, T = 120(1) K, θ_{max} = 66.713°, 10571 total reflections, 2511 with $I_o > 2\sigma(I_o)$, R_{int} = 0.0467, 2575 data, 201 parameters, 0 restraints, GooF = 1.073, R = 0.0238 and wR = 0.0601 [$I_o > 2\sigma(I_o)$], R = 0.0243 and wR = 0.0604 (all reflections), 0.634 <dΔρ < -0.702 eÅ^{-3}.

9. Complex PfIB-31

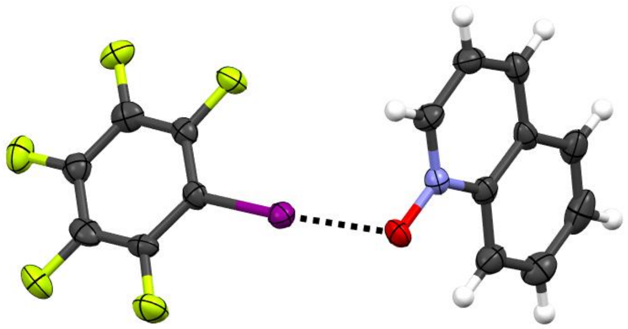

Figure S10. The X-ray crystal structure of **PfIB-31** with the thermal displacement parameter at 50% probability level. The black broken line represents the halogen bond. Colour Key: green = fluorine, purple = iodine, blue = nitrogen, dark grey = carbon, white = hydrogen. **Notes**: The asymmetric unit consists of two **PfIB** molecules and two PyNO molecules. For viewing clarity, just the 1:1 donor:acceptor ratio complex is shown.

Crystal data for PfIB-31 (crystallization solvent: $CHCl_3$): CCDC-2336847, $C_{15}H_7F_5INO$, M = 439.12 gmol^{-1}, orange block, 0.12 × 0.08 × 0.04 mm, monoclinic, space group *P2₁*, a = 7.3699(2) Å, b = 24.2899(8) Å, c = 8.0010(2) Å, α = 90°, β = 94.514(3)°, γ = 90°, V = 1427.85(7) Å^3, Z = 4, D_{calc} = 2.043 gcm^{-3}, F(000) = 840, μ = 18.215 mm^{-1}, T = 120(1) K, θ_{max} = 66.746°, 12946 total reflections, 3924 with $I_o > 2\sigma(I_o)$, R_{int} = 0.0577, 4146 data, 415 parameters, 1 restraints, GooF = 1.064, R = 0.0414 and wR = 0.1041 [$I_o > 2\sigma(I_o)$], R = 0.0434 and wR = 0.1051 (all reflections), 1.923 <dΔρ < -1.148 eÅ^{-3}.

10. Complex PfIB-32

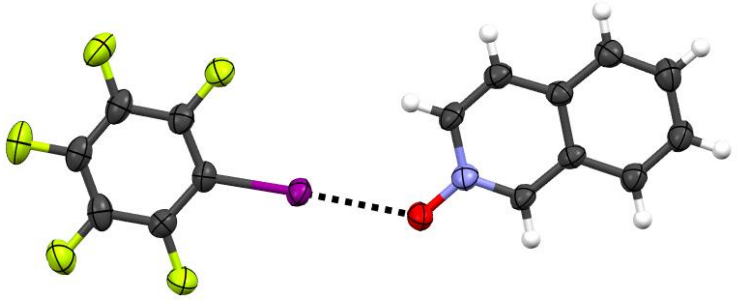

,

Figure S11. The X-ray crystal structure of **PfIB-32** with the thermal displacement parameter at 50% probability level. The black broken line represents the halogen bond. Colour Key: green = fluorine, purple = iodine, blue = nitrogen, dark grey = carbon, white = hydrogen. **Notes**: The asymmetric unit consists of one **PfIB** and one PyNO molecule.

Crystal data for PfIB-32 (crystallization solvent: CH_2Cl_2): CCDC-2336848, $C_{15}H_7F_5INO$, M = 439.12 gmol^{-1}, colourless plate, 0.149 × 0.062 × 0.034 mm, triclinic, space group *P*2/*c*, a = 6.5661(4) Å, b = 10.0693(5) Å, c = 11.4071(5) Å, α =84.970(4) °, β = 78.488(5)°, γ = 77.750(5)°, V = 721.38(7) Å^3, Z = 2, D_{calc} = 2.022 gcm^{-3}, F(000) = 420, μ = 18.026 mm^{-1}, T = 120 K, θ_{max} = 66.733°, 9190 total reflections, 2223 with $I_o > 2\sigma(I_o)$, R_{int} = 0.0680, 2534 data, 208 parameters, 0 restraints, GooF = 1.092, R = 0.0510 and wR = 0.1413 [$I_o > 2\sigma(I_o)$], R = 0.0576 and wR = 0.1447 (all reflections), 1.890 <dΔρ < -1.632 eÅ^{-3}.

2.4. oDIB-PyNO halogen-bonded complexes

1. Complex oDIB-1

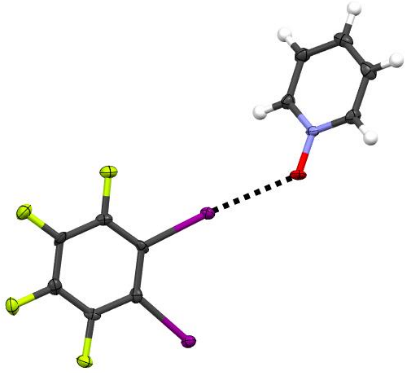

Figure S12. The X-ray crystal structure of **oDIB-1** with the thermal displacement parameter at 50% probability level. The black broken line represents the halogen bond. Colour Key: green = fluorine, purple = iodine, blue = nitrogen, dark grey = carbon, white = hydrogen. **Notes**: The asymmetric unit consists of one **oDIB** and one PyNO molecule.

Crystal data for oDIB-1 (crystallization solvent: $CHCl_3$): CCDC-2337233, $C_{11}H_5F_4I_2NO$, M = 496.96 gmol^{-1}, colourless block, 0.2132 × 0.1672 × 0.1003 mm, triclinic, space group *P-1*, a = 7.0486(5) Å, b = 8.5134(6) Å, c = 11.5208(8) Å, α = 105.501(6)°, β = 96.349(6)°, γ = 92.529(6)°, V = 660.14(8) Å^3, Z = 2, D_{calc} = 2.500 gcm^{-3}, F(000) = 456, μ = 4.801 mm^{-1}, T = 120(1) K, θ_{max} = 25.247°, 4233 total reflections, 2055 with $I_o > 2\sigma(I_o)$, R_{int} = 0.0315, 2374 data, 172 parameters, 0 restraints, GooF = 1.010, R = 0.0288 and wR = 0.0540 [$I_o > 2\sigma(I_o)$], R = 0.0347 and wR = 0.0579 (all reflections), 0.656<dΔρ < -0.657 eÅ^{-3}.

2. Complex oDIB-2

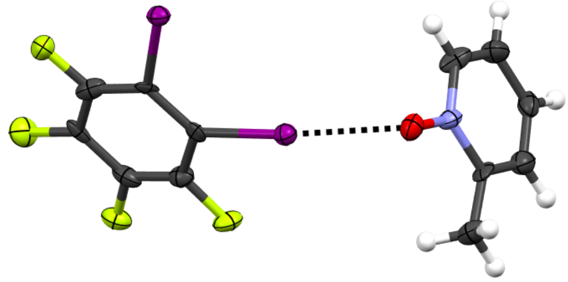

Figure S13. The X-ray crystal structure of **oDIB-2** with the thermal displacement parameter at 50% probability level. The black broken line represents the halogen bond. Colour Key: green = fluorine, purple = iodine, blue = nitrogen, dark grey = carbon, white = hydrogen. **Notes**: The asymmetric unit consists of two **oDIB** molecules and two PyNO molecules. For viewing clarity, just the 1:1 donor:acceptor ratio complex is shown.

Crystal data for oDIB-2 (crystallization solvent: $CHCl_3$ or acetone): CCDC- 2337234, $C_{12}H_7F_4I_2NO$, M = 510.99 gmol^{-1}, colourless plate, 0.17 × 0.13 × 0.07 mm, triclinic, space group *P-1*, a = 10.7146(8) Å, b = 11.4624(5) Å, c = 12.8623(8) Å, α = 84.319(5)°, β = 65.679(7)°, γ = 89.725(5)°, V = 1431.29(17) Å^3, Z = 4, D_{calc} = 2.371 gcm^{-3}, F(000) = 944, μ = 34.920 mm^{-1}, T = 123 K, θ_{max} = 66.741°, 8448 total reflections, 3696 with $I_o > 2\sigma(I_o)$, R_{int} = 0.0556, 5030 data, 357 parameters, 0 restraints, GooF = 1.030, R = 0.0579 and wR = 0.1458 [$I_o > 2\sigma(I_o)$], R = 0.0742 and wR = 0.1722 (all reflections), 2.847<dΔρ < -3.023 eÅ^{-3}.

3. Complex oDIB-3

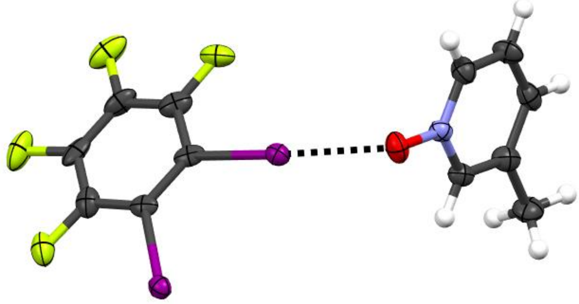

Figure S14. The X-ray crystal structure of **oDIB-3** with the thermal displacement parameter at 50% probability level. The black broken line represents the halogen bond. Colour Key: green = fluorine, purple = iodine, blue = nitrogen, dark grey = carbon, white = hydrogen. **Notes**: The asymmetric unit consists of one **oDIB** and one PyNO molecule.

Crystal data for oDIB-3 (crystallization solvent: $CHCl_3$): CCDC- 2337235, $C_{12}H_7F_4I_2NO$, M = 510.99 gmol^{-1}, colourless plate, 0.27 × 0.19 × 0.04 mm, monoclinic, space group *P2₁/c*, a = 7.90125(19)Å, b = 23.2344(5) Å, c = 7.8124(2) Å, α = 90°, β = 92.010(2)°, γ = 90°, V = 1433.33(6) Å^3, Z = 4, D_{calc} = 2.368 gcm^{-3}, F(000) = 944, μ = 34.871 mm^{-1}, T = 123 K, θ_{max} = 66.741°, 7870 total reflections, 2281 with $I_o > 2\sigma(I_o)$, R_{int} = 0.0797, 2545 data, 182 parameters, 0 restraints, GooF = 1.035, R = 0.0716 and wR = 0.1922 [$I_o > 2\sigma(I_o)$], R = 0.0753 and wR = 0.2033 (all reflections), 4.356<dΔρ < -2.708 eÅ^{-3}.

4. Complex oDIB-4

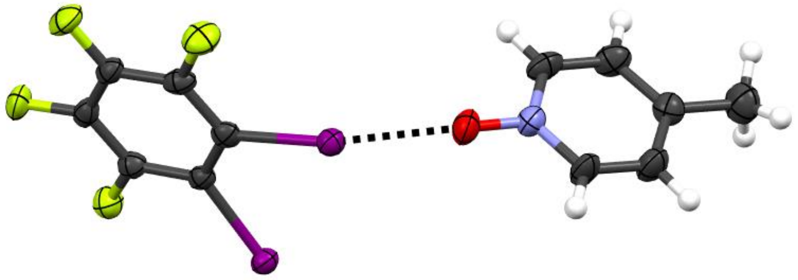

Figure S15. The X-ray crystal structure of **oDIB-4** with the thermal displacement parameter at 50% probability level. The black broken line represents the halogen bond. Colour Key: green = fluorine, purple = iodine, blue = nitrogen, dark grey = carbon, white = hydrogen. **Notes**: The asymmetric unit consists of two **oDIB** molecules and two PyNO molecules. For viewing clarity, just the 1:1 donor:acceptor ratio complex is shown.

Crystal data for oDIB-4 (crystallization solvent: $CHCl_3$): CCDC- 2337236, $C_{12}H_7F_4I_2NO$, M = 510.99 $gmol^{-1}$, colourless plate, 0.38 × 0.23 × 0.09 mm, triclinic, space group *P-1*, a = 7.6490(15) Å, b = 13.003(3) Å, c = 15.197(3) Å, α = 107.99(3)°, β = 97.50(3)°, γ = 93.82(3)°, V = 1416.1(5) $Å^3$, Z = 4, D_{calc} = 2.397 gcm^{-3}, F(000) = 944, μ = 4.480 mm^{-1}, T = 170 K, θ_{max} = 25.250°, 9661 total reflections, 4394 with $I_o > 2\sigma(I_o)$, R_{int} = 0.0239, 5039 data, 363 parameters, 0 restraints, GooF = 1.052, R = 0.0259 and wR = 0.0568 [$I_o > 2\sigma(I_o)$], R = 0.0330 and wR = 0.0592 (all reflections), 1.003<dΔρ < -0.709 $eÅ^{-3}$.

5. Complex oDIB-4a

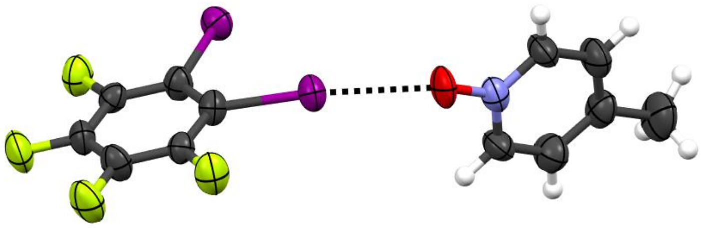

Figure S16. The X-ray crystal structure of **oDIB-4a** with the thermal displacement parameter at 50% probability level. The black broken line represents the halogen bond. Colour Key: green = fluorine, purple = iodine, blue = nitrogen, dark grey = carbon, white = hydrogen. **Notes**: The asymmetric unit consists of one **oDIB** and one PyNO molecule.

Crystal data for oDIB-4a (crystallization solvent: $CHCl_3$): CCDC- 2337237, $C_{12}H_7F_4I_2NO$, M = 510.99 $gmol^{-1}$, colourless plate, 0.30 × 0.17 × 0.08 mm, triclinic, space group *P-1*, a = 7.9641(2) Å, b = 8.68497(19) Å, c = 11.71195(17) Å, α = 90.9045(14)°, β = 93.4378(16)°, γ = 115.168(2)°, V = 731.10(3) $Å^3$, Z = 2, D_{calc} = 2.321 gcm^{-3}, F(000) = 472, μ = 4.339 mm^{-1}, T = 170 K, θ_{max} = 25.247°, 116800 total reflections, 76593 with $I_o > 2\sigma(I_o)$, R_{int} = twin, 116800 data, 183 parameters, 0 restraints, GooF = 0.962, R = 0.0607 and wR = 0.1446 [$I_o > 2\sigma(I_o)$], R = 0.0863 and wR = 0.1666 (all reflections), 2.350<dΔρ < -1.221 $eÅ^{-3}$.

6. Complex oDIB-5

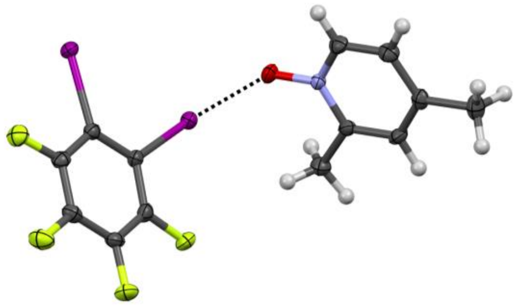

Figure S17. The X-ray crystal structure of **oDIB-5** with the thermal displacement parameter at 50% probability level. The black broken line represents the halogen bond. Colour Key: green = fluorine, purple = iodine, blue = nitrogen, dark grey = carbon, white = hydrogen. **Notes**: The asymmetric unit consists of one **oDIB** and one PyNO molecule.

Crystal data for oDIB-5 (crystallization solvent: $CHCl_3$): CCDC- 2337238, $C_{13}H_9F_4I_2NO$, M = 525.01 gmol^{-1}, colourless plate, 0.11 × 0.08 × 0.04 mm, triclinic, space group *P-1*, a = 7.8596(2) Å, b = 8.1053(2) Å, c = 12.3697(2) Å, α = 104.993(2)°, β = 94.568(2)°, γ = 91.984(2)°, V = 757.51(3) Å^3, Z = 2, D_{calc} = 2.302 gcm^{-3}, F(000) = 488, μ = 33.014 mm^{-1}, T = 123 K, θ_{max} = 66.734°, 11080 total reflections, 2540 with $I_o > 2\sigma(I_o)$, R_{int} = 0.0445, 2689 data, 192 parameters, 0 restraints, GooF = 1.029, R = 0.0233 and wR = 0.0588 [$I_o > 2\sigma(I_o)$], R = 0.0248 and wR = 0.0597 (all reflections), 0.539<dΔρ < -0.864 eÅ^{-3}.

7. Complex oDIB-6

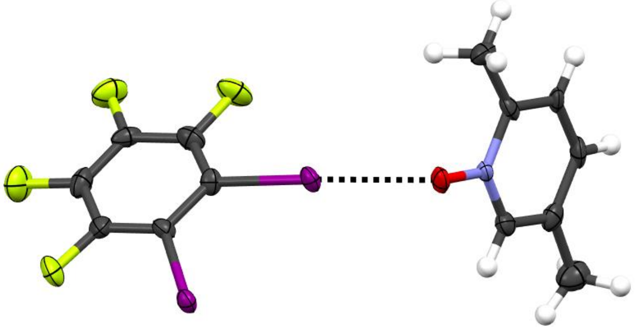

Figure S18. The X-ray crystal structure of **oDIB-6** with the thermal displacement parameter at 50% probability level. The black broken line represents the halogen bond. Colour Key: green = fluorine, purple = iodine, blue = nitrogen, dark grey = carbon, white = hydrogen. **Notes**: The asymmetric unit consists of one **oDIB** and one PyNO molecule.

Crystal data for oDIB-6 (crystallization solvent: $CHCl_3$): CCDC-2337239, $C_{13}H_9F_4I_2NO$, M = 525.01 gmol^{-1}, colourless block, 0.18 × 0.12 × 0.07 mm, triclinic, space group *P-1*, a = 7.5445(9) Å, b = 8.6260(10) Å, c = 12.6918(14) Å, α = 73.031(10)°, β = 74.663(10)°, γ = 85.033(10)°, V = 761.84(16) Å^3, Z = 2, D_{calc} = 2.289 gcm^{-3}, F(000) = 488, μ = 4.167 mm^{-1}, T = 123 K, θ_{max} = 25.250°, 4030 total reflections, 3568 with $I_o > 2\sigma(I_o)$, R_{int} = 0.0423, 4030 data, 193 parameters, 0 restraints, GooF = 1.192, R = 0.0434 and wR = 0.1099 [$I_o > 2\sigma(I_o)$], R = 0.0493 and wR = 0.1133 (all reflections), 1.847<dΔρ < -1.349 eÅ^{-3}.

8. Complex oDIB-7

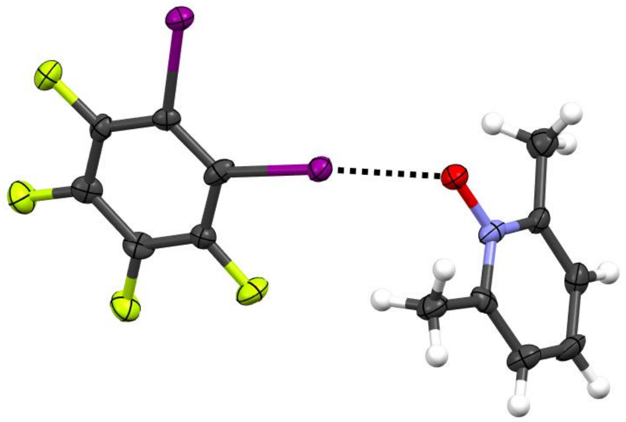

Figure S19. The X-ray crystal structure of **oDIB-7** with the thermal displacement parameter at 50% probability level. The black broken line represents the halogen bond. Colour Key: green = fluorine, purple = iodine, blue = nitrogen, dark grey = carbon, white = hydrogen. **Notes**: The asymmetric unit consists of one **oDIB** and one PyNO molecule.

Crystal data for oDIB-7 (crystallization solvent: $CHCl_3$): CCDC- 2337240, $C_{13}H_9F_4I_2NO$, M = 525.01 gmol^{-1}, colourless block, 0.18 × 0.14 × 0.14 mm, triclinic, space group *P-1*, a = 7.8838(7) Å, b = 9.4886(9) Å, c = 11.1690(13) Å, α = 77.040(9)°, β = 70.007(10)°, γ = 84.056(8)°, V = 764.83(14) Å^3, Z = 2, D_{calc} = 2.280 gcm^{-3}, F(000) = 488, μ = 4.151 mm^{-1}, T = 120 K, θ_{max} = 25.250°, 4303 total reflections, 2077 with $I_o > 2\sigma(I_o)$, R_{int} = 0.0406, 2766 data, 192 parameters, 0 restraints, GooF = 1.090, R = 0.0500 and wR = 0.1001 [$I_o > 2\sigma(I_o)$], R = 0.0760 and wR = 0.1115 (all reflections), 1.535 <dΔρ < -1.149 eÅ^{-3}.

9. Complex oDIB-8

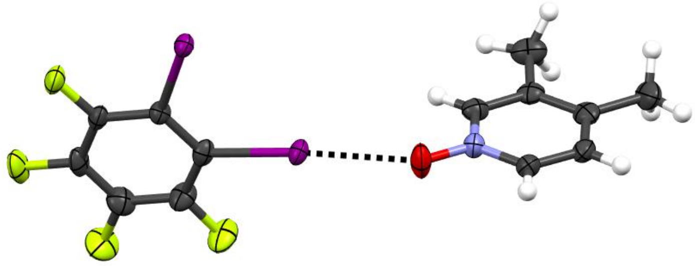

Figure S20. The X-ray crystal structure of **oDIB-8** with the thermal displacement parameter at 50% probability level. The black broken line represents the halogen bond. Colour Key: green = fluorine, purple = iodine, blue = nitrogen, dark grey = carbon, white = hydrogen. **Notes**: The asymmetric unit contains four molecules of **oDIB** and four molecules of PyNO. For clarity, only the 1:1 donor:acceptor ratio complex is displayed.

Crystal data for oDIB-8 (crystallization solvent: $CHCl_3$): CCDC-2337241, $C_{13}H_9F_4I_2NO$, M = 525.01 gmol^{-1}, colourless block, 0.12 × 0.07 × 0.07 mm, triclinic, space group *P-1*, a = 14.4926(2) Å, b = 15.0727(2) Å, c = 15.0821(3) Å, α = 86.8170(10)°, β = 70.9220(10)°, γ = 77.9800(10)°, V = 3045.07(9) Å^3, Z = 8, D_{calc} = 2.290 gcm^{-3}, F(000) = 1952, μ = 32.851 mm^{-1}, T = 120 K, θ_{max} = 66.743°, 60261 total reflections, 10044 with $I_o > 2\sigma(I_o)$, R_{int} = 0.0429, 10774 data, 747 parameters, 0 restraints, GooF = 1.031, R = 0.0470 and wR = 0.1208 [$I_o > 2\sigma(I_o)$], R = 0.0499 and wR = 0.1228 (all reflections), 7.734 <dΔρ < -2.186 eÅ^{-3}.

10. Complex oDIB-9

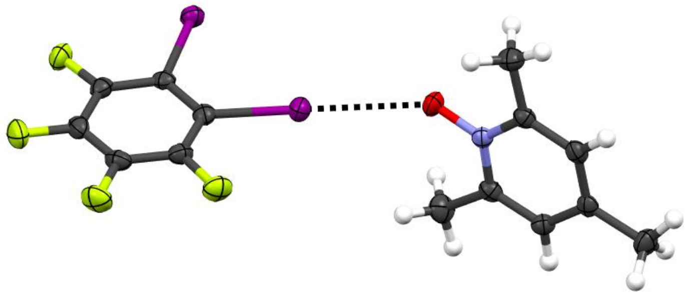

Figure S21. The X-ray crystal structure of **oDIB-9** with the thermal displacement parameter at 50% probability level. The black broken line represents the halogen bond. Colour Key: green = fluorine, purple = iodine, blue = nitrogen, dark grey = carbon, white = hydrogen. **Notes**: The asymmetric unit consists of two **oDIB** molecules and two PyNO molecules. For viewing clarity, just the 1:1 donor:acceptor ratio complex is shown.

Crystal data for oDIB-9 (crystallization solvent: $CHCl_3$): CCDC-2337242, $C_{14}H_{11}F_4I_2NO$, M = 539.04 $gmol^{-1}$, colourless plate, 0.27 × 0.2 × 0.08 mm, triclinic, space group *P-1*, a = 7.9055(2) Å, b = 8.5386(3) Å, c = 24.4563(8) Å, α = 96.781(3)°, β = 95.895(3)°, γ = 90.108(3)°, V = 1630.47(9) $Å^3$, Z = 4, D_{calc} = 2.196 gcm^{-3}, F(000) = 1008, μ = 3.897 mm^{-1}, T = 120 K, θ_{max} = 29.936°, 25516 total reflections, 7359 with $I_o > 2\sigma(I_o)$, R_{int} = 0.0292, 8532 data, 403 parameters, 0 restraints, GooF = 1.082, R = 0.0281 and wR = 0.0496 [$I_o > 2\sigma(I_o)$], R = 0.0356 and wR = 0.0521 (all reflections), 0.592<dΔρ < -0.643 $eÅ^{-3}$.

11. Complex oDIB-10

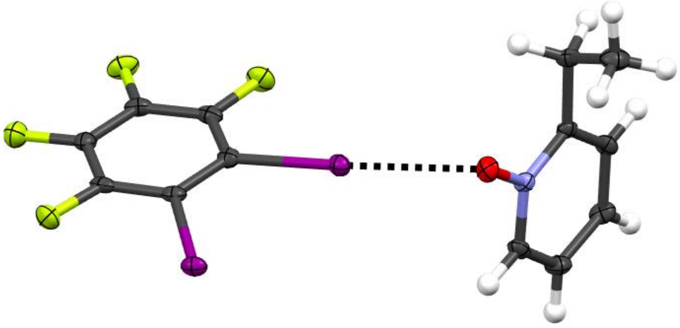

Figure S22. The X-ray crystal structure of **oDIB-10** with the thermal displacement parameter at 50% probability level. The black broken line represents the halogen bond. Colour Key: green = fluorine, purple = iodine, blue = nitrogen, dark grey = carbon, white = hydrogen. **Notes**: The asymmetric unit consists of one **oDIB** and one PyNO molecule.

Crystal data for oDIB-10 (crystallization solvent: $CHCl_3$): CCDC- 2337243, $C_{13}H_9F_4I_2NO$, M = 525.01 $gmol^{-1}$, colourless block, 0.253 × 0.163 × 0.12 mm, triclinic, space group *P-1*, a = 7.4025(5) Å, b = 8.8458(5)Å, c = 12.8667(8) Å, α = 104.676(5)°, β = 105.857(6)°, γ = 99.553(5)°, V = 758.57(9) $Å^3$, Z = 2, D_{calc} = 2.299 gcm^{-3}, F(000) = 488, μ = 4.185 mm^{-1}, T = 180 K, θ_{max} = 25.248°, 5056 total reflections, 2547 with $I_o > 2\sigma(I_o)$, R_{int} = 0.0227, 2751 data, 191 parameters, 0 restraints, GooF = 1.022, R = 0.0194 and wR = 0.0425 [$I_o > 2\sigma(I_o)$], R = 0.0216 and wR = 0.0437 (all reflections), 0.611<dΔρ < -0.683 $eÅ^{-3}$.

12. Complex oDIB-11

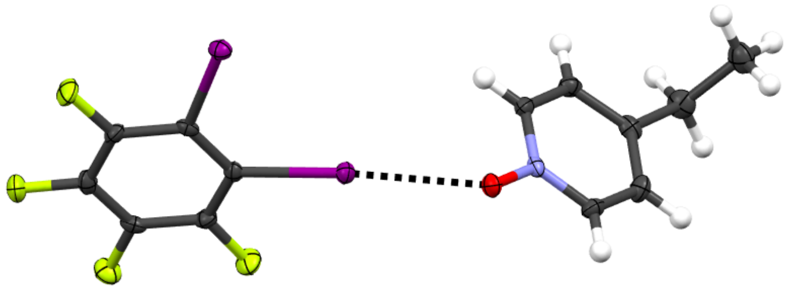

Figure S23. The X-ray crystal structure of **oDIB-11** with the thermal displacement parameter at 50% probability level. The black broken line represents the halogen bond. Colour Key: green = fluorine, purple = iodine, blue = nitrogen, dark grey = carbon, white = hydrogen. **Notes**: The asymmetric unit consists of one **oDIB** and one PyNO molecule.

Crystal data for oDIB-11 (crystallization solvent: $CHCl_3$): CCDC- 2337244, $C_{13}H_9F_4I_2NO$, M = 525.01 gmol^{-1}, colourless block, 0.19 × 0.14 × 0.09 mm, monoclinic, space group *P2$_1$/c*, a = 11.2587(2) Å, b = 18.3684(3) Å, c = 7.4901(2) Å, α = 90°, β = 96.961(2)°, γ = 90°, V = 1537.57(6) Å^3, Z = 4, D_{calc} = 2.268 gcm^{-3}, F(000) = 976, μ = 4.129 mm^{-1}, T = 180 K, θ_{max} = 25.250°, 11038 total reflections, 2545 with $I_o > 2\sigma(I_o)$, R_{int} = 0.0289, 2774 data, 191 parameters, 0 restraints, GooF = 1.071, R = 0 0.0182 and wR = 0.0359 [$I_o > 2\sigma(I_o)$], R = 0.0214 and wR = 0.0371 (all reflections), 0.461<dΔρ < -0.408 eÅ^{-3}.

13. Complex oDIB-12

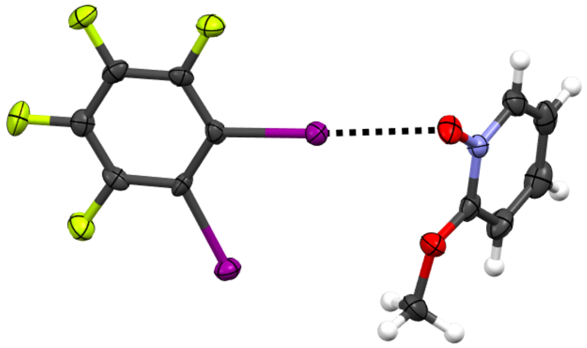

Figure S24. The X-ray crystal structure of **oDIB-12** with the thermal displacement parameter at 50% probability level. The black broken line represents the halogen bond. Colour Key: green = fluorine, purple = iodine, blue = nitrogen, dark grey = carbon, white = hydrogen. **Notes**: The asymmetric unit consists of two **oDIB** molecules and two PyNO molecules. For viewing clarity, just the 1:1 donor:acceptor ratio complex is shown.

Crystal data for oDIB-12 (crystallization solvent: $CHCl_3$): CCDC-2337245, $C_{12}H_7F_4I_2NO_2$, M = 526.99 gmol^{-1}, colourless prism, 0.140 × 0.110 × 0.090 mm, triclinic, space group *P-1*, a = 7.3072(3) Å, b = 13.6208(3) Å, c = 15.2311(5) Å, α = 88.581(2)°, β = 78.6010(10)°, γ = 83.225(2)°, V = 1475.67(8) Å^3, Z = 4, D_{calc} = 2.372 gcm^{-3}, F(000) = 976, μ = 4.308 mm^{-1}, T = 170(2) K, θ_{max} = 27.813°, 13126 total reflections, 5227 with $I_o > 2\sigma(I_o)$, R_{int} = 0.0542, 6982 data, 379 parameters, 0 restraints, GooF = 1.010, R = 0.0463 and wR = 0.0731 [$I_o > 2\sigma(I_o)$], R = 0.0723 and wR = 0.0810 (all reflections), 0.851<dΔρ < -0.823 eÅ^{-3}.

14. Complex oDIB-12a

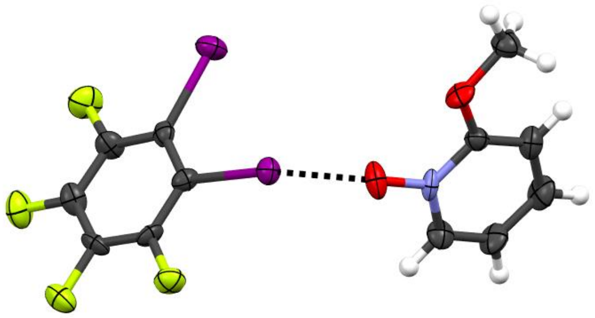

Figure S25. The X-ray crystal structure of **oDIB-12a** with the thermal displacement parameter at 50% probability level. The black broken line represents the halogen bond. Colour Key: green = fluorine, purple = iodine, blue = nitrogen, dark grey = carbon, white = hydrogen. **Notes**: The asymmetric unit consists of six **oDIB** molecules and two PyNO molecules. For viewing clarity, just the 1:1 donor:acceptor ratio complex is shown.

Crystal data for oDIB-12a (crystallization solvent: $CHCl_3$): CCDC-2337246, $C_{24}H_7F_{12}I_6NO_2$, M = 1330.71 gmol^{-1}, colourless prism, 0.220 × 0.04 × 0.03 mm, triclinic, space group *P-1*, a = 7.95020(10) Å, b = 14.3526(3) Å, c = 28.7384(6) Å, α = 84.3490(10)°, β = 85.3340(10)°, γ = 77.1500(10)°, V = 3175.58(10) Å^3, Z = 4, D_{calc} = 2.783 gcm^{-3}, F(000) = 2400, μ = 5.966 mm^{-1}, T = 170(2) K, θ_{max} = 27.500°, 22057 total reflections, 10036 with $I_o > 2\sigma(I_o)$, R_{int} = 0.0417, 14244 data, 811 parameters, 0 restraints, GooF = 1.084, R = 0.0570 and wR = 0.0989 [$I_o > 2\sigma(I_o)$], R = 0.0896 and wR = 0.1090 (all reflections), 0.960<dΔρ < -0.982 eÅ^{-3}.

15. Complex oDIB-12b

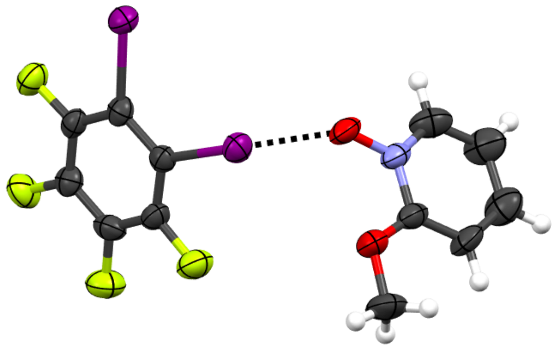

Figure S26. The X-ray crystal structure of **oDIB-12b** with the thermal displacement parameter at 50% probability level. The black broken line represents the halogen bond. Colour Key: green = fluorine, purple = iodine, blue = nitrogen, dark grey = carbon, white = hydrogen. **Notes**: The asymmetric unit consists of one **oDIB** and one PyNO molecule.

Crystal data for oDIB-12b (crystallization solvent: $CHCl_3$): CCDC- 2337247, $C_{12}H_7F_4I_2NO_2$, M = 526.99 gmol^{-1}, colourless prism, 0.15 × 0.12 × 0.11 mm, monoclinic, space group *P2₁/c*, a = 12.7141(6) Å, b = 8.2936(3) Å, c = 15.1565(8) Å, α = 90°, β = 107.835(5)°, γ = 90°, V = 1521.38(13) Å^3, Z = 4, D_{calc} = 2.301 gcm^{-3}, F(000) = 976, μ = 4.179 mm^{-1}, T = 123 K, θ_{max} = 25.246°, 5479 total reflections, 1855 with $I_o > 2\sigma(I_o)$, R_{int} = 0.0351, 2738 data, 191 parameters, 0 restraints, GooF = 1.009, R = 0.0384 and wR = 0.0515 [$I_o > 2\sigma(I_o)$], R = 0.0688 and wR = 0.0634 (all reflections), 0.831<dΔρ < -0.733 eÅ^{-3}.

16. Complex oDIB-13

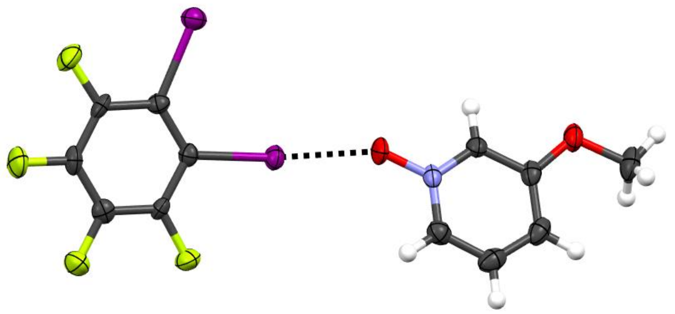

Figure S27. The X-ray crystal structure of **oDIB-13** with the thermal displacement parameter at 50% probability level. The black broken line represents the halogen bond. Colour Key: green = fluorine, purple = iodine, blue = nitrogen, dark grey = carbon, white = hydrogen. **Notes**: The asymmetric unit consists of two **oDIB** molecules and two PyNO molecules. For viewing clarity, just the 1:1 donor:acceptor ratio complex is shown.

Crystal data for oDIB-13 (crystallization solvent: $CHCl_3$): CCDC-2337248, $C_{12}H_7F_4I_2NO_2$, M = 526.99 gmol^{-1}, colourless prism, 0.280 × 0.120 × 0.1 mm, triclinic, space group *P-1*, a = 7.7565(2) Å, b = 13.1707(3) Å, c = 15.0431(2) Å, α = 95.2820(10)°, β = 98.1700(10)°, γ = 104.6980(10)°, V = 1458.17(5) Å^3, Z = 4, D_{calc} = 2.400 gcm^{-3}, F(000) = 976, μ = 4.360 mm^{-1}, T = 173(2) K, θ_{max} = 28.853°, 13507 total reflections, 5609 with $I_o > 2\sigma(I_o)$, R_{int} = 0.0366, 7547 data, 379 parameters, 0 restraints, GooF = 1.088, R = 0.0443 and wR = 0.0752 [$I_o > 2\sigma(I_o)$], R = 0.0687 and wR = 0.0818 (all reflections), 0.738<dΔρ < -0.832 eÅ^{-3}.

17. Complex oDIB-14

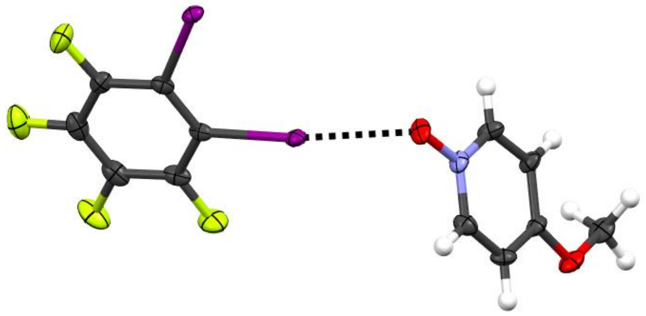

Figure S28. The X-ray crystal structure of **oDIB-14** with the thermal displacement parameter at 50% probability level. The black broken line represents the halogen bond. Colour Key: green = fluorine, purple = iodine, blue = nitrogen, dark grey = carbon, white = hydrogen. **Notes**: The asymmetric unit consists of two **oDIB** molecules and two PyNO molecules. For viewing clarity, just the 1:1 donor:acceptor ratio complex is shown.

Crystal data for oDIB-14 (crystallization solvent: $CHCl_3$): CCDC-2337249, $C_{12}H_7F_4I_2NO_2$, M = 526.99 gmol^{-1}, colourless plate, 0.23 × 0.12 × 0.08 mm, monoclinic, space group *P2₁/n*, a = 11.21599(13) Å, b = 14.59126(16) Å, c = 18.0115(2) Å, α = 90°, β = 94.9203(10) °, γ = 90°, V = 2936.83(6) Å^3, Z = 8, D_{calc} = 2.384 gcm^{-3}, F(000) = 1952, μ = 34.120 mm^{-1}, T = 123 K, θ_{max} = 66.746°, 16372 total reflections, 4760 with $I_o > 2\sigma(I_o)$, R_{int} = 0.0491, 5200 data, 381 parameters, 0 restraints, GooF = 1.054, R = 0.0333 and wR = 0.0825 [$I_o > 2\sigma(I_o)$], R = 0.0361 and wR = 0.0856 (all reflections), 1.063<dΔρ < -1.252 eÅ^{-3}.

18. Complex oDIB-15

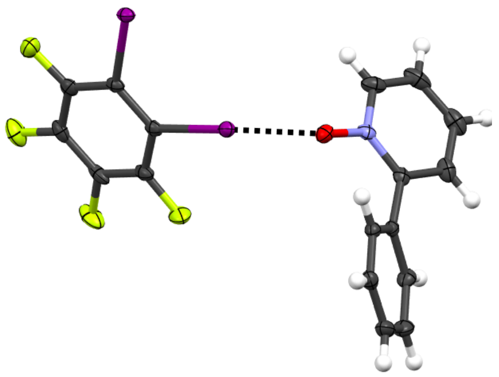

Figure S29. The X-ray crystal structure of **oDIB-15** with the thermal displacement parameter at 50% probability level. The black broken line represents the halogen bond. Colour Key: green = fluorine, purple = iodine, blue = nitrogen, dark grey = carbon, white = hydrogen. **Notes**: The asymmetric unit consists of one **oDIB** and one PyNO molecule.

Crystal data for oDIB-15 (crystallization solvent: $CHCl_3$): CCDC-2337250, $C_{17}H_9F_4I_2NO$, M = 573.05 gmol^{-1}, colourless block, 0.1 × 0.08 × 0.07 mm, triclinic, space group *P-1*, a = 7.64450(10) Å, b = 8.70930(10) Å, c = 13.39840(10) Å, α = 77.1590(10)°, β = 88.8370(10)°, γ = 87.4190(10)°, V = 868.802(17) Å^3, Z = 2, D_{calc} = 2.191 gcm^{-3}, F(000) = 536, μ = 28.868 mm^{-1}, T = 120 K, θ_{max} = 66.744°, 12660 total reflections, 3030 with $I_o > 2\sigma(I_o)$, R_{int} = 0.0190, 3069 data, 226 parameters, 0 restraints, GooF = 1.103, R = 0.0177 and wR = 0.0457 [$I_o > 2\sigma(I_o)$], R = 0.0179 and wR = 0.0459 (all reflections), 0.750<dΔρ < -0.725 eÅ^{-3}.

19. Complex oDIB-15a

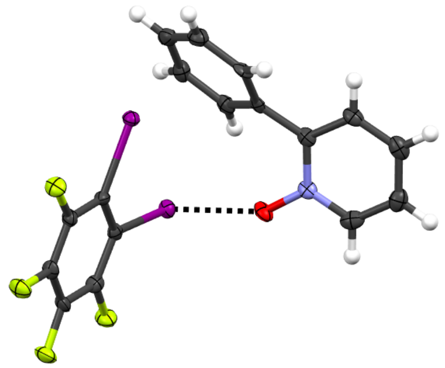

Figure S30. The X-ray crystal structure of **oDIB-15a** with the thermal displacement parameter at 50% probability level. The black broken line represents the halogen bond. Colour Key: green = fluorine, purple = iodine, blue = nitrogen, dark grey = carbon, white = hydrogen. **Notes**: The asymmetric unit consists of 1.5 oDIB molecules and one PyNO molecule. For viewing clarity, just the 1:1 donor:acceptor ratio complex is shown. **oDIB-15** and **oDIB-15a** measured from the same bulk sample.

Crystal data for oDIB-15a (crystallization solvent: $CHCl_3$): CCDC-2337251, $C_{20}H_9F_6I_3NO$, M = 773.98 gmol^{-1}, colourless block, 0.13 × 0.11 × 0.1 mm, monoclinic, space group *C2/c*, a = 18.2635(5) Å, b = 13.5683(3) Å, c = 18.2088(5) Å, α = 90°, β = 107.929(3)°, γ = 90°, V = 4293.10(19) Å^3, Z = 8, D_{calc} = 2.395 gcm^{-3}, F(000) = 2856, μ = 4.433 mm^{-1}, T = 120 K, θ_{max} = 25.249°, 15226 total reflections, 3200 with $I_o > 2\sigma(I_o)$, R_{int} = 0.0517, 3891 data, 280 parameters, 0 restraints, GooF = 1.055, R = 0.0302 and wR = 0.0671 [$I_o > 2\sigma(I_o)$], R = 0.0430 and wR = 0.0741 (all reflections), 1.467<dΔρ <-1.502 eÅ^{-3}.

20. Complex oDIB-17

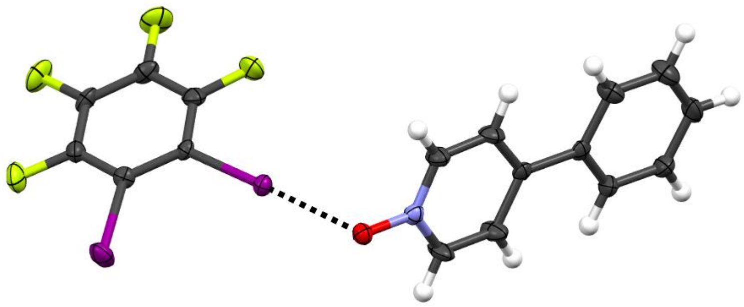

Figure S31. The X-ray crystal structure of **oDIB-17** with the thermal displacement parameter at 50% probability level. The black broken line represents the halogen bond. Colour Key: green = fluorine, purple = iodine, blue = nitrogen, dark grey = carbon, white = hydrogen. **Notes**: The asymmetric unit consists of two **oDIB** molecules and one PyNO molecule. For viewing clarity, just the 1:1 donor:acceptor ratio complex is shown.

Crystal data for oDIB-17 (crystallization solvent: $CHCl_3$): CCDC-2337252, $C_{23}H_9F_8I_4NO$, M = 974.91 gmol^{-1}, colourless block, 0.21 × 0.19 × 0.12 mm, monoclinic, space group *C2/c*, a = 27.069(5) Å, b = 8.2329(16) Å, c = 24.070(5) Å, α = 90°, β = 104.46(3)°, γ = 90°, V = 5194.4(19) Å^3, Z = 8, D_{calc} = 2.493 gcm^{-3}, F(000) = 3568, μ = 4.875 mm^{-1}, T = 170 K, θ_{max} = 25.249°, 14783 total reflections, 3831 with $I_o > 2\sigma(I_o)$, R_{int} = 0.0473, 4683 data, 334 parameters, 0 restraints, GooF = 1.042, R = 0.0313 and wR = 0.0595 [$I_o > 2\sigma(I_o)$], R = 0.0440 and wR = 0.0632 (all reflections), 0.529<dΔρ < -0.622 eÅ^{-3}.

21. Complex oDIB-18

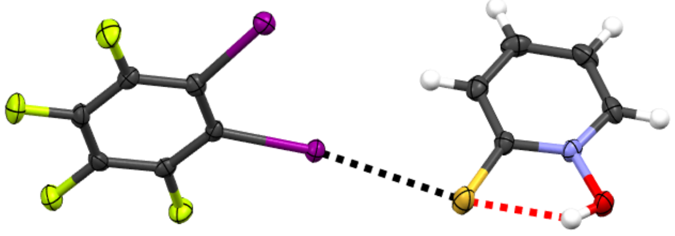

Figure S32. The X-ray crystal structure of **oDIB-18** with the thermal displacement parameter at 50% probability level. The black broken line represents the halogen bond and red broken line represents the hydrogen bond. Colour Key: green = fluorine, purple = iodine, blue = nitrogen, dark grey = carbon, white = hydrogen. **Notes**: The asymmetric unit consists of two **oDIB** molecules and two PyNO molecules. For viewing clarity, just the 1:1 donor:acceptor ratio complex is shown.

Crystal data for oDIB-18 (crystallization solvent: $CHCl_3$): CCDC-2337253, $C_{11}H_5F_4I_2NOS$, M = 529.02 gmol^{-1}, colourless stick/rod, 0.23 × 0.09 × 0.05 mm, triclinic, space group *P-1*, a = 7.8866(5) Å, b = 12.5079(5) Å, c = 15.0257(6) Å, α = 102.433(4)°, β = 96.690(4)°, γ = 99.416(4)°, V = 1410.13(12) Å^3, Z = 4, D_{calc} = 2.492 gcm^{-3}, F(000) = 976, μ = 4.646 mm^{-1}, T = 123 K, θ_{max} = 29.908°, 12197 total reflections, 5814 with $I_o > 2\sigma(I_o)$, R_{int} = 0.0240, 7018 data, 367 parameters, 2 restraints, GooF = 1.024, R = 0.0295 and wR = 0.0536 [$I_o > 2\sigma(I_o)$], R = 0.0411 and wR = 0.0587 (all reflections), 0.729<dΔρ < -0.669 eÅ^{-3}.

22. Complex oDIB-18a

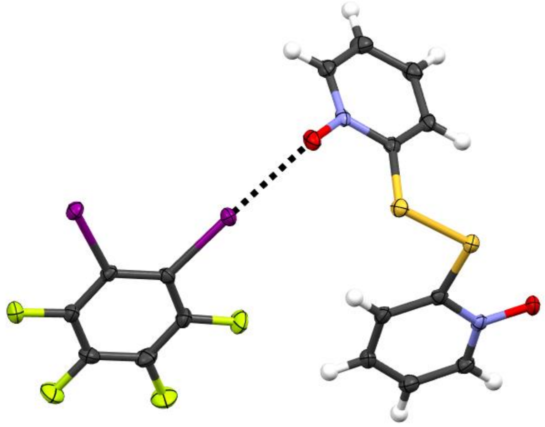

Figure S33. The X-ray crystal structure of **oDIB-18a** with the thermal displacement parameter at 50% probability level. The black broken line represents the halogen bond. Colour Key: green = fluorine, purple = iodine, blue = nitrogen, dark grey = carbon, white = hydrogen. **Notes**: The asymmetric unit consists of one **oDIB** molecule and 0.5 PyNO molecule. For viewing clarity, just the 1:1 donor:acceptor ratio complex is shown.

Crystal data for oDIB-18a (crystallization solvent: $CHCl_3$): CCDC- 2337807, $C_{22}H_8F_8I_4N_2O_2S_2$, M = 1056.02 gmol^{-1}, colourless block, 0.11 × 0.08 × 0.04 mm, monoclinic, space group *P2/c*, a = 12.0021(2) Å, b = 4.34120(10) Å, c = 27.3027(3) Å, α = 90°, β = 98.0650(10)°, γ = 90°, V = 1408.50(4) Å^3, Z = 2, D_{calc} = 2.490 gcm^{-3}, F(000) = 972, μ = 36.871 mm^{-1}, T = 120 K, θ_{max} = 66.719°, 11849 total reflections, 2418 with $I_o > 2\sigma(I_o)$, R_{int} = 0.0264, 2497 data, 181 parameters, 0 restraints, GooF = 1.127, R = 0.0213 and wR = 0.0552 [$I_o > 2\sigma(I_o)$], R = 0.0222 and wR = 0.0558 (all reflections), 1.005<dΔρ < -0.571 eÅ^{-3}.

23. Complex oDIB-19

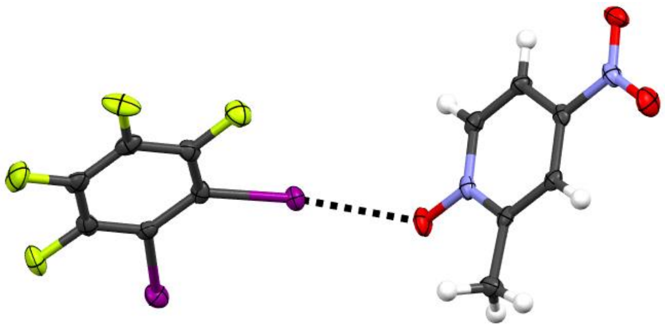

Figure S34. The X-ray crystal structure of **oDIB-19** with the thermal displacement parameter at 50% probability level. The black broken line represents the halogen bond and red broken line represents the hydrogen bond. Colour Key: green = fluorine, purple = iodine, blue = nitrogen, dark grey = carbon, white = hydrogen. **Notes**: The asymmetric unit consists of one **oDIB** molecule and 1.5 PyNO molecules. For viewing clarity, just the 1:1 donor:acceptor ratio complex is shown.

Crystal data for oDIB-19 (crystallization solvent: $CHCl_3$): CCDC-2337254, $C_{30}H_{18}F_8I_4N_6O_9$, M = 1266.10 gmol^{-1}, colourless block, 0.09 × 0.09 × 0.04 mm, orthorhombic, space group *Pnma*, a = 12.2911(3) Å, b = 34.0825(7)Å, c = 8.84928(17) Å, α = 90°, β = 90°, γ = 90°, V = 3707.08(13)Å^3, Z = 4, D_{calc} = 2.269 gcm^{-3}, F(000) = 2384, μ = 3.463 mm^{-1}, T = 123 K, θ_{max} = 25.249°, 11631 total reflections, 2816 with $I_o > 2\sigma(I_o)$, R_{int} = 0.0317, 3417 data, 276 parameters, 0 restraints, GooF = 1.028, R = 0.0352 and wR = 0.0794 [$I_o > 2\sigma(I_o)$], R = 0.0461 and wR = 0.0854 (all reflections), 2.527<dΔρ < -0.530 eÅ^{-3}.

24. Complex oDIB-20

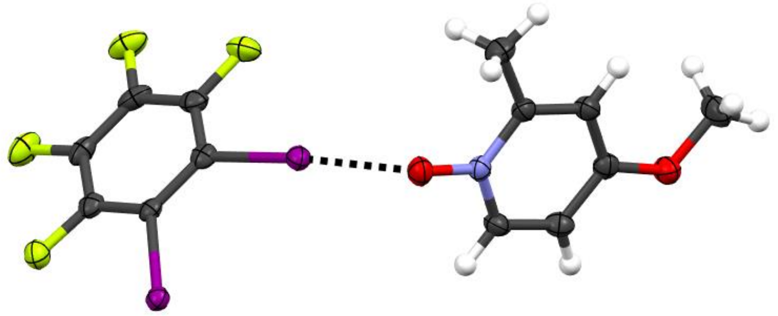

Figure S35. The X-ray crystal structure of **oDIB-20** with the thermal displacement parameter at 50% probability level. The black broken line represents the halogen bond. Colour Key: green = fluorine, purple = iodine, blue = nitrogen, dark grey = carbon, white = hydrogen. **Notes**: The asymmetric unit consists of one **oDIB** and one PyNO molecule.

Crystal data for oDIB-20 (crystallization solvent: $CHCl_3$): CCDC-2337255, $C_{13}H_9F_4I_2NO_2$, M = 541.01 gmol^{-1}, colourless plate, 0.22 × 0.21 × 0.06 mm, monoclinic, space group *P2₁/c*, a = 7.63590(10) Å, b = 8.05660(10) Å, c = 26.0081(3) Å, α = 90°, β = 96.7520(10)°, γ = 90°, V = 1588.91(3) Å^3, Z = 4, D_{calc} = 2.262 gcm^{-3}, F(000) = 1008, μ = 31.556 mm^{-1}, T = 120 K, θ_{max} = 66.697°, 10528 total reflections, 2732 with $I_o > 2\sigma(I_o)$, R_{int} = 0.0342, 2822 data, 201 parameters, 0 restraints, GooF = 1.068, R = 0.0265 and wR = 0.0673 [$I_o > 2\sigma(I_o)$], R = 0.0273 and wR = 0.0679 (all reflections), 0.898<dΔρ < -1.167 eÅ^{-3}.

25. Complex oDIB-21

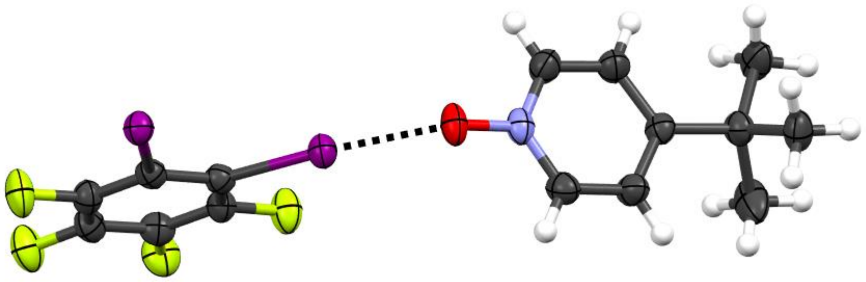

Figure S36. The X-ray crystal structure of **oDIB-21** with the thermal displacement parameter at 50% probability level. The black broken line represents the halogen bond. Colour Key: green = fluorine, purple = iodine, blue = nitrogen, dark grey = carbon, white = hydrogen. **Notes**: The asymmetric unit consists of one **oDIB** and one PyNO molecule. The disordered tert-butyl group is omitted for viewing clarity.

Crystal data for oDIB-21 (crystallization solvent: EtOAc or $CHCl_3$): CCDC-2337256, $C_{15}H_{13}F_4I_2NO$, M = 553.06 gmol^{-1}, colourless block, 0.198 × 0.142 × 0.118 mm, monoclinic, space group *C2/c*, a = 19.1856(2) Å, b = 8.43820(10) Å, c = 22.7977(2) Å, α = 90°, β = 96.8040(10)°, γ = 90°, V = 3664.77(7)Å^3, Z = 8, D_{calc} = 2.005 gcm^{-3}, F(000) = 2080, μ = 27.336 mm^{-1}, T = 120 K, θ_{max} = 66.750°, 26156 total reflections, 3180 with $I_o > 2\sigma(I_o)$, R_{int} = 0.0472, 3245 data, 241 parameters, 0 restraints, GooF = 1.105, R = 0.0214 and wR = 0.0564 [$I_o > 2\sigma(I_o)$], R = 0.0218 and wR = 0.0567 (all reflections), 0.356<dΔρ <-0.356 eÅ^{-3}.

26. Complex oDIB-23

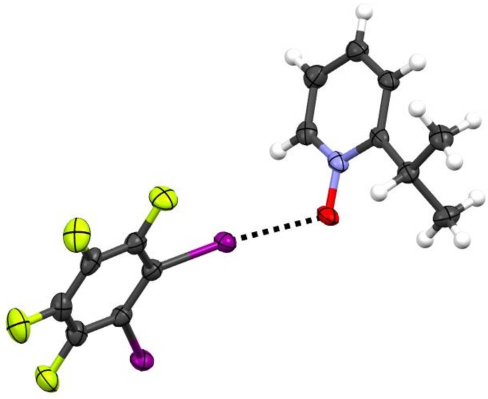

Figure S37. The X-ray crystal structure of **oDIB-23** with the thermal displacement parameter at 50% probability level. The black broken line represents the halogen bond. Colour Key: green = fluorine, purple = iodine, blue = nitrogen, dark grey = carbon, white = hydrogen. **Notes**: The asymmetric unit consists of one **oDIB** and one PyNO molecule.

Crystal data for oDIB-23 (crystallization solvent: $CHCl_3$): CCDC-2337257, $C_{14}H_{11}F_4I_2NO$, M = 539.04 gmol^{-1}, colourless block, 0.2 × 0.2 × 0.18 mm, triclinic, space group P-1, a = 8.0413(16) Å, b = 8.4364(17) Å, c = 13.211(3) Å, α = 107.12(3)°, β = 107.49(3)°, γ = 91.79(3)°, V = 809.8(3)Å^3, Z = 2, D_{calc} = 2.211 gcm^{-3}, F(000) = 504, μ = 3.923 mm^{-1}, T = 180 K, θ_{max} = 25.247°, 5580 total reflections, 2520 with $I_o > 2\sigma(I_o)$, R_{int} = 0.0257, 2931 data, 201 parameters, 0 restraints, GooF = 1.071, R = 0.0283 and wR = 0.0583 [$I_o > 2\sigma(I_o)$], R = 0.0355 and wR = 0.0605 (all reflections), 0.443<dΔρ < -0.447 eÅ^{-3}.

27. Complex oDIB-24

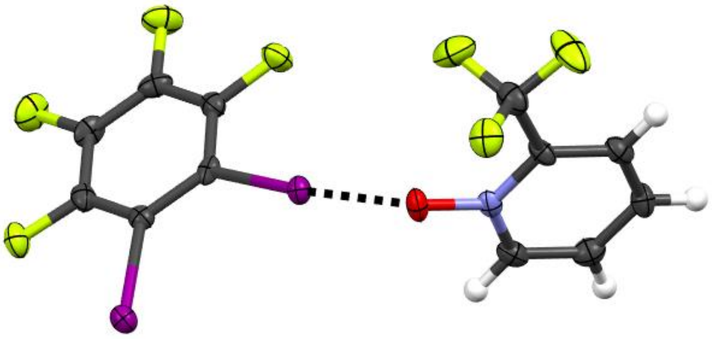

Figure S38. The X-ray crystal structure of **oDIB-24** with the thermal displacement parameter at 50% probability level. The black broken line represents the halogen bond. Colour Key: green = fluorine, purple = iodine, blue = nitrogen, dark grey = carbon, white = hydrogen. **Notes**: The asymmetric unit consists of one **oDIB** and one PyNO molecule.

Crystal data for oDIB-24 (crystallization solvent: $CHCl_3$): CCDC-2337258, $C_{12}H_4F_7I_2NO$, M = 564.96 gmol^{-1}, colourless block, 0.13 × 0.11 × 0.07 mm, triclinic, space group P-1, a = 7.7871(4) Å, b = 8.3460(5) Å, c = 12.8485(8) Å, α = 107.008(6)°, β = 106.797(5)°, γ = 93.543(5)°, V = 754.62(8) Å^3, Z = 2, D_{calc} = 2.486 gcm^{-3}, F(000) = 520, μ = 4.244 mm^{-1}, T = 123 K, θ_{max} = 25.245°, 4237 total reflections, 2354 with $I_o > 2\sigma(I_o)$, R_{int} = 0.0208, 2704 data, 208 parameters, 0 restraints, GooF = 1.009, R = 0.0246 and wR = 0.0446 [$I_o > 2\sigma(I_o)$], R = 0.0298 and wR = 0.0471 (all reflections), 0.521<dΔρ < -0.517 eÅ^{-3}.

28. Complex oDIB-25

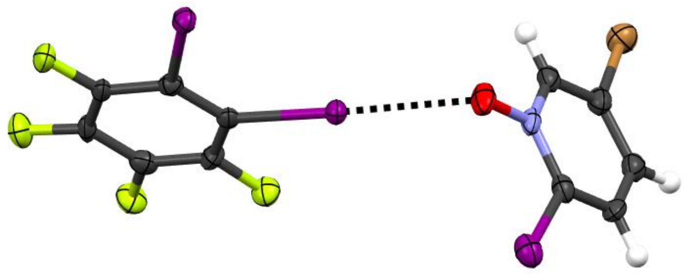

Figure S39. The X-ray crystal structure of **oDIB-25** with the thermal displacement parameter at 50% probability level. The black broken line represents the halogen bond. Colour Key: green = fluorine, purple = iodine, blue = nitrogen, dark grey = carbon, white = hydrogen. **Notes**: The asymmetric unit consists of one **oDIB** and one PyNO molecule.

Crystal data for oDIB-25 (crystallization solvent: $CHCl_3$): CCDC-2337259, $C_{11}H_3BrF_4I_3NO$, M = 701.75 gmol^{-1}, colourless block, 0.20 × 0.12 × 0.04 mm, triclinic, space group P-1, a = 7.4577(4) Å, b = 8.3647(6) Å, c = 13.1105(10) Å, α = 75.980(6)°, β = 80.742(6)°, γ = 86.943(5)°, V = 783.07(10) Å^3, Z = 2, D_{calc} = 2.976 gcm^{-3}, F(000) = 628, μ = 8.576 mm^{-1}, T = 123 K, θ_{max} = 25.249°, 4825 total reflections, 2498 with $I_o > 2\sigma(I_o)$, R_{int} = 0.0240, 2829 data, 190 parameters, 0 restraints, GooF = 1.042, R = 0.0351 and wR = 0.0807 [$I_o > 2\sigma(I_o)$], R = 0.0412 and wR = 0.0852 (all reflections), 3.670<dΔρ < -1.039 eÅ^{-3}.

29. Complex oDIB-26

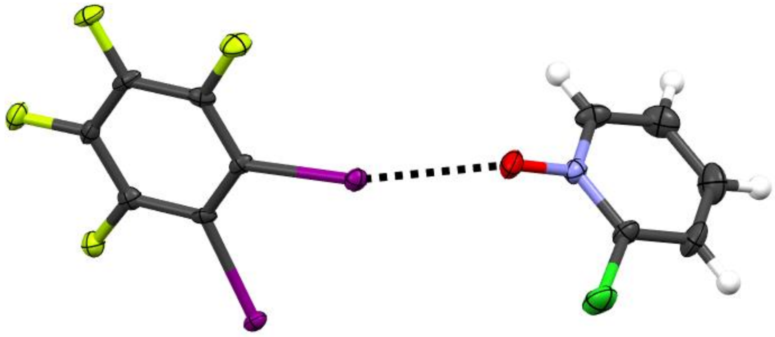

Figure S40. The X-ray crystal structure of **oDIB-26** with the thermal displacement parameter at 50% probability level. The black broken line represents the halogen bond and red broken line represents the hydrogen bond. Colour Key: green = fluorine, purple = iodine, blue = nitrogen, dark grey = carbon, white = hydrogen. **Notes**: The asymmetric unit consists of two **oDIB** molecules and two PyNO molecules. For viewing clarity, just the 1:1 donor:acceptor ratio complex is shown.

Crystal data for oDIB-26 (crystallization solvent: CH_2Cl_2): CCDC-2337260, $C_{11}H_4ClF_4I_2NO$, M = 531.40 gmol^{-1}, colourless block, 0.127 × 0.067 × 0.056 mm, triclinic, space group *P-1*, a = 7.45050(10) Å, b = 13.2565(2) Å, c = 15.1037(2) Å, α = 103.1070(10)°, β = 97.5710(10)°, γ = 103.6800(10)°, V = 1384.32(3)Å^3, Z = 4, D_{calc} = 2.550 gcm^{-3}, F(000) = 976, μ = 37.881 mm^{-1}, T = 120 K, θ_{max} = 66.743°, 23960 total reflections, 4689 with $I_o > 2\sigma(I_o)$, R_{int} = 0.0401, 4895 data, 361 parameters, 0 restraints, GooF = 1.064, R = 0.0239 and wR = 0.0585 [$I_o > 2\sigma(I_o)$], R = 0.0253 and wR = 0.0592 (all reflections), 1.078<dΔρ <-1.065 eÅ^{-3}.

30. Complex oDIB-27

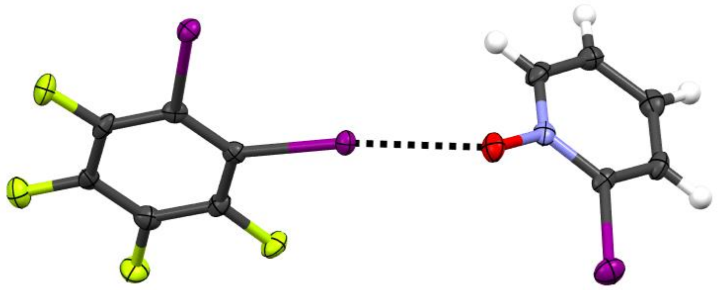

Figure S41. The X-ray crystal structure of **oDIB-27** with the thermal displacement parameter at 50% probability level. The black broken line represents the halogen bond. Colour Key: green = fluorine, purple = iodine, blue = nitrogen, dark grey = carbon, white = hydrogen. **Notes**: The asymmetric unit consists of one **oDIB** and one PyNO molecule.

Crystal data for oDIB-27 (crystallization solvent: $CHCl_3$): CCDC-2337261, $C_{11}H_4F_4I_3NO$, M = 622.85 gmol^{-1}, colourless block, 0.133 × 0.101 × 0.031 mm, triclinic, space group *P-1*, a = 7.6179(5) Å, b = 8.1223(5) Å, c = 12.6777(6) Å, α = 105.552(4)°, β = 102.470(5)°, γ = 92.072(5)°, V = 734.18(7) Å^3, Z = 2, D_{calc} = 2.817 gcm^{-3}, F(000) = 560, μ = 6.423 mm^{-1}, T = 123 K, θ_{max} = 25.250°, 4505 total reflections, 2411 with $I_o > 2\sigma(I_o)$, R_{int} = 0.0216, 2651 data, 181 parameters, 0 restraints, GooF = 1.021, R = 0.0228 and wR = 0.0435 [$I_o > 2\sigma(I_o)$], R = 0.0259 and wR = 0.0451 (all reflections), 0.615<dΔρ <-0.778 eÅ^{-3}.

31. Complex oDIB-28

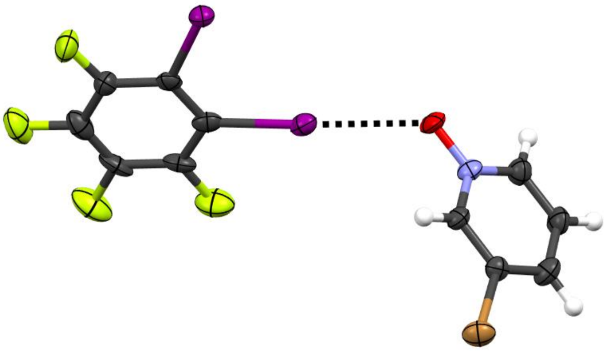

Figure S42. The X-ray crystal structure of **oDIB-28** with the thermal displacement parameter at 50% probability level. The black broken line represents the halogen bond and red broken line represents the hydrogen bond. Colour Key: green = fluorine, purple = iodine, blue = nitrogen, dark grey = carbon, white = hydrogen. **Notes**: The asymmetric unit consists of one **oDIB** molecule and three PyNO molecules. For viewing clarity, just the 1:1 donor:acceptor ratio complex is shown.

Crystal data for oDIB-28 (crystallization solvent: $CHCl_3$): CCDC-2337262, $C_{21}H_{12}Br_3F_4I_2N_3O_3$, M = 923.87 gmol^{-1}, colourless block, 0.21 × 0.06 × 0.05 mm, monoclinic, space group *P2₁/n*, a = 7.8030(2) Å, b = 22.3895(6) Å, c = 14.8027(3) Å, α = 90°, β = 94.396(2)°, γ = 90°, V = 2578.50(11) Å^3, Z = 4, D_{calc} = 2.380 gcm^{-3}, F(000) = 1720, μ = 7.147 mm^{-1}, T = 103 K, θ_{max} = 28.856°, 13013 total reflections, 4277 with $I_o > 2\sigma(I_o)$, R_{int} = 0.0586, 6720 data, 325 parameters, 0 restraints, GooF = 1.021, R = 0.0523 and wR = 0.0746 [$I_o > 2\sigma(I_o)$], R = 0.1037 and wR = 0.0856 (all reflections), 0.789<dΔρ <-0.795 eÅ^{-3}.

32. Complex oDIB-31

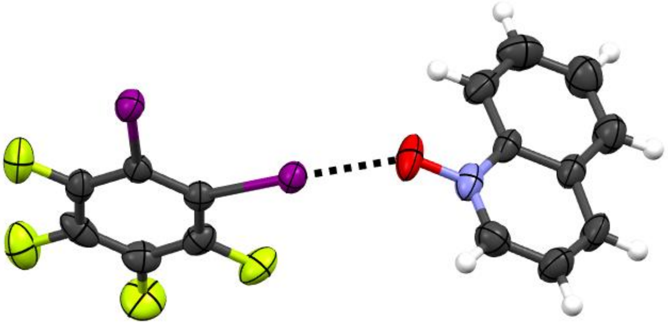

Figure S43. The X-ray crystal structure of **oDIB-31** with the thermal displacement parameter at 50% probability level. The black broken line represents the halogen bond. Colour Key: green = fluorine, purple = iodine, blue = nitrogen, dark grey = carbon, white = hydrogen. **Notes**: The asymmetric unit consists of one **oDIB** and one PyNO molecule.

Crystal data for oDIB-31 (crystallization solvent: $CHCl_3$): CCDC-2337263, $C_{15}H_7F_4I_2NO$, M = 547.02 gmol^{-1}, colourless block, 0.23 × 0.18 × 0.11 mm, monoclinic, space group *P2$_1$/c*, a = 7.8698(16) Å, b = 25.074(5) Å, c = 8.0337(16) Å, α = 90°, β = 90.32(3)°, γ = 90°, V = 1585.2(6) Å^3, Z = 4, D_{calc} = 2.292 gcm^{-3}, F(000) = 1016, μ = 4.011 mm^{-1}, T = 170 K, θ_{max} = 25.247°, 13481 total reflections, 2258 with $I_o > 2\sigma(I_o)$, R_{int} = 0.0489, 2872 data, 208 parameters, 0 restraints, GooF = 1.079, R = 0.0349 and wR = 0.0620 [$I_o > 2\sigma(I_o)$], R = 0.0551 and wR = 0.0672 (all reflections), 0.843<dΔρ < -0.503 eÅ^{-3}.

33. Complex oDIB-32

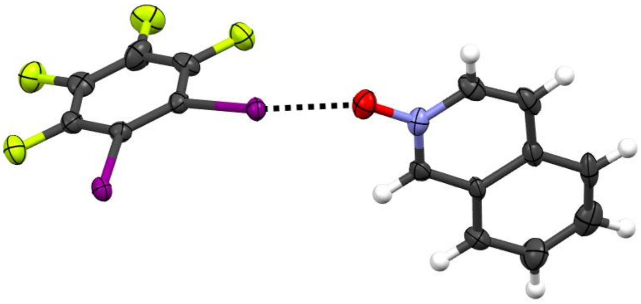

Figure S44. The X-ray crystal structure of **oDIB-32** with the thermal displacement parameter at 50% probability level. The black broken line represents the halogen bond. Colour Key: green = fluorine, purple = iodine, blue = nitrogen, dark grey = carbon, white = hydrogen. **Notes**: The asymmetric unit consists of one **oDIB** and one PyNO molecule.

Crystal data for oDIB-32 (crystallization solvent: $CHCl_3$): CCDC-2337264, $C_{15}H_7F_4I_2NO$, M = 547.02 gmol^{-1}, colourless plate, 0.01 × 0.09 × 0.04 mm, triclinic, space group *P-1*, a = 7.5407(4) Å, b = 8.2700(4) Å, c = 13.1339(7) Å, α = 101.991(4)°, β = 97.552(4)°, γ = 92.600(4)°, V = 791.99(7) Å^3, Z = 2, D_{calc} = 2.294 gcm^{-3}, F(000) = 508, μ = 31.622 mm^{-1}, T = 170 K, θ_{max} = 66.723°, 4286 total reflections, 2381 with $I_o > 2\sigma(I_o)$, R_{int} = 0.0458, 2765 data, 208 parameters, 0 restraints, GooF = 1.050, R = 0.0573 and wR = 0.1387 [$I_o > 2\sigma(I_o)$], R = 0.0631 and wR = 0.1471 (all reflections), 4.102<dΔρ < -1.902 eÅ^{-3}.

2.5. mDIB donor and mDIB-PyNO halogen-bonded complexes

1. Donor mDIB

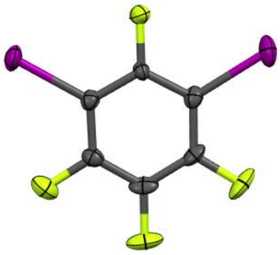

Figure S45. The X-ray crystal structure of **mDIB** with the thermal displacement parameter at 50% probability level. Colour Key: green = fluorine, purple = iodine, dark grey = carbon. **Notes**: The asymmetric unit consists of three **mDIB** molecules.

Crystal data for mDIB (crystallization solvent: commercial source): CCDC-2337518, $C_6F_4I_2$, M = 401.86 gmol^{-1}, colourless block, 0.13 × 0.06 × 0.05 mm, monoclinic, space group *P2₁/n*, a = 16.8549(4) Å, b = 8.7979(3) Å, c = 17.6708(4) Å, α = 90°, β = 90.722(2)°, γ = 90°, V = 2620.15(12) Å^3, Z = 12, D_{calc} = 3.056 gcm^{-3}, F(000) = 2136, μ = 56.759 mm^{-1}, T = 120(1) K, θ_{max} = 79.623°, 9835 total reflections, 8884 with $I_o > 2\sigma(I_o)$, R_{int} = twin, 9835 data, 326 parameters, 0 restraints, GooF = 1.052, R = 0.0754 and wR = 0.2127 [$I_o > 2\sigma(I_o)$], R = 0.0793 and wR = 0.2186 (all reflections), 2.023 <dΔρ < -3.199 eÅ^{-3}.

2. Complex mDIB-1

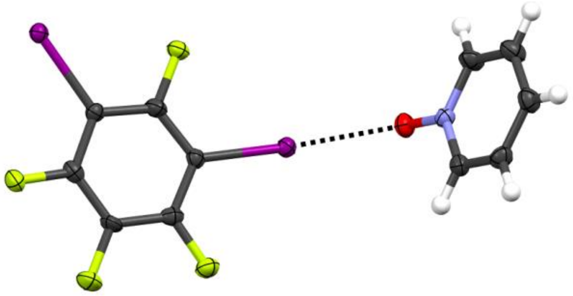

Figure S46. The X-ray crystal structure of **mDIB-1** with the thermal displacement parameter at 50% probability level. The black broken line represents the halogen bond. Colour Key: green = fluorine, purple = iodine, blue = nitrogen, dark grey = carbon, white = hydrogen. **Notes**: The asymmetric unit consists of one **mDIB** molecule, one PyNO molecule and one water molecule.

Crystal data for mDIB-1 (crystallization solvent: acetone): CCDC-2337501, $C_{16}H_{12}F_4I_2N_2O_3$, M = 610.08 gmol^{-1}, colourless plate, 0.21 × 0.16 × 0.03 mm, monoclinic, space group *C2/c*, a = 14.1341(3) Å, b = 18.1278(5) Å, c = 7.2867(2) Å, α = 90°, β = 93.565(2)°, γ = 90°, V = 1863.39(8) Å^3, Z = 4, D_{calc} = 2.175 gcm^{-3}, F(000) = 1152, μ = 27.068 mm^{-1}, T = 120(1) K, θ_{max} = 66.195°, 5993 total reflections, 1539 with $I_o > 2\sigma(I_o)$, R_{int} = 0.0644, 1618 data, 121 parameters, 0 restraints, GooF = 1.089, R = 0.0335 and wR = 0.0860 [$I_o > 2\sigma(I_o)$], R = 0.0379 and wR = 0.0916 (all reflections), 0.806 <dΔρ < -0.972 eÅ^{-3}.

3. Complex mDIB-2

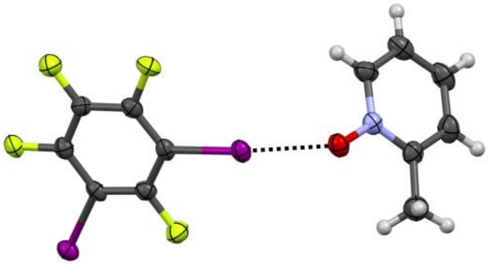

Figure S47. The X-ray crystal structure of **mDIB-2** with the thermal displacement parameter at 50% probability level. The black broken line represents the halogen bond. Colour Key: green = fluorine, purple = iodine, blue = nitrogen, dark grey = carbon, white = hydrogen. **Notes**: The asymmetric unit consists of one **mDIB** molecule, two PyNO molecules and one water molecule. For viewing clarity, just the 1:1 donor:acceptor ratio complex is shown.

Crystal data for mDIB-2 (crystallization solvent: acetone): CCDC-2337502, $C_{18}H_{16}F_4I_2N_2O_3$, M = 638.13 gmol^{-1}, colourless block, 0.09 × 0.04 × 0.03 mm, triclinic, space group *P-1*, a = 7.2238(2) Å, b = 12.5075(5) Å, c = 13.0724(4) Å, α = 104.221(3)°, β = 104.717(2)°, γ = 104.716(3)°, V = 1042.38(6) Å^3, Z = 2, D_{calc} = 2.033 gcm^{-3}, F(000) = 608, μ = 24.229 mm^{-1}, T = 120(1) K, θ_{max} = 79.294°, 7966 total reflections, 6822 with $I_o > 2\sigma(I_o)$, R_{int} = twin, 7966 data, 268 parameters, 0 restraints, GooF = 1.021, R = 0.0605 and wR = 0.1550 [$I_o > 2\sigma(I_o)$], R = 0.0677 and wR = 0.1619 (all reflections), 2.940 <dΔρ < -2.070 eÅ^{-3}.

4. Complex mDIB-3

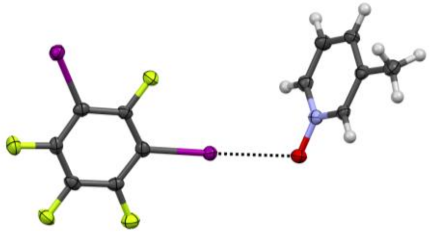

Figure S48. The X-ray crystal structure of **mDIB-3** with the thermal displacement parameter at 50% probability level. The black broken line represents the halogen bond. Colour Key: green = fluorine, purple = iodine, blue = nitrogen, dark grey = carbon, white = hydrogen. **Notes**: The asymmetric unit consists of two **mDIB** molecules and three PyNO molecules. For viewing clarity, just the 1:1 donor:acceptor ratio complex is shown.

Crystal data for mDIB-3 (crystallization solvent: acetone): CCDC-2337503 $C_{30}H_{21}F_8I_4N_3O_3$, M = 1131.10 gmol^{-1}, colourless block, 0.11 × 0.1 × 0.07 mm, triclinic, space group *P-1*, a = 8.27570(10) Å, b = 13.0277(2) Å, c = 16.6523(2) Å, α = 92.9880(10)°, β = 100.5830(10)°, γ = 100.3240(10)°, V = 1729.50(4) Å^3, Z = 2, D_{calc} = 2.172 gcm^{-3}, F(000) = 1060, μ = 29.017 mm^{-1}, T = 120(1) K, θ_{max} = 66.749°, 30876 total reflections, 5826 with $I_o > 2\sigma(I_o)$, R_{int} = 0.0490, 6127 data, 436 parameters, 0 restraints, GooF = 1.077, R = 0.0286 and wR = 0.0708 [$I_o > 2\sigma(I_o)$], R = 0.0302 and wR = 0.0717 (all reflections), 1.001 <dΔρ < -0.938 eÅ^{-3}.

5. Complex mDIB-4

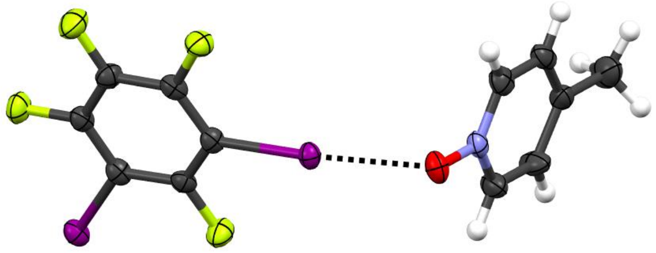

Figure S49. The X-ray crystal structure of **mDIB-4** with the thermal displacement parameter at 50% probability level. The black broken line represents the halogen bond. Colour Key: green = fluorine, purple = iodine, blue = nitrogen, dark grey = carbon, white = hydrogen. **Notes**: The asymmetric unit consists of two **mDIB** molecules and two PyNO molecules. For viewing clarity, just the 1:1 donor:acceptor ratio complex is shown.

Crystal data for mDIB-4 (crystallization solvent: acetone): CCDC-2337504, $C_{12}H_7F_4I_2NO$, M = 510.99 gmol^{-1}, colourless needle, 0.07 × 0.05 × 0.03 mm, triclinic, space group *P-1*, a = 7.5318(2) Å, b = 13.0077(2) Å, c = 15.7375(2) Å, α = 93.4850(10)°, β = 98.1080(10)°, γ = 95.225(2)°, V = 1515.82(5) Å^3, Z = 4, D_{calc} = 2.239 gcm^{-3}, F(000) = 944, μ = 32.973 mm^{-1}, T = 120(1) K, θ_{max} = 66.730°, 23308 total reflections, 4862 with $I_o > 2\sigma(I_o)$, R_{int} = 0.0604, 5348 data, 363 parameters, 0 restraints, GooF = 1.026, R = 0.0410 and wR = 0.1019 [$I_o > 2\sigma(I_o)$], R = 0.0448 and wR = 0.1045 (all reflections), 2.701 <dΔρ < -1.402 eÅ^{-3}.

6. Complex mDIB-5

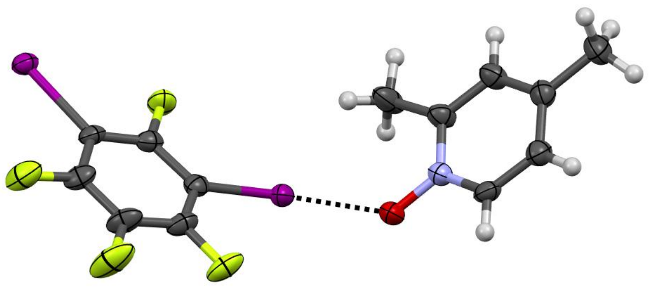

Figure S50. The X-ray crystal structure of **mDIB-5** with the thermal displacement parameter at 50% probability level. The black broken line represents the halogen bond. Colour Key: green = fluorine, purple = iodine, blue = nitrogen, dark grey = carbon, white = hydrogen. **Notes**: The asymmetric unit consists of one **mDIB** molecule and one PyNO molecule.

Crystal data for mDIB-5 (crystallization solvent: acetone): CCDC-2337505, $C_{13}H_9F_4I_2NO$, M = 525.01 gmol^{-1}, colourless block, 0.06 × 0.03 × 0.02 mm, monoclinic, space group *P2₁/c*, a = 9.8475(3) Å, b = 8.2581(2) Å, c = 20.0183(6) Å, α = 90°, β = 101.192(3)°, γ = 90°, V = 1596.96(8) Å^3, Z = 4, D_{calc} = 2.184 gcm^{-3}, F(000) = 976, μ = 31.320 mm^{-1}, T = 120(1) K, θ_{max} = 66.730°, 14831 total reflections, 2530 with $I_o > 2\sigma(I_o)$, R_{int} = 0.0469, 2829 data, 192 parameters, 0 restraints, GooF = 1.067, R = 0.0321 and wR = 0.0817 [$I_o > 2\sigma(I_o)$], R = 0.0361 and wR = 0.0835 (all reflections), 1.602 <dΔρ < -0.678 eÅ^{-3}.

7. Complex mDIB-6

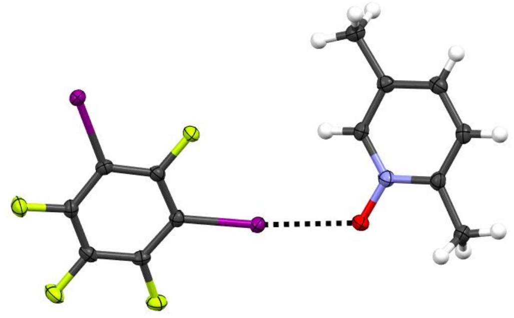

Figure S51. The X-ray crystal structure of **mDIB-6** with the thermal displacement parameter at 50% probability level. The black broken line represents the halogen bond. Colour Key: green = fluorine, purple = iodine, blue = nitrogen, dark grey = carbon, white = hydrogen. **Notes**: The asymmetric unit consists of one 0.5 **mDIB** molecule, and one PyNO molecule.

Crystal data for mDIB-6 (crystallization solvent: acetone): CCDC-2337506, $C_{20}H_{18}F_4I_2N_2O_2$, M = 648.16 gmol^{-1}, colourless block, 0.128 × 0.109 × 0.094 mm, monoclinic, space group *I2/a*, a = 7.20550(5) Å, b = 11.29821(7) Å, c = 25.83585(16) Å, α = 90°, β = 92.7282(6)°, γ = 90°, V = 2100.89(2) Å^3, Z = 4, D_{calc} = 2.049 gcm^{-3}, F(000) = 1240, μ = 24.019 mm^{-1}, T = 120(1) K, θ_{max} = 66.707°, 12837 total reflections, 1845 with $I_o > 2\sigma(I_o)$, R_{int} = 0.0369, 1867 data, 140 parameters, 0 restraints, GooF = 1.096, R = 0.0178 and wR = 0.0452 [$I_o > 2\sigma(I_o)$], R = 0.0180 and wR = 0.0452 (all reflections), 0.295<dΔρ < -0.574 eÅ^{-3}.

8. Complex mDIB-7

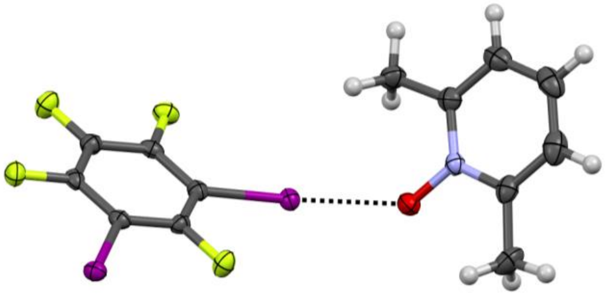

Figure S52. The X-ray crystal structure of **mDIB-7** with the thermal displacement parameter at 50% probability level. The black broken line represents the halogen bond. Colour Key: green = fluorine, purple = iodine, blue = nitrogen, dark grey = carbon, white = hydrogen. **Notes**: The asymmetric unit consists of one 0.5 **mDIB** molecule, one PyNO molecule, and one 0.5 water molecule. For viewing clarity, just the 1:1 donor:acceptor ratio complex is shown.

Crystal data for mDIB-7 (crystallization solvent: acetone): CCDC-2337507, $C_{20}H_{20}F_4I_2N_2O_3$, M = 666.18 gmol^{-1}, colourless block, 0.1 × 0.04 × 0.03 mm, monoclinic, space group *I2/a*, a = 7.36560(10) Å, b = 21.0500(3) Å, c = 14.5570(2) Å, α = 90°, β = 95.3880(10)°, γ = 90°, V = 2247.03(5) Å^3, Z = 4, D_{calc} = 1.969 gcm^{-3}, F(000) = 1280, μ = 22.512 mm^{-1}, T = 120(1) K, θ_{max} = 66.684°, 11117 total reflections, 1883 with $I_o > 2\sigma(I_o)$, R_{int} = 0.0622, 1999 data, 148 parameters, 0 restraints, GooF = 1.115, R = 0.0292 and wR = 0.0763 [$I_o > 2\sigma(I_o)$], R = 0.0305 and wR = 0.0771 (all reflections), 1.038<dΔρ < -1.369 eÅ^{-3}.

9. Complex mDIB-8

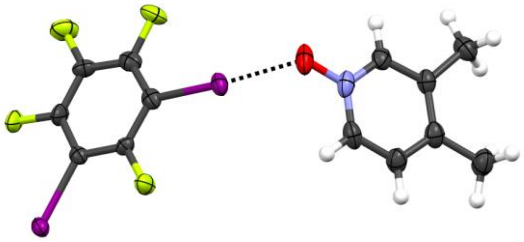

Figure S53. The X-ray crystal structure of **mDIB-8** with the thermal displacement parameter at 50% probability level. The black broken line represents the halogen bond. Colour Key: green = fluorine, purple = iodine, blue = nitrogen, dark grey = carbon, white = hydrogen. **Notes**: The asymmetric unit consists of one **mDIB** molecule, and two PyNO molecules. For viewing clarity, just the 1:1 donor:acceptor ratio complex is shown.

Crystal data for mDIB-8 (crystallization solvent: $CHCl_3$): CCDC-2337508, $C_{20}H_{18}F_4I_2N_2O_2$, M = 648.16 gmol^{-1}, colourless block, 0.06 × 0.04 × 0.04 mm, monoclinic, space group P*2₁/c*, a = 14.6704(2) Å, b = 19.1122(3) Å, c = 7.82700(10) Å, α = 90°, β = 92.3870(10)°, γ = 90°, V = 2192.66(5) Å^3, Z = 4, D_{calc} = 1.963 gcm^{-3}, F(000) = 1240, μ = 23.014 mm^{-1}, T = 120(1) K, θ_{max} = 66.741°, 24724 total reflections, 3653 with $I_o > 2\sigma(I_o)$, R_{int} = 0.0563, 3895 data, 269 parameters, 0 restraints, GooF = 1.059, R = 0.0513 and wR = 0.1285 [$I_o > 2\sigma(I_o)$], R = 0.0539 and wR = 0.1306 (all reflections), 4.499<dΔρ < -1.429 eÅ^{-3}.

10. Complex mDIB-9

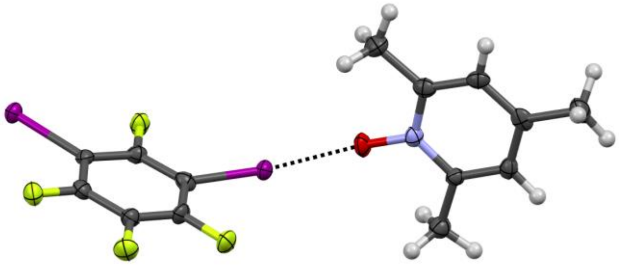

Figure S54. The X-ray crystal structure of **mDIB-9** with the thermal displacement parameter at 50% probability level. The black broken line represents the halogen bond. Colour Key: green = fluorine, purple = iodine, blue = nitrogen, dark grey = carbon, white = hydrogen. **Notes**: The asymmetric unit consists of one **mDIB** molecule, and two PyNO molecules. For viewing clarity, just the 1:1 donor:acceptor ratio complex is shown.

Crystal data for mDIB-9 (crystallization solvent: acetone): CCDC-2337509, $C_{20}H_{11}F_8I_4NO$, M = 940.90 gmol^{-1}, colourless block, 0.05 × 0.04 × 0.03 mm, monoclinic, space group C*2/c*, a = 36.6769(5) Å, b = 7.72810(10) Å, c = 18.0529(3) Å, α = 90°, β = 97.8430(10)°, γ = 90°, V = 5069.10(13) Å^3, Z = 8, D_{calc} = 2.466 gcm^{-3}, F(000) = 3440, μ = 39.307 mm^{-1}, T = 120(1) K, θ_{max} = 66.750°, 30954 total reflections, 3787 with $I_o > 2\sigma(I_o)$, R_{int} = 0.0813, 4489 data, 310 parameters, 0 restraints, GooF = 1.021, R = 0.0303 and wR = 0.0661 [$I_o > 2\sigma(I_o)$], R = 0.0396 and wR = 0.0691 (all reflections), 0.678<dΔρ < -1.290 eÅ^{-3}.

11. Complex mDIB-11

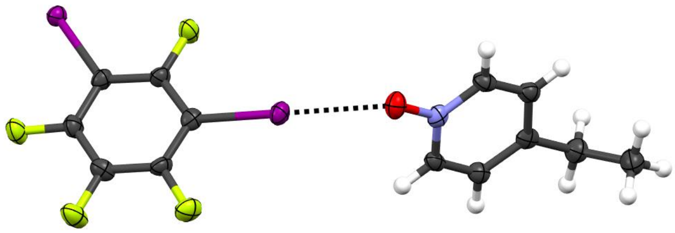

Figure S55. The X-ray crystal structure of **mDIB-11** with the thermal displacement parameter at 50% probability level. The black broken line represents the halogen bond. Colour Key: green = fluorine, purple = iodine, blue = nitrogen, dark grey = carbon, white = hydrogen. **Notes**: The asymmetric unit consists of two **mDIB** molecules and two PyNO molecules. For viewing clarity, just the 1:1 donor:acceptor ratio complex is shown.

Crystal data for mDIB-11 (crystallization solvent: $CHCl_3$): CCDC-2337510, $C_{13}H_9F_4I_2NO$, M = 525.01 gmol^{-1}, colourless block, 0.021 × 0.013 × 0.011 mm, triclinic, space group P-1, a = 7.5931(2) Å, b = 13.8297(3) Å, c = 15.5472(4) Å, α = 88.749(2)°, β = 77.674(2)°, γ = 76.480(2)°, V = 1550.09(7) Å^3, Z = 4, D_{calc} = 2.250 gcm^{-3}, F(000) = 976, μ = 32.267 mm^{-1}, T = 120(1) K, θ_{max} = 66.745°, 20486 total reflections, 4737 with $I_o > 2\sigma(I_o)$, R_{int} = 0.0388, 5480 data, 381 parameters, 0 restraints, GooF = 1.012, R = 0.0337 and wR = 0.0901 [$I_o > 2\sigma(I_o)$], R = 0.0400 and wR = 0.0933 (all reflections), 1.352<dΔρ < -1.175 eÅ^{-3}.

12. Complex mDIB-15

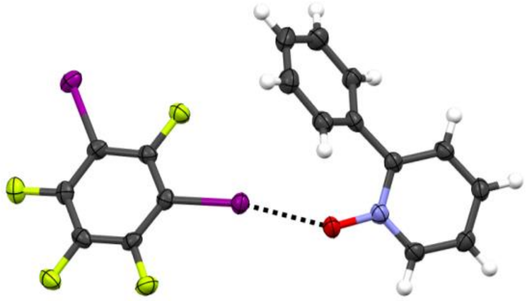

Figure S56. The X-ray crystal structure of **mDIB-15** with the thermal displacement parameter at 50% probability level. The black broken line represents the halogen bond. Colour Key: green = fluorine, purple = iodine, blue = nitrogen, dark grey = carbon, white = hydrogen. **Notes**: The asymmetric unit consists of one **mDIB** molecule and one PyNO molecule.

Crystal data for mDIB-15 (crystallization solvent: $CHCl_3$): CCDC-2337511, $C_{17}H_9F_4I_2NO$, M = 573.05 gmol^{-1}, colourless block, 0.22 × 0.05 × 0.04 mm, triclinic, space group *P-1*, a = 6.0279(2) Å, b = 8.6511(3) Å, c = 17.4905(5)Å, α = 79.328(3)°, β = 89.109(3)°, γ = 77.652(3)°, V = 875.31(5) Å^3, Z = 2, D_{calc} = 2.174 gcm^{-3}, F(000) = 536, μ = 28.653 mm^{-1}, T = 120(1) K, θ_{max} = 66.731°, 12242 total reflections, 2870 with $I_o > 2\sigma(I_o)$, R_{int} = 0.0404, 3090 data, 226 parameters, 0 restraints, GooF = 1.049, R = 0.0282 and wR = 0.0763 [$I_o > 2\sigma(I_o)$], R = 0.0303 and wR = 0.0777 (all reflections), 1.033<dΔρ < -0.863 eÅ^{-3}.

13. Complex mDIB-19

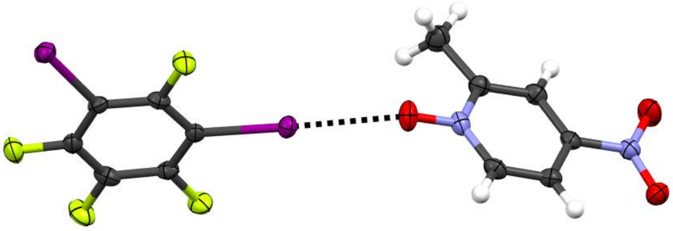

Figure S57. The X-ray crystal structure of **mDIB-19** with the thermal displacement parameter at 50% probability level. The black broken line represents the halogen bond. Colour Key: green = fluorine, purple = iodine, blue = nitrogen, dark grey = carbon, white = hydrogen. **Notes**: The asymmetric unit consists of two **mDIB** molecule and one PyNO molecule. For viewing clarity, just the 1:1 donor:acceptor ratio complex is shown.

Crystal data for mDIB-19 (crystallization solvent: $CHCl_3$): CCDC-2337512, $C_{18}H_6F_8I_4N_2O_3$, M = 957.85 gmol^{-1}, colourless block, 0.1 × 0.04 × 0.03 mm, triclinic, space group *P-1*, a = 9.2811(2) Å, b = 11.5319(3) Å, c = 11.7259(2) Å, α = 81.828(2)°, β = 88.173(2)°, γ = 78.317(2)°, V = 1216.52(5) Å^3, Z = 2, D_{calc} = 2.615 gcm^{-3}, F(000) = 872, μ = 41.046 mm^{-1}, T = 120(1) K, θ_{max} = 66.724°, 19086 total reflections, 4125 with $I_o > 2\sigma(I_o)$, R_{int} = 0.0317, 4297 data, 317 parameters, 0 restraints, GooF = 1.043, R = 0.0246 and wR = 0.0610 [$I_o > 2\sigma(I_o)$], R = 0.0257 and wR = 0.0616 (all reflections), 1.082<dΔρ < -1.597eÅ^{-3}.

14. Complex mDIB-21

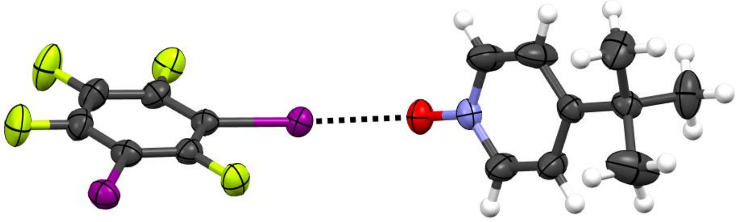

Figure S58. The X-ray crystal structure of **mDIB-21** with the thermal displacement parameter at 50% probability level. The black broken line represents the halogen bond. Colour Key: green = fluorine, purple = iodine, blue = nitrogen, dark grey = carbon, white = hydrogen. **Notes**: The asymmetric unit consists of one **mDIB** molecule and one PyNO molecule.

Crystal data for mDIB-21 (crystallization solvent: $CHCl_3$): CCDC-2337513, $C_{15}H_{13}F_4I_2NO$, M = 553.06 gmol^{-1}, colourless block, 0.095 × 0.063 × 0.027 mm, monoclinic, space group *P2₁/c*, a = 9.3341(2) Å, b = 14.5878(3) Å, c = 12.8932(3) Å, α = 90°, β = 90.113(2)°, γ = 90°, V = 1755.59(7) Å^3, Z = 4, D_{calc} = 2.092 gcm^{-3}, F(000) = 1040, μ = 28.532 mm^{-1}, T = 120(1) K, θ_{max} = 66.746°, 22160 total reflections, 2948 with $I_o > 2\sigma(I_o)$, R_{int} = 0.0478, 3110 data, 211 parameters, 0 restraints, GooF = 1.071, R = 0.0370 and wR = 0.0981 [$I_o > 2\sigma(I_o)$], R = 0.0384 and wR = 0.0993 (all reflections), 2.035<dΔρ < -0.697eÅ^{-3}.

15. Complex mDIB-22

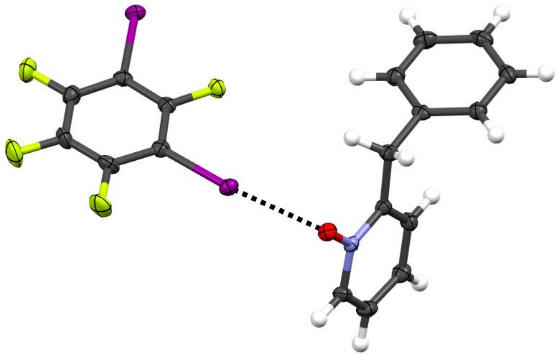

Figure S59. The X-ray crystal structure of **mDIB-22** with the thermal displacement parameter at 50% probability level. The black broken line represents the halogen bond. Colour Key: green = fluorine, purple = iodine, blue = nitrogen, dark grey = carbon, white = hydrogen. **Notes**: The asymmetric unit consists of one **mDIB** molecule and one PyNO molecule.

Crystal data for mDIB-22 (crystallization solvent: $CHCl_3$): CCDC-2337514, $C_{18}H_{11}F_4I_2NO$, M = 587.08 gmol^{-1}, colourless block, 0.11 × 0.06 × 0.06 mm, triclinic, space group *P-1*, a = 7.2141(2) Å, b = 10.4780(2) Å, c = 13.3498(3) Å, α = 103.821(2)°, β = 102.215(2)°, γ = 90.689(2)°, V = 955.62(4) Å^3, Z = 2, D_{calc} = 2.040 gcm^{-3}, F(000) = 552, μ = 26.265 mm^{-1}, T = 120(1) K, θ_{max} = 66.744°, 14016 total reflections, 3272 with $I_o > 2\sigma(I_o)$, R_{int} = 0.0259, 3392 data, 235 parameters, 0 restraints, GooF = 1.064, R = 0.0201 and wR = 0.0533 [$I_o > 2\sigma(I_o)$], R = 0.0210 and wR = 0.0539 (all reflections), 1.237<dΔρ < -0.453 eÅ^{-3}.

16. Complex mDIB-29

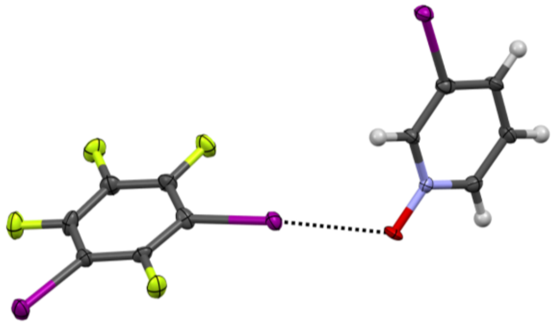

Figure S60. The X-ray crystal structure of **mDIB-29** with the thermal displacement parameter at 50% probability level. The black broken line represents the halogen bond. Colour Key: green = fluorine, purple = iodine, blue = nitrogen, dark grey = carbon, white = hydrogen. **Notes**: The asymmetric unit consists of two **mDIB** molecules and two PyNO molecules. For viewing clarity, just the 1:1 donor:acceptor ratio complex is shown.

Crystal data for mDIB-29 (crystallization solvent: $CHCl_3$): CCDC-2337515, $C_{11}H_4F_4I_3NO$, M = 622.85 gmol^{-1}, colourless plate, 0.14 × 0.11 × 0.03 mm, triclinic, space group *P-1*, a = 8.0836(2) Å, b = 8.4793(2) Å, c = 24.4881(4) Å, α = 88.648(2)°, β = 82.191(2)°, γ = 64.159(2)°, V = 1495.45(6) Å^3, Z = 4, D_{calc} = 2.766 gcm^{-3}, F(000) = 1120, μ = 49.633 mm^{-1}, T = 120(1) K, θ_{max} = 66.745°, 24804 total reflections, 4955 with $I_o > 2\sigma(I_o)$, R_{int} = 0.0893, 5304 data, 361 parameters, 0 restraints, GooF = 1.059, R = 0.0461 and wR = 0.1229 [$I_o > 2\sigma(I_o)$], R = 0.0481 and wR = 0.1248 (all reflections), 1.776<dΔρ < -2.276 eÅ^{-3}.

17. Complex mDIB-31

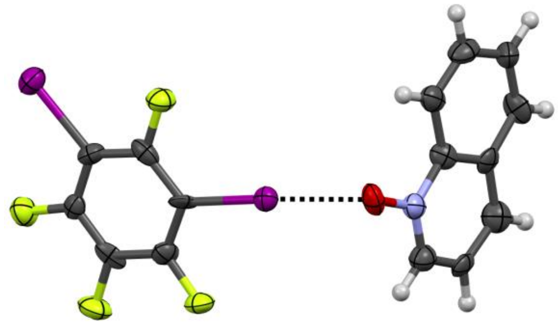

Figure S61. The X-ray crystal structure of **mDIB-31** with the thermal displacement parameter at 50% probability level. The black broken line represents the halogen bond. Colour Key: green = fluorine, purple = iodine, blue = nitrogen, dark grey = carbon, white = hydrogen. **Notes**: The asymmetric unit consists of one **mDIB** molecule and one PyNO molecule.

Crystal data for mDIB-31 (crystallization solvent: $CHCl_3$): CCDC-2337516, $C_{15}H_7F_4I_2NO$, M = 547.02 gmol^{-1}, colourless plate, 0.05 × 0.04 × 0.02 mm, orthorhombic, space group *Pbca*, a = 7.3052(12) Å, b = 17.596(3) Å, c = 23.819(3) Å, α = 90°, β = 90°, γ = 90°, V = 3061.8(8) Å^3, Z = 8, D_{calc} = 2.373 gcm^{-3}, F(000) = 2032, μ = 32.719 mm^{-1}, T = 120(1) K, θ_{max} = 66.726°, 16239 total reflections, 1884 with $I_o > 2\sigma(I_o)$, R_{int} = 0.1698, 2702 data, 208 parameters, 0 restraints, GooF = 1.023, R = 0.0679 and wR = 0.1614 [$I_o > 2\sigma(I_o)$], R = 0.1024 and wR = 0.1925 (all reflections), 2.116<dΔρ < -1.366 eÅ^{-3}.

18. Complex mDIB-32

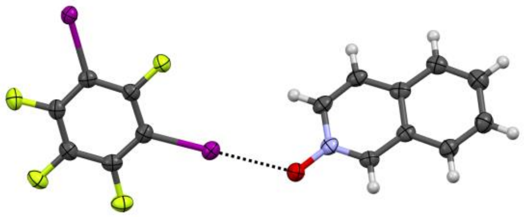

Figure S62. The X-ray crystal structure of **mDIB-32** with the thermal displacement parameter at 50% probability level. The black broken line represents the halogen bond. Colour Key: green = fluorine, purple = iodine, blue = nitrogen, dark grey = carbon, white = hydrogen. **Notes**: The asymmetric unit consists of one **mDIB** molecule and two PyNO molecules. For viewing clarity, just the 1:1 donor:acceptor ratio complex is shown.

Crystal data for mDIB-32 (crystallization solvent: acetone): CCDC-2337517, $C_{24}H_{14}F_4I_2N_2O_2$, M = 692.17 gmol^{-1}, colourless block, 0.13 × 0.07 × 0.05 mm, monoclinic, space group *P2₁/n*, a = 7.36980(10) Å, b = 15.0622(2) Å, c = 20.4609(3) Å, α = 90°, β = 91.5330(10)°, γ = 90°, V = 2270.46(5) Å^3, Z = 8, D_{calc} = 2.025 gcm^{-3}, F(000) = 1320, μ = 22.289 mm^{-1}, T = 120(1) K, θ_{max} = 66.725°, 22842 total reflections, 3797 with $I_o > 2\sigma(I_o)$, R_{int} = 0.0447, 4010 data, 307 parameters, 0 restraints, GooF = 1.073, R = 0.0323 and wR = 0.0806 [$I_o > 2\sigma(I_o)$], R = 0.0338 and wR = 0.0816 (all reflections), 1.165<dΔρ < -0.715 eÅ^{-3}.

2.6. pDIB-PyNO halogen-bonded complexes

1. Complex pDIB-1

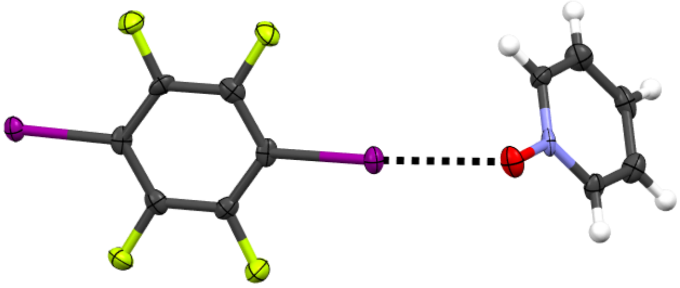

Figure S63. The X-ray crystal structure of **pDIB-1** with the thermal displacement parameter at 50% probability level. The black broken line represents the halogen bond. Colour Key: green = fluorine, purple = iodine, blue = nitrogen, dark grey = carbon, white = hydrogen. **Notes**: The asymmetric unit consists of one **pDIB** molecule, two PyNO molecules and one water molecule. For viewing clarity, just the 1:1 donor:acceptor ratio complex is shown.

Crystal data for pDIB-1 (crystallization solvent: $CHCl_3$): CCDC-2337658, $C_{16}H_{12}F_4I_2N_2O_3$, M = 610.08 gmol^{-1}, colourless plate, 0.320 × 0.141 × 0.012 mm, monoclinic, space group P2$_1$/c, a = 14.7306(7) Å, b = 18.1510(9)Å, c = 7.2857(3) Å, α = 90°, β = 100.138(5)°, γ = 90°, V = 1917.61(17) Å^3, Z = 8, D_{calc} = 2.113 gcm^{-3}, F(000) = 1152, μ = 3.336 mm^{-1}, T = 170 K, θ_{max} = 25.249°, 13366 total reflections, 2864 with $I_o > 2\sigma(I_o)$, R_{int} = 0.0559, 3474 data, 244 parameters, 1 restraints, GooF = 1.055, R = 0.0337 and wR = 0.0667 [$I_o > 2\sigma(I_o)$], R = 0.0458 and wR = 0.0749 (all reflections), 1.039<dΔρ <-0.897 eÅ^{-3}.

2. Complex pDIB-2

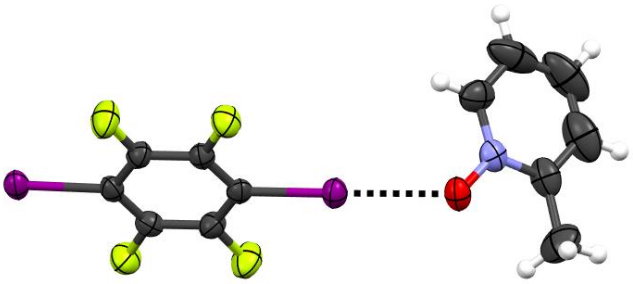

Figure S64. The X-ray crystal structure of **pDIB-2** with the thermal displacement parameter at 50% probability level. The black broken line represents the halogen bond. Colour Key: green = fluorine, purple = iodine, blue = nitrogen, dark grey = carbon, white = hydrogen. **Notes**: The asymmetric unit consists of one **pDIB** molecule and one PyNO molecule.

Crystal data for pDIB-2 (crystallization solvent: $CHCl_3$ or acetone): CCDC-2337659, $C_{12}H_7F_4I_2NO$, M = 510.99 gmol^{-1}, colourless block, 0.34 × 0.29 × 0.2 mm, orthorhombic, space group P*bca*, a = 19.231(4) Å, b = 7.4129(15) Å, c = 21.741(4) Å, α = 90°, β = 90°, γ = 90°, V = 3099.3(11) Å^3, Z = 8, D_{calc} = 2.190 gcm^{-3}, F(000) = 976, μ = 4.094 mm^{-1}, T = 170 K, θ_{max} = 25.249°, 26671 total reflections, 2202 with $I_o > 2\sigma(I_o)$, R_{int} = 0.0482, 2804 data, 182 parameters, 6 restraints, GooF = 1.067, R = 0.0249 and wR = 0.0444 [$I_o > 2\sigma(I_o)$], R = 0.0397 and wR = 0.0485 (all reflections), 0.381 <dΔρ < -0.334 eÅ^{-3}.

3. Complex pDIB-3

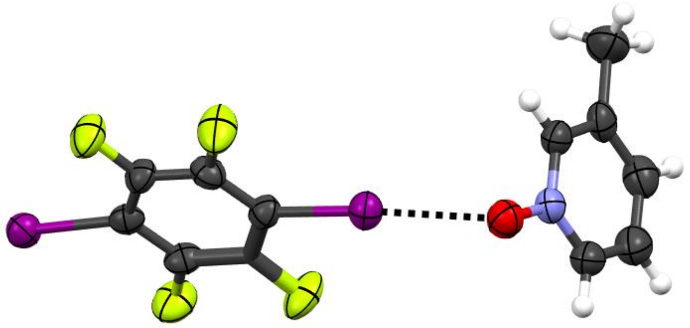

Figure S65. The X-ray crystal structure of **pDIB-3** with the thermal displacement parameter at 50% probability level. The black broken line represents the halogen bond. Colour Key: green = fluorine, purple = iodine, blue = nitrogen, dark grey = carbon, white = hydrogen. **Notes**: The asymmetric unit consists of one **pDIB** molecule and one PyNO molecule.

Crystal data for pDIB-3 (crystallization solvent: $CHCl_3$): CCDC-2337660, $C_{12}H_7F_4I_2NO$, M = 510.99 gmol^{-1}, colourless block, 0.36 × 0.28 × 0.09 mm, monoclinic, space group $P2_1/n$, a = 10.220(2) Å, b = 10.937(2) Å, c = 13.094(3) Å, α = 90°, β = 101.60(3)°, γ = 90°, V = 1433.8(5) Å^3, Z = 4, D_{calc} = 2.367 gcm^{-3}, F(000) = 944, μ = 4.425 mm^{-1}, T = 170 K, θ_{max} = 25.250°, 9588 total reflections, 2024 with $I_o > 2\sigma(I_o)$, R_{nt} = 0.0444, 2593 data, 182 parameters, 0 restraints, GooF = 1.030, R = 0.0344 and wR = 0.0587 [$I_o > 2\sigma(I_o)$], R = 0.0520 and wR = 0.0637 (all reflections), 0.568<dΔρ < -0.456 eÅ^{-3}.

4. Complex pDIB-4

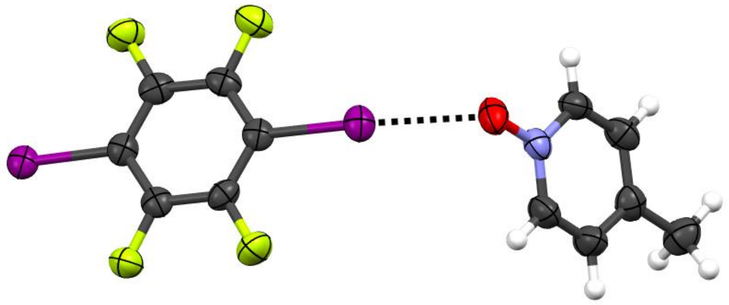

Figure S66. The X-ray crystal structure of **pDIB-4** with the thermal displacement parameter at 50% probability level. The black broken line represents the halogen bond. Colour Key: green = fluorine, purple = iodine, blue = nitrogen, dark grey = carbon, white = hydrogen. **Notes**: The asymmetric unit consists of one **pDIB** molecule and one PyNO molecule.

Crystal data for pDIB-4 (crystallization solvent: $CHCl_3$): CCDC-2337661, $C_{12}H_7F_4I_2NO$, M = 510.99 gmol^{-1}, colourless block, 0.36 × 0.33 × 0.18 mm, orthorhombic, space group P*bcn*, a = 18.571(4) Å, b = 7.8261(16) Å, c = 21.045(4) Å, α = 90°, β = 90°, γ = 90°, V = 3058.6(11) Å^3, Z = 4, D_{calc} = 2.219 gcm^{-3}, F(000) = 1888, μ = 4.148 mm^{-1}, T = 170 K, θ_{max} = 25.248 °, 25826 total reflections, 2339 with $I_o > 2\sigma(I_o)$, R_{int} = 0.0557, 2767 data, 182 parameters, 0 restraints, GooF = 1.071, R = 0.0299 and wR = 0.0515 [$I_o > 2\sigma(I_o)$], R = 0.0410 and wR = 0.0544 (all reflections), 0.589 <dΔρ < -0.484 eÅ^{-3}.

5. Complex pDIB-5

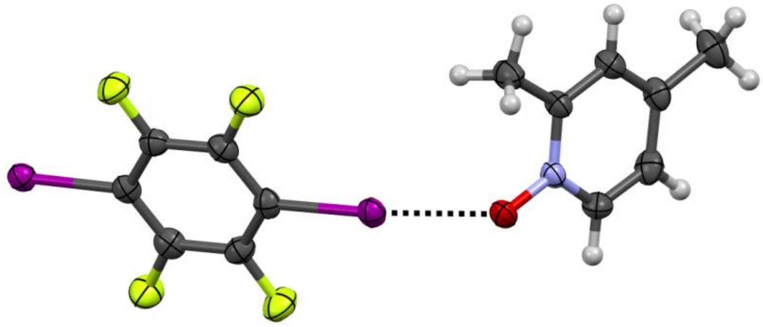

Figure S67. The X-ray crystal structure of **pDIB-5** with the thermal displacement parameter at 50% probability level. The black broken line represents the halogen bond. Colour Key: green = fluorine, purple = iodine, blue = nitrogen, dark grey = carbon, white = hydrogen. **Notes**: The asymmetric unit consists of two **pDIB** molecules and two PyNO molecules.

Crystal data for pDIB-5 (crystallization solvent: $CHCl_3$): CCDC-2337662, $C_{13}H_9F_4I_2NO$, M = 525.01 gmol^{-1}, colourless plate, 0.13 × 0.06 × 0.03 mm, monoclinic, space group P2$_1$/c, a = 19.8650(2) Å, b = 19.4487(2) Å, c = 8.43660(10) Å, α = 90°, β = 90.4670(10)°, γ = 90°, V = 3259.36(6) Å^3, Z = 8, D_{calc} = 2.140 gcm^{-3}, F(000) = 1952, μ = 30.692 mm^{-1}, T = 170 K, θ_{max} = 66.739°, 41378 total reflections, 5250 with $I_o > 2\sigma(I_o)$, R_{int} = 0.0596, 5773 data, 383 parameters, 0 restraints, GooF = 1.099, R = 0.0335 and wR = 0.0815 [$I_o > 2\sigma(I_o)$], R = 0.0374 and wR = 0.0837 (all reflections), 0.979<dΔρ < -0.803eÅ^{-3}.

6. Complex pDIB-6

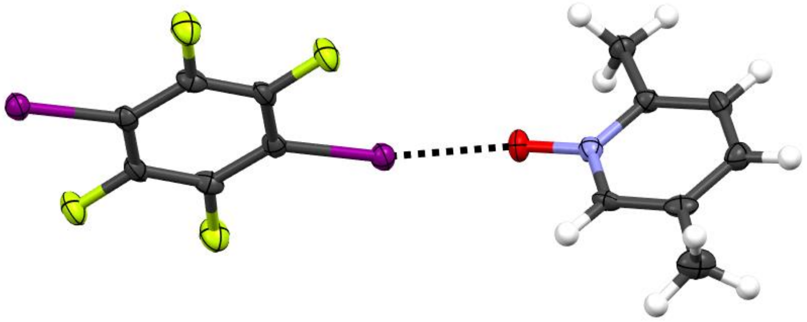

Figure S68. The X-ray crystal structure of **pDIB-6** with the thermal displacement parameter at 50% probability level. The black broken line represents the halogen bond. Colour Key: green = fluorine, purple = iodine, blue = nitrogen, dark grey = carbon, white = hydrogen. **Notes**: The asymmetric unit consists of two 0.5 **pDIB** molecules and one PyNO molecule.

Crystal data for pDIB-6 (crystallization solvent: $CHCl_3$): CCDC-2337663, $C_{13}H_9F_4I_2NO$, M = 525.01 gmol^{-1}, colourless plate, 0.14 × 0.08 × 0.04 mm, triclinic, space group P-1, a = 4.8069(3) Å, b = 11.5395(7) Å, c = 14.3345(8) Å, α = 88.989(5)°, β = 83.482(5)°, γ = 82.515(5)°, V = 783.25(8) Å^3, Z = 2, D_{calc} = 2.226 gcm^{-3}, F(000) = 488, μ = 4.053 mm^{-1}, T = 170 K, θ_{max} = 25.235 °, 4355 total reflections, 2465 with $I_o > 2\sigma(I_o)$, R_{int} = 0.0177, 2819 data, 192 parameters, 0 restraints, GooF = 1.018, R = 0.0227 and wR = 0.0390 [$I_o > 2\sigma(I_o)$], R = 0.0275 and wR = 0.0416 (all reflections), 0.479<dΔρ < -0.473 eÅ^{-3}.

7. Complex pDIB-7

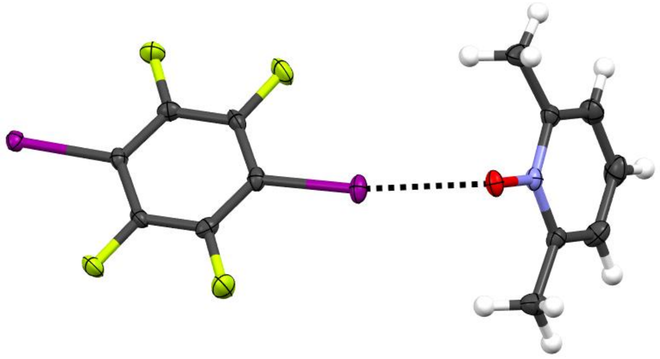

Figure S69. The X-ray crystal structure of **pDIB-7** with the thermal displacement parameter at 50% probability level. The black broken line represents the halogen bond. Colour Key: green = fluorine, purple = iodine, blue = nitrogen, dark grey = carbon, white = hydrogen. **Notes**: The asymmetric unit consists of two **pDIB** molecules and one PyNO molecule.

Crystal data for pDIB-7 (crystallization solvent: $CHCl_3$): CCDC-2337664, $C_{19}H_9F_8I_4NO$, M = 926.87 gmol^{-1}, colourless block, 0.11 × 0.11 × 0.1 mm, monoclinic, space group C2/c, a = 28.0488(5) Å, b = 7.56577(13) Å, c = 23.0582(4) Å, α = 90°, β = 95.8374(16)°, γ = 90°, V = 4867.84(14) Å^3, Z = 8, D_{calc} = 2.529 gcm^{-3}, F(000) = 488, μ = 5.195 mm^{-1}, T = 120 K, θ_{max} = 25.250 °, 11997 total reflections, 4161 with $I_o > 2\sigma(I_o)$, R_{int} = 0.0197, 4400 data, 300 parameters, 0 restraints, GooF = 1.150, R = 0.0205 and wR = 0.0447 [$I_o > 2\sigma(I_o)$], R = 0.0227 and wR = 0.0455 (all reflections), 1.010<dΔρ<-1.030 eÅ^{-3}.

8. Complex pDIB-8

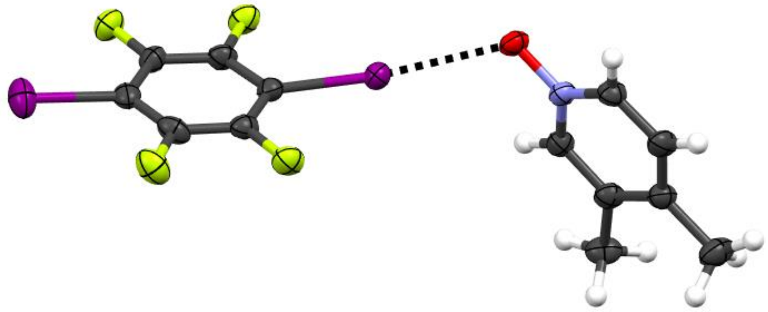

Figure S70. The X-ray crystal structure of **pDIB-8** with the thermal displacement parameter at 50% probability level. The black broken line represents the halogen bond. Colour Key: green = fluorine, purple = iodine, blue = nitrogen, dark grey = carbon, white = hydrogen. **Notes**: The asymmetric unit consists of two 0.5 **pDIB** molecules, one full **pDIB** molecule and two PyNO molecule. For viewing clarity, just the 1:1 donor:acceptor ratio complex is shown.

Crystal data for pDIB-8 (crystallization solvent: $CHCl_3$): CCDC-2337665, $C_{13}H_9F_4I_2NO$, M = 525.01 gmol^{-1}, colourless block, 0.06 × 0.05 × 0.04 mm, triclinic, space group P-1, a = 8.5223(3) Å, b = 14.4403(5) Å, c = 14.6243(4) Å, α = 108.996(3)°, β = 102.466(3)°, γ = 106.040(3)°, V = 1540.09(10) Å^3, Z = 4, D_{calc} = 2.264 gcm^{-3}, F(000) = 976, μ = 32.477 mm^{-1}, T = 120(1) K, θ_{max} = 66.739°, 17984 total reflections, 4749 with $I_o > 2\sigma(I_o)$, R_{int} = 0.0392, 5431 data, 383 parameters, 0 restraints, GooF = 1.096, R = 0.0380 and wR = 0.1013 [$I_o > 2\sigma(I_o)$], R = 0.0430 and wR = 0.1039 (all reflections), 0.929 <dΔρ < -1.812 eÅ^{-3}.

9. Complex pDIB-9

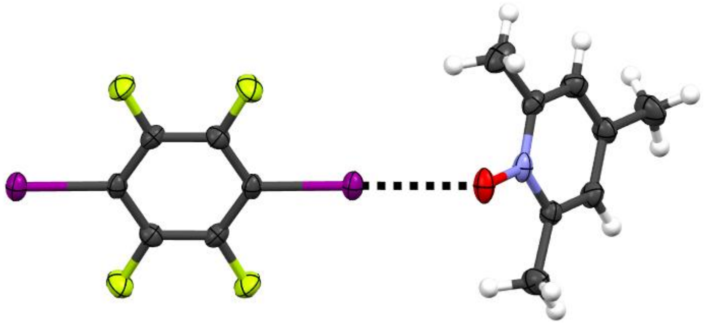

Figure S71. The X-ray crystal structure of **pDIB-9** with the thermal displacement parameter at 50% probability level. The black broken line represents the halogen bond. Colour Key: green = fluorine, purple = iodine, blue = nitrogen, dark grey = carbon, white = hydrogen. **Notes**: The asymmetric unit consists of two 0.5 **pDIB** molecules and one PyNO molecule. For viewing clarity, just the 1:1 donor:acceptor ratio complex is shown.

Crystal data for pDIB-9 (crystallization solvent: $CHCl_3$): CCDC-2337666, $C_{14}H_{11}F_4I_2NO$, M = 539.04 gmol^{-1}, colourless plate, 0.24 × 0.12 × 0.07 mm, monoclinic, space group $P2_1/n$, a = 8.5534(2) Å, b = 16.8429(4) Å, c = 12.1612(3) Å, α = 90°, β = 96.079(2)°, γ = 90°, V = 1742.14(7) Å^3, Z = 4, D_{calc} = 2.055 gcm^{-3}, F(000) = 1008, μ = 3.647 mm^{-1}, T = 120(2) K, θ_{max} = 29.694°, 14055 total reflections, 3795 with $I_o > 2\sigma(I_o)$, R_{int} = 0.0396, 4483 data, 202 parameters, 0 restraints, GooF = 1.030, R = 0.0267 and wR = 0.0507 [$I_o > 2\sigma(I_o)$], R = 0.0350 and wR = 0.0546 (all reflections), 0.646 <dΔρ < -0.444 eÅ^{-3}.

10. Complex pDIB-10

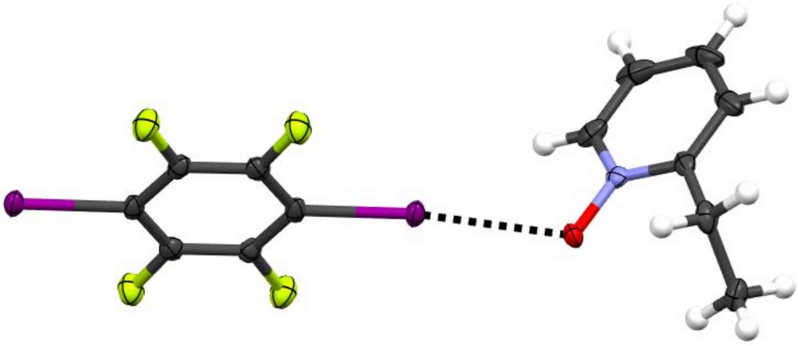

Figure S72. The X-ray crystal structure of **pDIB-10** with the thermal displacement parameter at 50% probability level. The black broken line represents the halogen bond. Colour Key: green = fluorine, purple = iodine, blue = nitrogen, dark grey = carbon, white = hydrogen. **Notes**: The asymmetric unit consists of two 0.5 **pDIB** molecules and one PyNO molecule. For viewing clarity, just the 1:1 donor:acceptor ratio complex is shown.

Crystal data for pDIB-10 (crystallization solvent: $CHCl_3$): CCDC-2337667, $C_{13}H_9F_4I_2NO$, M = 525.01 gmol^{-1}, colourless plate, 0.204 × 0.147 × 0.122 mm, triclinic, space group P-1, a = 7.3464(2) Å, b = 8.8282(3) Å, c = 13.3115(4) Å, α = 98.929(3)°, β = 103.789(3)°, γ = 108.873(3)°, V = 767.60(5)Å^3, Z = 2, D_{calc} = 2.271 gcm^{-3}, F(000) = 488, μ = 4.136 mm^{-1}, T = 173(2) K, θ_{max} = 25.249°, 11150 total reflections, 2544 with $I_o > 2\sigma(I_o)$, R_{int} = 0.0312, 2782 data, 191 parameters, 0 restraints, GooF = 1.061, R = 0.0171 and wR = 0.0355 [$I_o > 2\sigma(I_o)$], R = 0.0199 and wR = 0.0369 (all reflections), 0.344 <dΔρ < -0.392 eÅ^{-3}.

11. Complex pDIB-11

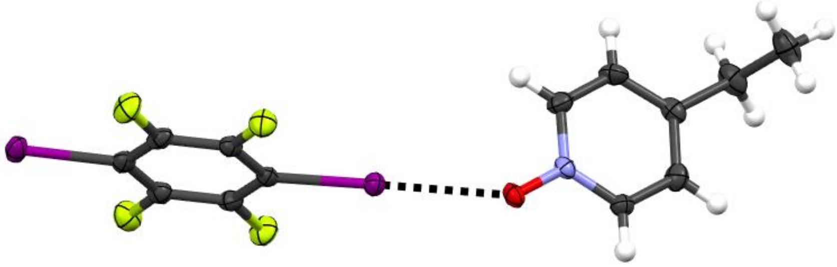

Figure S73. The X-ray crystal structure of **pDIB-11** with the thermal displacement parameter at 50% probability level. The black broken line represents the halogen bond. Colour Key: green = fluorine, purple = iodine, blue = nitrogen, dark grey = carbon, white = hydrogen. **Notes**: The asymmetric unit consists of one **pDIB** molecule and one PyNO molecule.

Crystal data for pDIB-11 (crystallization solvent: $CHCl_3$): CCDC-2337668, $C_{13}H_9F_4I_2NO$, M = 525.01 gmol^{-1}, colourless plate, 0.22 × 0.18 × 0.08 mm, monoclinic, space group $P2_1/c$, a = 11.3309(3) Å, b = 18.9240(5) Å, c = 7.5974(2) Å, α = 90°, β = 98.978(3)°, γ = 90°, V = 1609.12(7) Å^3, Z = 4, D_{calc} = 2.167 gcm^{-3}, F(000) = 976, μ = 3.946 mm^{-1}, T = 173(2) K, θ_{max} = 25.249°, 11093 total reflections, 2560 with $I_o > 2\sigma(I_o)$, R_{int} = 0.0228, 2912 data, 191 parameters, 0 restraints, GooF = 1.059, R = 0.0185 and wR = 0.0393 [$I_o > 2\sigma(I_o)$], R = 0.0225 and wR = 0.0410 (all reflections), 0.739 <dΔρ < -0.674 eÅ^{-3}.

12. Complex pDIB-12

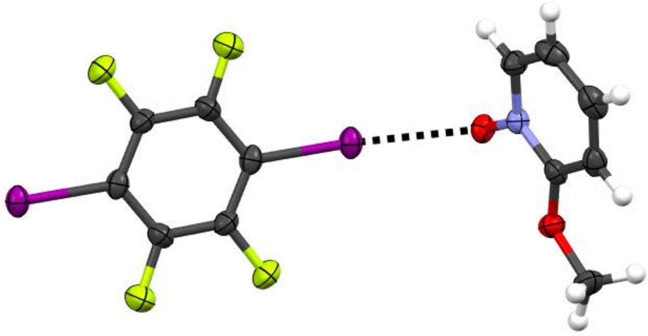

Figure S74. The X-ray crystal structure of **pDIB-12** with the thermal displacement parameter at 50% probability level. The black broken line represents the halogen bond. Colour Key: green = fluorine, purple = iodine, blue = nitrogen, dark grey = carbon, white = hydrogen. **Notes**: The asymmetric unit consists of two 0.5 **pDIB** molecules, one PyNO molecule and one water molecule. For viewing clarity, just the 1:1 donor:acceptor ratio complex is shown.

Crystal data for pDIB-12 (crystallization solvent: $CHCl_3$): CCDC-2337669, $C_{12}H_9F_4I_2NO_3$, M = 545.00 gmol^{-1}, colourless block, 0.11 × 0.10 × 0.09 mm, triclinic, space group $P2_1/c$, a = 7.8612(16) Å, b = 8.9810(18) Å, c = 12.765(3) Å, α = 88.02(3)°, β = 83.31(3)°, γ = 64.73(3)°, V = 809.3(3) Å^3, Z = 2, D_{calc} = 2.236 gcm^{-3}, F(000) = 976, μ = 3.936 mm^{-1}, T = 180 K, θ_{max} = 25.243°, 6321 total reflections, 2459 with $I_o > 2\sigma(I_o)$, R_{int} = 0.0263, 2904 data, 207 parameters, 0 restraints, GooF = 1.084, R = 0.0272 and wR = 0.0572 [$I_o > 2\sigma(I_o)$], R = 0.0353 and wR = 0.0599 (all reflections), 0.432<dΔρ < -0.683 eÅ^{-3}.

13. Complex pDIB-13

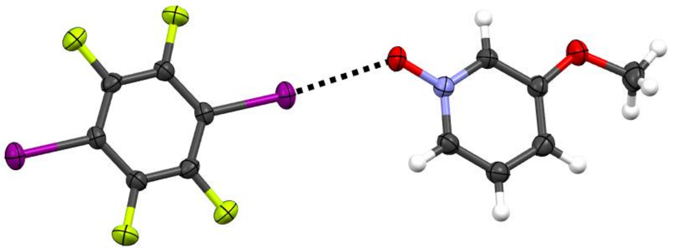

Figure S75. The X-ray crystal structure of **pDIB-13** with the thermal displacement parameter at 50% probability level. The black broken line represents the halogen bond. Colour Key: green = fluorine, purple = iodine, blue = nitrogen, dark grey = carbon, white = hydrogen. **Notes**: The asymmetric unit consists of one 0.5 **pDIB** molecule and one PyNO molecule.

Crystal data for pDIB-13 (crystallization solvent: $CHCl_3$): CCDC-2337670, $C_{18}H_{14}F_4I_2N_2O_4$, M = 652.11 gmol^{-1}, colourless plate, 0.21 × 0.13 × 0.05 mm, triclinic, space group P-1, a = 5.9699(3) Å, b = 9.7020(6) Å, c = 9.8059(6) Å, α = 69.355(3)°, β = 82.818(3)°, γ = 76.785(3)°, V = 516.80(5) Å^3, Z = 4, D_{calc} = 2.095 gcm^{-3}, F(000) = 310, μ = 3.106 mm^{-1}, T = 173(2) K, θ_{max} = 28.837°, 4772 total reflections, 2281 with $I_o > 2\sigma(I_o)$, R_{int} = 0.0272, 2657 data, 136 parameters, 0 restraints, GooF = 1.030, R = 0.0333 and wR = 0.0652 [$I_o > 2\sigma(I_o)$], R = 0.0433 and wR = 0.0685 (all reflections), 0.646 <dΔρ < -0.444 eÅ^{-3}.

14. Complex pDIB-14

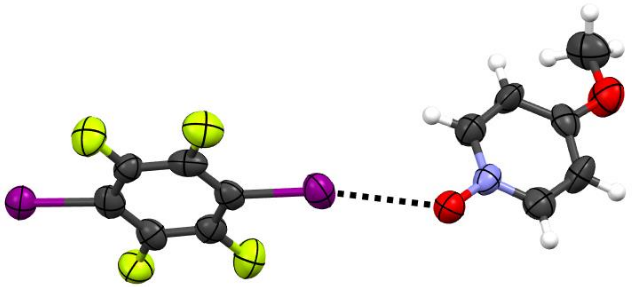

Figure S76. The X-ray crystal structure of **pDIB-14** with the thermal displacement parameter at 50% probability level. The black broken line represents the halogen bond. Colour Key: green = fluorine, purple = iodine, blue = nitrogen, dark grey = carbon, white = hydrogen. **Notes**: The asymmetric unit consists of two **pDIB** molecules and two PyNO molecules. For viewing clarity, just the 1:1 donor:acceptor ratio complex is shown.

Crystal data for pDIB-14 (crystallization solvent: $CHCl_3$): CCDC-2337671, $C_{12}H_7F_4I_2NO_2$, M = 526.99 gmol^{-1}, colourless block, 0.29 × 0.22 × 0.13 mm, triclinic, space group P-1, a = 7.7317(15) Å, b = 8.4003(17) Å, c = 23.736(5) Å, α = 87.71(3)°, β = 89.22(3)°, γ = 86.17(3)°, V = 1536.9(5) Å^3, Z = 4, D_{calc} = 2.278 gcm^{-3}, F(000) = 976, μ = 4.136 mm^{-1}, T = 170 K, θ_{max} = 25.250°, 30816 total reflections, 4193 with $I_o > 2\sigma(I_o)$, R_{int} = 0.0633, 5545 data, 382 parameters, 0 restraints, GooF = 1.039, R = 0.0818 and wR = 0.2250 [$I_o > 2\sigma(I_o)$], R = 0.1048 and wR = 0.2452 (all reflections), 3.525 <dΔρ < -1.986 eÅ^{-3}.

15. Complex pDIB-15

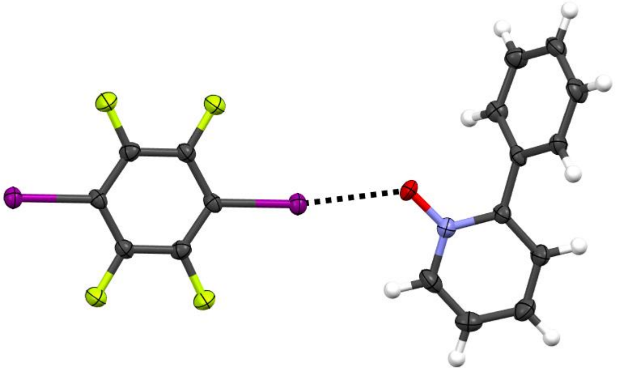

Figure S77. The X-ray crystal structure of **pDIB-15** with the thermal displacement parameter at 50% probability level. The black broken line represents the halogen bond. Colour Key: green = fluorine, purple = iodine, blue = nitrogen, dark grey = carbon, white = hydrogen. **Notes**: The asymmetric unit consists of one 0.5 **pDIB** molecule and one PyNO molecule.

Crystal data for pDIB-15 (crystallization solvent: $CHCl_3$): CCDC-2337672, $C_{14}H_9F_2INO$, M = 372.12 gmol^{-1}, colourless prism, 0.10 × 0.07 × 0.05 mm, monoclinic, space group $P2_1/c$, a = 5.8380(2) Å, b = 13.5701(7) Å, c = 16.4943(6) Å, α = 90°, β = 95.941(2)°, γ = 90°, V = 1299.70(9) Å^3, Z = 4, D_{calc} = 1.902 gcm^{-3}, F(000) = 716, μ = 2.478 mm^{-1}, T = 170 K, θ_{max} = 28.340°, 5866 total reflections, 2281 with $I_o > 2\sigma(I_o)$, R_{int} = 0.0523, 3233 data, 172 parameters, 0 restraints, GooF = 1.078, R = 0.0512 and wR = 0.0844 [$I_o > 2\sigma(I_o)$], R = 0.0839 and wR = 0.0926 (all reflections), 0.757 <dΔρ < -0.759 eÅ^{-3}.

16. Complex pDIB-16

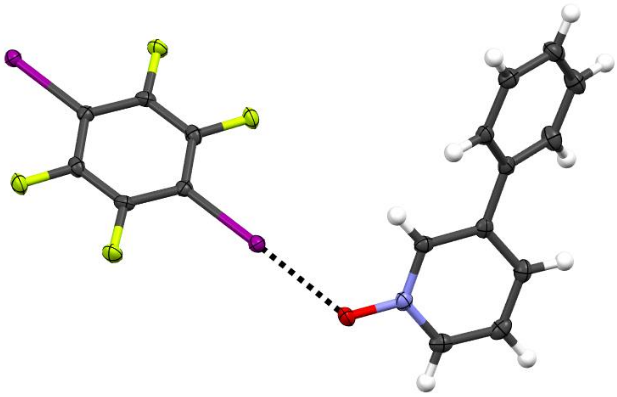

Figure S78. The X-ray crystal structure of **pDIB-16** with the thermal displacement parameter at 50% probability level. The black broken line represents the halogen bond. Colour Key: green = fluorine, purple = iodine, blue = nitrogen, dark grey = carbon, white = hydrogen. **Notes**: The asymmetric unit consists of one 0.5 **pDIB** molecule and one PyNO molecule.

Crystal data for pDIB-16 (crystallization solvent: $CHCl_3$): CCDC-2337673, $C_{14}H_9F_2INO$, M = 372.12 gmol^{-1}, colourless block, 0.3 × 0.2 × 0.11 mm, monoclinic, space group $P2_1/n$, a = 13.5087(3) Å, b = 6.7658(2) Å, c = 13.9627(4) Å, α = 90°, β = 90.086(2)°, γ = 90°, V = 1276.15(6) Å^3, Z = 4, D_{calc} = 1.937 gcm^{-3}, F(000) = 716, μ = 2.524 mm^{-1}, T = 170 K, θ_{max} = 25.247°, 8866 total reflections, 2144 with $I_o > 2\sigma(I_o)$, R_{int} = 0.0282, 2309 data, 172 parameters, 0 restraints, GooF = 1.049, R = 0.0181 and wR = 0.0424 [$I_o > 2\sigma(I_o)$], R = 0.0205 and wR = 0.0436 (all reflections), 0.491 <dΔρ < -0.283 eÅ^{-3}.

17. Complex pDIB-17

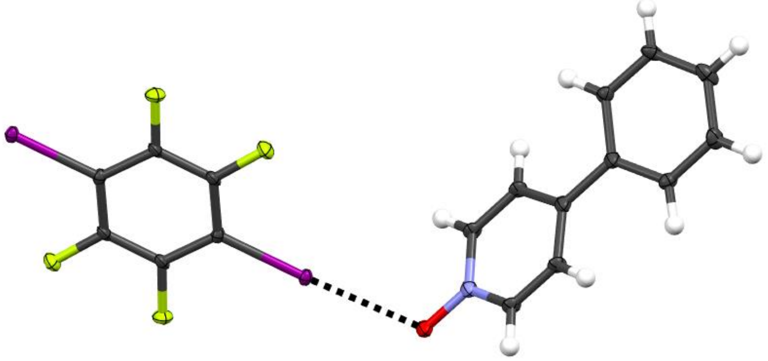

Figure S79. The X-ray crystal structure of **pDIB-17** with the thermal displacement parameter at 50% probability level. The black broken line represents the halogen bond. Colour Key: green = fluorine, purple = iodine, blue = nitrogen, dark grey = carbon, white = hydrogen. **Notes**: The asymmetric unit consists of two 0.5 **pDIB** molecules and one 0.5 PyNO molecule.

Crystal data for pDIB-17 (crystallization solvent: $CHCl_3$): CCDC-2337674, $C_{23}H_9F_8I_4NO$, M = 974.91 gmol^{-1}, colourless block, 0.23 × 0.19 × 0.11 mm, monoclinic, space group C2/c, a = 7.42750(17) Å, b = 28.9561(7) Å, c = 11.9853(3) Å, α = 90°, β = 98.405(2)°, γ = 90°, V = 2550.00(10) Å^3, Z = 4, D_{calc} = 2.539 gcm^{-3}, F(000) = 1784, μ = 39.111 mm^{-1}, T = 123 K, θ_{max} = 66.624°, 7113 total reflections, 2063 with $I_o > 2\sigma(I_o)$, R_{int} = 0.0583, 2258 data, 142 parameters, 0 restraints, GooF = 1.054, R = 0.0442 and wR = 0.1162 [$I_o > 2\sigma(I_o)$], R = 0.0473 and wR = 0.1215 (all reflections), 1.480 <dΔρ < -2.351 eÅ^{-3}.

18. Complex pDIB-18

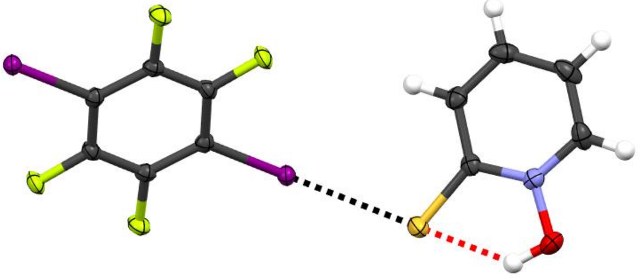

Figure S80. The X-ray crystal structure of **pDIB-18** with the thermal displacement parameter at 50% probability level. The black broken line represents the halogen bond. Colour Key: green = fluorine, purple = iodine, blue = nitrogen, dark grey = carbon, white = hydrogen. **Notes**: The asymmetric unit consists of one 0.5 **pDIB** molecules and one PyNO molecule.

Crystal data for pDIB-18 (crystallization solvent: $CHCl_3$): CCDC-2337675, $C_{16}H_{10}F_4I_2N_2O_2S_2$, M = 656.18 gmol^{-1}, colourless block, 0.01 × 0.06 × 0.034 mm, triclinic, space group P-1, a = 6.8809(3) Å, b = 7.1411(3) Å, c = 11.4662(6) Å, α = 75.827(4)°, β = 83.728(4)°, γ = 61.884(4)°, V = 481.79(4) Å^3, Z = 1, D_{calc} = 2.262 gcm^{-3}, F(000) = 310, μ = 28.172 mm^{-1}, T = 120 K, θ_{max} = 66.616°, 5758 total reflections, 1686 with $I_o > 2\sigma(I_o)$, R_{int} = 0.0278, 1710 data, 131 parameters, 0 restraints, GooF = 1.099, R = 0.0195 and wR = 0.0493 [$I_o > 2\sigma(I_o)$], R = 0.0197 and wR = 0.0495 (all reflections), 0.572 <dΔρ < -0.967 eÅ^{-3}.

19. Complex pDIB-18a

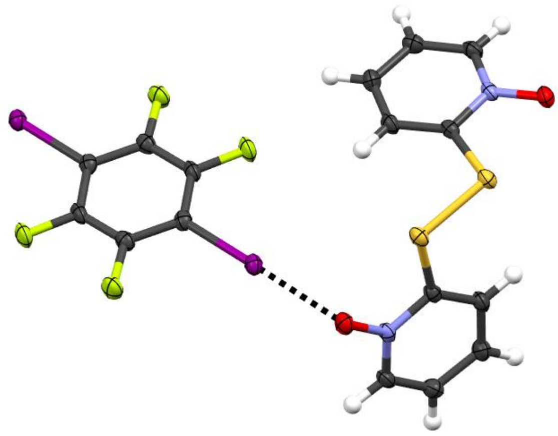

Figure S81. The X-ray crystal structure of **pDIB-18a** with the thermal displacement parameter at 50% probability level. The black broken line represents the halogen bond. Colour Key: green = fluorine, purple = iodine, blue = nitrogen, dark grey = carbon, white = hydrogen. **Notes**: The asymmetric unit consists of one 0.5 **pDIB** molecules and one 0.5 PyNO molecule.

Crystal data for pDIB-18a (crystallization solvent: $CHCl_3$): CCDC-2337676, $C_{16}H_8F_4I_2N_2O_2S_2$, M = 654.16 gmol^{-1}, colourless block, 0.27 × 0.08 × 0.05 mm, monoclinic, space group C2/c, a = 20.0308(4) Å, b = 4.55960(10) Å, c = 22.5770(4) Å, α = 90°, β = 107.353(2)°, γ = 90°, V = 1968.16(7) Å^3, Z = 4, D_{calc} = 2.208 gcm^{-3}, F(000) = 1232, μ = 3.460 mm^{-1}, T = 120 K, θ_{max} = 25.242°, 15343 total reflections, 2488 with $I_o > 2\sigma(I_o)$, R_{int} = 0.0230, 2663 data, 127 parameters, 0 restraints, GooF = 1.052, R = 0.0180 and wR = 0.0398 [$I_o > 2\sigma(I_o)$], R = 0.0203 and wR = 0.0410 (all reflections), 0.682 <dΔρ <-0.531 eÅ^{-3}.

20. Complex pDIB-19

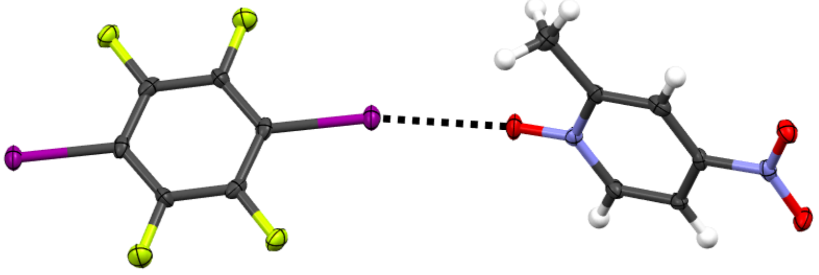

Figure S82. The X-ray crystal structure of **pDIB-19** with the thermal displacement parameter at 50% probability level. The black broken line represents the halogen bond. Colour Key: green = fluorine, purple = iodine, blue = nitrogen, dark grey = carbon, white = hydrogen. **Notes**: The asymmetric unit consists of two 0.5 **pDIB** molecules and one 0.5 PyNO molecule.

Crystal data for pDIB-19 (crystallization solvent: $CHCl_3$): CCDC-2337677, $C_{12}H_6F_4I_2N_2O_3$, M = 555.99 gmol^{-1}, yellow plate, 0.15 × 0.13 × 0.10 mm, monoclinic, space group $P2_1/c$, a = 22.3283(5) Å, b = 6.31653(14) Å, c = 10.8482(2) Å, α = 90°, β = 97.924(2)°, γ = 90°, V = 1515.39(6) Å^3, Z = 1, D_{calc} = 2.437 gcm^{-3}, F(000) = 1032, μ = 4.209 mm^{-1}, T = 123 K, θ_{max} = 25.249°, 4812 total reflections, 2300 with $I_o > 2\sigma(I_o)$, R_{int} = 0.0284, 2714 data, 209 parameters, 0 restraints, GooF = 1.000, R = 0.0264 and wR = 0.0465 [$I_o > 2\sigma(I_o)$], R = 0.0341 and wR = 0.0508 (all reflections), 0.464 <dΔρ < -0.564 eÅ^{-3}.

21. Complex pDIB-21

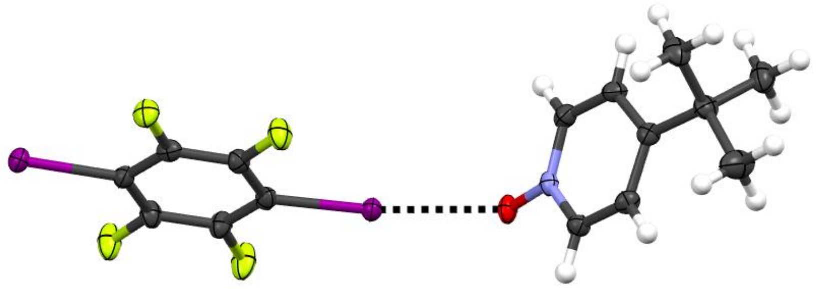

Figure S83. The X-ray crystal structure of **pDIB-21** with the thermal displacement parameter at 50% probability level. The black broken line represents the halogen bond. Colour Key: green = fluorine, purple = iodine, blue = nitrogen, dark grey = carbon, white = hydrogen. **Notes**: The asymmetric unit consists of two **pDIB** molecules and two PyNO molecules. For viewing clarity, just the 1:1 donor:acceptor ratio complex is shown.

Crystal data for pDIB-21 (crystallization solvent: $CHCl_3$): CCDC-2337678, $C_{15}H_{13}F_4I_2NO$, M = 553.06 gmol^{-1}, colourless prism, 0.20 × 0.15 × 0.14 mm, triclinic, space group P-1, a = 10.1852(3) Å, b = 13.1769(4) Å, c = 15.1672(4) Å, α = 111.830(3)°, β = 106.583(2)°, γ = 93.933(3)°, V = 1775.75(10) Å^3, Z = 4, D_{calc} = 2.069 gcm^{-3}, F(000) = 1040, μ = 3.581 mm^{-1}, T = 150(2) K, θ_{max} = 30.472°, 30565 total reflections, 8153 with $I_o > 2\sigma(I_o)$, R_{int} = 0.0276, 9754 data, 415 parameters, 0 restraints, GooF = 1.030, R = 0.0245 and wR = 0.0463 [$I_o > 2\sigma(I_o)$], R = 0.0334 and wR = 0.0498 (all reflections), 0.460<dΔρ < -0.571 eÅ^{-3}.

22. Complex pDIB-23

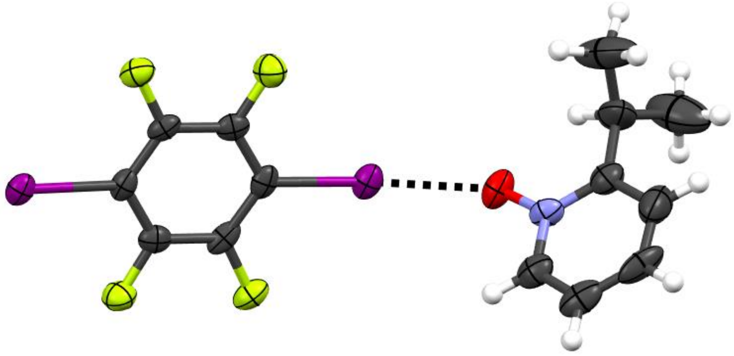

Figure S84. The X-ray crystal structure of **pDIB-23** with the thermal displacement parameter at 50% probability level. The black broken line represents the halogen bond. Colour Key: green = fluorine, purple = iodine, blue = nitrogen, dark grey = carbon, white = hydrogen. **Notes**: The asymmetric unit consists of one **pDIB** molecule and one PyNO molecule.

Crystal data for pDIB-23 (crystallization solvent: $CHCl_3$): CCDC-2337679, $C_{14}H_{11}F_4I_2NO$, M = 539.04 gmol^{-1}, colourless block, 0.15 × 0.13 × 0.06 mm, orthorhombic, space group Pbca, a = 7.4662(4) Å, b = 20.9801(14) Å, c = 21.6655(14) Å, α = 90°, β = 90°, γ = 90°, V = 3393.7(4) Å^3, Z = 8, D_{calc} = 2.110 gcm^{-3}, F(000) = 2016, μ = 3.745 mm^{-1}, T = 180 K, θ_{max} = 25.248°, 6954 total reflections, 2108 with $I_o > 2\sigma(I_o)$, R_{int} = 0.0468, 3048 data, 201 parameters, 0 restraints, GooF = 1.060, R = 0.0435 and wR = 0.0806 [$I_o > 2\sigma(I_o)$], R = 0.0755 and wR = 0.0979 (all reflections), 1.167<dΔρ < -0.631 eÅ^{-3}.

23. Complex pDIB-24

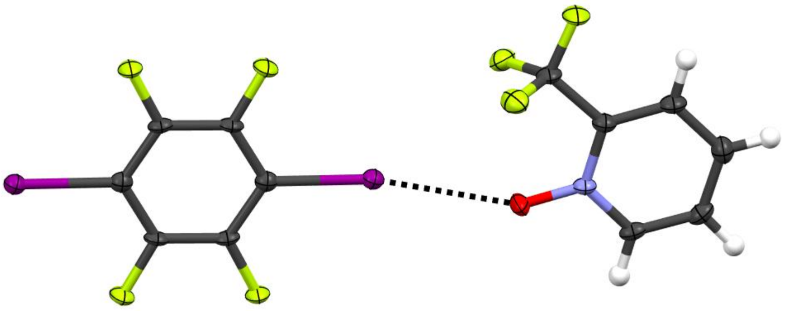

Figure S85. The X-ray crystal structure of **pDIB-24** with the thermal displacement parameter at 50% probability level. The black broken line represents the halogen bond. Colour Key: green = fluorine, purple = iodine, blue = nitrogen, dark grey = carbon, white = hydrogen. **Notes**: The asymmetric unit consists of one 0.5 **pDIB** molecule and one 0.5 PyNO molecule.

Crystal data for pDIB-24 (crystallization solvent: $CHCl_3$): CCDC-2337680, $C_{18}H_8F_{10}I_2N_2O_2$, M = 728.06 gmol^{-1}, colourless block, 0.26 × 0.07 × 0.06 mm, monoclinic, space group C2/m, a = 14.9249(10) Å, b = 6.5724(2) Å, c = 13.2819(9) Å, α = 90°, β = 127.446(10)°, γ = 90°, V = 1034.38(15) Å^3, Z = 2, D_{calc} = 2.338 gcm^{-3}, F(000) = 684, μ = 3.150 mm^{-1}, T = 123 K, θ_{max} = 25.247°, 1789 total reflections, 948 with $I_o > 2\sigma(I_o)$, R_{int} = 0.0215, 1006 data, 100 parameters, 0 restraints, GooF = 1.051, R = 0.0226 and wR = 0.0523 [$I_o > 2\sigma(I_o)$], R = 0.0244 and wR = 0.0538 (all reflections), 0.626<dΔρ < -0.570 eÅ^{-3}.

24. Complex pDIB-26

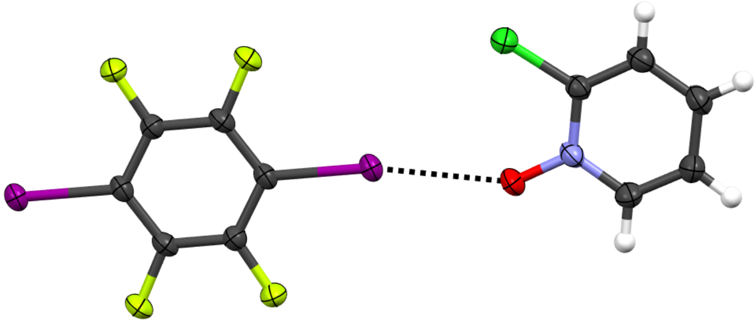

Figure S86. The X-ray crystal structure of **pDIB-26** with the thermal displacement parameter at 50% probability level. The black broken line represents the halogen bond. Colour Key: green = fluorine, purple = iodine, blue = nitrogen, dark grey = carbon, white = hydrogen. **Notes**: The asymmetric unit consists of one 0.5 **pDIB** molecule and one PyNO molecule.

Crystal data for pDIB-26 (crystallization solvent: acetone): CCDC-2337681, $C_{16}H_8Cl_2F_4I_2N_2O_2$, M = 660.94 gmol^{-1}, colourless block, 0.075 × 0.053 × 0.026 mm, monoclinic, space group $P2_1/c$, a = 5.10190(10) Å, b = 10.6241(2) Å, c = 18.0664(4) Å, α = 90°, β = 92.408(2)°, γ = 90°, V = 978.39(3) Å^3, Z = 2, D_{calc} = 2.244 gcm^{-3}, F(000) = 620, μ = 28.265 mm^{-1}, T = 120 K, θ_{max} = 66.732°, 9318 total reflections, 1611 with $I_o > 2\sigma(I_o)$, R_{int} = 0.0405, 1724 data, 127 parameters, 0 restraints, GooF = 1.082, R = 0.0237 and wR = 0.0628 [$I_o > 2\sigma(I_o)$], R = 0.0254 and wR = 0.0638 (all reflections), 0.698<dΔρ < -0.846 eÅ^{-3}.

25. Complex pDIB-27

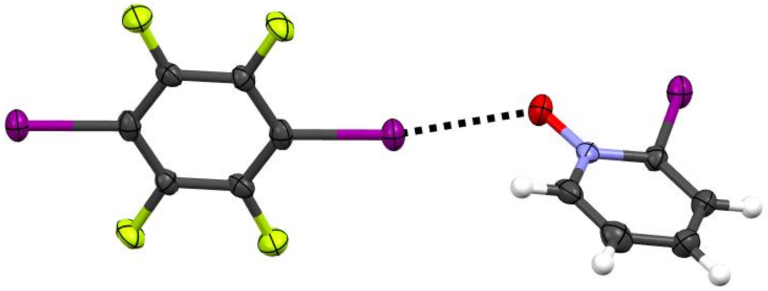

Figure S87. The X-ray crystal structure of **pDIB-27** with the thermal displacement parameter at 50% probability level. The black broken line represents the halogen bond. Colour Key: green = fluorine, purple = iodine, blue = nitrogen, dark grey = carbon, white = hydrogen. **Notes**: The asymmetric unit consists of two 0.5 **pDIB** molecules, one **pDIB** molecule and two PyNO molecules. For viewing clarity, just the 1:1 donor:acceptor ratio complex is shown.

Crystal data for pDIB-27 (crystallization solvent: $CHCl_3$): CCDC-2337682, $C_{11}H_4F_4I_3NO$, M = 622.85 gmol^{-1}, colourless block, 0.28 × 0.02 × 0.02 mm, triclinic, space group P-1, a = 7.9737(16)Å, b = 13.645(3) Å, c = 13.974(3) Å, α = 87.51(3)°, β = 82.92(3)°, γ = 89.69(3)°, V = 1507.3(5)Å^3, Z = 4, D_{calc} = 2.745 gcm^{-3}, F(000) = 1120, μ = 6.257 mm^{-1}, T = 180 K, θ_{max} = 25.245°, 11629 total reflections, 4538 with $I_o > 2\sigma(I_o)$, R_{int} = 0.0307, 5433 data, 361 parameters, 0 restraints, GooF = 1.067, R = 0.0337 and wR = 0.0695 [$I_o > 2\sigma(I_o)$], R = 0.0448 and wR = 0.0741 (all reflections), 0.768<dΔρ < -1.123 eÅ^{-3}.

26. Complex pDIB-28

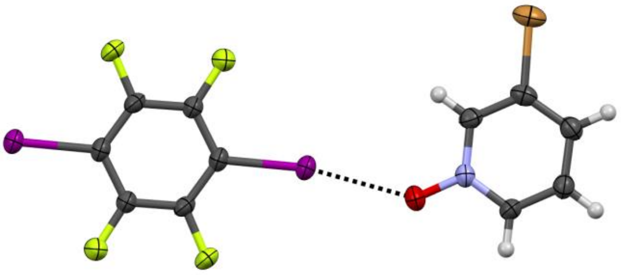

Figure S88. The X-ray crystal structure of **pDIB-28** with the thermal displacement parameter at 50% probability level. The black broken line represents the halogen bond. Colour Key: green = fluorine, purple = iodine, blue = nitrogen, dark grey = carbon, white = hydrogen. **Notes**: The asymmetric unit consists of one 0.5 **pDIB** molecules, one PyNO molecule and one 0.25 chloroform. For viewing clarity, just the 1:1 donor:acceptor ratio complex is shown.

Crystal data for pDIB-28 (crystallization solvent: $CHCl_3$): CCDC-2337683, $C_{33}H_{17}Br_4Cl_3F_8I_4N_4O_4$, M = 1619.09 gmol^{-1}, colourless block, 0.11 × 0.08 × 0.04 mm, monoclinic, space group I2/a, a = 7.19310(10)Å, b = 12.65200(10) Å, c = 24.7981(3) Å, α = 90°, β = 93.7520(10)°, γ = 90°, V = 2251.97(5)Å^3, Z = 2, D_{calc} = 2.388 gcm^{-3}, F(000) = 1500, μ = 28.206 mm^{-1}, T = 180 K, θ_{max} = 79.375°, 13099 total reflections, 2332 with $I_o > 2\sigma(I_o)$, R_{int} = 0.0460, 2440 data, 163 parameters, 9 restraints, GooF = 1.062, R = 0.0388 and wR = 0.1113 [$I_o > 2\sigma(I_o)$], R = 0.0398 and wR = 0.1124 (all reflections), 0.958<dΔρ < -1.058 eÅ^{-3}.

27. Complex pDIB-29

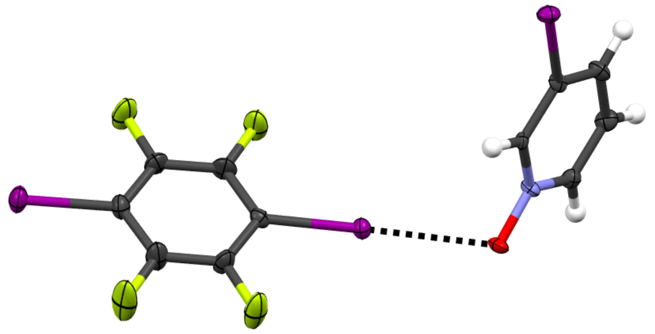

Figure S89. The X-ray crystal structure of **pDIB-29** with the thermal displacement parameter at 50% probability level. The black broken line represents the halogen bond. Colour Key: green = fluorine, purple = iodine, blue = nitrogen, dark grey = carbon, white = hydrogen. **Notes**: The asymmetric unit consists of one **pDIB** molecule and one PyNO molecule.

Crystal data for pDIB-29 (crystallization solvent: $CHCl_3$): CCDC-2337684, $C_{11}H_4F_4I_3NO$, M = 622.85 $gmol^{-1}$, colourless block, 0.183 × 0.164 × 0.044 mm, triclinic, space group P-1, a = 8.0697(6)Å, b = 8.2165(6) Å, c = 12.7569(9) Å, α = 82.494(6)°, β = 88.506(6)°, γ = 65.219(7)°, V = 760.99(10) $Å^3$, Z = 2, D_{calc} = 2.718 gcm^{-3}, F(000) = 560, μ = 6.196 mm^{-1}, T = 123 K, $θ_{max}$ = 25.242°, 4148 total reflections, 2435 with $I_o > 2σ(I_o)$, R_{int} = 0.0268, 2737 data, 181 parameters, 0 restraints, GooF = 1.011, R = 0.0298 and wR = 0.0552 [$I_o > 2σ(I_o)$], R = 0.0346 and wR = 0.0586 (all reflections), 0.800<dΔρ < -0.819 $eÅ^{-3}$.

28. Complex pDIB-31

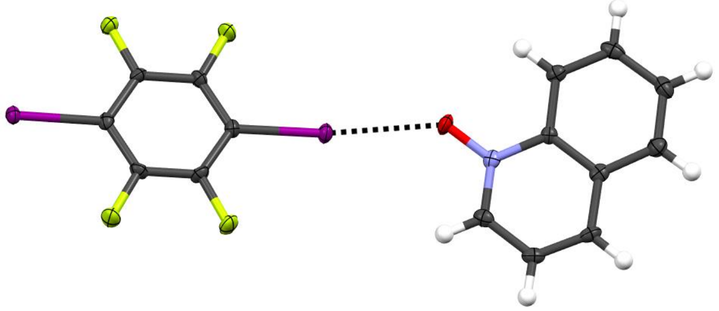

Figure S90. The X-ray crystal structure of **pDIB-31** with the thermal displacement parameter at 50% probability level. The black broken line represents the halogen bond. Colour Key: green = fluorine, purple = iodine, blue = nitrogen, dark grey = carbon, white = hydrogen. **Notes**: The asymmetric unit consists of one **pDIB** molecule and two PyNO molecules. For viewing clarity, just the 1:1 donor:acceptor ratio complex is shown.

Crystal data for pDIB-31 (crystallization solvent: $CHCl_3$): CCDC-2337685, $C_{24}H_{14}F_4I_2N_2O_2$, M = 692.17 $gmol^{-1}$, colourless block, 0.152 × 0.101 × 0.061 mm, triclinic, space group P-1, a = 6.9539(2) Å, b = 12.2400(4) Å, c = 14.6681(6) Å, α = 66.390(4)°, β = 86.017(3)°, γ = 78.132(3)°, V = 1119.41(8) $Å^3$, Z = 2, D_{calc} = 2.054 gcm^{-3}, F(000) = 660, μ = 2.868 mm^{-1}, T = 120 K, $θ_{max}$ = 25.247°, 6196 total reflections, 3579 with $I_o > 2σ(I_o)$, R_{int} = 0.0228, 4030 data, 307 parameters, 0 restraints, Goof = 1.028, R = 0.0247 and wR = 0.0473 [$I_o > 2σ(I_o)$], R = 0.0294 and wR = 0.0508 (all reflections), 0.588<dΔρ < -0.600 $eÅ^{-3}$.

2.7. trIB-PyNO halogen-bonded complexes

1. Complex trIB-1

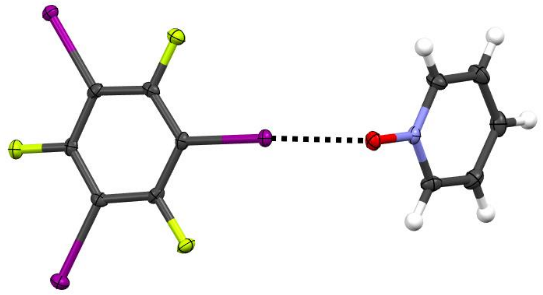

Figure S91. The X-ray crystal structure of **trIB-1** with the thermal displacement parameter at 50% probability level. The black broken line represents the halogen bond. Colour Key: green = fluorine, purple = iodine, blue = nitrogen, dark grey = carbon, white = hydrogen. **Notes**: The asymmetric unit consists of one **trIB** molecule and one PyNO molecule.

Crystal data for trIB-1 (crystallization solvent: $CHCl_3$): CCDC-2337690, $C_{10}H_{10}N_1I_1F_3$, M = 604.86 $gmol^{-1}$, colourless plate, 0.21 × 0.14 × 0.12 mm, monoclinic, space group *P2₁/n*, a = 12.5240(3) Å, b = 7.3246(2) Å, c = 15.5270(3) Å, α = 90°, β = 97.627(2)°, γ = 90°, V = 1411.74(6) $Å^3$, Z = 4, D_{calc} = 2.846 gcm^{-3}, F(000) = 1088, μ = 6.666 mm^{-1}, T = 120(1) K, θ_{max} = 25.248°, 11178 total reflections, 2455 with $I_o > 2\sigma(I_o)$, R_{int} = 0.0265, 2557 data, 172 parameters, 0 restraints, GooF = 1.132, R = 0.0168 and wR = 0.0390 $[I_o > 2\sigma(I_o)]$, R = 0.0180 and wR = 0.0395 (all reflections), 0.536<dΔρ < -0.547 $eÅ^{-3}$.

2. Complex trIB-2

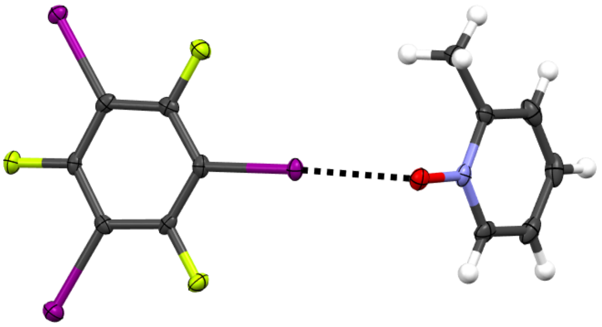

Figure S92. The X-ray crystal structure of **trIB-2** with the thermal displacement parameter at 50% probability level. The black broken line represents the halogen bond. Colour Key: green = fluorine, purple = iodine, blue = nitrogen, dark grey = carbon, white = hydrogen. **Notes**: The asymmetric unit consists of one **trIB** molecule and one PyNO molecule.

Crystal data for trIB-2 (crystallization solvent: CH_2Cl_2): CCDC- 2337691, $C_{12}H_7F_3I_3NO$, M = 618.89 $gmol^{-1}$, colourless block, 0.13 × 0.1 × 0.08 mm, monoclinic, space group *P2₁/c*, a = 9.7641(2) Å, b = 7.4105(2) Å, c = 21.1679(5) Å, α = 90°, β = 98.033(2)°, γ = 90°, V = 1516.61(6) $Å^3$, Z = 4, D_{calc} = 2.710 gcm^{-3}, F(000) = 1120, μ = 6.208 mm^{-1}, T = 120(1) K, θ_{max} = 25.250°, 10601 total reflections, 2592 with $I_o > 2\sigma(I_o)$, R_{int} = 0.0233, 2746 data, 182 parameters, 0 restraints, GooF = 1.055, R = 0.0151 and wR = 0.0348 $[I_o > 2\sigma(I_o)]$, R = 0.0166 and wR = 0.0353 (all reflections), 0.395<dΔρ < -0.414 $eÅ^{-3}$.

3. Complex trIB-3

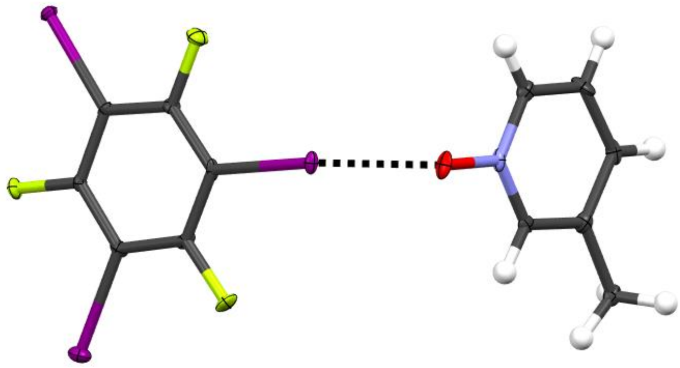

Figure S93. The X-ray crystal structure of **trIB-3** with the thermal displacement parameter at 50% probability level. The black broken line represents the halogen bond. Colour Key: green = fluorine, purple = iodine, blue = nitrogen, dark grey = carbon, white = hydrogen. **Notes**: The asymmetric unit consists of one **trIB** molecule and one PyNO molecule.

Crystal data for trIB-3 (crystallization solvent: $CHCl_3$): CCDC- 2337692, $C_{12}H_7F_3I_3NO$, M = 618.89 gmol^{-1}, colourless block, 0.12 × 0.09 × 0.08 mm, monoclinic, space group *P2₁/n*, a = 7.40100(10) Å, b = 9.51330(10) Å, c = 21.7904(2) Å, α = 90°, β = 99.3490(10)°, γ = 90°, V = 1513.84(3) Å^3, Z = 4, D_{calc} = 2.715 gcm^{-3}, F(000) = 1120, μ = 48.923 mm^{-1}, T = 120(1) K, θ_{max} = 66.744°, 18882 total reflections, 2675 with $I_o > 2\sigma(I_o)$, R_{int} = 0.0292, 2677 data, 170 parameters, 6 restraints, GooF = 1.203, R = 0.0224 and wR = 0.0581 [$I_o > 2\sigma(I_o)$], R = 0.0224 and wR = 0.0581 (all reflections), 0.638<dΔρ <-1.248 eÅ^{-3}.

4. Complex trIB-4

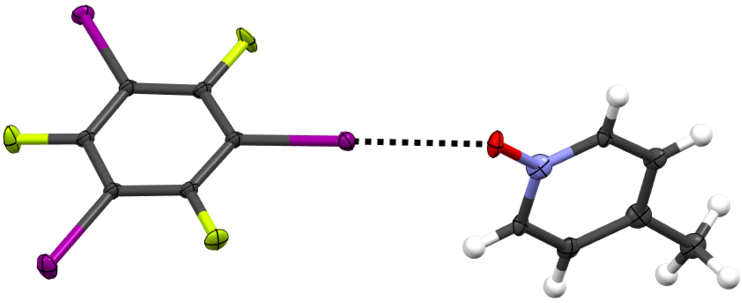

Figure S94. The X-ray crystal structure of **trIB-4** with the thermal displacement parameter at 50% probability level. The black broken line represents the halogen bond. Colour Key: green = fluorine, purple = iodine, blue = nitrogen, dark grey = carbon, white = hydrogen. **Notes**: The asymmetric unit consists of one **trIB** molecule and one PyNO molecule.

Crystal data for trIB-4 (crystallization solvent: $CHCl_3$): CCDC-2337693, $C_{12}H_7F_3I_3NO$, M = 618.89 gmol^{-1}, colourless block, 0.15 × 0.07 × 0.05 mm, triclinic, space group *P-1*, a = 7.5434(2) Å, b = 9.6134(4) Å, c = 10.89290(10) Å, α = 97.039(2)°, β = 94.3040(10)°, γ = 95.262(3)°, V = 777.69(4) Å^3, Z = 2, D_{calc} = 2.643 gcm^{-3}, F(000) = 560, μ = 47.617 mm^{-1}, T = 120(1) K, θ_{max} = 66.659°, 10713 total reflections, 2648 with $I_o > 2\sigma(I_o)$, R_{int} = 0.0451, 2747 data, 134 parameters, 6 restraints, GooF = 1.211, R = 0.0600 and wR = 0.1812 [$I_o > 2\sigma(I_o)$], R = 0.0617 and wR = 0.1817 (all reflections), 3.239<dΔρ < -2.230 eÅ^{-3}.

5. Complex trIB-5

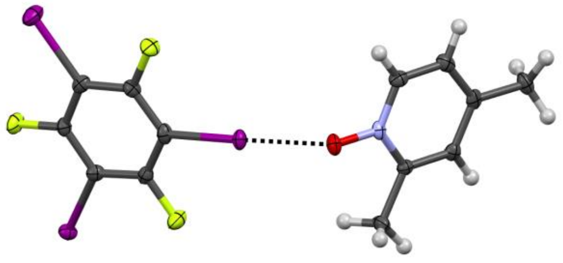

Figure S95. The X-ray crystal structure of **trIB-5** with the thermal displacement parameter at 50% probability level. The black broken line represents the halogen bond. Colour Key: green = fluorine, purple = iodine, blue = nitrogen, dark grey = carbon, white = hydrogen. **Notes**: The asymmetric unit consists of one **trIB** molecule and one PyNO molecule.

Crystal data for trIB-5 (crystallization solvent: $CHCl_3$): CCDC-2337694, $C_{13}H_9F_3I_3NO$, M = 632.91 $gmol^{-1}$, colourless block, 0.1 × 0.06 × 0.04 mm, monoclinic, space group *P2₁/c*, a = 9.81280(10) Å, b = 8.27820(10) Å, c = 20.6163(3) Å, α = 90°, β = 99.1620(10)°, γ = 90°, V = 1653.34(4) $Å^3$, Z = 4, D_{calc} = 2.543 gcm^{-3}, F(000) = 1152, μ = 44.817 mm^{-1}, T = 120(1) K, $θ_{max}$ = 66.747°, 17159 total reflections, 2840 with $I_o > 2σ(I_o)$, R_{int} = 0.0371, 2929 data, 192 parameters, 0 restraints, GooF = 1.114, R = 0.0232 and wR = 0.0568 [$I_o > 2σ(I_o)$], R = 0.0241 and wR = 0.0574 (all reflections), 0.789<dΔρ < -0.726 $eÅ^{-3}$.

6. Complex trIB-6

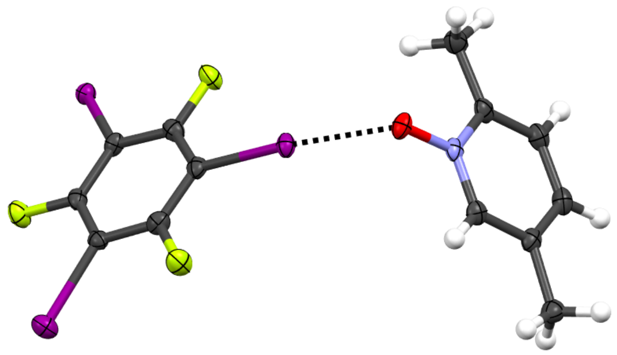

Figure S96. The X-ray crystal structure of **trIB-6** with the thermal displacement parameter at 50% probability level. The black broken line represents the halogen bond. Colour Key: green = fluorine, purple = iodine, blue = nitrogen, dark grey = carbon, white = hydrogen. **Notes**: The asymmetric unit consists of one **trIB** molecule and two PyNO molecules.

Crystal data for trIB-6 (crystallization solvent: $CHCl_3$): CCDC-2337695, $C_{13}H_9F_3I_3NO$, M = 632.91$gmol^{-1}$, colourless block, 0.18 × 0.13 × 0.08 mm, monoclinic, space group *P2₁/n*, a = 12.81090(10) Å, b = 8.20280(10) Å, c = 15.4866(2) Å, α = 90°, β = 96.7230(10)°, γ = 90°, V = 1616.22(3) $Å^3$, Z = 4, D_{calc} = 2.601 gcm^{-3}, F(000) = 1152, μ = 45.847 mm^{-1}, T = 120(1) K, $θ_{max}$ = 66.737°, 17852 total reflections, 2823 with $I_o > 2σ(I_o)$, R_{int} = 0.0424, 2860 data, 192 parameters, 0 restraints, GooF = 1.123, R = 0.0238 and wR = 0.0623 [$I_o > 2σ(I_o)$], R = 0.0242 and wR = 0.0626 (all reflections), 0.836<dΔρ < -0.862 $eÅ^{-3}$.

7. Complex trIB-7

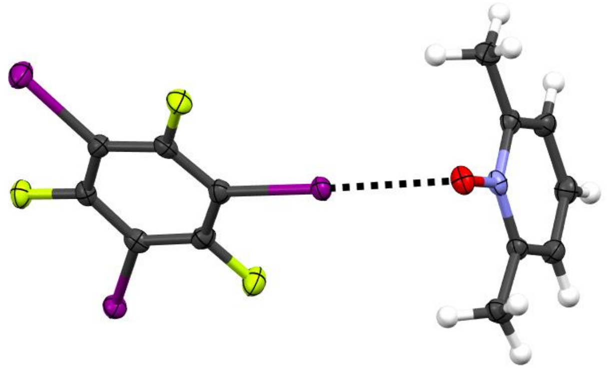

Figure S97. The X-ray crystal structure of **trIB-7** with the thermal displacement parameter at 50% probability level. The black broken line represents the halogen bond. Colour Key: green = fluorine, purple = iodine, blue = nitrogen, dark grey = carbon, white = hydrogen. **Notes**: The asymmetric unit consists of one **trIB** molecule and one PyNO molecule.

Crystal data for trIB-7 (crystallization solvent: $CHCl_3$): CCDC 2337696, $C_{13}H_9F_3I_3NO$, M = 632.91 gmol^{-1}, colourless block, 0.07 × 0.06 × 0.03 mm, triclinic, space group *P-1*, a = 7.2824(2) Å, b = 10.4915(3) Å, c = 11.1166(3) Å, α = 95.639(2)°, β = 97.777(2)°, γ = 98.144(2)°, V = 827.05(4) Å^3, Z = 2, D_{calc} = 2.541 gcm^{-3}, F(000) = 576, μ = 44.797 mm^{-1}, T = 120(1) K, θ_{max} = 66.746°, 12016 total reflections, 2697 with $I_o > 2\sigma(I_o)$, R_{int} = 0.0374, 2923 data, 192 parameters, 6 restraints, GooF = 1.013, R = 0.0239 and wR = 0.0562 [$I_o > 2\sigma(I_o)$], R = 0.0268 and wR = 0.0573 (all reflections), 1.216<dΔρ < -0.670 eÅ^{-3}.

8. Complex trIB-8

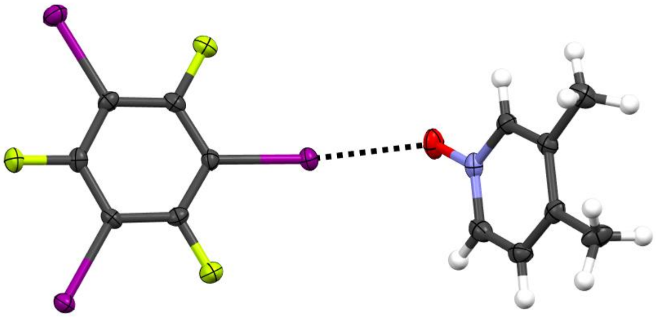

Figure S98. The X-ray crystal structure of **trIB-8** with the thermal displacement parameter at 50% probability level. The black broken line represents the halogen bond. Colour Key: green = fluorine, purple = iodine, blue = nitrogen, dark grey = carbon, white = hydrogen. **Notes**: The asymmetric unit consists of one **trIB** molecule and one PyNO molecule.

Crystal data for trIB-8 (crystallization solvent: $CHCl_3$): CCDC- 2337697, $C_{13}H_9F_3I_3NO$, M = 632.91 gmol^{-1}, colourless block, 0.08 × 0.04 × 0.03 mm, triclinic, space group *P-1*, a = 8.0471(3) Å, b = 9.4347(2) Å, c = 11.2492(3) Å, α = 82.531(2)°, β = 78.532(3)°, γ = 76.864(3)°, V = 811.89(4) Å^3, Z = 2, D_{calc} = 2.589 gcm^{-3}, F(000) = 576, μ = 45.633 mm^{-1}, T = 120(1) K, θ_{max} = 66.748°, 11342 total reflections, 2715 with $I_o > 2\sigma(I_o)$, R_{int} = 0.0344, 2865 data, 192 parameters, 0 restraints, GooF = 1.043, R = 0.0214 and wR = 0.0511 [$I_o > 2\sigma(I_o)$], R = 0.0227 and wR = 0.0517 (all reflections), 0.719<dΔρ < -0.763 eÅ^{-3}.

9. Complex trIB-9

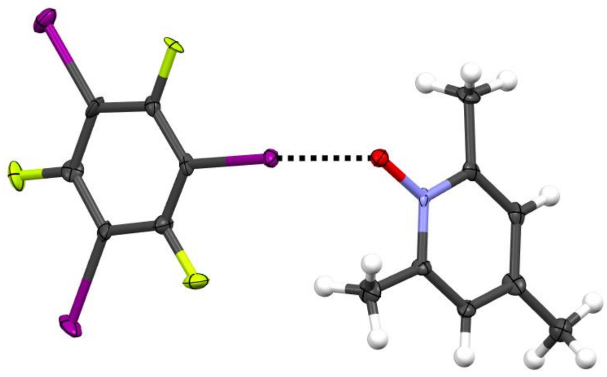

Figure S99. The X-ray crystal structure of **trIB-9** with the thermal displacement parameter at 50% probability level. The black broken line represents the halogen bond. Colour Key: green = fluorine, purple = iodine, blue = nitrogen, dark grey = carbon, white = hydrogen. **Notes**: The asymmetric unit consists of two **trIB** molecules and one PyNO molecule.

Crystal data for trIB-9 (crystallization solvent: $CHCl_3$): CCDC- 2337698, $C_{20}H_{11}F_6I_6NO$, M = 1156.70 $gmol^{-1}$, colourless block, 0.17 × 0.16 × 0.12 mm, monoclinic, space group *Ia*, a = 8.14230(10) Å, b = 40.9830(4) Å, c = 8.36870(10) Å, α = 90°, β = 90.8170(10)°, γ = 90°, V = 2792.32(6) Å^3, Z = 4, D_{calc} = 2.751 gcm^{-3}, F(000) = 2072, μ = 52.927 mm^{-1}, T = 120(1) K, θ_{max} = 65.247°, 20865 total reflections, 4724 with $I_o > 2\sigma(I_o)$, R_{int} = 0.0531, 4743 data, 310 parameters, 62 restraints, GooF = 1.032, R = 0.0416 and wR = 0.1117 [$I_o > 2\sigma(I_o)$], R = 0.0417 and wR = 0.1118 (all reflections), 1.535<dΔρ < -1.982 eÅ^{-3}.

10. Complex trIB-11

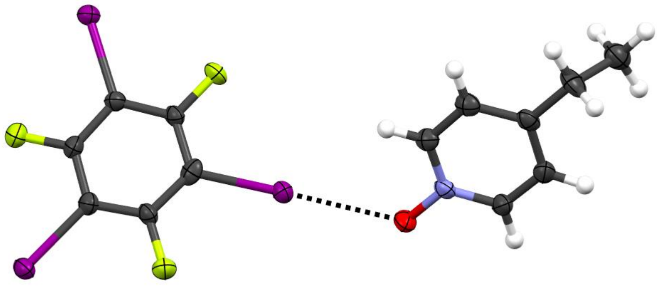

Figure S100. The X-ray crystal structure of **trIB-11** with the thermal displacement parameter at 50% probability level. The black broken line represents the halogen bond. Colour Key: green = fluorine, purple = iodine, blue = nitrogen, dark grey = carbon, white = hydrogen. **Notes**: The asymmetric unit consists of one **trIB** molecule and one PyNO molecule.

Crystal data for trIB-11 (crystallization solvent: $CHCl_3$): CCDC- 2337699, $C_{13}H_9F_3I_3NO$, M = 632.91 $gmol^{-1}$, colourless block, 0.05 × 0.05 × 0.03 mm, orthorhombic, space group *P n a 21*, a = 19.1952(4) Å, b = 4.54080(10) Å, c = 18.5099(3) Å, α = 90°, β = 90°, γ = 90°, V = 1613.35(6) Å^3, Z = 4, D_{calc} = 2.606 gcm^{-3}, F(000) = 1152, μ = 45.928 mm^{-1}, T = 120(1) K, θ_{max} = 66.692°, 19129 total reflections, 2613 with $I_o > 2\sigma(I_o)$, R_{int} = 0.0565, 2704 data, 191 parameters, 1 restraints, GooF = 1.019, R = 0.0384 and wR = 0.0967 [$I_o > 2\sigma(I_o)$], R = 0.0396 and wR = 0.0977 (all reflections), 1.413<dΔρ < -1.324 eÅ^{-3}.

11. Complex trIB-12

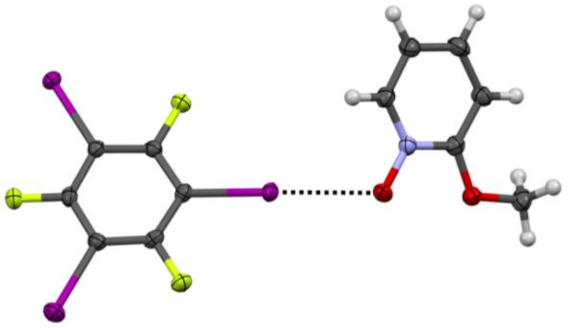

Figure S101. The X-ray crystal structure of **trIB-12** with the thermal displacement parameter at 50% probability level. The black broken line represents the halogen bond. Colour Key: green = fluorine, purple = iodine, blue = nitrogen, dark grey = carbon, white = hydrogen. **Notes**: The asymmetric unit consists of one 0.5 **trIB** molecule and one PyNO molecule.

Crystal data for trIB-12 (crystallization solvent: acetone): CCDC-2337700, $C_{18}H_{14}F_3I_3N_2O_4$, M = 760.01 gmol^{-1}, colourless block, 0.06 × 0.05 × 0.02 mm, orthorhombic, space group *Pbcn*, a = 29.0122(7) Å, b = 10.0014(2) Å, c = 7.4766(2) Å, α = 90°, β = 90°, γ = 90°, V = 2169.43(9) Å^3, Z = 4, D_{calc} = 2.327 gcm^{-3}, F(000) = 1416, μ = 34.439 mm^{-1}, T = 120(1) K, θ_{max} = 66.668°, 12254 total reflections, 1742 with $I_o > 2\sigma(I_o)$, R_{int} = 0.0405, 1918 data, 139 parameters, 0 restraints, GooF = 1.028, R = 0.0221 and wR = 0.0554 [$I_o > 2\sigma(I_o)$], R = 0.0248 and wR = 0.0568 (all reflections), 0.668<dΔρ < -0.779 eÅ^{-3}.

12. Complex trIB-13

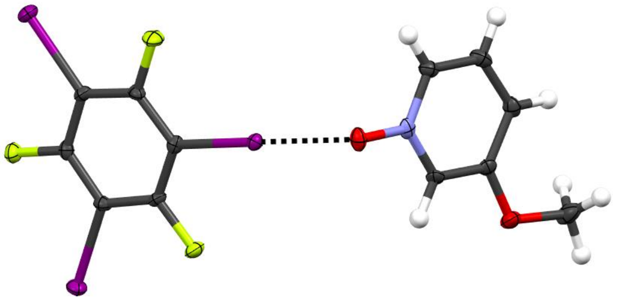

Figure S102. The X-ray crystal structure of **trIB-13** with the thermal displacement parameter at 50% probability level. The black broken line represents the halogen bond. Colour Key: green = fluorine, purple = iodine, blue = nitrogen, dark grey = carbon, white = hydrogen. **Notes**: The asymmetric unit consists of one **trIB** molecule and one PyNO molecule.

Crystal data for trIB-13 (crystallization solvent: $CHCl_3$): CCDC-2337701, $C_{12}H_7F_3I_3NO_2$, M = 634.89 gmol^{-1}, colourless block, 0.08 × 0.06 × 0.03 mm, monoclinic, space group *I2/a*, a = 18.3332(2) Å, b = 7.34610(10) Å, c = 23.7714(2) Å, α = 90°, β = 90.7380(10)°, γ = 90°, V = 2792.32(6) Å^3, Z = 8, D_{calc} = 2.635 gcm^{-3}, F(000) = 2304, μ = 46.347 mm^{-1}, T = 120(1) K, θ_{max} = 66.708°, 13771 total reflections, 2761 with $I_o > 2\sigma(I_o)$, R_{int} = 0.0296, 2837 data, 191 parameters, 0 restraints, GooF = 1.109, R = 0.0181 and wR = 0.0423 [$I_o > 2\sigma(I_o)$], R = 0.0189 and wR = 0.0427 (all reflections), 0.377<dΔρ < -0.704 eÅ^{-3}.

13. Complex trIB-15

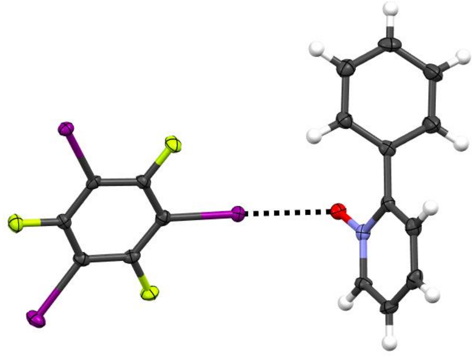

Figure S103. The X-ray crystal structure of **trIB-15** with the thermal displacement parameter at 50% probability level. The black broken line represents the halogen bond. Colour Key: green = fluorine, purple = iodine, blue = nitrogen, dark grey = carbon, white = hydrogen. **Notes**: The asymmetric unit consists of one **trIB** molecule and one PyNO molecule.

Crystal data for trIB-15 (crystallization solvent: $CHCl_3$): CCDC-2337702, $C_{17}H_9F_3I_3NO$, M = 680.95 gmol^{-1}, colourless block, 0.11 × 0.1 × 0.04 mm, monoclinic, space group *I2/a*, a = 21.4631(2) Å, b = 7.18440(10) Å, c = 24.5905(2) Å, α = 90°, β = 102.1610(10)°, γ = 90°, V = 3706.75(7) Å^3, Z = 8, D_{calc} = 2.440 gcm^{-3}, F(000) = 2496, μ = 40.058 mm^{-1}, T = 120(1) K, θ_{max} = 66.711°, 14983 total reflections, 3182 with $I_o > 2\sigma(I_o)$, R_{int} = 0.0397, 3287 data, 226 parameters, 0 restraints, GooF = 1.094, R = 0.0216 and wR = 0.0527 [$I_o > 2\sigma(I_o)$], R = 0.0225 and wR = 0.0531 (all reflections), 0.435<dΔρ < -0.796 eÅ^{-3}.

14. Complex trIB-16

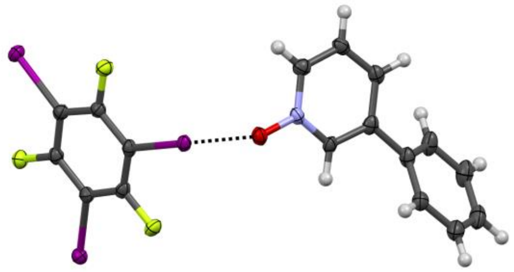

Figure S104. The X-ray crystal structure of **trIB-16** with the thermal displacement parameter at 50% probability level. The black broken line represents the halogen bond. Colour Key: green = fluorine, purple = iodine, blue = nitrogen, dark grey = carbon, white = hydrogen. **Notes**: The asymmetric unit consists of one **trIB** molecule, one PyNO molecule, and one acetone molecule.

Crystal data for trIB-16 (crystallization solvent: acetone): CCDC-2337703, $C_{20}H_{15}F_3I_3NO_2$, M = 739.03 gmol^{-1}, colourless block, 0.11 × 0.08 × 0.03 mm, monoclinic, space group *P2₁/c*, a = 13.67760(10) Å, b = 7.48300(10) Å, c = 21.4571(2) Å, α = 90°, β = 93.4020(10)°, γ = 90°, V = 2192.25(4) Å^3, Z = 4, D_{calc} = 2.239 gcm^{-3}, F(000) = 1376, μ = 33.971 mm^{-1}, T = 120(1) K, θ_{max} = 66.748°, 22199 total reflections, 3729 with $I_o > 2\sigma(I_o)$, R_{int} = 0.0505, 3878 data, 264 parameters, 0 restraints, GooF = 1.068, R = 0.0275 and wR = 0.0689 [$I_o > 2\sigma(I_o)$], R = 0.0286 and wR = 0.0697 (all reflections), 0.804<dΔρ < -0.994 eÅ^{-3}.

15. Complex trIB-17

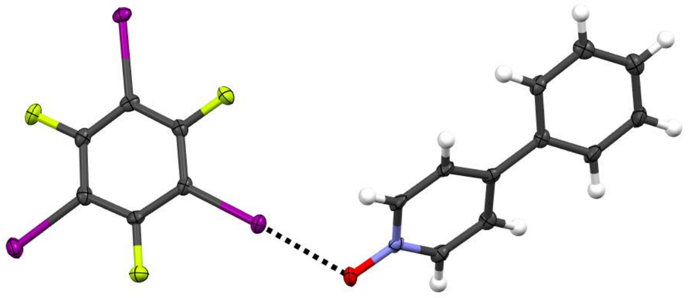

Figure S105. The X-ray crystal structure of **trIB-17** with the thermal displacement parameter at 50% probability level. The black broken line represents the halogen bond. Colour Key: green = fluorine, purple = iodine, blue = nitrogen, dark grey = carbon, white = hydrogen. **Notes**: The asymmetric unit consists of three **trIB** molecules, three PyNO molecules and one acetone molecule.

Crystal data for trIB-17 (crystallization solvent: acetone): CCDC-2337704, $C_{54}H_{33}F_9I_9N_3O_4$, M = 2100.93 gmol^{-1}, colourless block, 0.11 × 0.09 × 0.04 mm, triclinic, space group *P-1*, a = 11.08500(10) Å, b = 12.17010(10) Å, c = 23.1491(3) Å, α = 79.9660(10)°, β = 88.1700(10)°, γ = 78.1080(10)°, V = 3009.15(6) Å^3, Z = 2, D_{calc} = 2.319 gcm^{-3}, F(000) = 1936, μ = 37.047 mm^{-1}, T = 120(1) K, θ_{max} = 66.747°, 44787 total reflections, 10018 with $I_o > 2\sigma(I_o)$, R_{int} = 0.0415, 10683 data, 714 parameters, 0 restraints, GooF = 1.036, R = 0.0233 and wR = 0.0546 [$I_o > 2\sigma(I_o)$], R = 0.0255 and wR = 0.0556 (all reflections), 1.091<dΔρ < -1.101 eÅ^{-3}.

16. Complex trIB-18

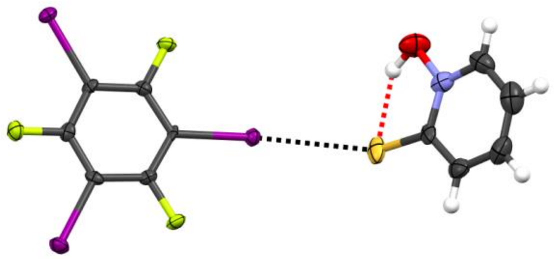

Figure S106. The X-ray crystal structure of **trIB-18** with the thermal displacement parameter at 50% probability level. The black broken line represents the halogen bond, and the red broken line represents the hydrogen bond. Colour Key: green = fluorine, purple = iodine, blue = nitrogen, dark grey = carbon, white = hydrogen. **Notes**: The asymmetric unit consists of one **trIB** molecule and one PyNO molecule.

Crystal data for trIB-18 (crystallization solvent: $CHCl_3$): CCDC-2337705, $C_{11}H_5F_3I_3NOS$, M = 636.92 gmol^{-1}, red block, 0.11 × 0.05 × 0.04 mm, monoclinic, space group *P2₁/n*, a = 8.00710(10) Å, b = 21.6474(4) Å, c = 9.1877(2) Å, α = 90°, β = 102.390(2)°, γ = 90°, V = 1555.44(5) Å^3, Z = 4, D_{calc} = 2.720 gcm^{-3}, F(000) = 1152, μ = 48.869 mm^{-1}, T = 120(1) K, θ_{max} = 66.750°, 15474 total reflections, 2623 with $I_o > 2\sigma(I_o)$, R_{int} = 0.0473, 2751 data, 182 parameters, 0 restraints, GooF = 1.066, R = 0.0344 and wR = 0.0881 [$I_o > 2\sigma(I_o)$], R = 0.0360 and wR = 0.0894 (all reflections), 2.720<dΔρ < -1.159 eÅ^{-3}.

17. Complex trIB-20

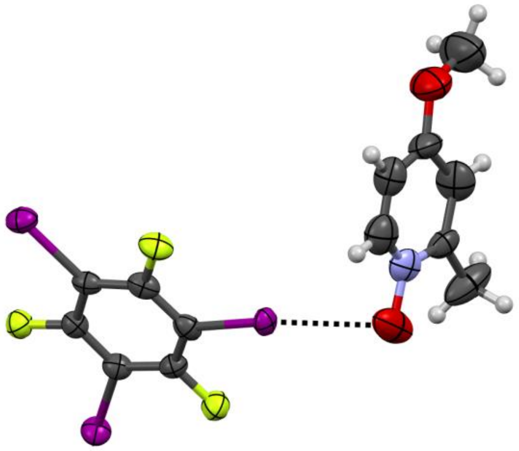

Figure S107. The X-ray crystal structure of **trIB-20** with the thermal displacement parameter at 50% probability level. The black broken line represents the halogen bond. Colour Key: green = fluorine, purple = iodine, blue = nitrogen, dark grey = carbon, white = hydrogen. **Notes**: The asymmetric unit consists of two **trIB** molecule and two PyNO molecules, one of which is disordered over two positions in a 50:50 ratio.

Crystal data for trIB-20 (crystallization solvent: $CHCl_3$): CCDC-2337706, $C_{13}H_9F_3I_3NO_2$, M = 648.91 gmol^{-1}, colourless block, 0.08 × 0.05 × 0.04 mm, monoclinic, space group *P2₁/c*, a = 20.2669(2) Å, b = 7.32790(10) Å, c = 23.2038(2) Å, α = 90°, β = 94.0350(10)°, γ = 90°, V = 3437.54(7) Å^3, Z = 8, D_{calc} = 2.508 gcm^{-3}, F(000) = 2368, μ = 43.182 mm^{-1}, T = 120(1) K, θ_{max} = 66.705°, 41036 total reflections, 5343 with $I_o > 2\sigma(I_o)$, R_{int} = 0.0647, 6093 data, 481 parameters, 136 restraints, GooF = 1.039, R = 0.0375 and wR = 0.0951 [$I_o > 2\sigma(I_o)$], R = 0.0434 and wR = 0.0988 (all reflections), 1.388<dΔρ< -1.181 eÅ^{-3}.

18. Complex trIB-21

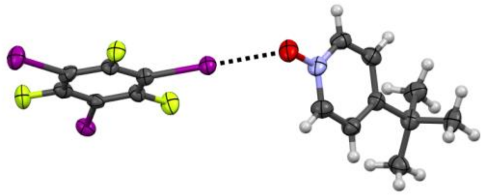

Figure S108. The X-ray crystal structure of **trIB-21** with the thermal displacement parameter at 50% probability level. The black broken line represents the halogen bond. Colour Key: green = fluorine, purple = iodine, blue = nitrogen, dark grey = carbon, white = hydrogen. **Notes**: The asymmetric unit consists of one **trIB** molecule and one PyNO molecule.

Crystal data for trIB-21 (crystallization solvent: $CHCl_3$): CCDC-2337707, $C_{15}H_{13}F_3I_3NO$, M = 660.96 gmol^{-1}, colourless block, 0.05 × 0.03 × 0.02 mm, monoclinic, space group *P2₁/n*, a = 10.0330(5) Å, b = 10.3506(5) Å, c = 18.4835(7) Å, α = 90°, β = 97.998(4)°, γ = 90°, V = 1900.80(15) Å^3, Z = 4, D_{calc} = 2.310 gcm^{-3}, F(000) = 1216, μ = 39.021 mm^{-1}, T = 120(1) K, θ_{max} = 66.745°, 18914 total reflections, 2727 with $I_o > 2\sigma(I_o)$, R_{int} = 0.1181, 3363 data, 211 parameters, 0 restraints, GooF = 1.052, R = 0.0506 and wR = 0.1364 [$I_o > 2\sigma(I_o)$], R = 0.0615 and wR = 0.1421 (all reflections), 1.388<dΔρ< -2.269 eÅ^{-3}.

19. Complex trIB-22

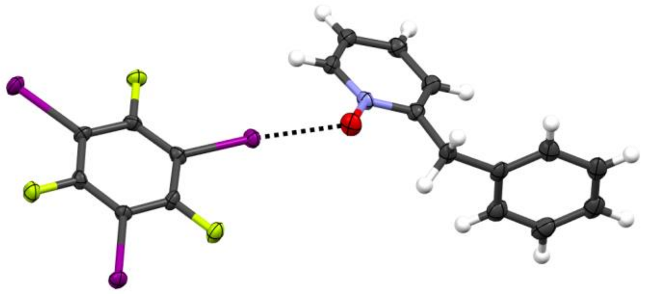

Figure S109. The X-ray crystal structure of **trIB-22** with the thermal displacement parameter at 50% probability level. The black broken line represents the halogen bond. Colour Key: green = fluorine, purple = iodine, blue = nitrogen, dark grey = carbon, white = hydrogen. **Notes**: The asymmetric unit consists of one **trIB** molecule and one PyNO molecule.

Crystal data for trIB-22 (crystallization solvent: $CHCl_3$): CCDC-2337708, $C_{18}H_{11}F_3I_3NO$, M = 694.98 gmol^{-1}, red block, 0.10 × 0.07 × 0.05 mm, monoclinic, space group *P2₁/c*, a = 9.1633(2) Å, b = 13.5795(3) Å, c = 15.6809(3) Å, α = 90°, β = 93.940(2)°, γ = 90°, V = 1555.44(5) Å^3, Z = 4, D_{calc} = 2.371 gcm^{-3}, F(000) = 1280, μ = 38.158 mm^{-1}, T = 120(1) K, θ_{max} = 66.748°, 22451 total reflections, 3209 with $I_o > 2\sigma(I_o)$, R_{int} = 0.0488, 3449 data, 235 parameters, 0 restraints, GooF = 1.034, R = 0.0275 and wR = 0.0696 [$I_o > 2\sigma(I_o)$], R = 0.0299 and wR = 0.0709 (all reflections), 0.858<dΔρ< -1.019 eÅ^{-3}.

20. Complex trIB-28

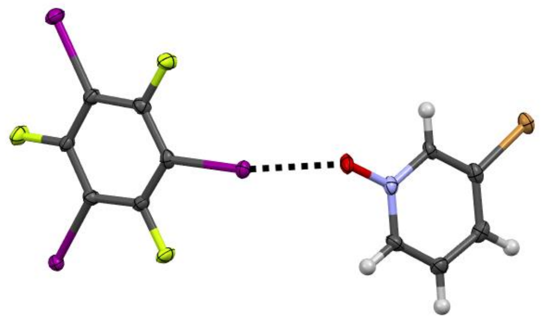

Figure S110. The X-ray crystal structure of **trIB-28** with the thermal displacement parameter at 50% probability level. The black broken line represents the halogen bond. Colour Key: green = fluorine, purple = iodine, blue = nitrogen, dark grey = carbon, white = hydrogen. **Notes**: The asymmetric unit consists of one **trIB** molecule and one PyNO molecule.

Crystal data for trIB-28 (crystallization solvent: $CHCl_3$): CCDC-2337709, $C_{11}H_4BrF_3I_3NO$, M = 683.76 gmol^{-1}, colourless block, 0.1 × 0.05 × 0.04 mm, triclinic, space group *P-1*, a = 7.8469(2) Å, b = 9.1546(3) Å, c = 11.2517(3) Å, α = 101.384(2)°, β = 103.582(2)°, γ = 90.634(2)°, V = 768.81(4) Å^3, Z = 2, D_{calc} = 2.954 gcm^{-3}, F(000) = 612, μ = 51.212 mm^{-1}, T = 120(1) K, θ_{max} = 66.734°, 10796 total reflections, 2604 with $I_o > 2\sigma(I_o)$, R_{int} = 0.0405, 2732 data, 181 parameters, 0 restraints, GooF = 1.029, R = 0.0251 and wR = 0.0617 [$I_o > 2\sigma(I_o)$], R = 0.0267 and wR = 0.0625 (all reflections), 1.033<dΔρ < -0.842 eÅ^{-3}.

21. Complex trIB-29

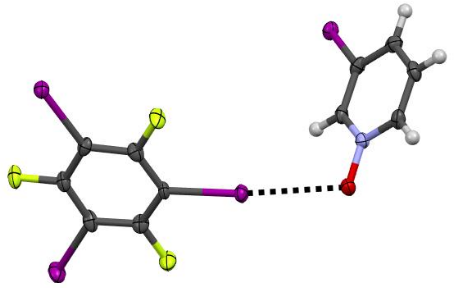

Figure S111. The X-ray crystal structure of **trIB-29** with the thermal displacement parameter at 50% probability level. The black broken line represents the halogen bond. Colour Key: green = fluorine, purple = iodine, blue = nitrogen, dark grey = carbon, white = hydrogen. **Notes**: The asymmetric unit consists of one **trIB** molecule and one PyNO molecule.

Crystal data for trIB-29 (crystallization solvent: $CHCl_3$): CCDC-2337710, $C_{11}H_4F_3I_4NO$, M = 730.75 gmol^{-1}, colourless block, 0.09 × 0.07 × 0.04 mm, triclinic, space group *P-1*, a = 8.0605(2) Å, b = 9.1505(2) Å, c = 11.3187(2) Å, α = 101.421(2)°, β = 103.162(2)°, γ = 92.095(2)°, V = 793.89(3) Å^3, Z = 2, D_{calc} = 3.057 gcm^{-3}, F(000) = 648, μ = 66.744 mm^{-1}, T = 120(1) K, θ_{max} = 66.740°, 10553 total reflections, 2678 with $I_o > 2\sigma(I_o)$, R_{int} = 0.0546, 2818 data, 181 parameters, 0 restraints, GooF = 1.084, R = 0.0387 and wR = 0.0976 [$I_o > 2\sigma(I_o)$], R = 0.0404 and wR = 0.0992 (all reflections), 1.729 <dΔρ < -1.591 eÅ^{-3}.

22. Complex trIB-30

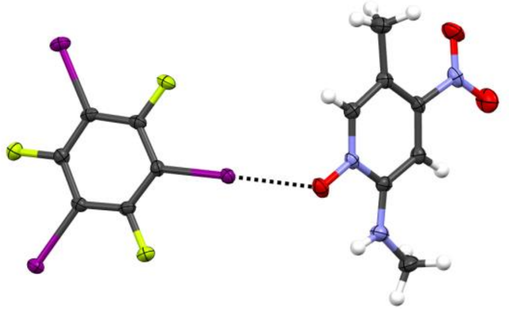

Figure S112. The X-ray crystal structure of **trIB-30** with the thermal displacement parameter at 50% probability level. The black broken line represents the halogen bond. Colour Key: green = fluorine, purple = iodine, blue = nitrogen, dark grey = carbon, white = hydrogen. **Notes**: The asymmetric unit consists of one **trIB** molecule and one PyNO molecule.

Crystal data for trIB-30 (crystallization solvent: $CHCl_3$): CCDC-2337711, $C_{13}H_9F_3I_3N_3O_3$, M = 692.93 gmol^{-1}, yellow block, 0.10 × 0.07 × 0.05 mm, triclinic, space group *P-1*, a = 7.62400(10) Å, b = 9.19750(10) Å, c = 13.5984(2) Å, α = 96.3260(10)°, β = 91.9580(10)°, γ = 107.8210(10)°, V = 899.94(2) Å^3, Z = 2, D_{calc} = 2.557 gcm^{-3}, F(000) = 636, μ = 41.381 mm^{-1}, T = 120(1) K, θ_{max} = 66.740°, 13842 total reflections, 3055 with $I_o > 2\sigma(I_o)$, R_{int} = 0.0297, 3169 data, 228 parameters, 0 restraints, GooF = 1.079, R = 0.0202 and wR = 0.0505 [$I_o > 2\sigma(I_o)$], R = 0.0213 and wR = 0.0511 (all reflections), 0.622 <dΔρ < -0.662 eÅ^{-3}.

23. Complex trIB-32

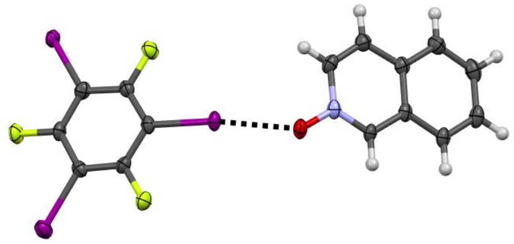

Figure S113. The X-ray crystal structure of **trIB-32** with the thermal displacement parameter at 50% probability level. The black broken line represents the halogen bond. Colour Key: green = fluorine, purple = iodine, blue = nitrogen, dark grey = carbon, white = hydrogen. **Notes**: The asymmetric unit consists of one **trIB** molecule and one PyNO molecule.

Crystal data for trIB-32 (crystallization solvent: $CHCl_3$): CCDC-2337712, $C_{15}H_7F_3I_3NO$, M = 654.92 gmol^{-1}, colourless block, 0.10 × 0.09 × 0.07 mm, triclinic, space group *P-1*, a = 8.7896(4) Å, b = 9.4920(4) Å, c = 11.3689(4) Å, α = 81.624(3)°, β = 72.581(4)°, γ = 71.396(4)°, V = 856.41(7) Å^3, Z = 2, D_{calc} = 2.540 gcm^{-3}, F(000) = 596, μ = 43.303 mm^{-1}, T = 120(1) K, θ_{max} = 66.745°, 12787 total reflections, 2904 with $I_o > 2\sigma(I_o)$, R_{int} = 0.0503, 3042 data, 208 parameters, 0 restraints, GooF = 1.077, R = 0.0398 and wR = 0.0976 [$I_o > 2\sigma(I_o)$], R = 0.0411 and wR = 0.0988 (all reflections), 2.343 <dΔρ < -1.375 eÅ^{-3}.

2.8. Pyridine N-oxides crystallized from acetone:water mixture

1. Structure of 4

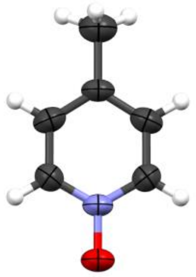

Figure S114. The X-ray crystal structure of **4** with the thermal displacement parameter at 50% probability level. Colour Key: blue = nitrogen, dark grey = carbon, white = hydrogen. **Notes**: The asymmetric unit consists of one 0.5 PyNO molecule.

Crystal data for 4 (crystallization solvent: Acetone:H_2O): CCDC-2337775, $C_6H_{10}NO$, M = 112.15 gmol^{-1}, colourless block, 0.13 × 0.11 × 0.11 mm, tetragonal, space group *I4₁/amd*, a = 7.921(3) Å, b = 7.921(3) Å, c = 19.587(4) Å, α = 90°, β = 90°, γ = 90°, V = 1228.9(10) Å^3, Z = 8, D_{calc} = 1.212 gcm^{-3}, F(000) = 488, μ = 0.083 mm^{-1}, T = 170 K, θ_{max} = 25.243°, 3914 total reflections, 176 with $I_o > 2\sigma(I_o)$, R_{int} = 0.1009, 324 data, 29 parameters, 0 restraints, GooF = 1.087, R = 0.0756 and wR = 0.2174 [$I_o > 2\sigma(I_o)$], R = 0.1350 and wR = 0.2632 (all reflections), 0.268 <dΔρ < -0.227 eÅ^{-3}.

2. Structure of 6-H_2O

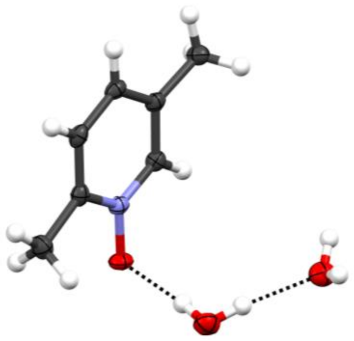

Figure S115. The X-ray crystal structure of **6-H_2O** with the thermal displacement parameter at 50% probability level. Colour Key: blue = nitrogen, dark grey = carbon, white = hydrogen. **Notes**: The asymmetric unit consists of one PyNO molecule and two water molecules.

Crystal data for 6-H_2O (crystallization solvent: Acetone:H_2O): CCDC- 2337776, $C_7H_{13}NO_3$, M = 159.18 gmol^{-1}, colourless block, 0.07 × 0.06 × 0.04 mm, monoclinic, space group *P2₁/c*, a = 6.7369(2) Å, b = 13.9982(4)Å, c = 9.2130(3)Å, α = 90°, β = 105.295(3)°, γ = 90°, V = 838.05(5) Å^3, Z = 4, D_{calc} = 1.262 gcm^{-3}, F(000) = 344, μ = 0.822 mm^{-1}, T = 120 K, θ_{max} = 66.734°, 6492 total reflections, 1312 with $I_o > 2\sigma(I_o)$, R_{int} = 0.0252, 1479 data, 112 parameters, 0 restraints, GooF = 1.055, R = 0.0306 and wR = 0.0833 [$I_o > 2\sigma(I_o)$], R = 0.0356 and wR = 0.0869 (all reflections), $0.150 < d\Delta\rho < -0.236$ eÅ^{-3}.

3. Structure of 9-H_2O

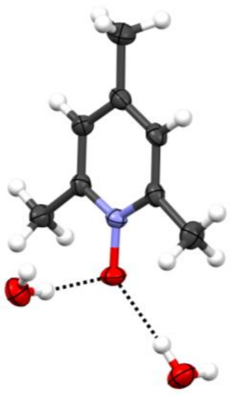

Figure S116. The X-ray crystal structure of **9-H_2O** with the thermal displacement parameter at 50% probability level. Colour Key: blue = nitrogen, dark grey = carbon, white = hydrogen. **Notes**: The asymmetric unit consists of three PyNO molecules and three water molecules. For viewing clarity, just the 1:2 PyNO:H_2O hydrogen-bonded structure is shown. **9-H_2O** and **9a-H_2O** structures were isolated from the same bulk sample.

Crystal data for 9-H_2O (crystallization solvent: Acetone:H_2O): CCDC-2337777, $C_8H_{13}NO_2$, M = 155.19 gmol^{-1}, colourless block, 0.19 × 0.16 × 0.11 mm, monoclinic, space group Cc, a = 8.6104(17) Å, b = 14.570(3) Å, c = 20.342(4) Å, α = 90°, β = 92.98(3)°, γ = 90°, V = 2548.7(9) Å^3, Z = 12, D_{calc} = 1.213 gcm^{-3}, F(000) = 1008, μ = 0.087 mm^{-1}, T = 170 K, θ_{max} = 25.244°, 10474 total reflections, 3314 with $I_o > 2\sigma(I_o)$, R_{int} = 0.0413, 3937 data, 316 parameters, 2 restraints, GooF = 1.043, R = 0.0428 and wR = 0.0933 [$I_o > 2\sigma(I_o)$], R = 0.0562 and wR = 0.0992 (all reflections), $0.146 < d\Delta\rho < -0.230$ eÅ^{-3}.

4. Structure of 9a-H_2O

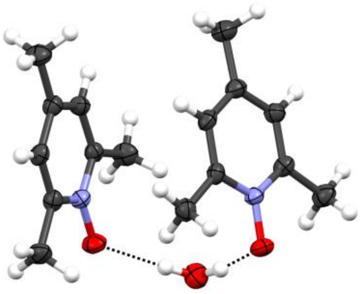

Figure S117. The X-ray crystal structure of **9a-H_2O** with the thermal displacement parameter at 50% probability level. Colour Key: blue = nitrogen, dark grey = carbon, white = hydrogen. **Notes**: The asymmetric unit consists of two PyNO molecules and one water molecule. **9-H_2O** and **9a-H_2O** structures were isolated from the same bulk sample.

Crystal data for 9a-H_2O (crystallization solvent: Acetone:H_2O): CCDC-2337778, $C_{16}H_{24}N_2O_3$, M = 292.37 gmol^{-1}, colourless block, 0.16 × 0.15 × 0.13 mm, monoclinic, space group Cc, a = 14.381(3) Å, b = 8.4750(17) Å, c = 13.727(3) Å, α = 90°, β = 107.75(3)°, γ = 90°, V = 1593.5(6) Å^3, Z = 4, D_{calc} = 1.219 gcm^{-3}, F(000) = 632, μ = 0.084 mm^{-1}, T = 170 K, θ_{max} = 25.237°, 17024 total reflections, 2462 with $I_o > 2\sigma(I_o)$, R_{int} = 0.0469, 2860 data, 203 parameters, 2 restraints, GooF = 1.045, R = 0.0354 and wR = 0.0776 [$I_o > 2\sigma(I_o)$], R = 0.0463 and wR = 0.0832 (all reflections), 0.139 <dΔρ < -0.162 eÅ^{-3}.

5. Structure of 12-H_2O

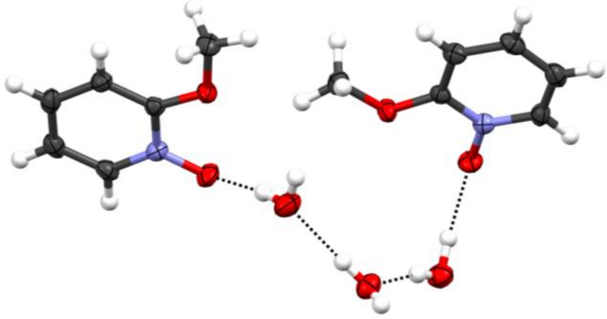

Figure S118. The X-ray crystal structure of **12-H_2O** with the thermal displacement parameter at 50% probability level. Colour Key: blue = nitrogen, dark grey = carbon, white = hydrogen. **Notes**: The asymmetric unit consists of two PyNO molecules and three water molecules.

Crystal data for 12-H_2O (crystallization solvent: Acetone:H_2O): CCDC-2337779, $C_{12}H_{20}N_2O_7$, M = 304.30 gmol^{-1}, colourless plate, 0.13 × 0.07 × 0.04 mm, triclinic, space group Cc, a = 6.5062(13) Å, b = 6.7831(14) Å, c = 17.340(4) Å, α = 79.70(3)°, β = 88.96(3)°, γ = 73.57(3)°, V = 721.7(3) Å^3, Z = 2, D_{calc} = 1.400 gcm^{-3}, F(000) = 324, μ = 0.116 mm^{-1}, T = 170 K, θ_{max} = 25.245°, 6874 total reflections, 1819 with $I_o > 2\sigma(I_o)$, R_{int} = 0.0501, 2600 data, 205 parameters, 2 restraints, GooF = 1.042, R = 0.0523 and wR = 0.1147 [$I_o > 2\sigma(I_o)$], R = 0.0847 and wR = 0.1300 (all reflections), 0.193 <dΔρ < -0.303 eÅ^{-3}.

6. Structure of 14-H_2O

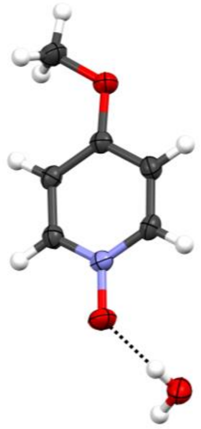

Figure S119. The X-ray crystal structure of **14-H_2O** with the thermal displacement parameter at 50% probability level. Colour Key: blue = nitrogen, dark grey = carbon, white = hydrogen. **Notes**: The asymmetric unit consists of one PyNO molecule and one water molecule.

Crystal data for 14-H_2O (crystallization solvent: Acetone:H_2O): CCDC-2337780, $C_6H_9NO_3$, M = 143.14 gmol^{-1}, colourless block, 0.14 × 0.13 × 0.12 mm, orthorhombic, space group Pbca, a = 12.035(2)Å, b = 7.0570(14) Å, c = 16.271(3) Å, α = 90°, β = 90°, γ = 90°, V = 1381.9(5) Å^3, Z = 8, D_{calc} = 1.376 gcm^{-3}, F(000) = 608, μ = 0.111 mm^{-1}, T = 170 K, θ_{max} = 25.250°, 31136 total reflections, 972 with $I_o > 2\sigma(I_o)$, R_{int} = 0.0776, 1258 data, 95 parameters, 0 restraints, GooF = 1.030, R = 0.0385 and wR = 0.0881 [$I_o > 2\sigma(I_o)$], R = 0.0553 and wR = 0.0957 (all reflections), 0.142<dΔρ < -0.194 eÅ^{-3}.

7. Structure of 15

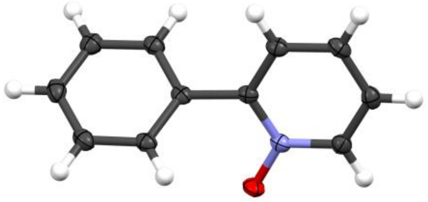

Figure S120. The X-ray crystal structure of **15** with the thermal displacement parameter at 50% probability level. Colour Key: blue = nitrogen, dark grey = carbon, white = hydrogen. **Notes**: The asymmetric unit consists of one PyNO molecule.

Crystal data for 15 (crystallization solvent: Acetone:H_2O): CCDC-2337781, $C_{11}H_9NO$, M = 171.19 gmol^{-1}, colourless plate, 0.08× 0.07 × 0.06 mm, monoclinic, space group $P2_1/c$, a = 5.7195(9)Å, b = 23.356(2) Å, c = 6.8110(9) Å, α = 90°, β = 114.290(18)°, γ = 90°, V = 829.3(2) Å^3, Z = 4, D_{calc} = 1.371 gcm^{-3}, F(000) = 360, μ = 0.710 mm^{-1}, T = 120 K, θ_{max} = 66.726°, 4594 total reflections, 1179 with $I_o > 2\sigma(I_o)$, R_{int} = 0.0443, 1467 data, 118 parameters, 0 restraints, GooF = 1.091, R = 0.0383 and wR = 0.0895 [$I_o > 2\sigma(I_o)$], R = 0.0512 and wR = 0.0987 (all reflections), 0.182<dΔρ < -0.191 eÅ^{-3}.

8. Structure of 16

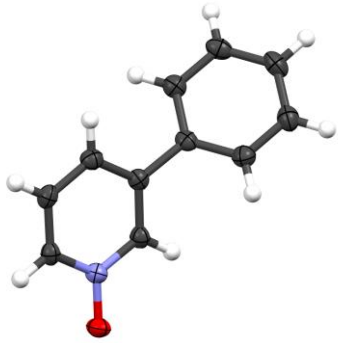

Figure S121. The X-ray crystal structure of **16** with the thermal displacement parameter at 50% probability level. Colour Key: blue = nitrogen, dark grey = carbon, white = hydrogen. **Notes**: The asymmetric unit consists of one PyNO molecule.

Crystal data for 16 (crystallization solvent: Acetone:H_2O): CCDC-2337782, $C_{11}H_9NO$, M = 171.19 gmol^{-1}, colourless plate, 0.14× 0.11 × 0.1 mm, monoclinic, space group $P2_1/n$, a = 5.9607(12)Å, b = 20.795(4) Å, c = 7.2438(14) Å, α = 90°, β = 109.79(3)°, γ = 90°, V = 844.9(3) Å^3, Z = 4, D_{calc} = 1.346 gcm^{-3}, F(000) = 360, μ = 0.087 mm^{-1}, T = 120 K, θ_{max} = 25.239°, 5859 total reflections, 1124 with $I_o > 2\sigma(I_o)$, R_{int} = 0.0418, 1522 data, 118 parameters, 0 restraints, GooF = 1.030, R = 0.0394 and wR = 0.0866 [$I_o > 2\sigma(I_o)$], R = 0.0615 and wR = 0.0965 (all reflections), 0.163<dΔρ < -0.163 eÅ^{-3}.

9. Structure of 17-H_2O

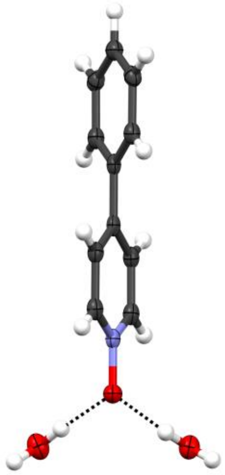

Figure S122. The X-ray crystal structure of **17** with the thermal displacement parameter at 50% probability level. Colour Key: blue = nitrogen, dark grey = carbon, white = hydrogen. **Notes**: The asymmetric unit consists of one 0.5 PyNO molecule and two 0.5 water molecules.

Crystal data for 17-H_2O (crystallization solvent: Acetone:H_2O): CCDC-2337783, $C_{11}H_{13}NO_3$, M = 207.22 gmol^{-1}, colourless plate, 0.18× 0.041 × 0.03 mm, orthorhombic, space group P*nna*, a = 12.0500(4)Å, b = 7.3638(2) Å, c = 11.4078(4) Å, α = 90°, β = 90°, γ = 90°, V = 1012.25(6) Å^3, Z = 4, D_{calc} = 1.360 gcm^{-3}, F(000) = 440, μ = 0.823 mm^{-1}, T = 123 K, θ_{max} = 66.654°, 4570 total reflections, 698 with $I_o > 2\sigma(I_o)$, R_{int} = 0.0385, 899 data, 80 parameters, 0 restraints, GooF = 1.106, R = 0.0421 and wR = 0.1277 [$I_o > 2\sigma(I_o)$], R = 0.0511 and wR = 0.1368 (all reflections), 0.143<dΔρ < -0.226 eÅ^{-3}.

10. Structure of 18

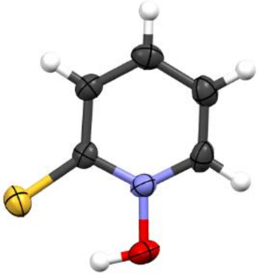

Figure S123. The X-ray crystal structure of **18** with the thermal displacement parameter at 50% probability level. Colour Key: blue = nitrogen, dark grey = carbon, white = hydrogen. **Notes**: The asymmetric unit consists of one PyNO molecule. **18** and **18a** structures were isolated from the same bulk sample.

Crystal data for 18 (crystallization solvent: Acetone:H_2O): CCDC-2337784, C_5H_5NOS, M = 127.16 gmol^{-1}, colourless block, 0.12× 0. 1 × 0.09 mm, monoclinic, space group P2$_1$/n, a = 6.2399(12) Å, b = 7.9396(16) Å, c = 11.697(2) Å, α = 90°, β = 92.19(3) °, γ = 90°, V = 579.1(2) Å^3, Z = 4, D_{calc} = 1.459 gcm^{-3}, F(000) = 264, μ = 0.445 mm^{-1}, T = 170 K, θ_{max} = 25.235°, 2498 total reflections, 681 with $I_o > 2\sigma(I_o)$, R_{int} = 0.0458, 1046 data, 77 parameters, 0 restraints, GooF = 1.035, R = 0.0427 and wR = 0.0901 [$I_o > 2\sigma(I_o)$], R = 0.0808 and wR = 0.1030 (all reflections), 0.246 <dΔρ < - -0.292 eÅ^{-3}.

11. Structure of 18a

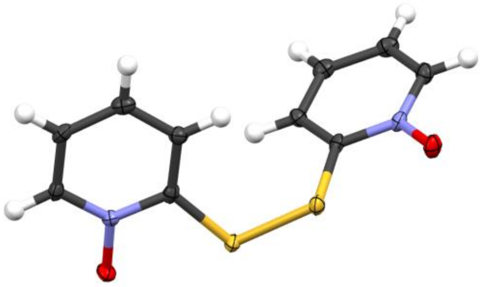

Figure S124. The X-ray crystal structure of **18a** with the thermal displacement parameter at 50% probability level. Colour Key: blue = nitrogen, dark grey = carbon, white = hydrogen. **Notes**: The asymmetric unit consists of one 0.5 PyNO molecule. **18** and **18a** structures were isolated from the same bulk sample.

Crystal data for 18a (crystallization solvent: Acetone:H_2O): CCDC-2337785, $C_{10}H_8N_2O_2S_2$, M = 252.30 gmol^{-1}, colourless block, 0.12× 0. 11 × 0.1 mm, triclinic, space group P-1, a = 7.0464(7) Å, b = 7.0662(11) Å, c = 10.7711(16) Å, α = 86.688(13)°, β = 73.575(11) °, γ = 88.887(11)°, V = 513.56(13) Å^3, Z = 2, D_{calc} = 1.632 gcm^{-3}, F(000) = 260, μ = 0.502 mm^{-1}, T = 170 K, θ_{max} = 25.246°, 2935 total reflections, 1541 with $I_o > 2\sigma(I_o)$, R_{int} = 0.0480, 1853 data, 145 parameters, 0 restraints, GooF = 0.986, R = 0.0483 and wR = 0.1376 [$I_o > 2\sigma(I_o)$], R = 0.0593 and wR = 0.1516 (all reflections), 0.553 <dΔρ < -0.605 eÅ^{-3}.

12. Structure of 19

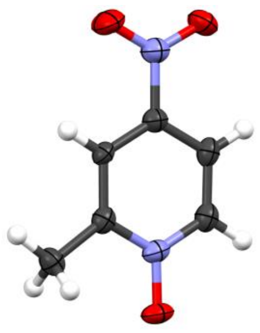

Figure S125. The X-ray crystal structure of **19** with the thermal displacement parameter at 50% probability level. Colour Key: blue = nitrogen, dark grey = carbon, white = hydrogen. **Notes**: The asymmetric unit consists of one 0.5 PyNO molecule.

Crystal data for 19 (crystallization solvent: Acetone:H_2O): CCDC-2337786, $C_6H_6N_2O_3$, M = 154.13 gmol^{-1}, yellow block, 0.13× 0. 12 × 0.12 mm, orthorhombic, space group Pbcm, a = 8.6809(17) Å, b = 12.377(3) Å, c = 6.2069(12) Å, α = 90°, β = 90°, γ = 90°, V = 666.9(2) Å^3, Z = 4, D_{calc} = 1.535 gcm^{-3}, F(000) = 320, μ = 0.126 mm^{-1}, T = 170 K, θ_{max} = 25.246°, 2125 total reflections, 560 with $I_o > 2\sigma(I_o)$, R_{int} = 0.0370, 668 data, 67 parameters, 0 restraints, GooF = 1.084, R = 0.0429 and wR = 0.1058 [$I_o > 2\sigma(I_o)$], R = 0.0526 and wR = 0.1112 (all reflections), 0.392<dΔρ < -0.242 eÅ^{-3}.

13. Structure of 20-H_2O

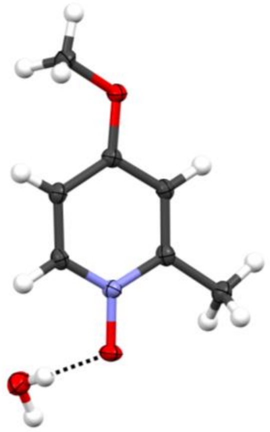

Figure S126. The X-ray crystal structure of **19** with the thermal displacement parameter at 50% probability level. Colour Key: blue = nitrogen, dark grey = carbon, white = hydrogen. **Notes**: The asymmetric unit consists of one PyNO molecule and one water molecule.

Crystal data for 20-H_2O (crystallization solvent: Acetone:H_2O): CCDC-2337787, $C_7H_{11}NO_3$, M = 157.17 gmol^{-1}, colourless block, 0.08× 0.05 × 0.03 mm, triclinic, space group P-1, a = 6.4989(2) Å, b = 7.4179(3) Å, c = 9.2625(3) Å, α = 103.962(3)°, β = 99.566(3)°, γ = 113.214(4)°, V = 380.78(3) Å^3, Z = 2, D_{calc} = 1.371 gcm^{-3}, F(000) = 168, μ = 0.904 mm^{-1}, T = 120 K, θ_{max} = 66.729°, 4451 total reflections, 1210 with $I_o > 2\sigma(I_o)$, R_{int} = 0.0240, 1352 data, 105 parameters, 0 restraints, GooF = 1.050, R = 0.0325 and wR = 0.0885 [$I_o > 2\sigma(I_o)$], R = 0.0364 and wR = 0.0911 (all reflections), 0.152<dΔρ < -0.244 eÅ^{-3}.

14. Structure of 22

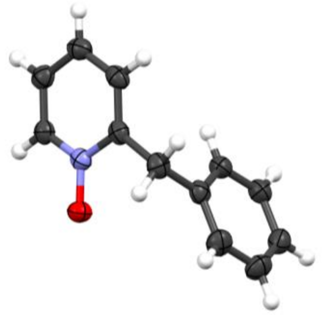

Figure S127. The X-ray crystal structure of **22** with the thermal displacement parameter at 50% probability level. Colour Key: blue = nitrogen, dark grey = carbon, white = hydrogen. **Notes**: The asymmetric unit consists of one PyNO molecule.

Crystal data for 22 (crystallization solvent: Acetone:H_2O): CCDC-2337788, $C_{12}H_{11}NO$, M = 185.22 gmol^{-1}, colourless block, 0.2× 0.16 × 0.14 mm, monoclinic, space group $P2_1/c$, a = 9.5321(2) Å, b = 11.11280(10) Å, c = 9.9897(2) Å, α = 90°, β = 116.239(2)°, γ = 90°, V = 949.15(3) Å^3, Z = 4, D_{calc} = 1.296 gcm^{-3}, F(000) = 392, μ = 0.659 mm^{-1}, T = 120 K, θ_{max} = 66.733°, 6224 total reflections, 1602 with $I_o > 2\sigma(I_o)$, R_{int} = 0.0231, 1684 data, 127 parameters, 0 restraints, GooF = 1.046, R = 0.0351 and wR = 0.0932 [$I_o > 2\sigma(I_o)$], R = 0.0362 and wR = 0.0944 (all reflections), 0.128<dΔρ < -0.216 eÅ^{-3}.

15. Structure of 30

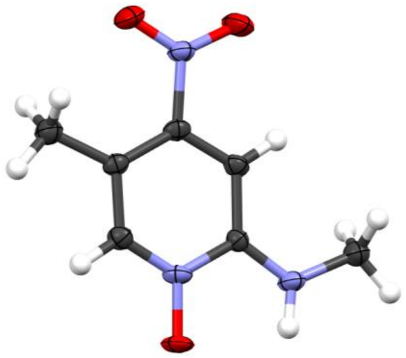

Figure S128. The X-ray crystal structure of **30** with the thermal displacement parameter at 50% probability level. Colour Key: blue = nitrogen, dark grey = carbon, white = hydrogen. **Notes**: The asymmetric unit consists of one PyNO molecule.

Crystal data for 30 (crystallization solvent: Acetone:H_2O): CCDC-2337789, $C_7H_9N_3O_3$, M = 183.17 gmol^{-1}, yellow needle, 0.16× 0.13 × 0.08 mm, monoclinic, space group $P2_1/c$, a = 11.1366(4) Å, b = 10.4053(6) Å, c = 7.1918(5) Å, α = 90°, β = 101.065(5)°, γ = 90°, V = 817.89(8) Å^3, Z = 4, D_{calc} = 1.488 gcm^{-3}, F(000) = 384, μ = 0.119 mm^{-1}, T = 123 K, θ_{max} = 25.249°, 7192 total reflections, 1022 with $I_o > 2\sigma(I_o)$, R_{int} = 0.0656, 1465 data, 120 parameters, 0 restraints, GooF = 1.038, R = 0.0598 and wR = 0.1533 [$I_o > 2\sigma(I_o)$], R = 0.0849 and wR = 0.1623 (all reflections), 0.622<dΔρ < -0.333 eÅ^{-3}.

16. Structure of 32

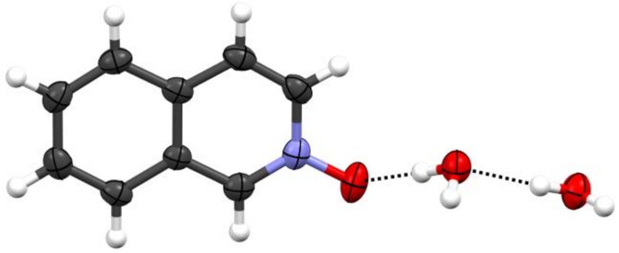

Figure S129. The X-ray crystal structure of **32** with the thermal displacement parameter at 50% probability level. Colour Key: blue = nitrogen, dark grey = carbon, white = hydrogen. **Notes**: The asymmetric unit consists of one PyNO molecule and two water molecules.

Crystal data for 32 (crystallization solvent: Acetone:H_2O): CCDC-2337790, $C_9H_{11}NO_3$, M = 181.19 gmol^{-1}, colourless block, 0.12× 0.12 × 0.12 mm, orthorhombic, space group $P2_12_12_1$, a = 6.6580(13) Å, b = 9.923(2) Å, c = 13.543(3) Å, α = 90°, β = 90°, γ = 90°, V = 894.7(3) Å^3, Z = 4, D_{calc} = 1.345 gcm^{-3}, F(000) = 384, μ = 0.102 mm^{-1}, T = 170 K, θ_{max} = 25.246°, 6717 total reflections, 1264 with $I_o > 2\sigma(I_o)$, R_{int} = 0.0433, 1603 data, 129 parameters, 0 restraints, GooF = 1.021, R = 0.0343 and wR = 0.0687 [$I_o > 2\sigma(I_o)$], R = 0.0523 and wR = 0.0750 (all reflections), 0.132<dΔρ < -0.106 eÅ^{-3}.

Table S1. Halogen bond parameters of **PfIB**-PyNO complexes

**Code**	Donor:acceptor ratio in AU§	N–O Denticity	N–O (Å)	C–I (Å)	Δ(C–I)♦ (Å)	I···O (Å)	∠C–I···O (°)	Δθ (°)†	∠N–O···I (°)	N···I (Å)	C···O (Å)
**PfIB-3**	1:1	1XB; 1HB▲	1.324(4)	2.109(3)	0.032	2.734(2)	173.80(11)	6.2	123.59(18)	--	4.837(5)
**PfIB-8**	5:3	2XB	1.331(3)	2.095(3)	0.018	2.760(2)	175.49(9)	4.51	131.96(18)	--	4.852(4)
				2.104(3)	0.027	2.765(2)	175.03(13)	4.97	126.28(18)	--	4.865(4)
		2XB	1.326(3)	2.094(3)	0.017	2.722(2)	172.61(10)	7.39	130.93(18)	--	4.806(4)
				2.098(3)	0.021	2.804(2)	173.99(14)	6.01	126.58(18)	--	4.895(4)
		1XB*	1.35(3)	2.102(4)	0.025	2.65(2)	168.5(4)	11.5	131.5(17)	--	4.73(2)
**PfIB-13**	1:1	1XB; 2HB	1.318(3)	2.096(3)	0.019	2.756(2)	175.46(10)	4.54	128.41(19)	--	4.849(4)
**PfIB-14**	1:1	1XB; 3HB	1.341(3)	2.102(3)	0.025	2.663(2)	175.87(9)	4.13	108.65(16)	3.343	4.762(4)
**PfIB-15**	1:1	1XB; 2HB	1.317(3)	2.091(2)	0.014	2.841(2)	167.02(8)	12.98	129.57(12)	--	4.901(3)
**PfIB-16**	2:2	1XB, 2HB	1.324(4)	2.090(9)	0.013	2.720(7)	166.6(3)	13.4	134.7(5)	--	4.779(13)
		1XB; 2HB	1.302(11)	2.070(8)	-0.007	2.755(7)	175.0(3)	5.0	127.2(6)	--	4.82(1)
**PfIB-17**	1:1	1XB; 1HB	1.332(10)	2.107(9)	0.03	2.668(7)	176.4(3)	3.6	113.7(3)	3.428	4.773(12)
**PfIB-20**	1:1	1XB; 2HB	1.336(3)	2.116(3)	0.039	2.648(2)	179.39(10)	0.61	109.51(15)	3.341	4.764(3)
**PfIB-31**	2:2	1XB; 1HB	1.313(13)	2.091(10)	0.014	2.737(7)	176.5(3)	3.4	106.1(5)	3.348	4.826(12)
		1XB; 1HB	1.326(13)	2.079(11)	0.002	2.748(8)	176.6(4)	3.4	101.1(5)	3.272	4.825(13)
**PfIB-32**	1:1	1XB; 1HB	1.317(8)	2.095(7)	0.018	2.708(5)	177.8(3)	2.2	127.3(5)	--	4.802(8)

§AU = Asymmetric unit; ♦The uncomplexed **PfIB**'s C–I bond distance is 2.077(4)Å (CCDC code = ZAHGAQ). Δ(C–I) = $(C–I)_{complexed}$ – $(C–I)_{uncomplexed}$; †Δ(θ) = 180 – $\theta_{complex}$ ▲1XB, 1HB denotes that the N-oxide oxygen is involved in one halogen bond and one hydrogen bond; *This monodentate PyNO is disordered over two positions in a 50:50 ratio.

Table S2. Halogen bond parameters of **oDIB**-PyNO complexes

Code	Donor:acceptor ratio in AU§	N–O Denticity	N–O (Å)	C–I (Å)	Δ(C–I)♦ (Å)	I···O (Å)	∠C–I···O (°)	Δθ (°)†	∠N–O···I (°)	N···I (Å)	C···O (Å)
**oDIB-1**	1:1	2XB	1.313(4)	2.105(4)	0.019	2.793(3)	168.03(14)	11.97	130.4(3)	--	4.872(5)
				2.093(5)	0.007	2.794(3)	171.63(14)	8.37	115.0(3)	--	4.875(6)
**oDIB-2**	2:2	2XB	1.314(12)	2.085(10)	-0.001	2.801(7)	171.8(3)	8.2	107.8(5)	3.439	4.874(12)
				2.107(9)	0.021	2.756(8)	173.7(3)	6.3	122.1(6)	--	4.856(11)
		2XB	1.321(12)	2.085(10)	-0.001	2.762(7)	177.5(3)	2.5	112.0(6)	3.479	4.845(12)
				2.115(9)	0.029	2.774(8)	175.7(3)	4.3	116.0(6)	--	4.886(11)
**oDIB-3**	1:1	2XB	1.348(10)	2.079(9)	-0.007	2.788(7)	172.0(3)	8	117.2(5)	--	4.856(12)
				2.106(10)	0.02	2.754(7)	175.6(3)	4.4	108.4(5)	3.428	4.857(12)
**oDIB-4**	2:2	2XB, 1HB▲	1.332(5)	2.106(4)	0.02	2.831(3)	169.73(13)	10.27	125.3(3)	--	4.918(6)
				2.109(4)	0.023	2.689(3)	172.56(14)	7.44	124.2(3)	--	4.787(5)
		2XB, 1HB	1.320(5)	2.087(4)*	0.001	I···O passive	--	--	--	--	--
				2.104(4)	0.018	2.842(3)	176.02(14)	3.98	114.2(2)	--	4.943(6)
**oDIB-4a**	1:1	2XB, 1HB	1.331(6)	2.105(5)	0.019	2.852(4)	167.67(17)	12.33	123.5(4)	--	4.929(7)
				2.106(5)	0.02	2.861(4)	169.41(17)	10.59	124.9(3)	--	4.946(7)
**oDIB-5**	1:1	2XB	1.325(3)	2.102(3)	0.016	2.783(2)	173.23(9)	6.77	109.38(16)	3.456	4.877(4)
				2.101(3)	0.015	2.790(2)	175.09(10)	5,91	120.50(19)	--	4.885(4)
**oDIB-6**	1:1	2XB	1.324(12)	2.090(10)	0.004	2.742(6)	176.6(4)	3.4	116.7(7)	3.530	4.829(10)
				2.098(9)	0.012	2.745(9)	178.2(4)	1.8	113.3(7)	3.488	4.842(15)
**oDIB-7**	1:1	2XB	1.325(10)	2.094(11)	0.008	2.777(9)	175.2(3)	4.8	108.9(6)	3.442	4.866(15)
				2.114(9)	0.028	2.863(7)	167.3(3)	12.6	142.4(7)	--	4.947(11)
**oDIB-8**	6:4	2XB, 1HB	1.332(9)	2.100(8)	0.014	2.868(7)	166.1(2)	13.9	129.7(4)		4.931(12)
				2.097(8)	0.011	2.806(7)	173.7(3)	6.3	122.7(4)		4.896(11)
		1XB 1HB	1.320(9)	2.095(8)	0.009	2.725(8)	175.4(3)	4.6	135.7(5)		4.816(12)
		1XB, 1HB	1.316(9)	2.102(7)	0.016	2.704(6)	174.6(2)	5.4	118.1(5)		4.801(10)
		2XB, 1HB	1.335(8)	2.102(8)	0.016	2.747(6)	174.1(3)	5.9	120.5(4)		4.843(10)
				2.106(7)	0.02	2.880(5)	167.4(3)	12.6	97.3(4)	3.325	4.957(9)

§AU = Asymmetric unit; ♦The uncomplexed **oDIB**'s C–I bond distances are 2.088(2) Å and 2.084(2) Å (CCDC code = 875313). The average of the two C–I bond distances is used to calculate $\Delta(C–I) = (C–I)_{complexed} – (C–I)_{uncomplexed}$; †$\Delta(\theta) = 180 – \theta_{complex}$ ▲E.g., 2XB, 1HB denotes that the N-oxide oxygen is involved in one halogen bond and two hydrogen bonds; *This C-I group is not participating in a I···O (PyNO) XB interaction.

Table S2 (continues). Halogen bond parameters of **oDIB**-PyNO complexes

Code	Donor:acceptor ratio in AU§	N–O Denticity	N–O (Å)	C–I (Å)	Δ(C–I)♦ (Å)	I···O (Å)	∠C–I···O (°)	Δθ (°)†	∠N–O···I (°)	N···I (Å)	C···O (Å)
**oDIB-9**	2:2	2XB	1.329(3)	2.108(3)	0.022	2.818(2)	172.75(10)	7.25	107.73(16)	3.457	4.916(3)
				2.102(3)	0.016	2.942(2)	164.37(9)	15.63	143.59(18)	--	4.999(4)
		2XB	1.322(3)	2.094(3)	0.008	2.943(2)	169.25(9)	10.75	133.90(17)	--	5.015(4)
				2.106(3)	0.02	2.817(2)	176.34(10)	3.66	124.71(16)	--	4.921(4)
**oDIB-10**	1:1	2XB	1.329(3)	2.109(3)	0.023	2.768(2)	173.93(9)	6.07	114.16(18)	3.527	4.871(4)
				2.116(3)	0.03	2.801(2)	177.37(8)	2.63	113.20(18)	--	4.916(3)
**oDIB-11**	1:1	2XB	1.334(3)	2.111(3)	0.025	2.772(2)	175.64(9)	4.36	120.85(14)	--	4.880(3)
				2.108(3)	0.022	2.845(2)	172.16(8)	7.84	102.81(13)	3.399	4.941(3)
**oDIB-12**	2:2	2XB;1HB	1.330(7)	2.102(6)	0.016	2.843(4)	174.73(18)	5.27	111.4(3)	--	4.941(7)
				2.110(6)	0.024	2.760(4)	175.3(2)	4.7	109.9(3)	3.448	4.865(7)
		2XB;1HB	1.320(7)	2.103(6)	0.017	2.851(4)	173.1(2)	6.9	119.1(4)	--	4.945(7)
				2.093(6)	0.007	2.984(5)	165.7(2)	14.3	104.4(3)	--	5.038(8)
**oDIB-12a**	6:2	2XB, 1HB	1.310(10)	2.099(8)	0.013	2.802(7)	173.2(3)	6.8	128.1(6)	--	4.893(10)
				2.121(10)	0.035	2.789(9)	172.5(3)	7.5	124.0(6)	--	4.900(13)
		2XB, 1HB	1.323(9)	2.126(9)	0.04	2.971(7)	169.3(3)	10.7	109.6(6)	--	5.074(11)
				2.106(8)	0.02	2.886(8)	167.3(3)	12.7	128.0(6)	--	4.962(12)
				Δ2.083(8)	-0.003	3.568(6)	152.3(3)	27.7	110.3(4)	--	5.498(10)
**oDIB-12b**	1:1	2XB	1.314(6)	2.096(6)	0.01	2.771(4)	173.3(2)	6.7	113.8(3)	3.513	4.859(7)
				2.096(6)	0.01	2.921(4)	166.21(19)	13.79	122.1(4)	--	4.981(8)
**oDIB-13**	2:2	1XB, 2HB	1.304(6)	2.099(5)	0.013	2.808(4)	172.21(15)	7.79	111.4(3)	3.501	4.895(7)
		3XB	1.326(5)	2.098(5)	0.012	2.779(4)	169.85(16)	10.15	141.5(3)	--	4.859(7)
				2.105(5)	0.019	3.056(4)	163.83(17)	16.17	91.3(3)	3.358	5.111(6)
				2.096(5)	0.01	3.252(4)	148.42(17)	31.58	113.8(3)	--	5.157(5)

§AU = Asymmetric unit; ♦The uncomplexed **oDIB**'s C–I bond distances are 2.088(2) Å and 2.084(2) Å (CCDC code = 875313). The average of the two C–I bond distances is used to calculate $\Delta(C–I) = (C–I)_{complexed} – (C–I)_{uncomplexed}$; †$\Delta(\theta) = 180 – \theta_{complex}$ ▲E.g., 2XB, 1HB denotes that the N-oxide oxygen is involved in one halogen bond and two hydrogen bonds; *This C-I group is not participating in a I···O (PyNO) XB interaction; ΔNote that this I···O interaction is just above the vdW of I+O = 3.50 Å and if this is regarded as XB, then the corresponding N-oxide oxygen is exhibiting a tridentate XB mode.

Table S2 (continues). Halogen bond parameters of **oDIB**-PyNO complexes

Code	Donor:acceptor ratio in AU[§]	N–O Denticity	N–O (Å)	C–I (Å)	Δ(C–I)[♦] (Å)	I···O (Å)	∠C–I···O/S (°)	Δθ (°)[†]	∠N–O···I (°)	N···I (Å)	C···O (Å)
**oDIB-14**	2:2	2XB	1.334(6)	2.112(5)	0.026	2.743(3)	176.69(15)	3.31	109.9(2)	3.436	4.853(6)
				2.108(5)	0.022	2.753(4)	173.36(15)	6.64	116.1(3)	--	4.853(6)
		2XB	1.332(6)	2.109(5)	0.023	2.790(4)	173.75(15)	6.25	103.5(2)	3.361	4.892(6)
				2.096(5)	0.01	2.745(4)	172.41(15)	7.59	120.3(3)	--	4.830(6)
**oDIB-15**	1:1	2XB	1.318(3)	2.106(3)	0.02	2.735(2)	176.31(9)	3.69	109.67(12)	3.413	4.839(3)
				2.105(2)	0.019	2.852(2)	173.47(7)	6.53	104.45(13)	3.423	4.949(3)
**oDIB-15a**	1.05:1	2XB	1.329(5)	2.090(5)	0.004	3.005(4)	158.56(17)	21.44	113.7(3)		5.010(7)
				2.106(5)	0.02	2.761(3)	176.79(17)	3.21	109.9(3)	3.448	4.865(7)
				2.080(5)*	-0.006	I···O passive	--	--	--	--	--
**oDIB-17**	2:1	2XB	1.314(5)	2.098(4)	0.012	2.952(3)	172.89(15)	7.11	124.4(3)	--	5.041(5)
				2.094(5)*	0.008	I···O passive	--	--	--	--	--
				2.091(5)	0.005	2.742(4)	178.70(14)	1.3	103.7(3)	3.311	4.833(6)
				2.085(5)*	-0.001	I···O passive	--	--	--	--	--
**oDIB-18a**	1:0.5[Δ]	2XB	1.320(3)	2.098(3)	0.012	2.795(2)	178.79(8)	1.21	113.95(19)	--	4.893(4)
				2.094(3)	0.008	2.911(2)	168.08(9)	11.92	119.43(14)	--	4.978(3)
**oDIB-18**	2:2[†]	Protonated N-O group. S-atom is bidentate XB acceptor.	1.368(4)	2.101(3)	0.015	I···S = 3.3914(9)	170.96(11)	9.04	--	--	5.477(3)
				2.113(4)	0.027	I···S = 3.3914(9)	175.78(8)	4.22	--	--	5.361(4)
		Protonated N-O group. S-atom is monodentate XB acceptor.	1.374(4)	2.117(3)	0.031	I···S = 3.1628(10)	171.17(11)	8.83	--	--	5.265(3)
**oDIB-19**	1:2	1XB; 1HB[▲]	1.306(6)	2.096(5)	0,01	2.850(4)	173.79(17)	6.21	111.7(3)	--	4.939(6)
				2.080(5)*	-0,006	I···O passive	--	--	--	--	--
		1HB	1.266(10)	--	--	--	--	--	--	--	--

[§]AU = Asymmetric unit; [♦]The uncomplexed **oDIB**'s C–I bond distances are 2.088(2) Å and 2.084(2) Å (CCDC code = 875313). The average of the two C–I bond distances is used to calculate $\Delta(C–I) = (C–I)_{complexed} - (C–I)_{uncomplexed}$; [†]$\Delta(\theta) = 180 - \theta_{complex}$ [▲]E.g., 1XB, 1HB denotes that the N-oxide oxygen is involved in one halogen bond and one hydrogen bond; *This C-I group is not participating in a I···O (PyNO) XB interaction; [Δ]Note that 2-mercaptopyridine is a N,N-dioxide disulfide form with only half of the molecule is located in the asymmetric unit; [†] In this complex, the 2-Mercaptopyridine has a protonated N-oxide oxygen, thus *ortho*-sulfur is forming I···S interactions.

Table S2 (continues). Halogen bond parameters of **oDIB**-PyNO complexes

Code	Donor:acceptor ratio in AU§	N–O Denticity	N–O (Å)	C–I (Å)	Δ(C–I)♦ (Å)	I···O (Å)	∠C–I···O (°)	Δθ (°)†	∠N–O···I (°)	N···I (Å)	C···O (Å)
**oDIB-20**	1:1	2XB	1.330(3)	2.106(3)	0.02	2.709(2)	175.73(11)	4.27	112.96(19)	3.452	4.812(4)
				2.099(3)	0.013	2.814(3)	171.52(10)	8.5	110.14(18)	3.502	4.899(4)
**oDIB-21**	1:1	2XB	1.333(3)	2.102(3)	0.016	2.754(2)	174.74(8)	5.26	121.98(16)	--	4.851(3)
				2.101(3)	0.015	2.761(2)	174.37(8)	5.63	117.66(15)	--	4.857(3)
**oDIB-23**	1:1	2XB	1.324(4)	2.099(5)	0.013	2.778(3)	172.99(17)	7.01	115.9(3)	--	4.867(8)
				2.106(5)	0.02	2.828(3)	178.35(14)	1.65	110.9(2)	3.524	4.933(5)
**oDIB-24**	1:1	2XB	1.306(4)	2.095(4)	0.009	2.794(2)	175.57(12)	4.43	116.6(2)	--	4.885(4)
				2.107(3)	0.021	2.814(3)	175.36(12)	4.64	114.0(2)	--	4.917(6)
**oDIB-25**	1:1	2XB	1.312(7)	2.067(7)	-0.019	2.814(6)	167.0(2)	13	127.3(4)	--	4.879(9)
				2.091(7)	0.005	2.802(6)	174.9(2)	5.1	119.2(4)	--	4.889(9)
**oDIB-26**	2:2	2XB	1.309(5)	2.102(4)	0.016	2.852(3)	167.52(12)	12.48	124.3(3)	--	4.925(5)
				2.095(4)	0.009	2.906(2)	174.25(14)	5.75	115.2(2)	--	4.996(4)
		1XB	1.308(5)	2.107(4)	0.021	2.721(2)	173.55(14)	6.45	124.9(2)	--	4.821(4)
**oDIB-27**	1:1	2XB	1.313(4)	2.100(4)	0.014	2.783(3)	173.50(12)	6.5	116.7(2)	--	4.876(4)
				2.107(4)	0.021	2.818(3)	175.04(12)	4.96	111.1(2)	3.512	4.921(5)
**oDIB-28**	1:3	1XB, 1HB▲	1.305(6)	2.097(7)	0.011	2.817(4)	177.7(2)	2.3	126.8(3)	--	4.912(7)
		2XB	1.313(6)	2.105(6)	0.019	2.856(5)	177.9(2)	2.1	119.3(3)	--	4.959(8)
				--	--	2.966(4)	172.9(2)	7.1	115.3(3)	--	4.844(7)
		2HB	1.295(6)	--	--	--	--	--	--	--	--
**oDIB-31**	1:1	2XB	1.317(6)	2.106(5)	0.02	2.779(4)	175.36(16)	4.64	119.8(3)	--	4.881(7)
				2.099(6)	0.013	2.742(4)	175.26(16)	4.74	123.3(3)	--	4.837(7)
**oDIB-32**	1:1	2XB	1.338(10)	2.099(8)	0.013	2.784(7)	172.7(3)	7.3	115.5(5)	--	4.874(11)
				2.100(8)	0.014	2.725(6)	176.7(3)	3.3	111.8(5)	3.454	4.823(10)

§AU = Asymmetric unit; ♦The uncomplexed **oDIB**'s C–I bond distances are 2.088(2) Å and 2.084(2) Å (CCDC code = 875313). The average of the two C–I bond distances is used to calculate $\Delta(\text{C–I}) = (\text{C–I})_{complexed} - (\text{C–I})_{uncomplexed}$; †$\Delta(\theta) = 180 - \theta_{complex}$ ▲E.g., 1XB, 1HB denotes that the N-oxide oxygen is involved in one halogen bond and one hydrogen bond.

Table S3. Halogen bond parameters of **mDIB**-PyNO complexes

**Code**	Donor:acceptor ratio in AU§	N–O Denticity	N–O (Å)	C–I (Å)	Δ(C–I)♦ (Å)	I···O (Å)	∠C–I···O (°)	Δθ (°)†	∠N–O···I (°)	N···I (Å)	C···O (Å)
**mDIB-1**	0.5:1	1XB; 2HB▲	1.328(5)	2.103(4)	0.027	2.740(3)	176.51(12)	3.49	111.7(2)	3.459	4.840(5)
**mDIB-2**	1:2	1XB; 2HB	1.315(8)	2.094(7)	0.018	2.739(4)	175.7(2)	4.3	111.0(4)	3.437	4.829(7)
		1XB; 2HB	1.318(8)	2.101(6)	0.025	2.728(4)	175.9(3)	4.1	107.7(4)	3.371	4.826(7)
**mDIB-3**	2:3	1XB, 2HB	1.324(4)	2.106(4)	0.03	2.740(3)	172.12(11)	7.88	110.6(2)	3.348	4.835(5)
		2XB, 1HB	1.320(4)	2.097(4)	0.021	2.763(3)	173,81(12)	6.19	134.8(2)	--	4.853(4)
				2.101(4)	0.025	2.726(3)	177.45(10)	2.55	118.3(2)	--	4.826(5)
		1XB, 1HB	1.315(4)	2.094(4)	0.018	2.765(3)	172.52(13)	7.48	133.0(2)	--	4.849(5)
**mDIB-4**	2:2	2XB; 2HB	1.323(6)	2.098(5)	0.022	2.714(5)	176.40(17)	3.6	106.8(3)	3.345	4.810(7)
				2.098(5)	0.022	2.771(4)	175.86(15)	4.14	119.7(3)	--	4.867(7)
		2XB; 2HB	1.339(7)	2.116(5)	0.04	2.715(5)	177.05(18)	2.95	106.6(3)	3.353	4.830(7)
				2.101(5)	0.025	2.728(4)	176.14(18)	3.86	122.3(3)	--	4.826(7)
**mDIB-5**	1:1	2XB	1.329(5)	2.093(5)	0,017	2,749(3)	177.25(13)	2.75	117.4(3)	--	4.839(6)
				2.104(4)	0,028	2.776(3)	177.41(13)	2.59	118.8(2)	--	4.879(5)
**mDIB-6**	0.5:1	1XB; 1HB	1.320(3)	2.101(2)	0.025	2.741(2)	172.73(8)	7.27	114.84(14)	3.507	4.833(3)
**mDIB-7**	0.5:1	1XB, 1HB	1.329(4)	2.103(3)	0,027	2.788(3)	175.95(10)	4.05	118.51(17)	--	4.888(4)
**mDIB-8**	1:2	1XB; 2HB	1.325(8)	2.105(7)	0.029	2.652(6)	176.4(3)	3.6	111.9(4)	3.379	4.755(10)
		1XB; 3HB	1.317(8)	2.097(6)	0.021	2.687(6)	174.8(2)	5.2	124.9(4)	--	4.780(8)
**mDIB-9**	2:1	2XB	1.339(6)	2.106(6)	0.03	2.736(4)	174.34(18)	5.66	125.9(3)	--	4.836(6)
				2.085(5)	0.009	I···O passive	--			--	
				2.103(5)	0.027	2.714(4)	177.09(16)	2.91	124.5(3)	--	4.815(7)
				2.088(5)	0.012	I···O passive				--	
**mDIB-11**	2:2	2XB; 1HB	1.325(6)	2.108(5)	0.032	2.745(3)	177.71(18)	2.29	107.1(3)	3.381	4.853(6)
				2.106(5)	0.03	2.810(3)	177.23(17)	2.77	125.0(3)	--	4.915(6)
		2XB; 1HB	1.321(6)	2.093(5)	0.017	2.779(4)	177.11(15)	2.89	125.7(3)	--	4.870(6)
				2.106(5)	0.03	2.733(4)	177.25(13)	2.75	108.8(3)	3.397	4.838(6)

§AU = Asymmetric unit; ♦The uncomplexed **mDIB**'s C–I bond distance is 2.076(9), 2.070(8), 2.073(9), 2.073(9), 2.087(8), 2.081(9)Å (our structure, CCDC code =2337518). Δ(C–I) = (C–I)$_{complexed}$ – (C–I)$_{uncomplexed}$. Note that the average (2.076(2) Å) of six C-I bond lengths is used for (C–I)$_{uncomplexed}$; †Δ(θ) = 180 – θ$_{complex}$ ▲1XB, 2HB denotes that the N-oxide oxygen is involved in one halogen bond and two hydrogen bonds; *This C-I group is not participating in a I···O (PyNO) XB interaction.

Table S3 (Continues). Halogen bond parameters of **mDIB**-PyNO complexes

Code	Donor:acceptor ratio in AU[§]	N–O Denticity	N–O (Å)	C–I (Å)	Δ(C–I)[♦] (Å)	I···O (Å)	∠C–I···O (°)	Δθ (°)[†]	∠N–O···I (°)	N···I (Å)	C···O (Å)
**mDIB-15**	1:1	1XB; 2HB	1.310(5)	2.102(4)	0.026	2.818(3)	167.84(13)	12.16	128.4(2)	--	4.893(4)
				2.073(4)*	-0.003	I···O passive	--	--	--	--	--
**mDIB-19**	2:1	1XB; 1HB	1.297(5)	2.090(4)	0.014	2.773(3)	174.64(13)	5.36	144.3(3)	--	4.858(5)
				2.096(4)*	0.02	I···O passive	--	--	--	--	--
				2.090(4)*	0.014	I···O passive	--	--	--	--	--
				2.087(4)*	0.011	I···O passive	--	--	--	--	--
**mDIB-21**	1:1	2XB; 2HB	1.329(6)	2.100(5)	0.024	2.767(4)	175.85(16)	4.15	115.9(3)	--	4.863(6)
				2.096(5)	0.02	2.881(4)	172.80(16)	7.2	120.3(3)	--	4.968(6)
**mDIB-22**	1:1	2XB; 1HB	1.327(3)	2.092(3)	0.016	2.792(2)	172.11(9)	7.89	103.09(12)	3.352	4.872(3)
				2.093(3)	0.017	2.918(2)	171.37(7)	8.63	117.71(15)	--	4.997(4)
**mDIB-29**	2:2	3XB	1.302(7)	2.093(6)	0.017	2.937(5)[₸]	174.27(19)	5.73	121.0(4)	--	5.024(9)
				2.087(6)	0.011	3.119(4)	154.1(2)	25.9	109.8(4)	--	5.078(8)
				2.105(6)	0.029	2.815(4)	171.4(3)	8.6	120.8(3)	--	4.906(8)
		2XB, 1HB	1.319(7)	2.094(7)	0.018	2.957(5)[₸]	170.3(2)	9.7	120.9(3)	--	5.034(9)
				2.091(7)	0.015	2.749(5)	170.76(18)	9.24	116.9(4)	--	4.825(9)
**mDIB-31**	1:1	1XB, 2HB	1.347(16)	2.092(14)	0.016	2.676(10)	177.0(4)	3.0	105.8(7)	3.308	4.767(16)
				2.091(14)*	0.015	I···O passive				--	
**mDIB-32**	1:2	1XB, 1HB	1.317(4)	2.100(4)	0.024	2.699(3)	176.85(10)	3.15	112.6(2)	3.427	4.797(4)
		1XB, 1HB	1.317(4)	2.103(4)	0.027	2.691(3)	177.91(12)	2.09	127.9(2)	--	4.792(4)

[§]AU = Asymmetric unit; [♦]The uncomplexed **mDIB**'s C–I bond distance is 2.076(9), 2.070(8), 2.073(9), 2.073(9), 2.087(8), 2.081(9)Å (our structure, CCDC code = 2337518). $\Delta(C–I) = (C–I)_{complexed} - (C–I)_{uncomplexed}$. Note that the average (2.076(2) Å) of six C-I bond lengths is used for $(C–I)_{uncomplexed}$; [†]$\Delta(\theta) = 180 - \theta_{complex}$ [▲]1XB, 2HB denotes that the N-oxide oxygen is involved in one halogen bond and two hydrogen bonds; *This C-I group is not participating in a I···O (PyNO) XB interaction; [₸] The PyNO's C-I group id XB donor.

Table S4. Halogen bond parameters of **pDIB**-PyNO complexes

Code	Donor:acceptor ratio in AU[$]	N–O Denticity	N–O (Å)	C–I (Å)	Δ(C–I)[♦] (Å)	I···O (Å)	∠C–I···O (°)	Δθ (°)[†]	∠N–O···I (°)	N···I (Å)	C···O (Å)
**pDIB-1**	2:2	1XB, 2XB[▲]	1.325(5)	2.095(5)	0.02	2.706(3)	176.68(16)	3.32	112.3(3)	3.435	4.798(6)
		1XB, 2XB	1.335(5)	2.097(5)	0.022	2.692(4)	177.87(15)	2.13	108.6(3)	--	4.789(6)
**pDIB-2**	1:1	2XB; 1HB	1.320(4)	2.096(4)	0.021	2.764(3)	174.88(13)	5.12	106.58(19)	3.364	4.855(5)
				2.098(4)	0.023	2.732(3)	178.22(13)	1.78	118.42(19)	--	4.829(5)
**pDIB-3**	1:1	2XB	1.328(6)	2.089(5)	0.014	2.858(4)	176.76(18)	3.24	123.4(3)	--	4.945(7)
				2.093(5)	0.018	2.823(4)	175.13(18)	4.87	106.3(3)	3.441	4.912(7)
**pDIB-4**	1:1	2XB; 1HB	1.328(5)	2.095(4)	0.02	2.746(3)	176.74(13)	3.26	115.9(2)	3.534	4.839(5)
				2.107(4)	0.032	2.725(3)	175.82(13)	4.18	123.2.(2)	--	4.829(5)
**pDIB-5**	2:2	2XB; 1HB	1.325(4)	2.099(4)	0.024	2.718(3)	174.19(13)	5.81	116.4(2)	3.513	4.810(5)
				2.101(4)	0.026	2.698(3)	175.13(13)	4.87	127.6(2)	--	4.794(5)
		2XB; 1HB	1.337(5)	2.103(4)	0.028	2.697(3)	174.52(13)	5.48	126.1(2)	--	4.795(5)
				2.096(4)	0,021	2.727(3)	172.18(13)	7.82	118.2(2)	--	4.811(5)
**pDIB-6**	2x0.5:1	2XB	1.324(3)	2.096(3)	0.021	2.762(2)	169.1(1)	10.9	125.59(18)	--	4.835(4)
				2.095(3)	0.02	2.738(2)	174.17(10)	5.83	112.70(17)	3.470	4.826(4)
**pDIB-7**	2:1	2XB	1.335(3)	2.101(3)	0.026	2.699(2)	179.14(10)	0.86	114.37(16)	3.470	4.799(4)
				2.101(3)	0.026	2.751(2)	171.73(11)	8.27	110.09(16)	3.446	4.839(4)
				2.075(3)*	0	I···O passive	--	--	--	--	--
				2.076(3)*	0.001	I···O passive	--	--	--	--	--
**pDIB-8**	2:2 (2x0.5 XB donors + 1 full XB donor)	2XB; 1HB	1.346(6)	2.093(6)	0.018	2.728(5)	166.9(2)	13.1	120.3(4)	--	4.791(10)
				2.105(6)	0.03	2.729(4)	176.1(2)	3.9	113.1(3)	3.485	4.831(6)
		1XB; 2HB	1.314(6)	2.109(5)	0.034	2.816(4)	176.6(2)	3.4	112.4(4)	3.532	4.923(7)
				2.069(6)*	-0.006	I···O passive	--		--	--	--
**pDIB-9**	2x0.5:1	2XB	1.337(3)	2.101(3)	0.026	2.707(2)	179.16(8)	0.84	116.93(15)	3.521	4.808(3)
				2.103(3)	0.028	2.745(2)	178.60(8)	1.4	127.70(15)	--	4.847(3)
**pDIB-10**	2x0.5:1	2XB	1.317(6)	2.093(3)	0.018	2.815(2)	172.08(8)	7.92	109.32(16)	3.489	4.897(3)
				2.086(3)	0.011	2.793(2)	173.42(8)	6.58	119.64(15)	--	4.871(3)
**pDIB-11**	1:1	2XB;2HB	1.333(3)	2.092(3)	0.017	2.739(2)	175.56(7)	4.42	129.95(14)	--	4.827(3)
				2.094(3)	0.019	2.775(2)	171.63(9)	8.37	102.76(12)	3.334	4.856(3)

[$]AU = Asymmetric unit; [♦]The uncomplexed **pDIB**'s C–I bond distance is 2.075(2) Å (CCDC code = ZZZAVM01). This is used to calculate $\Delta(C–I) = (C–I)_{complexed} – (C–I)_{uncomplexed}$; [†]$\Delta(\theta) = 180 – \theta_{complex}$ [▲]E.g., 1XB, 2HB denotes that the N-oxide oxygen is involved in one halogen bond and two hydrogen bonds; *This C-I group is not participating in a I···O (PyNO) XB interaction.

Table S4 (continues). Halogen bond parameters of **pDIB**-PyNO complexes

Code	Donor:acceptor ratio in AU§	N–O Denticity	N–O (Å)	C–I (Å)	Δ(C–I)♦ (Å)	I···O (Å)	∠C–I···O/S (°)	Δθ (°)†	∠N–O···I (°)	N···I (Å)	C···O (Å)
**pDIB-12**	2x0.5:2	1XB, 2HB	1.343(4)	2.094(4)	0.019	2.777(3)	175.89(12)	4.11	108.9(2)	3.455	4.869(5)
		water oxygen is a XB acceptor		2.092(4)	0.017	2.839(3)	177.14(13)	3.86	--	--	4.929(5)
**pDIB-13**	0.5:1	1XB, 2HB	1.322(4)	2.095(3)	0.02	2.799(3)	169.35(10)	10.65	132.2(2)	--	4.873(5)
**pDIB-14**	2:2	2XB; 1HB	1.349(17)	2.096(17)	0.021	2.800(12)	172.8(5)	7.2	109.7(8)	3.493	4.88(2)
				2.066(18)	-0.009	2.853(12)	172.1(5)	7.5	115.9(8)	--	4.91(2)
		2XB; 1HB	1.328(17)	2.087(16)	0.012	2.990(12)	168.0(5)	12	121.6(9)	--	5.05(2)
				2.088(18)	0.013	2.744(12)	172.5(5)	7.5	105.6(8)	3.355	4.89(2)
**pDIB-15**	0.5:1	1XB; 2HB	1.312(5)	2.094(5)	0.019	2.770(3)	172.15(17)	7.85	129.3(3)	--	4.853(6)
**pDIB-16**	0.5:1	1XB; 2HB	1.329(2)	2.099(2)	0.024	2.716(2)	175.84(7)	4.16	117.09(14)	3.526	4.812(3)
**pDIB-17**	2x0.5:1	2XB	1.311(10)	2.091(6)	0.016	2.770(2)	176.7(2)	3.3	111.07(11)	3.465	4.859(7)
				2.069(12)*	-0.006	I···O passive	--	--	--	--	--
				2.067(12)*	-0.008	I···O passive	--	--	--	--	--
**pDIB-18**	0.5:1	Protonated N–O group. *Ortho*-S-atom is a monodentate XB acceptor.	1.381(3)	2.092(3)	0.017	3.2076(2)	177.49(7)	2.51	--	--	5.367
**pDIB-18a**	0.5:0.5	1XB; 1HB	1.319(2)	2.091(2)	0.016	2.724(2)	178.92(7)	1.08	109.22(11)	3.395	4.814(2)
**pDIB-19**	2x0.5:1	1XB; 1HB	1.302(4)	2.096(4)	0.021	2.803(3)	169.07(13)	10.93	145.3(2)	--	4.877(5)
		-NO_2 group oxygen is a XB acceptor		2.083(4)	0.008	2.986(3)	170.95(12)	9.05	111.6(2)	--	5.054(5)
**pDIB-21**	2:2	2XB; 2HB	1.328(2)	2.080(2)	0.005	2.968(2)	161.19(6)	18.81	86.91(11)	3.186	4.983(3)
				2.093(2)	0.018	2.739(2)	170.58(8)	9.42	115.18(13)	3.516	4.817(3)
		2XB; 2HB	1.328(2)	2.079(2)	0.004	3.052(2)	159.85(8)	20.15	89.16(11)	3.310	5.055(3)
				2.089(2)	0.014	2.772(2)	174.44(9)	5.56	116.28(13)	--	4.856(3)
**pDIB-23**	1:1	2XB; 1HB	1.315(8)	2.090(7)	0.015	2.726(5)	174.1(3)	5.9	118.3(4)	--	4.812(9)
				2.088(7)	0.013	2.750(5)	174.0(3)	6	109.6(4)	3.425	4.833(9)

§AU = Asymmetric unit; ♦The uncomplexed **pDIB**'s C–I bond distance is 2.075(2) Å (CCDC code = ZZZAVM01). This is used to calculate Δ(C–I) = $(C–I)_{complexed}$ – $(C–I)_{uncomplexed}$; †$\Delta(\theta) = 180 - \theta_{complex}$ ▲E.g., 1XB, 1HB denotes that the N-oxide oxygen is involved in one halogen bond and one hydrogen bond; *This C-I group is not participating in a I···O (PyNO) XB interaction.

Table S4 (continues). Halogen bond parameters of **pDIB**-PyNO complexes

Code	Donor:acceptor ratio in AU[§]	N–O Denticity	N–O (Å)	C–I (Å)	Δ(C–I)[♦] (Å)	I···O (Å)	∠C–I···O (°)	Δθ (°)[┼]	∠N–O···I (°)	N···I (Å)	C···O (Å)
**pDIB-24**	0.5:0.5	1XB; 1XB	1.306(5)	2.083(4)	0.008	2.948(3)	168.37(14)	11.63	152.4(2)	--	5.006(5)
**pDIB-26**	0.5:1	1XB; 2HB	1.304(4)	2.083(3)	0.008	2.799(3)	169.67(10)	10.33	149.5(2)	--	4.862(4)
**pDIB-27**	2:2 (2x0.5 XB donors + 1 full XB donor)	2XB	1.318(7)	2.075(6)[Δ]	0	2.798(5)	174.16(18)	5.84	127.8(3)	--	4.867(8)
				2.095(6)	0.02	2.882(4)	170.63(19)	9.37	122.7(3)	--	4.961(7)
		2XB; 1HB	1.311(6)	2.084(6)[Δ]	0.009	2.802(4)	169.8(2)	10.2	113.6(3)	--	4.867(7)
				2.081(6)	0.006	2.785(4)	169.69(19)	10.31	126.2(3)	--	4.846(7)
				2.077(7)*	0.002	I···O passive	--	--	--	--	--
				2.071(7)*	-0.004	I···O passive	--	--	--	--	--
**pDIB-28**	0.5:1	1XB; 2HB	1.307(5)	2.092(4)	0.017	2.761(4)	168.26(15)	11.74	115.3(8)	--	4.827(6)
**pDIB-29**	1:1	3XB; 1HB	1.330(5)	2.087(6)	0.012	2.787(4)	176.63(15)	3.37	112.4(3)	3.515	4.871(7)
				2.077(6)	0.002	3.239(4)	152.40(16)	27.6	91.5(3)	3.534	5.170(7)
				2.107(5)[Δ]	0.032	2.974(3)	167.65(16)	12.35	120.4(2)	--	5.052(6)
**pDIB-31**	2:2	1XB; 2HB	1.304(3)	2.092(3)	0.017	2.721(2)	173.71(9)	6.29	127.63(15)	--	4.806(4)
		1XB; 1HB	1.308(3)	2.092(3)	0.017	2.760(2)	171.28(10)	8.72	133.5(2)	--	4.838(4)

[§]AU = Asymmetric unit; [♦]The uncomplexed **pDIB**'s C–I bond distance is 2.075(2) Å (CCDC code = ZZZAVM01). This is used to calculate Δ(C–I) = $(C–I)_{complexed}$ – $(C–I)_{uncomplexed}$; [┼]Δ(θ) = 180 – $θ_{complex}$ [▲]E.g., 1XB, 1HB denotes that the N-oxide oxygen is involved in one halogen bond and one hydrogen bond; *This C–I group is not participating in a I···O (PyNO) XB interaction; [Δ]The iodine or bromine of C–I/Br group of the PyNO is XB donor. 8

Table S5. Halogen bond parameters of **trIB**-PyNO complexes

Code	Donor:acceptor ratio in AU[$]	N–O Denticity	N–O (Å)	C–I (Å)	Δ(C–I)[♦] (Å)	I···O (Å)	∠C–I···O (°)	Δθ (°)†	∠N–O···I (°)	N···I (Å)	C···O (Å)
**trIB-1**	1:1	2XB; 1HB[▲]	1.330(3)	2.105(3)	0.015	2.750(2)	176.88(8)	3.12	105.24(14)	3.354	4.853(3)
				2.096(3)	0.006	2.819(2)	179.64(8)	0.36	112.41(15)	--	4.914(3)
				2.081(3)*	-0.009	I···O passive	--	--	--	--	--
**trIB-2**	1:1	2XB; 1HB	1.330(3)	2.103(3)	0.013	2.770(2)	177.58(8)	2.42	107.14(13)	3.408	4.871(3)
				2.099(3)	0.009	2.838(2)	175.44(9)	4.56	115.53(12)	--	4.934(3)
				2.078(3)*	-0.014	I···O passive	--	--	--	--	--
**trIB-3**	1:1	2XB; 2HB	1.332(4)	2.107(4)	0.017	2.786(3)	175.55(11)	4.45	102.9(2)	3.344	4.889(5)
				2.085(4)	-0.005	3.032(3)	166.12(12)	13.88	97.03(19)	3.457	5.080(5)
				2.081(4)*	-0.009	I···O passive	--	--	--	--	--
**trIB-4**	1:1	2XB; 1HB	1.309(19)	2.091(17)	0.001	2.766(12)	179.1(5)	0.9	119.3(9)	--	4.86(2)
				2.110(17)	0.02	2.795(13)	168.3(5)	11.7	101.6(9)	3.316	4.88(2)
				2.072(17)*	-0.018	I···O passive	--	--	--	--	
**trIB-5**	1:1	2XB	1.327(4)	2.107(3)	0.017	2.763(3)	175.98(11)	4.02	114.93(19)	3.533	4.867(5)
				2.096(3)	0.006	2.803(2)	173.15(13)	6.85	116.43(17)	--	4.890(4)
				2.074(4)	-0.016	I···O passive				--	
**trIB-6**	1:1	2XB	1.328(4)	2.099(4)	0.009	2.812(3)	174.02(11)	5.98	108.4(2)	3.468	4.905(4)
				2.101(4)	0.011	2.850(3)	169.86(10)	10.14	121.0(2)	--	4.932(5)
				2.082(4)*	-0.008	I···O passive	--	--	--	--	--
**trIB-7**	1:1	2XB	1.329(4)	2.098(4)	0.008	2.826(3)	174.77(12)	5.23	100.45(19)	3.334	4.920(5)
				2.089(4)	-0.001	2.882(2)	170.12(12)	9.88	128.3(2)	--	4.53(4)
				2.084(4)*	-0.006	I···O passive	--	--	--	--	--
**trIB-8**	1:1	2XB	1.337(4)	2.101(3)	0.011	2.787(3)	171.64(11)	8.36	106.17(18)	3.410	4.875(5)
				2.090(3)	0	2.999(2)	167.71(10)	12.29	111.4(2)	--	5.060(4)
				2.088(3)*	-0.002	I···O passive	--	--	--	--	--

[$]AU = Asymmetric unit; [♦]The uncomplexed **trIB**'s C–I bond distances are 2.091(4) Å, 2.089(4) Å, and 2.090(5) Å (CCDC code = UCEPEY01) and their average 2.090(4) Å is used to calculate Δ(C–I) = $(C–I)_{complexed}$ – $(C–I)_{uncomplexed}$; †Δ(θ) = 180 – $\theta_{complex}$ [▲]E.g., 2XB, 1HB denotes that the N-oxide oxygen is involved in two halogen bonds and one hydrogen bond; *This C–I group is not participating in a I···O (PyNO) XB interaction.

Table S5 (Continues). Halogen bond parameters of **trIB**-PyNO complexes

Code	Donor:acceptor ratio in AU[§]	N–O Denticity	N–O (Å)	C–I (Å)	Δ(C–I)[♦] (Å)	I···O (Å)	∠C–I···O (°)	Δθ (°)[┼]	∠N–O···I (°)	N···I (Å)	C···O (Å)
**trIB-9**	1:1	2XB	1.319(15)	2.089(16)	-0.004	2.918(9)	165.4(5)	14.6	123.1(7)	--	4.966(18)
				2.075(12)*	-0.015	I···O passive	--	--	--	--	--
				2.092(16)*	0.002	I···O passive	--	--	--	--	--
				2.096(14)	0.006	2.757(9)	176.5(4)	3.5	103.1(7)	3.315	4.851(16)
				2.079(15)*	-0.011	I···O passive	--	--	--	--	--
				2.080(16)*	-0.01	I···O passive	--	--	--	--	--
**trIB-11**	1:1	2XB	1.328(16)	2.092(12)	0.002	2.804(9)	170.1(4)	9.9	128.9(6)	--	4.879(15)
				2.112(13)	0.022	2.832(9)	174.5(3)	5.5	122.9(6)	--	4.939(16)
				2.074(14)*	-0.016	I···O passive	--	--	--	--	--
**trIB-12**	0.5:1	1XB; 2HB	1.326(4)	2.097(3)	0.007	2.736(2)	178.18(10)	1.82	115.37(18)	3.514	4.833(4)
				2.085(5)	-0.005	I···O passive	--	--	--	--	--
**trIB-13**	1:1	2XB; 1HB[▲]	1.319(3)	2.097(3)	0.007	2.696(2)	179.33(9)	0.67	108.26(17)	3.353	4.793(4)
			-OCH_3 group oxygen is XB acceptor	2.084(3)	-0.006	3.083(2)	165.36(10)	14.64	--	--	5.126(4)
				2.082(3)*	-0.008	I···O passive	--	--	--	--	--
**trIB-15**	1:1	2XB; 1HB	1.329(4)	2.099(3)	0.009	2.787(2)	177.19(11)	2.81	105.10(16)	3.386	4.885(4)
				2.092(3)	0.002	2.843(2)	177.85(10)	2.15	111.15(14)	--	4.934(3)
				2.084(3)*	-0.006	passive	--	--	--	--	--
**trIB-16**	1:1	2XB; 2HB		2.100(3)	0.01	2.798(3)	177.95(12)	2.05	107.09(18)	3.433	4.898(5)
				2.100(4)	0.01	2.945(3)	171.78(12)	8.22	110.53(19)	--	5.033(5)
				2.085(4)	-0.005	2.904(3)	166.12(13)	13.88	--	--	4.954(5)

[§]AU = Asymmetric unit; [♦]The uncomplexed **trIB**'s C–I bond distances are 2.091(4) Å, 2.089(4) Å, and 2.090(5) Å (CCDC code = UCEPEY01) and their average 2.090(4) Å is used to calculate $\Delta(C–I) = (C–I)_{complexed} – (C–I)_{uncomplexed}$; [┼]$\Delta(\theta) = 180 – \theta_{complex}$ [▲]E.g., 2XB, 1HB denotes that the N-oxide oxygen is involved in two halogen bonds and one hydrogen bond; *This C–I group is not participating in a I···O (PyNO) XB interaction;

Table S5 (Continues). Halogen bond parameters of **trIB**-PyNO complexes

**Code**	Donor:acceptor ratio in AU§	N–O Denticity	N–O (Å)	C–I (Å)	Δ(C–I)♦ (Å)	I···O/S (Å)	∠C–I···O/S (°)	Δθ (°)†	∠N–O···I (°)	N···I (Å)	C···O (Å)
**trIB-17**	3:3	2XB; 1HB	1.330(4)	2.089(4)	-0.001	2.877(2)	171.11(10)	8.89	102.83(18)	3.427	4.952(4)
				2.097(3)	0.007	2.778(3)	171.69(9)	8.31	116.45(19)	--	4.862(5)
				2.080(3)*	-0.01	I···O passive			--	--	
		2XB; 1HB	1.320(4)	2.093(3)	0.003	2.766(2)	174.16(11)	5.84	114.0(2)	3.517	4.852(4)
				2.101(4)	0.011	2.723(3)	175.13(11)	4.87	114.41(19)	3.482	4.820(5)
			Acetone oxygen is XB acceptor	2.087(4)*	-0.003	3.033(4)	168.88(11)	11.12	--	--	5.096(5)
		2XB; 1HB	1.323(4)	2.098(3)	0.008	2.815(3)	177.01(9)	2.99	103.10(9)	3.371	4.911(5)
				2.094(4)	0.004	2.824(2)	171.11(11)	8.89	130.2(2)	--	4.903(4)
				2.085(3)*	-0.005	I···O passive			--	--	4.952(4)
**trIB-18**	1:1	Protonated N–O group. *Ortho*-S-atom is a bidentate XB acceptor.	1.362(9)	2.084(6)	-0.006	--	--	--	--	--	--
				2.103(6)	0.013	3.287(3)	176.53(19)	3.47	--	--	5.387(7)
				2.107(6)	0.017	3.229(2)	176.0(1)	4.0	--	--	5.334(7)
**trIB-20****	2:2	2XB; 1HB	1.354(9)	2.134(7)	0.044	2.802(6)	172.9(3)	7.1	104,9(3)	--	4.926(9)
				2.108(6)	0.018	2.724(5)	173.5(5)	6.5	108.1(4)	--	4.824(9)
		2XB; 1HB	1.36(2) 1.230(19)	2.093(6)	0.003	2.691(17)	171,3(4)	8,7	106.8(10)	--	4.782(18)
				2.104(7)	0.014	2.824(18)	167.6(4)	12.4	125.8(11)	--	4.888(19)
			-OCH_3 group oxygen is XB acceptor	2.079(6)	-0.011	3.037(7)				--	
				2.080(6)	-0.01	I···O passive				--	
**trIB-21**	1:1	2XB	1.320(10)	2.099(9)	0.009	2.721(7)	179.9(3)	0.1	112.2(5)	3.442	4.820(11)
				2.089(9)	-0.001	2.763(6)	173.0(3)	7	132.2(5)	--	4.844(11)
				2.086(9)	-0.004	I ···O passive				--	--
**trIB-22**	1:1	1XB▲	1.319(5)	2.097(4)	0.007	2.741(3)	175.50(14)	4.5	131.9(3)	--	4.835(6)
				2.086(4)*	-0.004	I···O passive	--	--	--	--	--
				2.077(4)*	-0.013	I···O passive	--	--	--	--	--

§AU = Asymmetric unit; ♦The uncomplexed **trIB**'s C–I bond distances are 2.091(4) Å, 2.089(4) Å, and 2.090(5) Å (CCDC code = UCEPEY01) and their average 2.090(4) Å is used to calculate Δ(C–I) = $(C–I)_{complexed}$ – $(C–I)_{uncomplexed}$; †Δ(θ) = 180 – $θ_{complex}$ ▲E.g., 1XB denotes that the N-oxide oxygen is involved in one halogen bond; *This C–I group is not participating in a I···O (PyNO) XB interaction; **This data is not included in the statistical analysis due to disorder.

Table S5 (Continues). Halogen bond parameters of **trIB**-PyNO complexes

Code	Donor:acceptor ratio in AU§	N–O Denticity	N–O (Å)	C–I (Å)	Δ(C–I)♦ (Å)	I···O/S (Å)	∠C–I···O/S (°)	Δθ (°)†	∠N–O···I (°)	N···I (Å)	C···O (Å)
**trIB-28**	1:1	3XB; 1HB	1.325(5)	2.101(4)	0.011	2.863(4)	170.66(14)	9.34	112.8(2)	--	4.949(6)
				2.087(4)	-0.003	2.835(3)	165.32(14)	14.68	112.3(3)	--	4.883(5)
				1.882(5)Δ		2.966(3)	165.39(15)	14.61	120.3(3)	--	4.810(6)
				2.081(4)	-0.009	I···O passive				--	
**trIB-29**	1:1	3XB; 1HB	1.320(7)	2.089(6)	-0.001	2.889(4)	164.0(2)	16.0	111.4(3)	--	4.930(9)
				2.089(6)	-0.001	2.891(5)	171.62(18)	8.38	111.4(3)	--	4.966(9)
				2.087(6)Δ	-0.003	2.947(5)	167.6(2)	12.4	121.0(4)	--	5.006(8)
				2.082(6)	-0.008	I···O passive				--	
**trIB-30**	1:1	1XB	1.320(4)	2.096(3)	0.006	2.696(2)	179.33(9)	0.67	108.26(17)	3.412	4.793(4)
			-NO_2 group oxygen is XB acceptor	2.081(3)	-0.009	3.089(3)	164.85(9)	15.15	140.0(3)	--	5.126(5)
				2.084(3)*	-0.006	I···O passive	--	--	--	--	--
**trIB-32**	1:1	2XB; 2HB	1.328(6)	2.103(5)	0.013	2.849(4)	170.65(16)	9.35	114.1(3)	--	4.936(8)
				2.088(5)	-0.002	2.932(3)	161.48(13)	18.52	110.0(3)	--	4.956(6)
				2.083(5)	-0.007	I···O passive				--	

§AU = Asymmetric unit; ♦The uncomplexed **trIB**'s C–I bond distances are 2.091(4) Å, 2.089(4) Å, and 2.090(5) Å (CCDC code = UCEPEY01) and their average 2.090(4) Å is used to calculate Δ(C–I) = $(C–I)_{complexed}$ – $(C–I)_{uncomplexed}$; †Δ(θ) = 180 – $θ_{complex}$ ▲E.g., 1XB denotes that the N-oxide oxygen is involved in one halogen bond; *This C–I group is not participating in a I···O (PyNO) XB interaction; ΔThe iodine or bromine of C–I/Br group of the PyNO is XB donor.

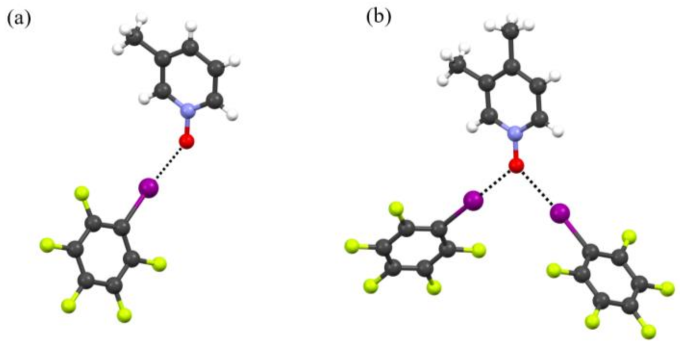

Figure S130. Examples of 1:1 and 2:1 donor-acceptor halogen-bonded complexes are shown using (a) **PfIB-1** and (b) **PfIB-8**. The halogen bond is shown by the broken black line.

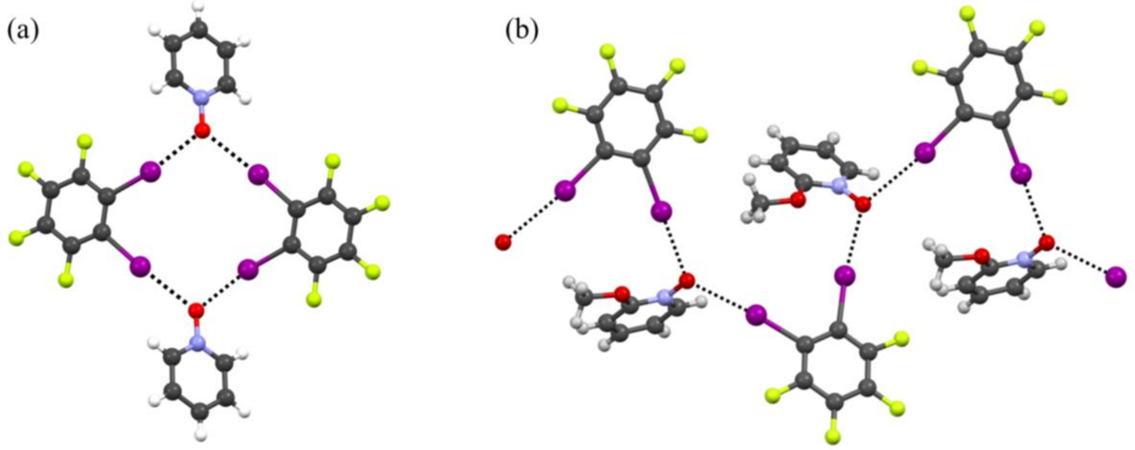

Figure S131. Examples of 2:2 donor:acceptor macrocycles and polymeric halogen-bonded complexes are shown using (a) **oDIB-1** and (b) **oDIB-12**. The halogen bond is shown by the broken black line.

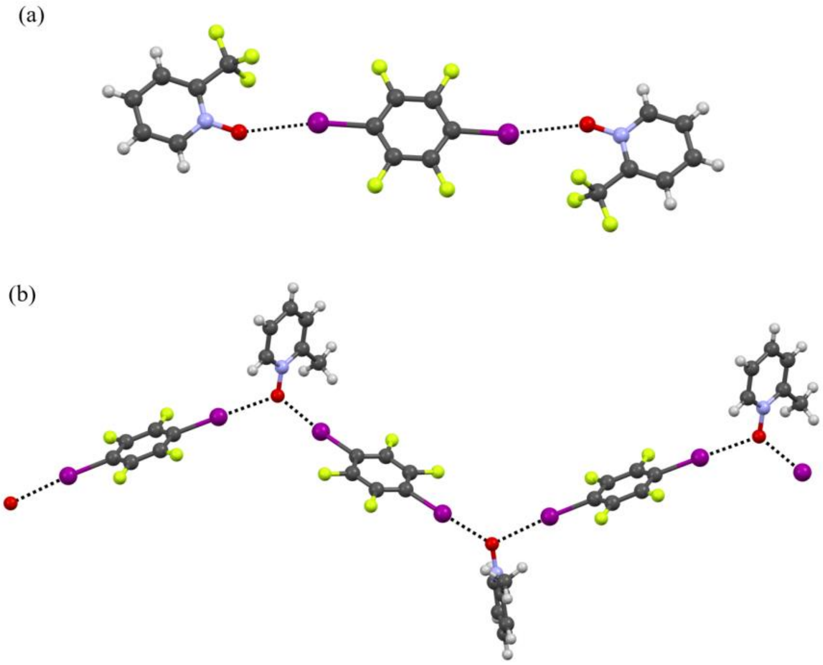

Figure S132. Example 1:2 donor:acceptor discrete and polymeric halogen-bonded complexes shown using (a) **pDIB-24** and (b) **pDIB-2**. The halogen bond is shown by the broken black line.

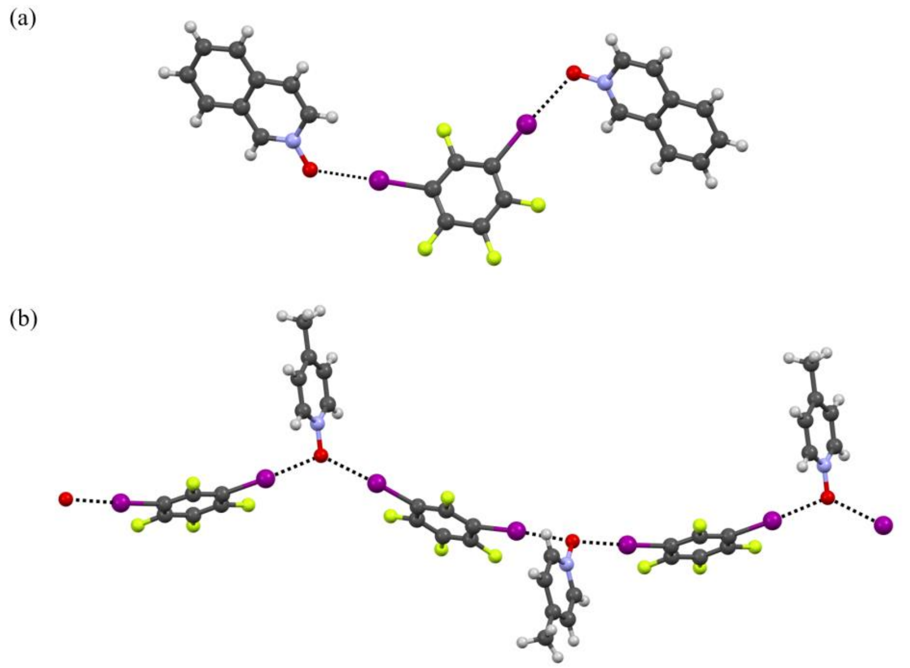

Figure S133. Example discrete and polymeric halogen-bonded complexes displayed using (a) **mDIB-32**, and **mDIB-4**. The halogen bond is shown by the broken black line.

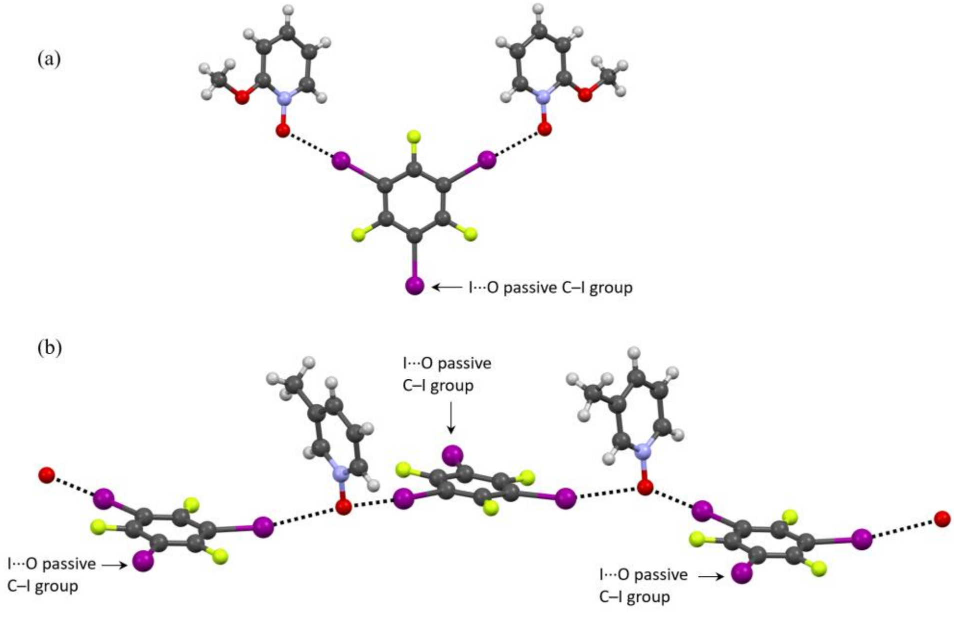

Figure S134. Example discrete and polymeric halogen-bonded complexes displayed using (a) **trIB-12**, and **trIB-3**. The halogen bond is shown by the broken black line.

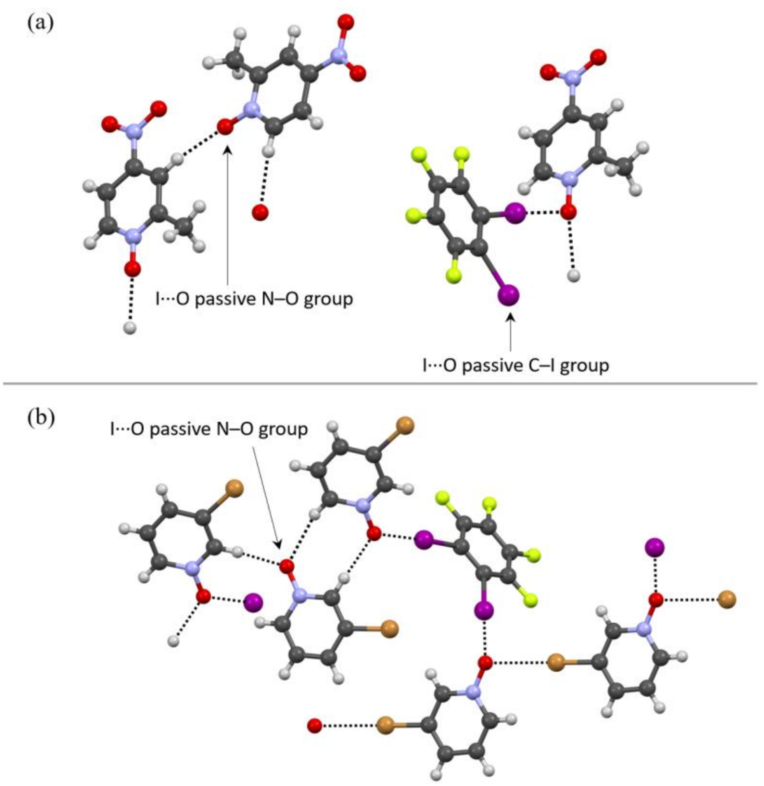

Figure S135. Partial packing structures of (a) **oDIB-19** and (b) **oDIB-28** displaying their I···O passive N-oxide N–O groups. The halogen bond and hydrogen bond are shown by the broken black lines.

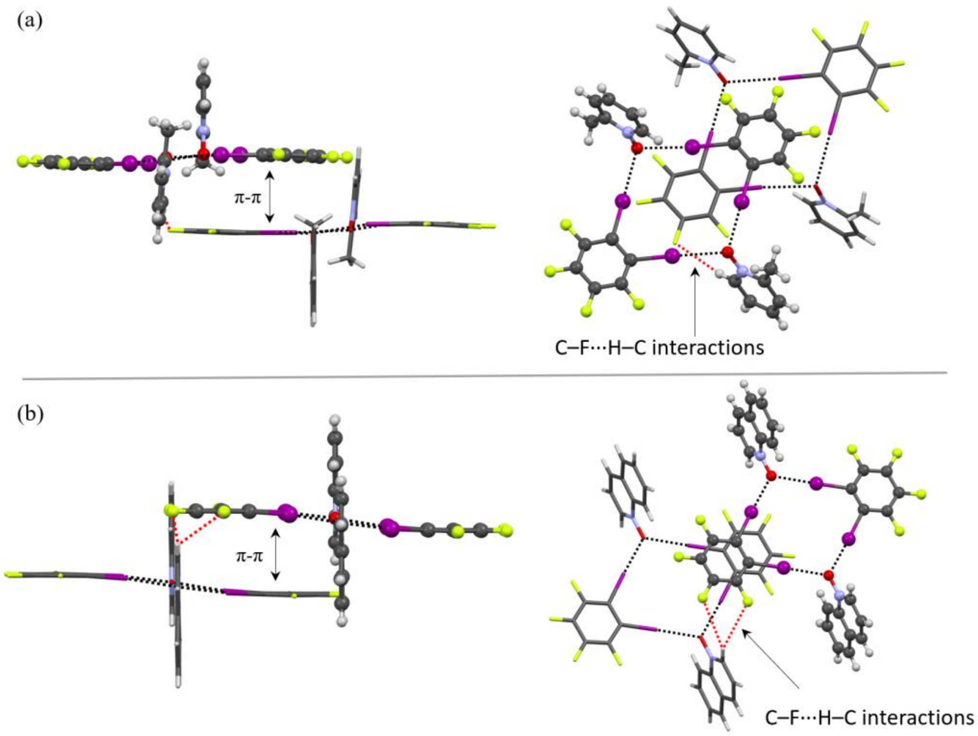

Figure S136. Examples of 2:2 donor:acceptor macrocycle stacking modes showing π-π interactions and (**oDIB**)C–F···H–C(PyNO) HBs are shown in (a) **oDIB-3** (top and side views) and (b) **oDIB-31** (top and side views). The halogen bond is shown by the broken black lines.

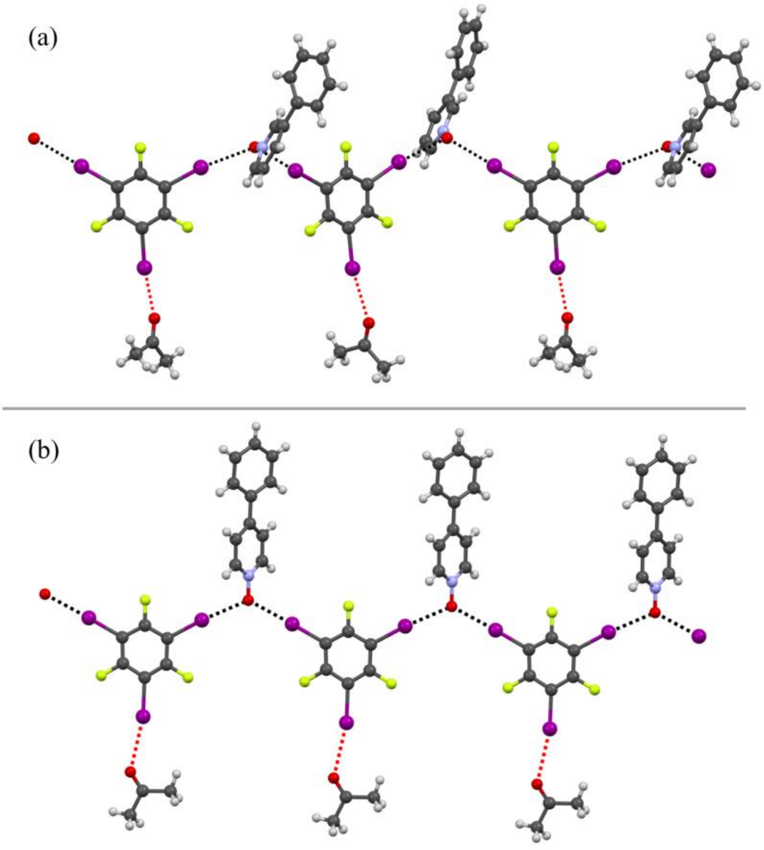

Figure S137. Partial packing structure of **trIB-16** and **trIB-17** showing (**trIB**)C–I···$^{-}$O–N^{+}(PyNO) halogen bonds (black lines) and (**trIB**)C–I···O=C(acetone) halogen bonds (red lines).

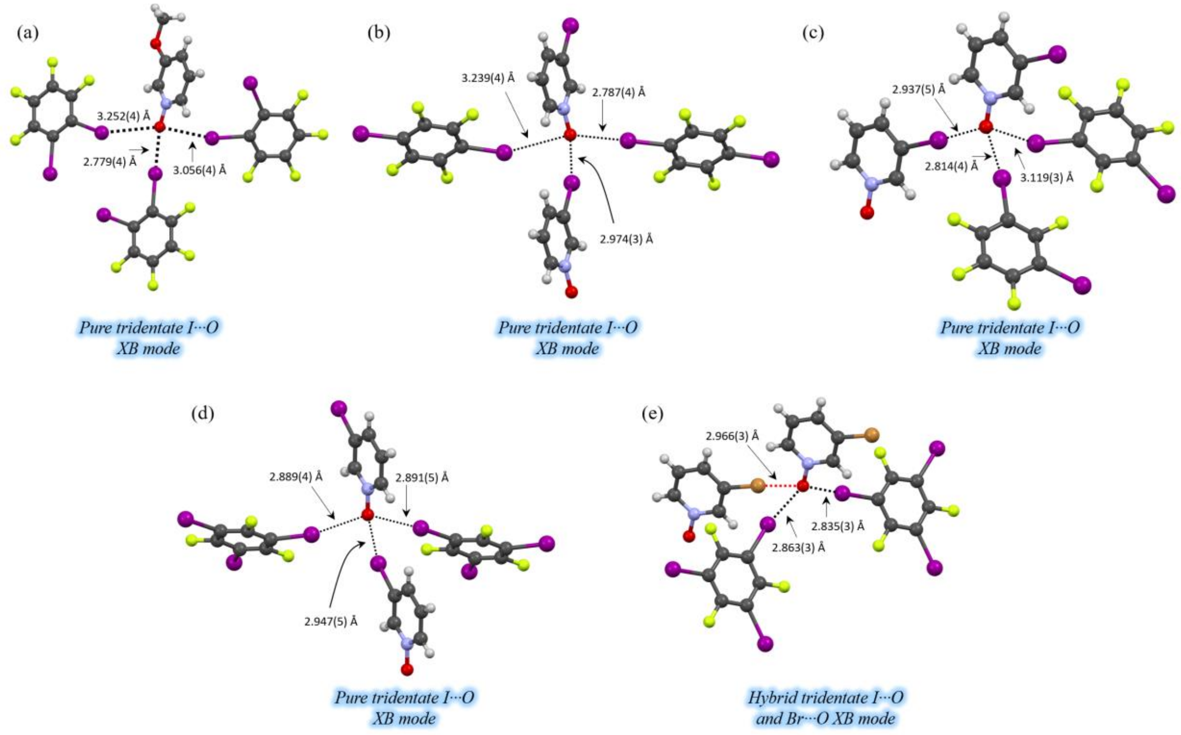

Figure S138. Hybrid tridentate HB-XB coordination modes of (a) **mDIB-1**, (b) **mDIB-2**, (c) **mDIB-7,** (b) **pDIB-1**, and (e) **pDIB-12**. The black broken line represents the XBs and HBs.

(a)

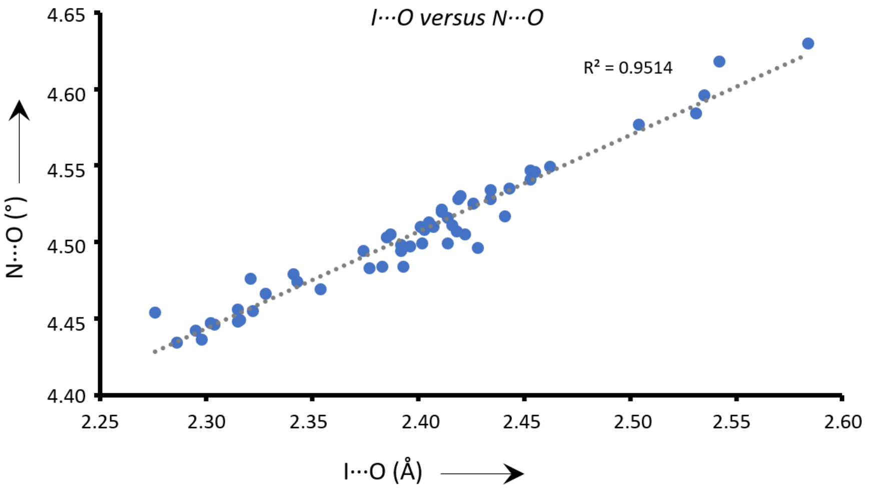

(b)

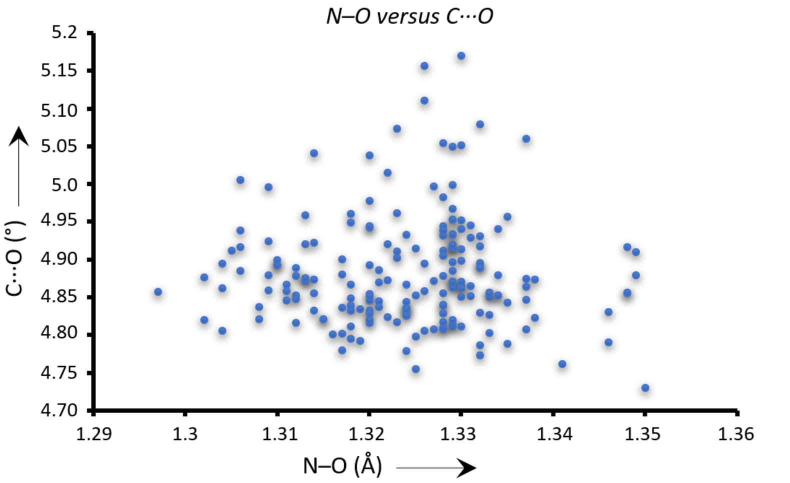

(c)

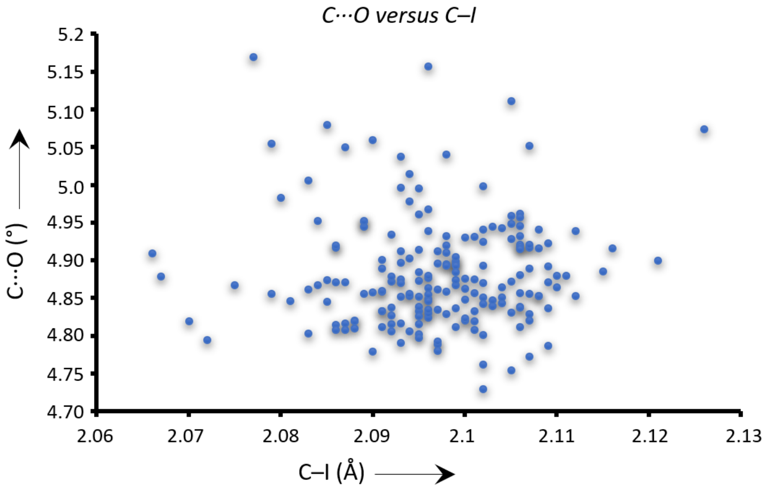

Figure S139. Correlation analysis of crystal structure parameters: (a) N···O versus I···O distances of N–I···$^{-}$O–N^{+} halogen-bonded system. (b) N–O bond lengths versus C···O distances and (c) C–I bond lengths versus C···O distances of C–I···$^{-}$O–N^{+} halogen-bonded system.

(a)

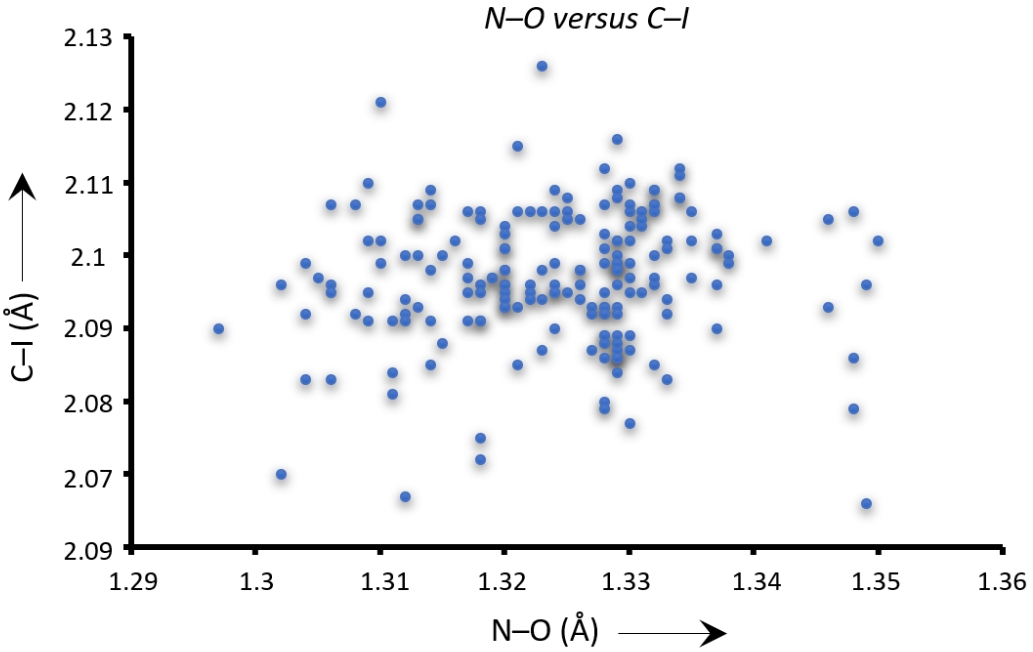

(b)

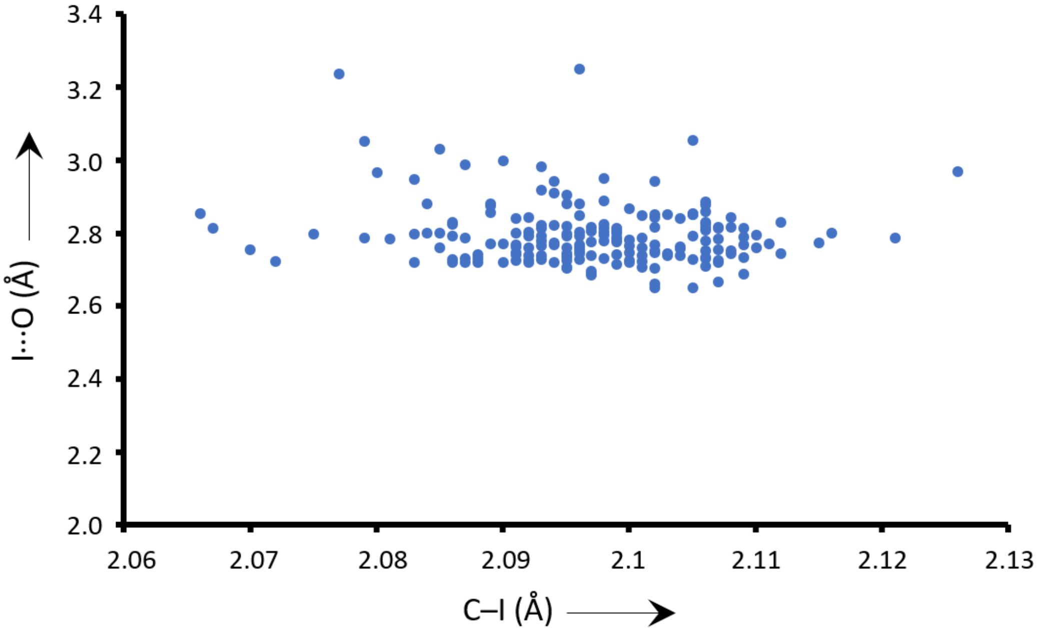

Figure S140. Correlation analysis of crystal structure parameters: (a) N–O versus C–I bond lengths (b) C–I bond lengths versus I···O distances.

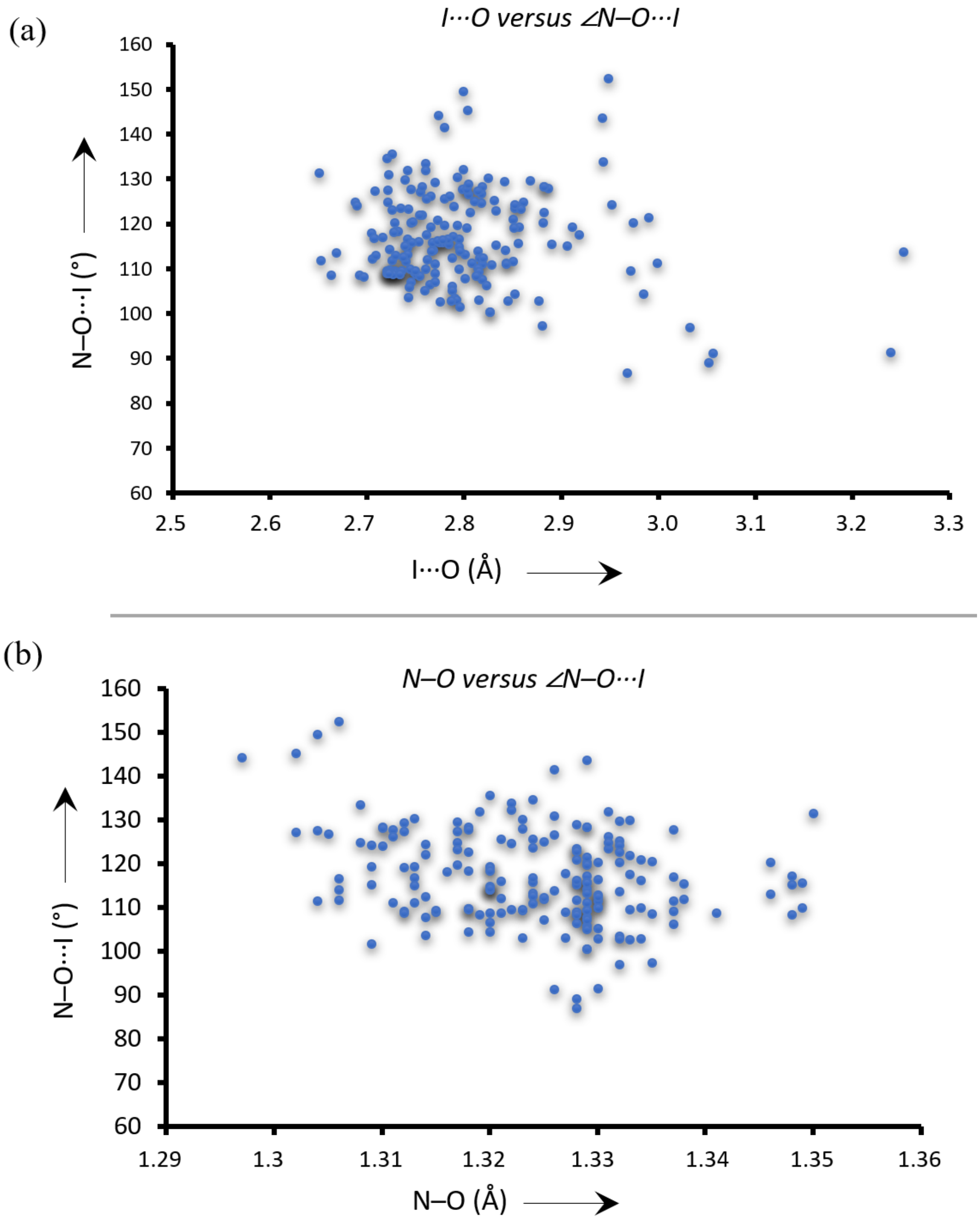

Figure S141. Correlation analysis of crystal structure parameters: (a) I···O distances versus N–O···I angles (b) N–O bond lengths versus N–O···I angles.

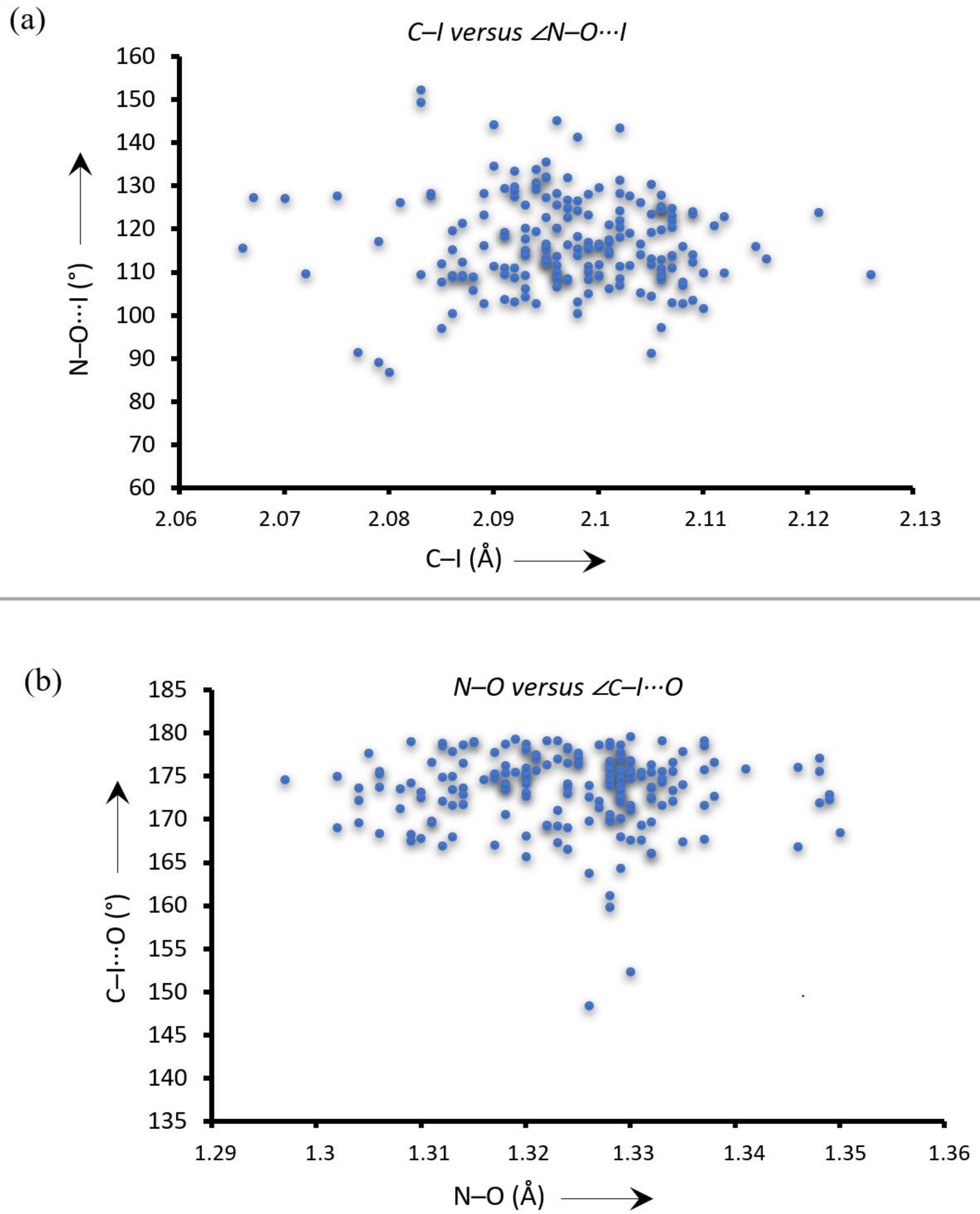

Figure S142. Correlation analysis of crystal structure parameters: (a) C–I bond lengths versus N–O···I angles. (b) N–O bond lengths versus C–I···O angles.

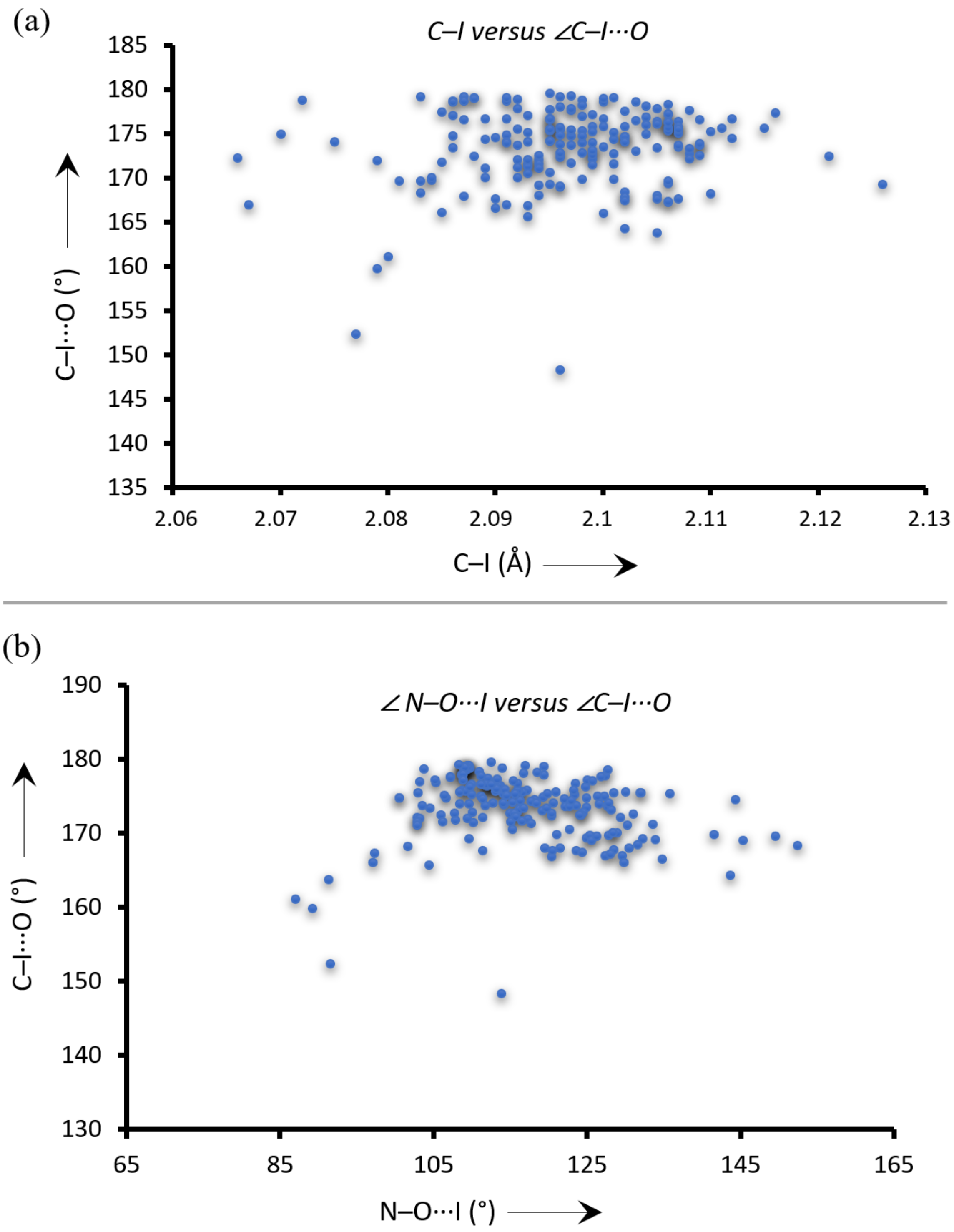

Figure S143. Correlation analysis of crystal structure parameters: (a) C–I bond lengths versus C–I···O angles, (b) N–O···I versus C–I···O angles.

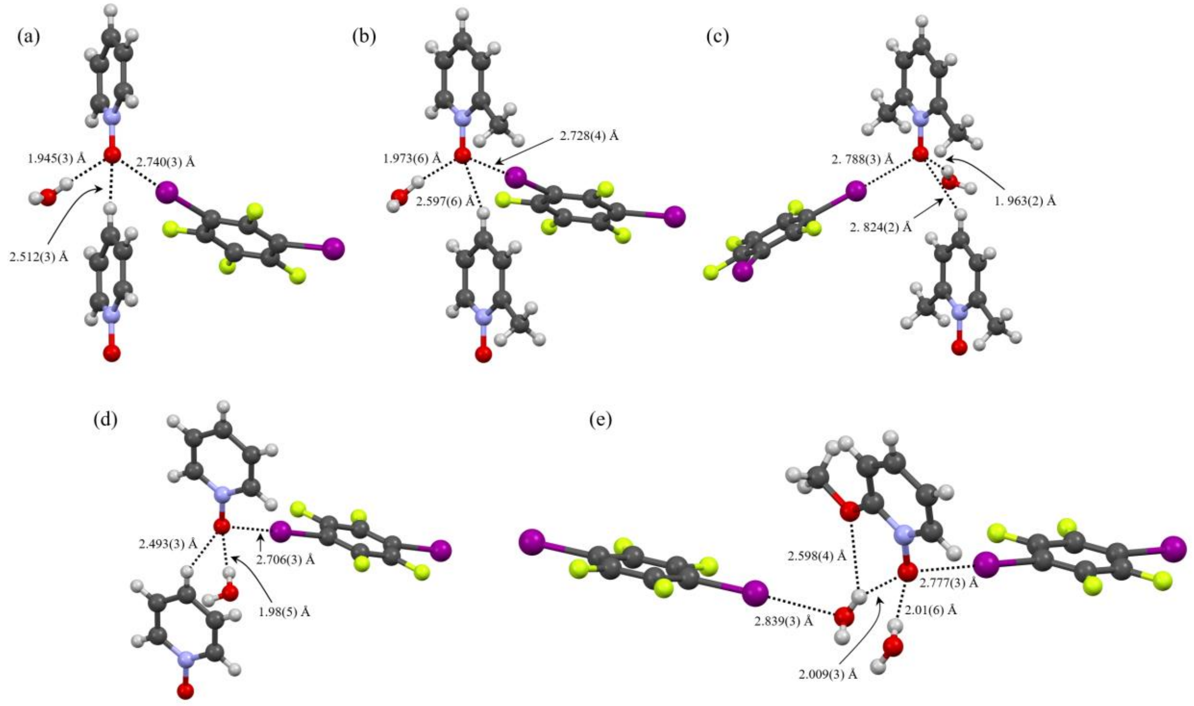

Figure S144. Hybrid tridentate HB-XB coordination modes of (a) **mDIB-1**, (b) **mDIB-2**, (c) **mDIB-7,** (b) **pDIB-1**, and (e) **pDIB-12**. The black broken line represents the XBs and HBs.

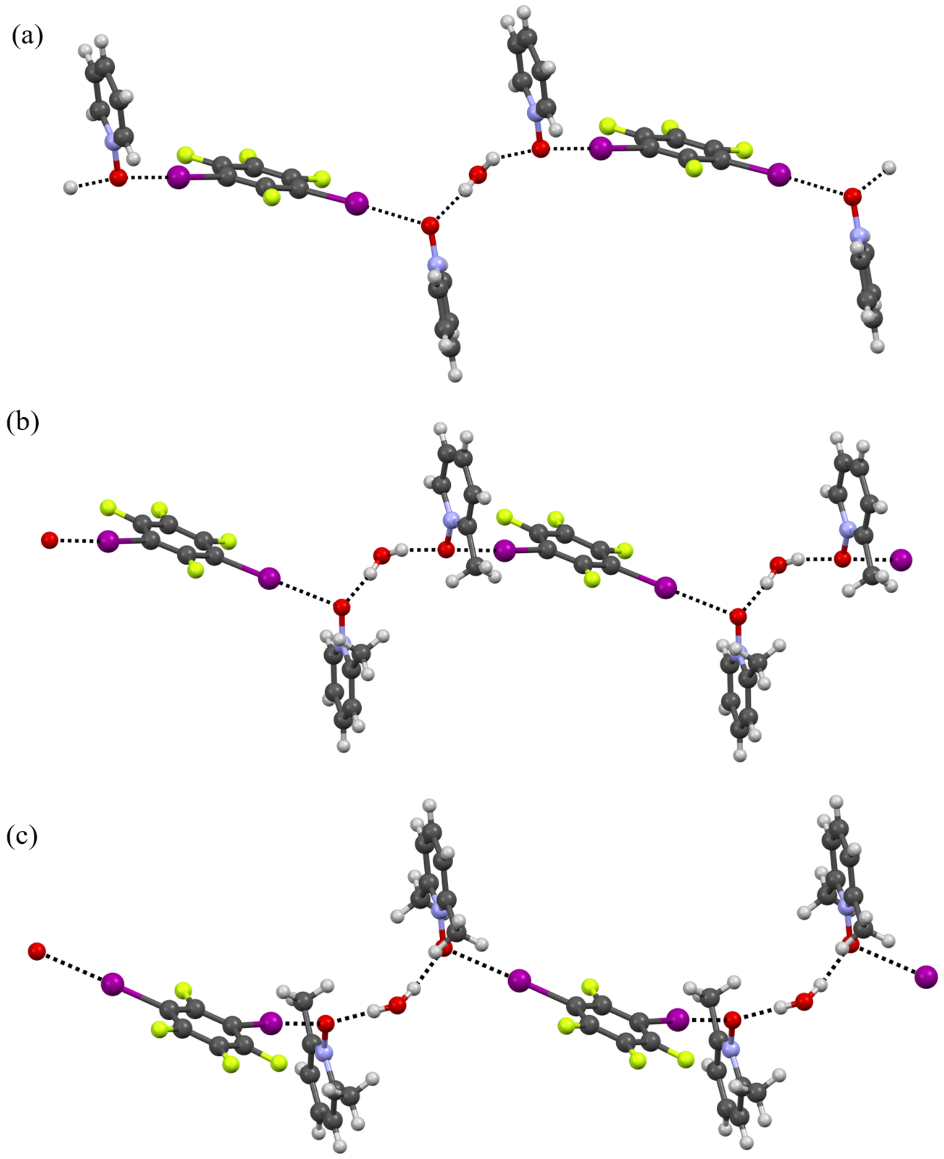

Figure S145. Hydrogen-bonded and halogen-bonded 1D polymeric structures of (a) **mDIB-1**, (b) **mDIB-2**, and (c) **mDIB-7.** The black broken line represents the XBs and HBs.

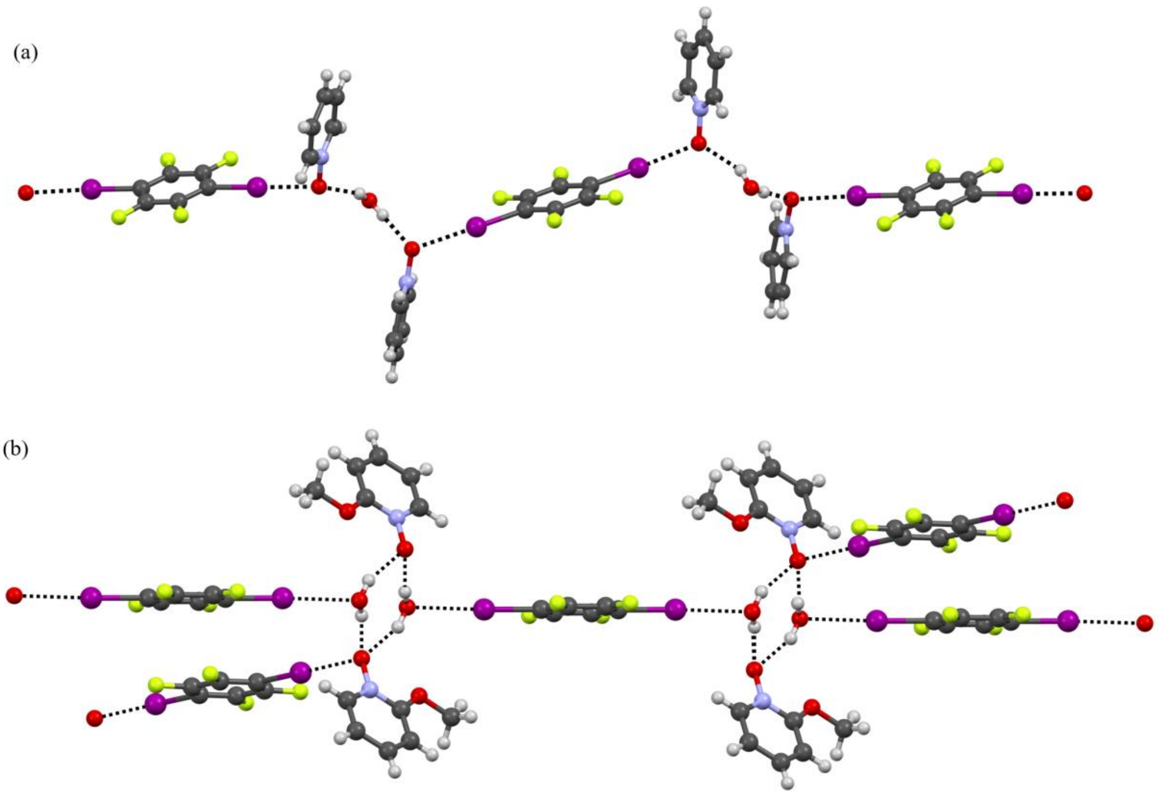

Figure S146. Hydrogen-bonded and halogen-bonded polymeric structures of (a) **pDIB-1**, and (b) **pDIB-12**. The black broken line represents the XBs and HBs.

Weak interactions between iodine and lone-pair containing functional groups such as oxygen of -OCH_3 group are traditionally challenging to characterize in solution. These weak XB electrostatic attractive interactions are presumed to coexist in solution but their XB strengths relative to the pure e.g., C–I···⁻O–N⁺ are unknown. Using PyNOs with broad range of functional groups including -OCH_3, -$NHCH_3$, -NO_2, and halogen groups and crystallizing them with distinct XB donors has provided the opportunity to examine the packing structures in search of interactions other than the relatively strong C–I···⁻O–N⁺ XBs.

In mDIB-**19** and trIB-**30** complexes, the oxygen of -NO_2 groups exhibits similar I···O interactions (ΔE_{int} –15.6 and –16.5 kJ mol^{-1}, respectively) regardless of the type of PyNO *ortho*-substituent, as demonstrated in Figure 14a-b. In trIB-**13** complex 3-methoxy group forms I···O(OCH_3) contact (3.083(2) Å) with ΔE_{int} of –15.3 kJ mol^{-1}. The I···O(OCH_3) contact in oDIB-**12** from 2-methoxy group is longer (3.154(6) Å) suggesting slightly weaker interaction but the ΔE_{int} value could not be determined because close proximity of the PyNO oxide prevented optimization to a minimum structure with I···O(OCH_3) interaction. The Cl, Br, and I substituents of ligands **25-27** are all XB passive and exhibit weak halogen···halogen interactions longer than the sum of the van der Waals radii of the corresponding atoms. The I···I interaction (3.933(2) Å) observed in **pDIB-27** is somewhat below the vdW(I+I) of 3.96 Å. The XB donors also show several I···π interactions; the shortest I···π (3.35 Å) contact appears in **trIB-22**, with an interaction energy of –26.1 kJ mol^{-1}. It is worth mentioning that donor-donor and acceptor-acceptor π-π interactions are a commonly observed phenomena in the bulk of these XB complexes, whereas donor-acceptor π-π interactions are an uncommon trait (For details, See Tables S6-S10).

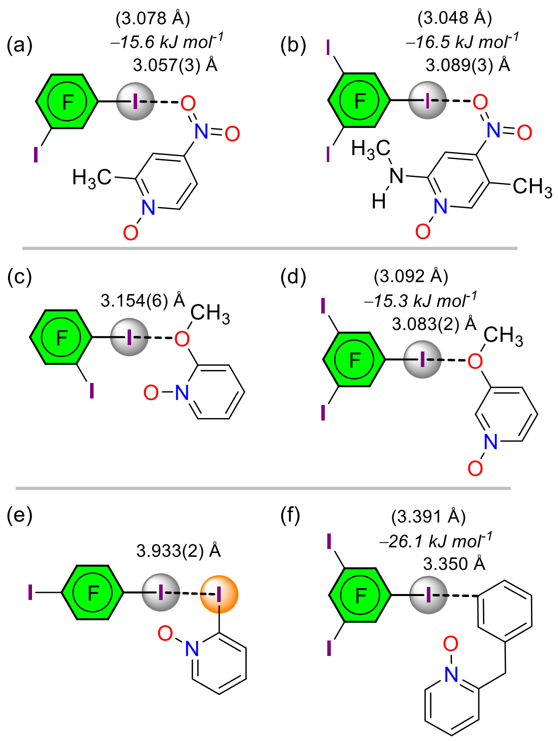

Figure S147. X-ray crystal structures data of non-N-oxide oxygen-based interactions, (a) **mDIB-19**, (b) **trIB-30**, (c) **oDIB-12**, (d) **trIB-13**, (e) **pDIB-27**, and (f) **trIB-22**. ΔE_{int} values are given in italics and optimized structures XB distances in parentheses. Note: All N-oxide oxygens in their complexes are forming I···O interactions and are not shown for viewing clarity. Energy values for the interactions from *ortho*-substituents since the optimized structures prefer C–I···$^-$O–N$^+$ interactions and the short contacts to *ortho* substituents could well be stronger than 5 kJ mol^{-1} but weaker than the C–I···$^-$O–N$^+$ interactions.

Table S6. Summary of π-π interactions* in PfIB-PyNO complexes.

Code	Acceptor-Acceptor	Donor-Acceptor	Donor-Donor
**PfIB-3**	Yes (3.758 Å)	No	Yes (3.901 Å)
**PfIB-8**	Yes (3.809 Å; 3.716 Å)	No	No
**PfIB-13**	Yes (3.535 Å)	Yes (3.657 Å)	No
**PfIB-14**	Yes (3.830 Å)	No	Yes (3.623 Å)
**PfIB-15**	Yes (3.555 Å)	No	Yes (3.949 Å)
**PfIB-16**	Yes (3.802 Å)	No	Yes (3.974 Å)
**PfIB-17**	No	No	No
**PfIB-20**	Yes (3.553 Å)	No	No
**PfIB-31**	No	Yes (3.488 Å; 3.536 Å)	No
**PfIB-32**	Yes (3.624 Å; 3.753 Å)	Yes (3.776 Å; 3.886Å)	Yes (3.70 Å)

*The analysis was carried out using the Olex2 software's "analysis π-π interactions" feature [Olex2 search criteria: Searches for π-π interactions in the structure; if any are found, e.g., the distance between the centroids of adjacent aromatic rings <4.0 Å)].

Table S7. Summary of π-π interactions* in **oDIB**-PyNO complexes.

Code	Acceptor-Acceptor	Donor-Acceptor	Donor-Donor
**oDIB-1**	Yes (3.683 Å)	No	No
**oDIB-2**	Yes (3.694 Å; 3.971 Å)	No	Yes (3.670 Å)
**oDIB-3**	Yes (3.945 Å)	No	Yes (3.801 Å)
**oDIB-4**	Yes (3.699 Å)	No	Yes (3.699 Å; 3.830 Å; 3.988 Å)
**oDIB-4a**	No	No	No
**oDIB-5**	Yes (3.720 Å)	No	Yes (3.722 Å)
**oDIB-6**	Yes (3.771 Å)	No	Yes (3.865 Å)
**oDIB-7**	Yes (3.824 Å)	No	No
**oDIB-8**	Yes (3.657 Å)	No	Yes (3.553 Å; 3.605 Å; 3.947 Å)
**oDIB-9**	Yes (3.885 Å)	No	Yes (3.932 Å)
**oDIB-10**	Yes (3.768 Å)	No	Yes (3.770 Å)
**oDIB-11**	No	No	Yes (3.861 Å)
**oDIB-12**	Yes (3.683 Å)	No	Yes (3.514 Å)
**oDIB-12a**	Yes (3.771 Å)	No	Yes (3.950 Å; 3.995 Å)
**oDIB-12b**	No	No	No
**oDIB-13**	Yes (3.754 Å)	Yes (3.537 Å)	No
**oDIB-14**	No	No	Yes (3.827 Å)
**oDIB-15**	Yes (3.906 Å)	No	Yes (3.780 Å)
**oDIB-15a**	Yes (3.714 Å)	No	Yes (3.872 Å)
**oDIB-17**	Yes (3.728 Å)	Yes (3.649 Å)	Yes (3.875 Å)
**oDIB-18**	Yes (3.595 Å)	No	Yes (3.977 Å; 3.980 Å)
**oDIB-18a**	No	No	No
**oDIB-19**	No	No	Yes (3.887 Å)
**oDIB-20**	No	No	Yes (3.774 Å)
**oDIB-21**	No	No	No
**oDIB-23**	Yes (3.753 Å)	No	No
**oDIB-24**	Yes (3.887 Å)	No	No
**oDIB-25**	No	No	Yes (3.795 Å)
**oDIB-26**	Yes (3.743 Å)	No	Yes (3.680 Å, 3.779 Å; 3.814 Å)
**oDIB-27**	Yes (3.953 Å)	No	Yes (3.704 Å)
**oDIB-28**	Yes (3.480 Å)	No	Yes (4.0 Å)
**oDIB-31**	Yes (4.073 Å)	No	Yes (3.720 Å)
**oDIB-32**	Yes (3.807 Å)	No	Yes (3.671 Å)

*The analysis was carried out using the Olex2 software's "analysis π-π interactions" feature [Olex2 search criteria: Searches for π-π interactions in the structure; if any are found, e.g., the distance between the centroids of adjacent aromatic rings <4.0 Å)].

Table S8. Summary of π-π interactions* in **mDIB**-PyNO complexes.

Code	Acceptor-Acceptor	Donor-Acceptor	Donor-Donor
**mDIB-1**	Yes (3.679 Å)	No	Yes (3.880 Å)
**mDIB-2**	Yes (3.525 Å)	No	Yes (3.619 Å; 3.634 Å)
**mDIB-3**	Yes (3.672 Å; 3.678 Å; 3.733 Å)	No	No
**mDIB-4**	No	No	Yes (3.821 Å; 3.854 Å; 3.992 Å)
**mDIB-6**	Yes (3.624 Å)	No	Yes (3.718 Å)
**mDIB-5**	No	No	Yes (3.913 Å)
**mDIB-7**	Yes (3.589 Å)	No	Yes (3.707 Å)
**mDIB-8**	Yes (3.828 Å)	No	No
**mDIB-9**	No	No	Yes (3.606 Å; 3.808 Å)
**mDIB-11**	Yes (3.741 Å)	No	Yes (3.832 Å)
**mDIB-15**	Yes (3.633 Å)	No	No
**mDIB-19**	No	No	Yes (3.768 Å)
**mDIB-21**	No	No	Yes (3.922 Å)
**mDIB-22**	Yes (3.690 Å)	Yes (3.637 Å)	No
**mDIB-29**	No	No	Yes (3.762 Å)
**mDIB-31**	Yes (3.571 Å)	No	Yes (3.987 Å)
**mDIB-32**	Yes (3.678 Å; 3.714 Å; 3.731 Å)	No	Yes (3.747 Å)

*The analysis was carried out using the Olex2 software's "analysis π-π interactions" feature [Olex2 search criteria: Searches for π-π interactions in the structure; if any are found, e.g., the distance between the centroids of adjacent aromatic rings <4.0 Å)].

Table S9. Summary of π-π interactions* in **pDIB**-PyNO complexes.

Code	Acceptor-Acceptor	Donor-Acceptor	Donor-Donor
**pDIB-1**	Yes (3.643 Å)	No	Yes (3.707 Å)
**pDIB-2**	No	No	Yes (3.733 Å)
**pDIB-3**	No	No	No
**pDIB-4**	No	No	Yes (3.919 Å)
**pDIB-5**	No	No	No
**pDIB-6**	No	No	No
**pDIB-7**	No	No	Yes (3.874 Å; 3.869 Å)
**pDIB-8**	No	No	Yes (3.734 Å; 3.774 Å)
**pDIB-9**	No	No	No
**pDIB-10**	Yes (3.911 Å)	No	Yes (3.673 Å)
**pDIB-11**	Yes (3.724 Å)	No	Yes (3.779 Å)
**pDIB-12**	Yes (3.719 Å)	No	Yes (3.930 Å)
**pDIB-13**	Yes (3.582 Å)	Yes (3.882 Å)	No
**pDIB-14**	Yes (3.636 Å)	No	Yes (3.886 Å; 3.920 Å)
**pDIB-15**	No	No	No
**pDIB-16**	No	No	No
**pDIB-17**	No	Yes (3.737 Å)	No
**pDIB-18**	Yes (3.564 Å)	No	No
**pDIB-18a**	No	No	No
**pDIB-19**	No	No	No
**pDIB-21**	No	No	No
**pDIB-23**	No	No	Yes (3.793 Å)
**pDIB-24**	No	No	No
**pDIB-26**	No	No	No
**pDIB-27**	Yes (3.863 Å)	No	Yes (3.834 Å)
**pDIB-28**	Yes (3.582 Å)	No	Yes (3.820 Å)
**pDIB-29**	No	No	No
**pDIB-31**	Yes (3.504 Å; 3.533 Å)	Yes (3.721 Å; 3.643 Å)	No

*The analysis was carried out using the Olex2 software's "analysis π-π interactions" feature [Olex2 search criteria: Searches for π-π interactions in the structure; if any are found, e.g., the distance between the centroids of adjacent aromatic rings <4.0 Å)].

Table S10. Summary of π-π interactions* in **trIB**-PyNO complexes.

Code	Acceptor-Acceptor	Donor-Acceptor	Donor-Donor
**trIB-1**	No	No	Yes (3.713 Å)
**trIB-2**	No	No	Yes (3.878 Å)
**trIB-3**	No	No	Yes (3.974 Å)
**trIB-4**	Yes (3.804 Å)	No	Yes (3.760 Å)
**trIB-5**	No	No	Yes (3.928 Å)
**trIB-6**	No	No	No
**trIB-7**	Yes (3.553 Å)	No	Yes (3.831 Å)
**trIB-8**	Yes (3.589 Å)	No	Yes (3.776 Å)
**trIB-9**	Yes (3.553 Å)	No	Yes (3.831 Å)
**trIB-11**	No	No	No
**trIB-12**	Yes (3.753 Å)	No	Yes (3.887 Å)
**trIB-13**	Yes (3.520 Å)	No	Yes (3.852 Å)
**trIB-15**	No	No	Yes (3.761 Å)
**trIB-16**	No	No	Yes (3.730 Å; 3.897 Å)
**trIB-17**	Yes (3.744 Å)	No	No
**trIB-18**	No	No	Yes (3.671 Å)
**trIB-20**	Yes (3.655 Å)	No	Yes (3.711 Å; 3.721 Å)
**trIB-21**	No	No	No
**trIB-22**	Yes (3.555 Å)	No	Yes (3.737 Å)
**trIB-28**	Yes (3.656 Å)	No	No
**trIB-29**	Yes (3.686 Å)	No	No
**trIB-30**	Yes (3.444 Å)	No	Yes (3.670 Å)
**trIB-32**	Yes (3.657 Å)	No	Yes (3.782 Å)

*The analysis was carried out using the Olex2 software's "analysis π-π interactions" feature [Olex2 search criteria: Searches for π-π interactions in the structure; if any are found, e.g., the distance between the centroids of adjacent aromatic rings <4.0 Å)].

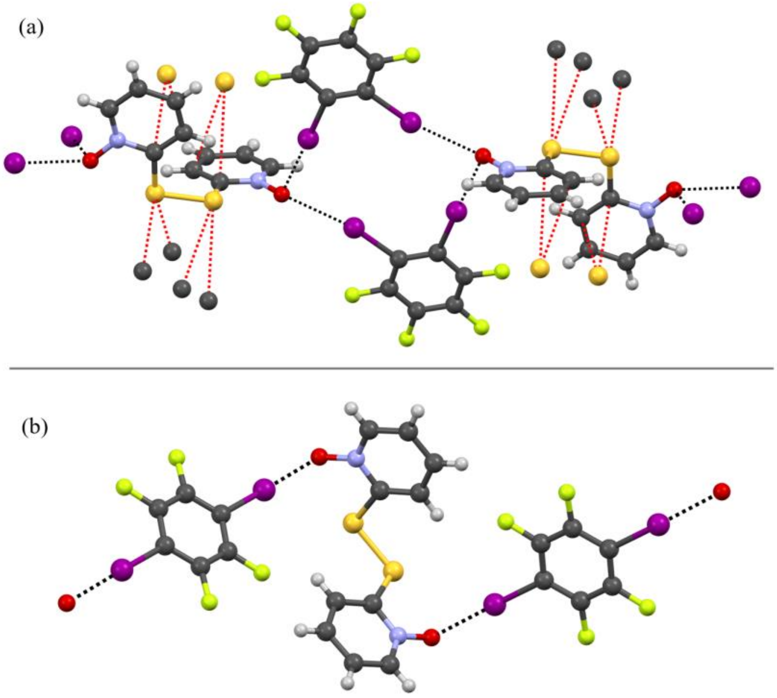

Figure S148. Partial packing structure of (a) **oDIB-18a** and (b) **pDIB-18** showing C–I···⁻O–N⁺ halogen bonds (black lines) and S··· C contacts (red lines).

3. Density Functional Theory studies

To be consistent with our earlier halogen bonding studies on PyNO systems[7,8] all DFT calculations were carried out with PBE0 hydrid functional[9–11] employing def2-TZVP basis sets[12,13] and treating dispersion interactions with empirical D3 model by Grimme that includes Becke-Johnson dampening.[14,15] Counterpoise method was used to derive basis set superposition error corrected complexation energies[16]. Gaussian 16 program package[17] was used for all DFT calculations. Energy scans for the XB interaction energy, ΔE_{int} in **PfIB**-PyNO complex were carried out by using the optimized structure of the complex as starting point and varying the N-O···I angle between 65 and 180 ° and C-N-O···I dihedral angle from 0 to 355 ° in five-degree intervals while keeping the other structural parameters fixed. The results are presented in Figures 11a-b in the main text. Similarly, the ΔE_{int} energies were scanned as a function of changes in σ-angles between 120 and 180 ° in five-degree intervals and XB distances between 1.96 and 4.58 Å in 0.025 Å intervals, respectively, and the information is combined in Figure 11d in the main text.

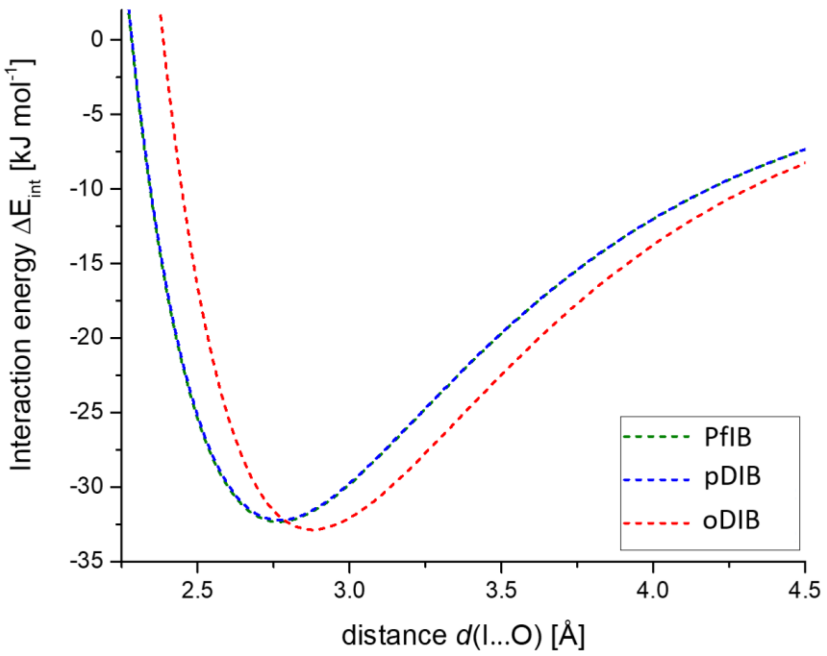

Figure S149. Interaction energy, ΔE_{int} scans as a function of I···O distance calculated at PBE0-D3/def2-TZVP level of theory for **PfIB**-PyNO**, pDIB**-PyNO, and **oDIB**-PyNO complexes.

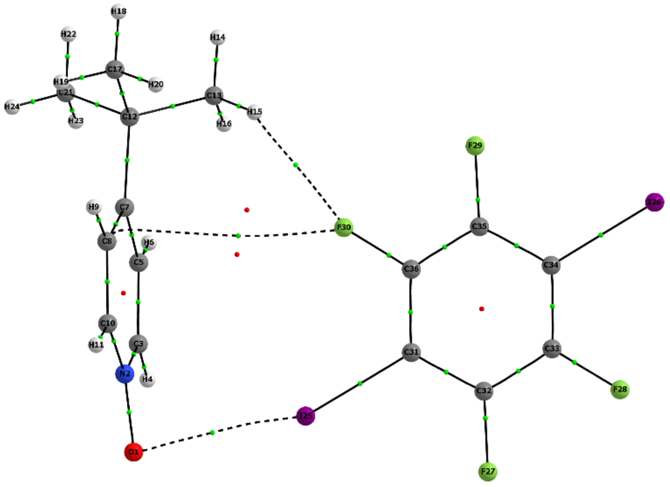

Figure S150. QTAIM analysis showing C–I···$^{-}$O–N^{+} XBs of **pDIB-21**.

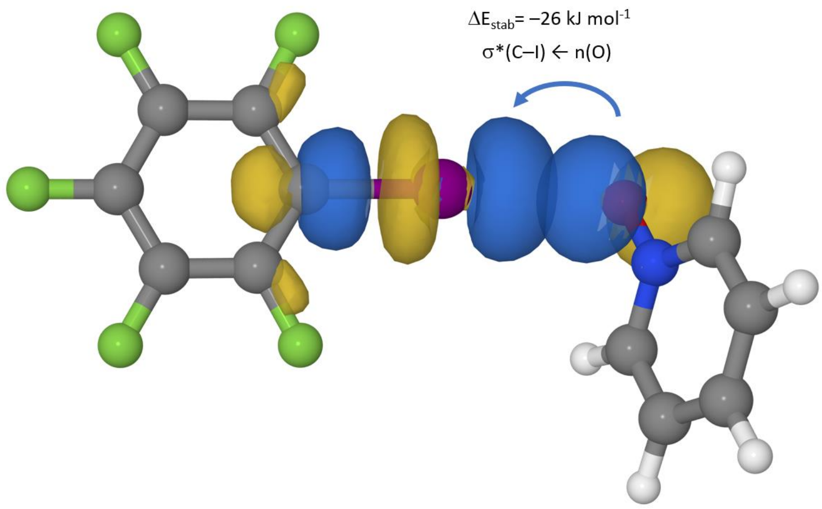

Figure S151. Superimposed NBO orbitals of **PfIB** and **1**.

4. NMR and Raman studies

4.1 General information

^{15}N NMR measurement: The ^{15}N NMR spectra was acquired using a Bruker Avance III 500 MHz spectrometer equipped with prodigy probe. **PfIB-9** was by mixing 1:1 eq of **PfIB** and **9** in $CDCl_3$. External ^{15}N reference used was 90% ^{15}N enriched nitromethane in a sealed tube. The ^{15}N spectra was measured with one scan/increment. The signal was calibrated to 0 ppm, and the **9** and **PfIB-9** were measured using the same parameters. Sine bell window function with zero shifts was used for apodization before Fourier transformation, and both the measurements were made using 64 scans.

^{19}F NMR measurement: The titrations were performed in $CDCl_3$ at the temperature using Bruker Avance III 300 MHz spectrometer. In an NMR tube, a 10 mM sample solution (volume 600 µL) of **PfIB** was prepared, and the titrations were performed up to 4.5-5.0 equivalents of the guest added, using 0.5 M stock solutions of **9** dissolved in $CDCl_3$.

Raman Measurement: Raman measurements were carried out using a Thermo Scientific DXR Raman Microscope equipped with a 532 nm laser at 10 mW of power and a 10x microscope objective, resulting in a 2 µm spot size. The Raman measurements were carried out using 2.0s of exposure.

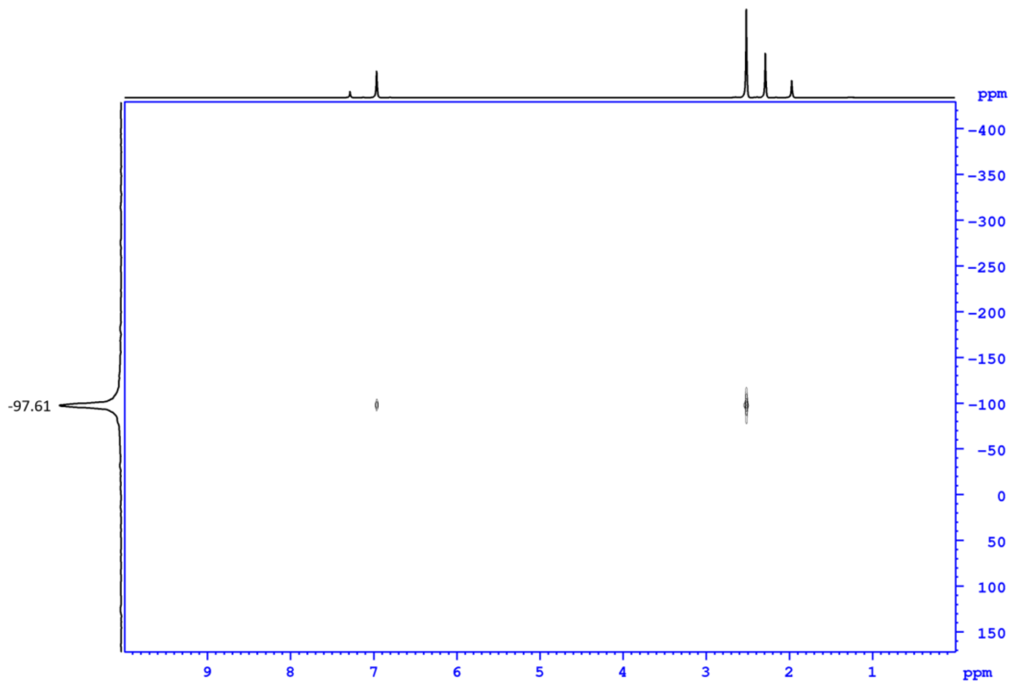

Figure S152. $^{1}H,^{15}N$ HMBC spectra of **9** in $CDCl_3$

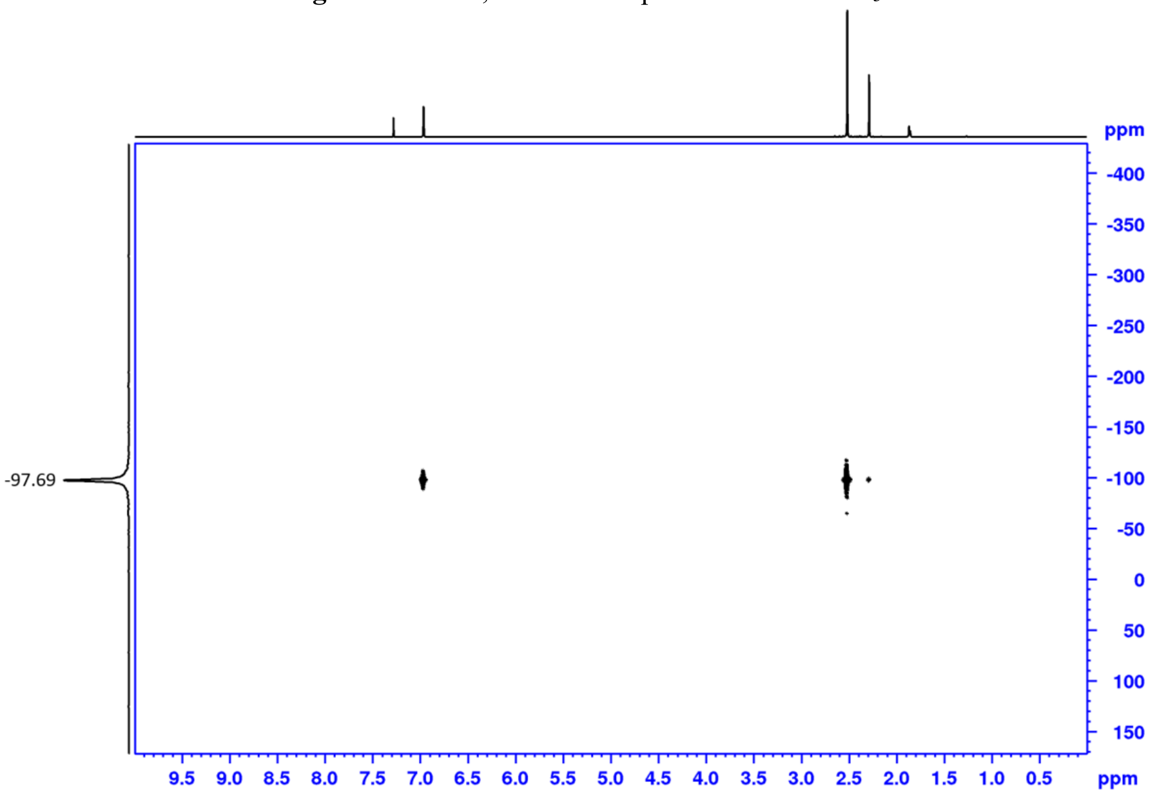

Figure S153. $^{1}H,^{15}N$ HMBC spectra of **pDIB-9** in $CDCl_3$

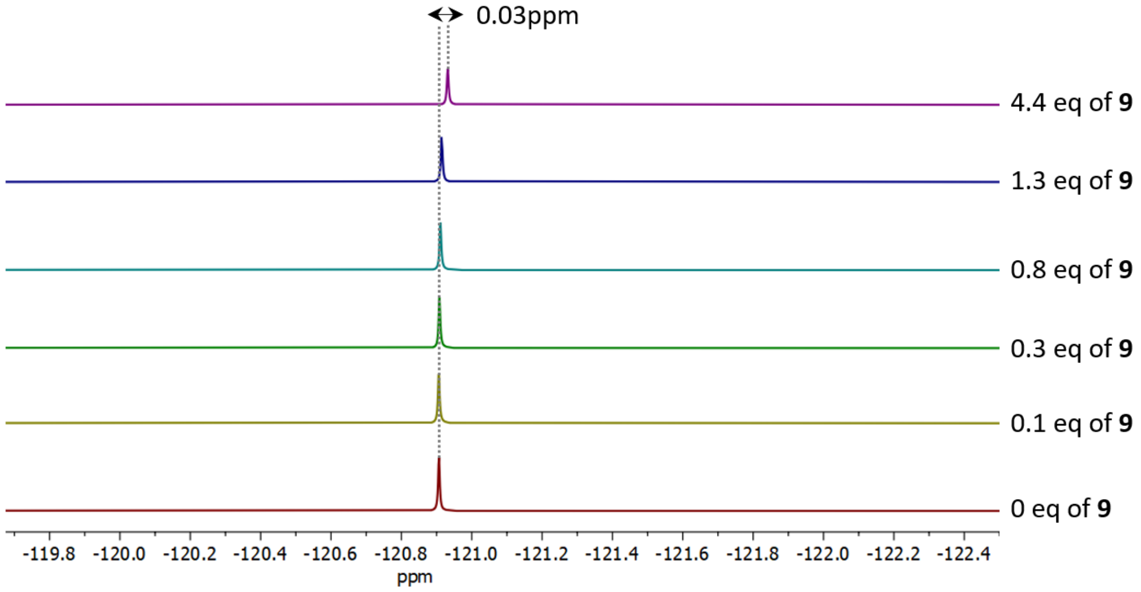

Figure S154. The selected ^{19}F NMR region stack spectra of **pDIB-9** in $CDCl_3$

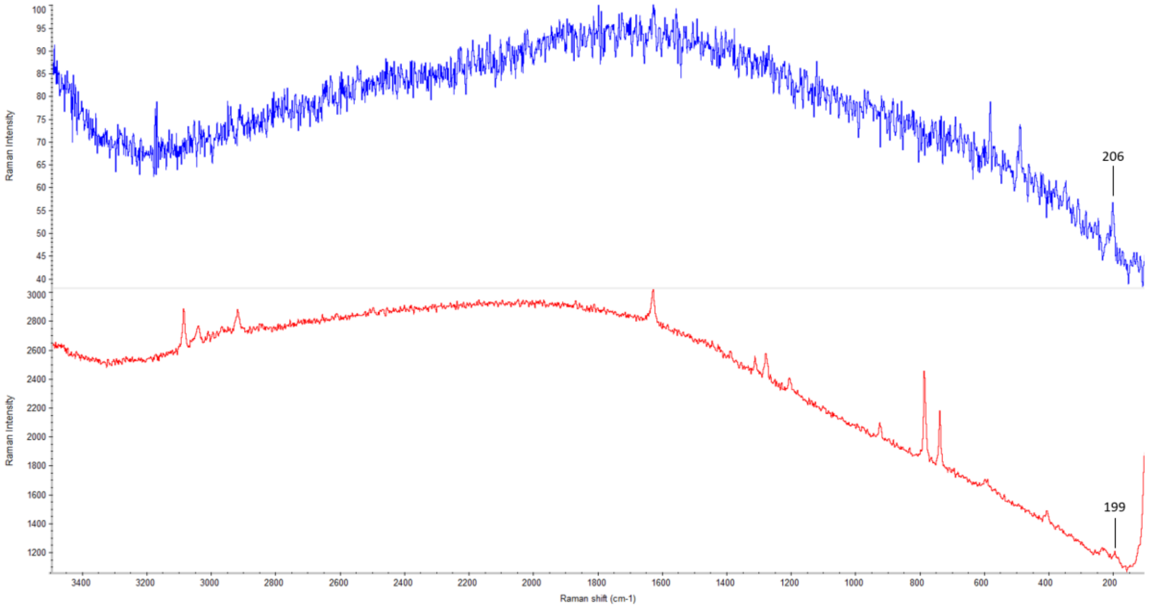

Figure S155. Raman stack spectra of **PfIB** (blue trace) and **PfIB-20** (red trace) from 100 to 3500 cm^{-1}.

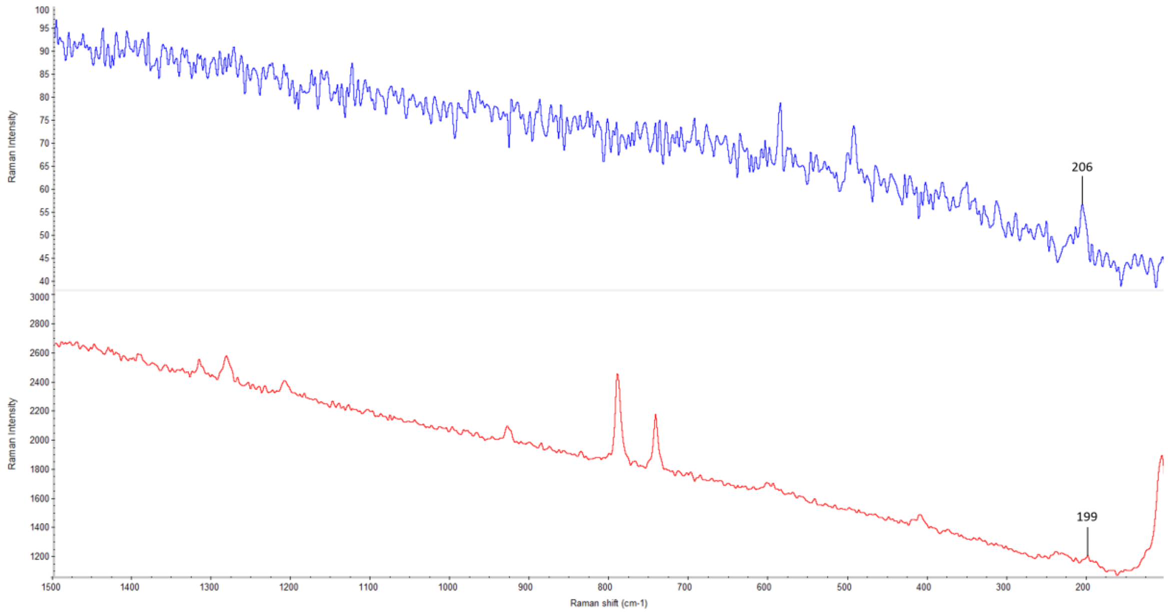

Figure S156. Raman stack spectra of **PfIB** (blue trace) and **PfIB-20** (red trace) from 100 to 500 cm^{-1}.

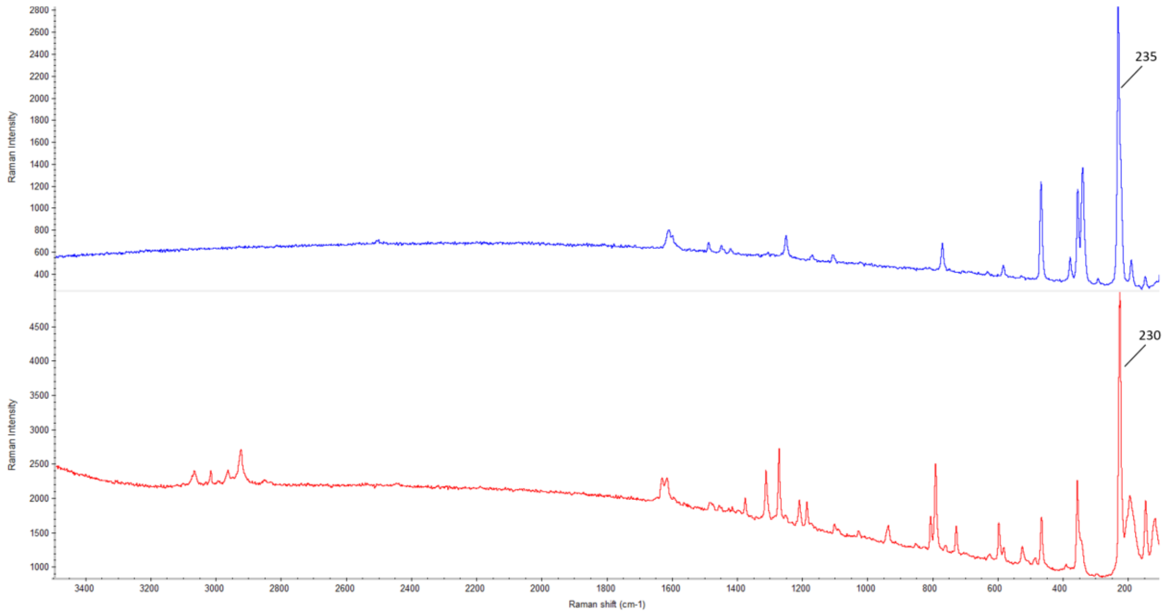

Figure S157. Raman stack spectra of **oDIB** (blue trace) and **oDIB-20** (red trace) from 100 to 3500 cm^{-1}.

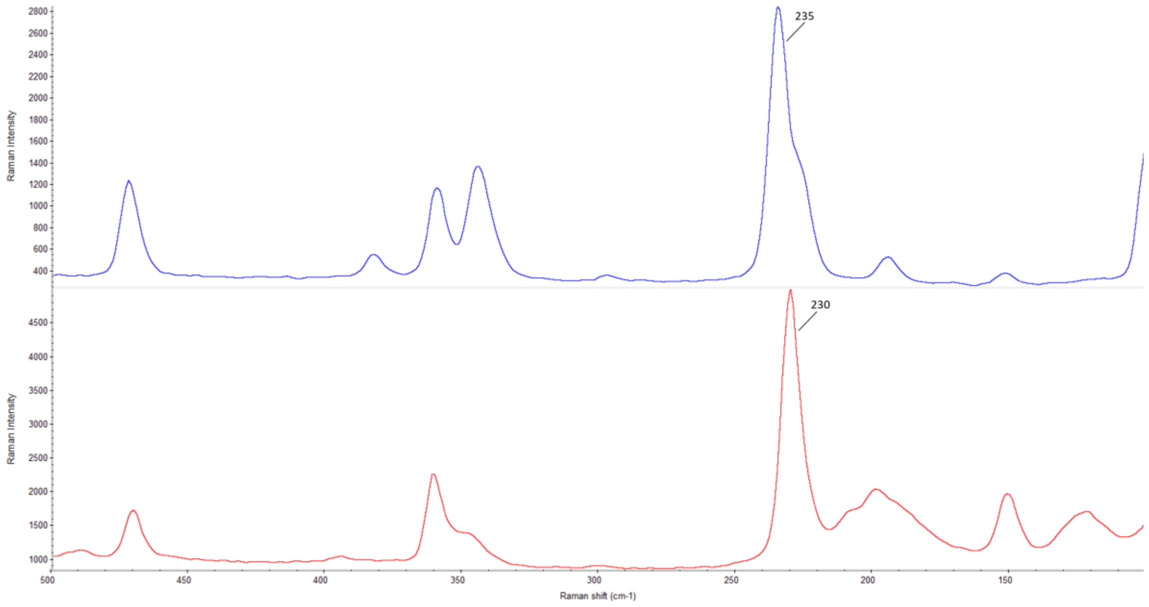

Figure S158. Raman stack spectra of **oDIB** (blue trace) and **oDIB-20** (red trace) from 100 to 500 cm^{-1}.

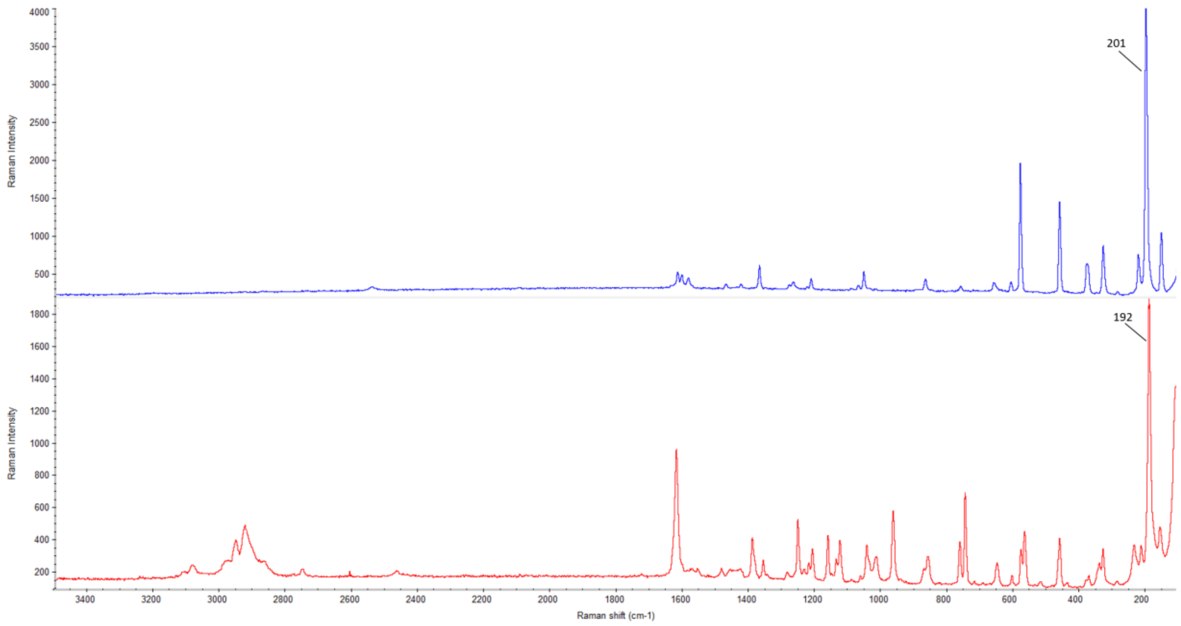

Figure S159. Raman stack spectra of **mDIB** (blue trace) and **mDIB-8** (red trace) from 100 to 3500 cm^{-1}.

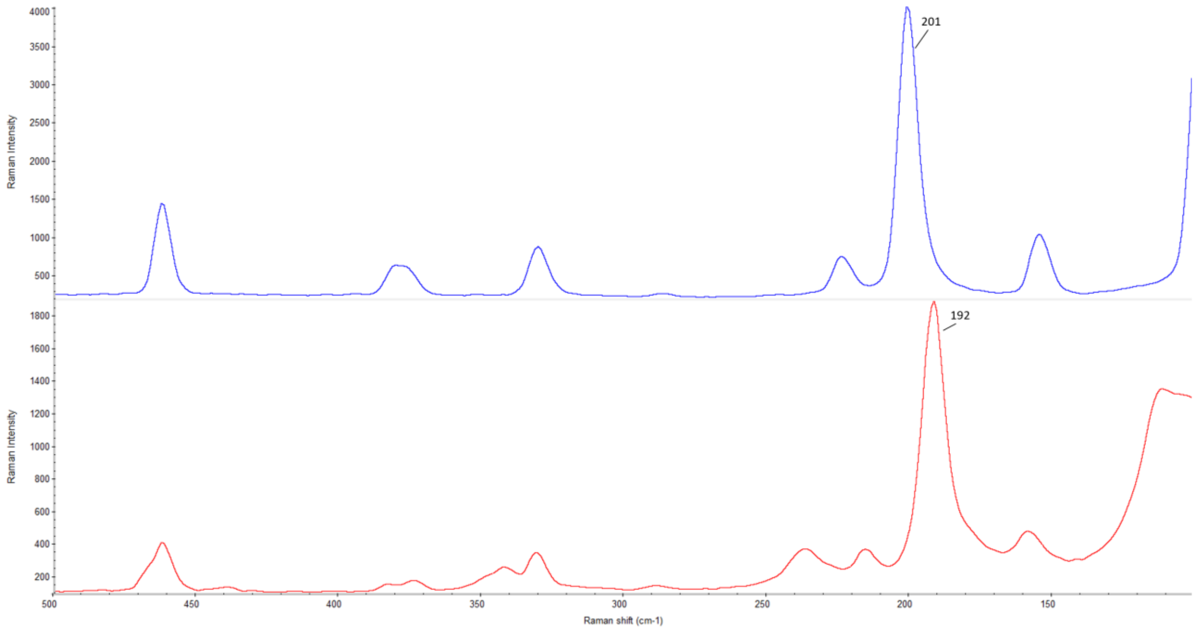

Figure S160. Raman stack spectra of **mDIB** (blue trace) and **mDIB-8** (red trace) from 100 to 500 cm^{-1}.

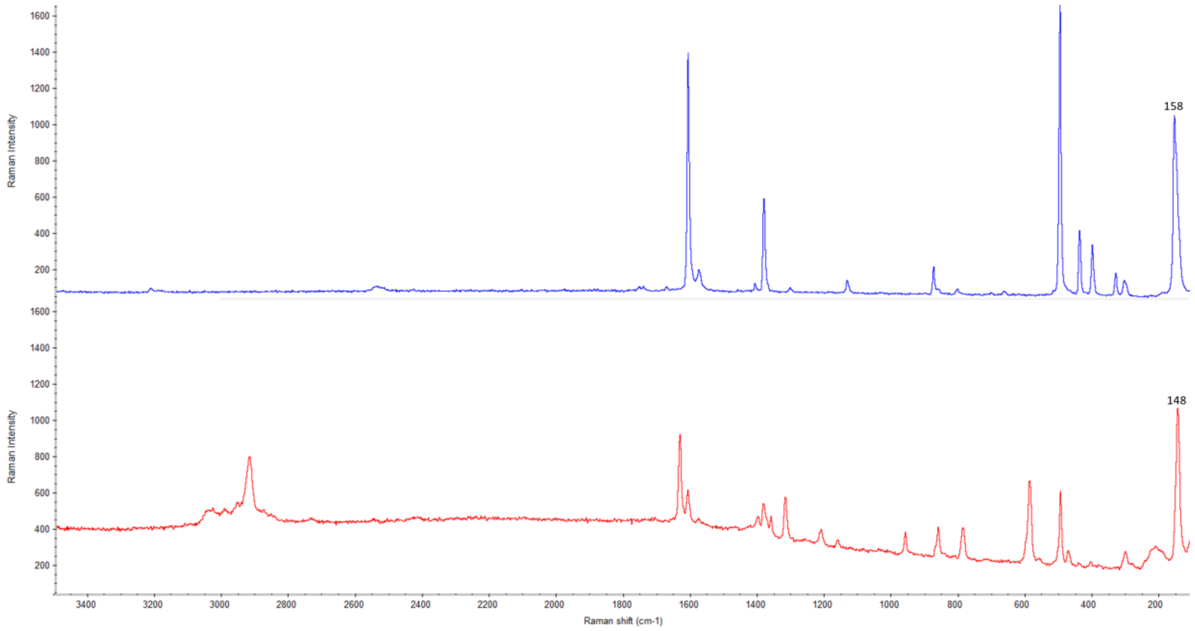

Figure S161. Raman stack spectra of **pDIB** (blue trace) and **pDIB-9** (red trace) from 100 to 3500 cm^{-1}.

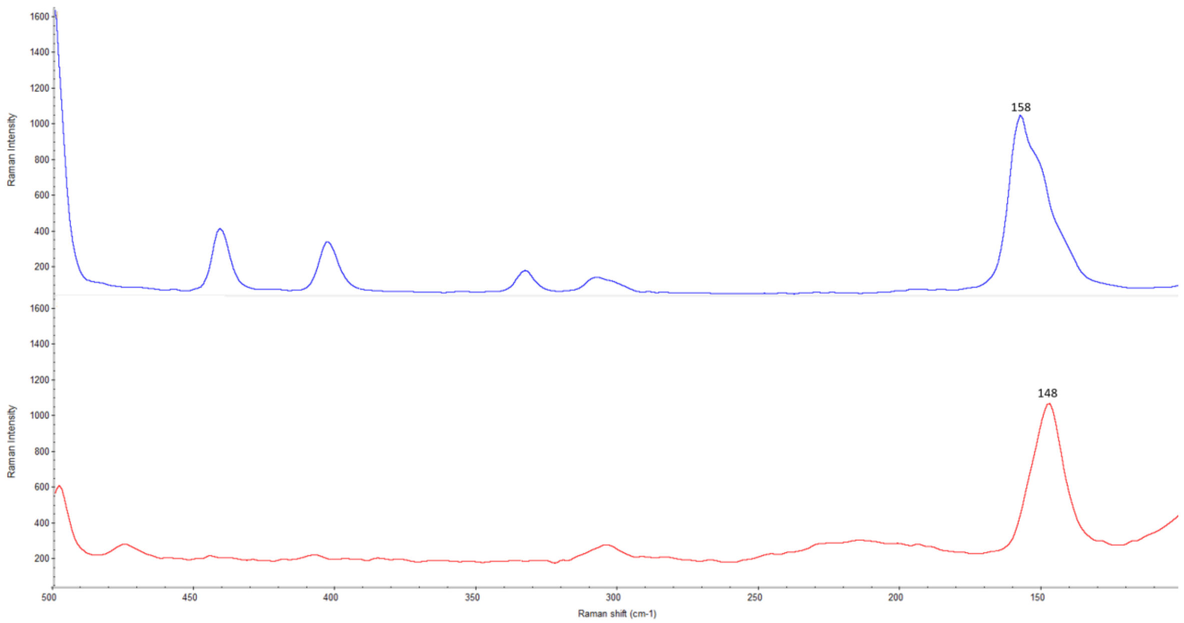

Figure S162. Raman stack spectra of **pDIB** (blue trace) and **pDIB-9** (red trace) from 100 to 500 cm^{-1}.

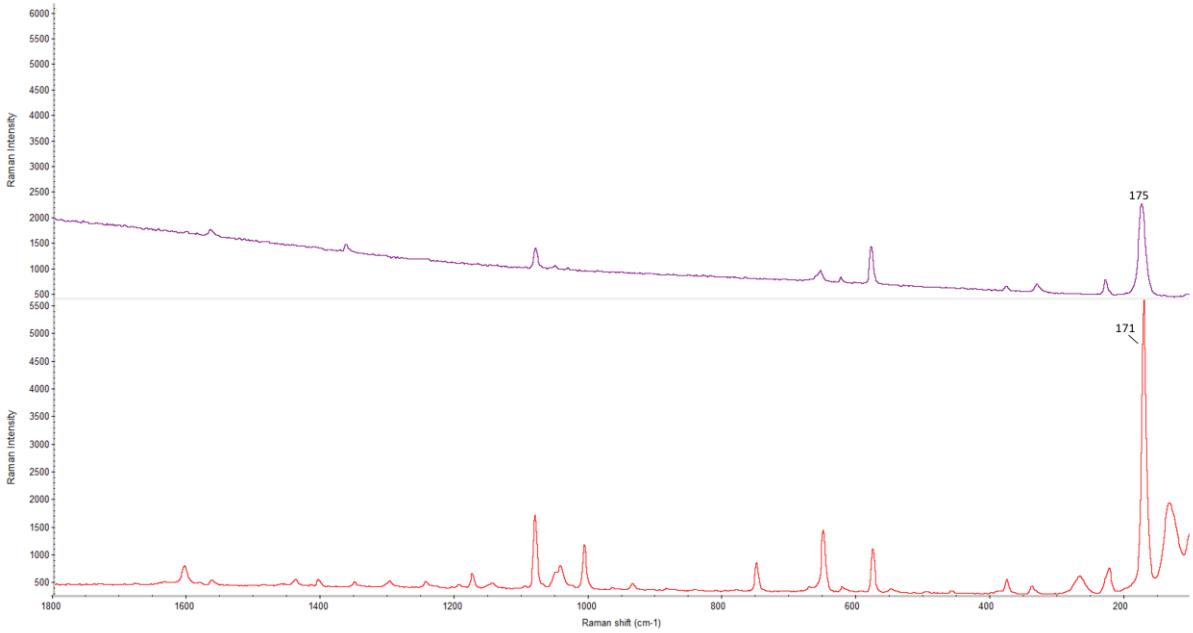

Figure S163. Raman stack spectra of **trIB** (blue trace) and **trIB-13** (red trace) from 100 to 1800 cm^{-1}.

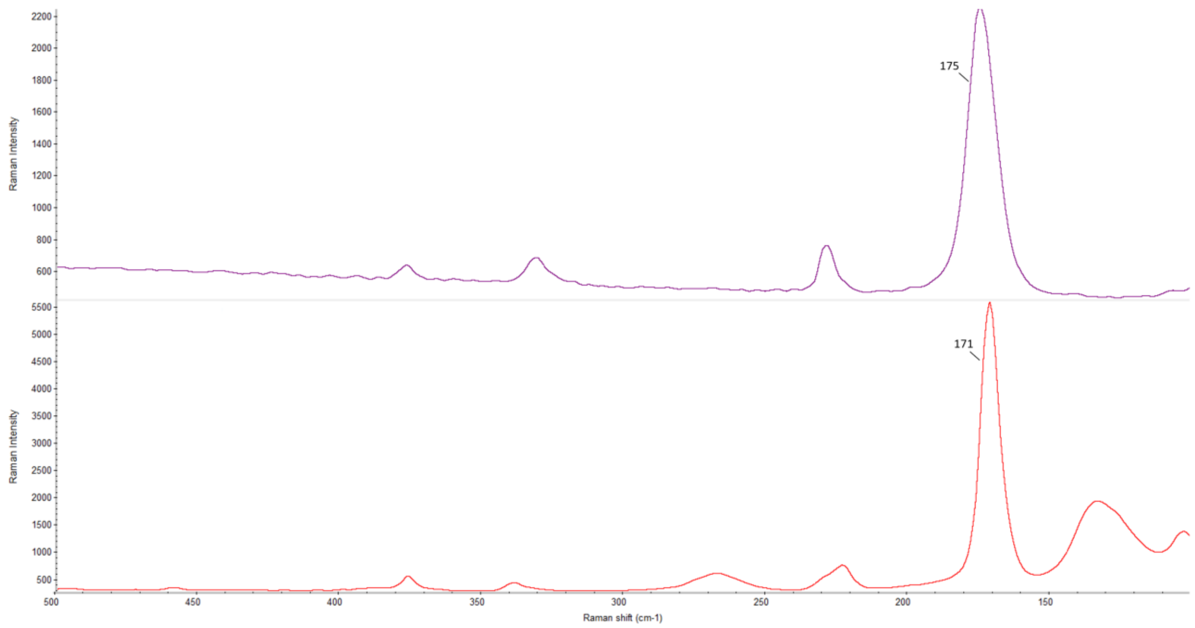

Figure S164. Raman stack spectra of **trIB** (blue trace) and **trIB-13** (red trace) from 100 to 3500 cm^{-1}.

5. References

[1] M. J. Tanga, J. E. Bupp, T. K. Tochimoto, *Journal of Heterocyclic Chemistry* **1997**, *34*, 717–727.

[2] Z. Otwinowski, W. Minor, in *Methods Enzymol* (Ed.: Jr.B.T.-M. in E. Charles W. Carter), Academic Press, **1997**, 307–326.

[3] G. M. Sheldrick, *Acta Crystallogr A* **2015**, *71*, 3-8.

[4] O. V. Dolomanov, L. J. Bourhis, R. J. Gildea, J. A. K. Howard, H. Puschmann, *Journal of Applied Crystallographica* **2009**, *42*, 339–341.

[5] G. M. Sheldrick, *Acta Crystallographica A* **2008**, *64*, 112–122.

[6] G. M. Sheldrick, *Acta Crystallographica C Structural Chemistry* **2015**, *71*, 3–8.

[7] R. Puttreddy, M. Rautiainen, S. Yu, K. Rissanen, *Angewandte Chemie International Edition* **2023**, DOI 10.1002/anie.202307372.

[8] R. Puttreddy, J. M. Rautiainen, T. Mäkelä, K. Rissanen, *Angewandte Chemie - International Edition* **2019**, *58*, 18610–18618.

[9] J. P. Perdew, K. Burke, M. Ernzerhof, *Physical Review Letters* **1996**, *77*, 3865–3868.

[10] J. P. Perdew, K. Burke, M. Ernzerhof, *Physical Review Letters* **1997**, *78*, 1396.

[11] C. Adamo, V. Barone, *Journal of Chemical Physics* **1999**, *110*, 6158–6170.

[12] F. Weigend, R. Ahlrichs, *Physical Chemistry Chemical Physics* **2005**, *7*, 3297–3305.

[13] F. Weigend, M. Häser, H. Patzelt, R. Ahlrichs, *Chemical Physical Letters* **1998**, *294*, 143–152.

[14] S. Grimme, J. Antony, S. Ehrlich, H. Krieg, *Journal of Chemical Physics* **2010**, *132*, DOI 10.1063/1.3382344.

[15] S. Grimme, S. Ehrlich, L. Goerigk, *Journal of Computational Chemistry* **2011**, *32*, 1456–1465.

[16] S. F. Boys, F. Bernardi, *Molecular Physics* **1970**, *19*, 553–566.

[17] Gaussian 16, Revision C.01, M. J. Frisch, G. W. Trucks, H. B. Schlegel, G. E. Scuseria, M. A. Robb, J. R. Cheeseman, G. Scalmani, V. Barone, G. A. Petersson, H. Nakatsuji, X. Li, M. Caricato, A. V Marenich, J. Bloino, B. G. Janesko, R. Gomperts, B. Mennucci, H. P. Hratchian, J. V Ortiz, A. F. Izmaylov, J. L. Sonnenberg, D. Williams-Young, F. Ding, F. Lipparini, F. Egidi, J. Goings, B. Peng, A. Petrone, T. Henderson, D. Ranasinghe, V. G. Zakrzewski, J. Gao, N. Rega, G. Zheng, W. Liang, M. Hada, M. Ehara, K. Toyota, R. Fukuda, J. Hasegawa, M. Ishida, T. Nakajima, Y. Honda, O. Kitao, H. Nakai, T. Vreven, K. Throssell, J. A. Montgomery Jr., J. E. Peralta, F. Ogliaro, M. J. Bearpark, J. J. Heyd, E. N. Brothers, K. N. Kudin, V. N. Staroverov, T. A. Keith, R. Kobayashi, J. Normand, K. Raghavachari, A. P. Rendell, J. C. Burant, S. S. Iyengar, J. Tomasi, M. Cossi, J. M. Millam, M. Klene, C. Adamo, R. Cammi, J. W. Ochterski, R. L. Martin, K. Morokuma, O. Farkas, J. B. Foresman, D. J. Fox, Gaussian, Inc., Wallingford CT, **2016**.
